# Diabetic retinopathy risk in patients with unhealthy lifestyle: A Mendelian randomization study

**DOI:** 10.3389/fendo.2022.1087965

**Published:** 2023-01-17

**Authors:** Zixuan Su, Zhixin Wu, Xueqing Liang, Meng Xie, Jia Xie, Huiqing Li, Xinghua Wang, Fagang Jiang

**Affiliations:** ^1^ Department of Ophthalmology, Union Hospital, Tongji Medical College, Huazhong University of Science and Technology, Wuhan, China; ^2^ Department of Endocrinology, Union Hospital, Tongji Medical College, Huazhong University of Science and Technology, Wuhan, China; ^3^ Department of Biostatistics, School of Public Health, Southern Medical University, Guangzhou, China

**Keywords:** Mendelian randomization, diabetic retinopathy, unhealthy lifestyle factors, obesity, smoking, alcohol intake

## Abstract

**Purpose:**

This study aimed to investigate the causal association between unhealthy lifestyle factors and diabetic retinopathy (DR) risk and to determine better interventions targeting these modifiable unhealthy factors.

**Design:**

Two-sample Mendelian randomization (MR) analysis was performed in this study. The inverse variance-weighted method was used as the primary method.

**Method:**

Our study included 687 single-nucleotide polymorphisms associated with unhealthy lifestyle factors as instrumental variables. Aggregated data on individual-level genetic information were obtained from the corresponding studies and consortia. A total of 292,622,3 cases and 739,241,18 variants from four large consortia (MRC Integrative Epidemiology Unit [MRC-IEU], Genetic Investigation of Anthropometric Traits [GIANT], GWAS & Sequencing Consortium of Alcohol and Nicotine Use [GSCAN], and Neale Lab) were included.

**Result:**

In the MR analysis, a higher body mass index (BMI) (odds ratio [OR], 95% confidence interval [CI] = 1.42, 1.30–1.54; *P* < 0.001] and cigarettes per day (OR, 95% CI = 1.16, 1.05–1.28; *P* = 0.003) were genetically predicted to be causally associated with an increased risk of DR, while patients with higher hip circumference (HC) had a lower risk of DR (OR, 95% CI = 0.85, 0.76–0.95; *P* = 0.004). In the analysis of subtypes of DR, the results of BMI and HC were similar to those of DR, whereas cigarettes per day were only related to proliferative DR (PDR) (OR, 95% CI = 1.18, 1.04–1.33; *P* = 0.009). In the MR-PRESSO analysis, a higher waist-to-hip ratio (WHR) was a risk factor for DR and PDR (OR, 95% CI = 1.24, 1.02–1.50, *P* = 0.041; OR, 95% CI = 1.32, 1.01–1.73, *P* = 0.049) after removing the outliers. Furthermore, no pleiotropy was observed in these exposures.

**Conclusion:**

Our findings suggest that higher BMI, WHR, and smoking are likely to be causal factors in the development of DR, whereas genetically higher HC is associated with a lower risk of DR, providing insights into a better understanding of the etiology and prevention of DR.

## 1 Introduction

Diabetic retinopathy (DR), a common microvascular complication of diabetes mellitus (DM), remains a leading cause of acquired vision loss in adults of working age and is associated with an increased risk of life-threatening systemic vascular complications (1). Moreover, 35% of individuals with diabetes experience DR, and 10% are in the stage of vision threatening (2). To confront the growing threat,revealing the risk factors of DR is an essential part of implementing interventions for prevention.

Considering unhealthy lifestyle a modifiable factor, mounting observational epidemiological studies have focused on the association between unhealthy lifestyle and DR. However, based on the current studies, the results of some unhealthy lifestyle factors are controversial and inconsistent (3–7). According to a meta-analysis of prospective cohort studies, obesity was a risk factor for non-proliferative DR (5). However, another meta-analysis failed to find any correlation between obesity and DR risk (7). Regarding alcohol intake, different meta-analyses also present controversial results. Chen et al. demonstrated no significant association between alcohol intake and DR (6) whereas Zhu et al. found protective effects in the wine group (3). Due to potential methodological limitations in observational studies, the causal association between such factors and DR can be confused by reverse causality or ethnicity, sex, and age.

Mendelian randomization (MR) analysis utilizes genetic predictors as instrumental variables (IVs) to investigate the causal association between risk factors and diseases (8). Because genetic variants are randomly distributed at conception, each IV is considered an alternative to randomized controlled trials and can avoid various bias commonly presented in traditional observational studies (9). However, most previous MR studies have focused on modified factors and diabetes, and we did not find any study concern about unhealthy lifestyle factors and DR. In addition, the influence of these factors on the different types of DR is uncertain. Thus, based on publicly available data, we performed MR analysis to assess the causal associations between unhealthy lifestyle factors (including smoking, alcohol intake, and obesity) and the risk of DR, including its subtypes.

## 2 Materials and methods

### 2.1 Genetic variants associated with unhealthy lifestyle

In this study, smoking, alcohol intake, and obesity were selected as typical examples of unhealthy lifestyle habits. We selected body mass index (BMI) (ukb-a-248), waist circumference (ieu-a-67), hip circumference (HC, ieu-a-55), and waist-to-hip ratio (WHR, ieu-a-79) as indicators of obesity. Single nucleotide variants (single-nucleotide polymorphisms [SNPs]) for BMI were retrieved from the Neale Lab consortium, and the rest were retrieved from a published meta-analysis of genome-wide association studies (GWAS) datasets summarized by Shungin (10) (datasets: ukb-a-248, ieu-a-79, ieu-a-55, ieu-a-67). The SNPs for smoking were obtained from another published meta-analysis of GWAS datasets summarized by Liu (11) and the MRC Integrative Epidemiology Unit (MRC-IEU) consortium (datasets: ieu-b-24, ieu-b-25, ukb-b-20261), including three phenotypes measured by the heaviness of smoking: age of smoking initiation, cigarettes per day, and ever smoked. For alcohol intake, we selected the SNPs of alcoholic drinks per week and alcohol intake frequency from a published meta-analysis of GWAS datasets summarized by Liu (11) and the GWAS & Sequencing Consortium of Alcohol and Nicotine use consortium (datasets: ieu-b-73, ukb-b-5779). All SNPs associated with exposures that reached the GWAS threshold of statistical significance (*P* < 5 × 10^−8^) were extracted as IVs. To ensure that they were entirely independent, linkage disequilibrium analysis was performed using a threshold of r^2^ < 0.001. Additionally, we extracted the summary data and effects of each SNP and calculated the effect sizes and standard errors using the MR-Base platform (12). Details of the data sources are provided in **Table 1**.

**Table 1:** Details of studies included in Mendelian randomization analyses.

Consortium	Phenotype	First author	Sample size	Year	Note
**MRC-IEU**	Diabetic retinopathy	NA	14,584	2021	https://gwas.mrcieu.ac.uk/datasets/finn-b-DM_RETINOPATHY_EXMORE/
	Background diabetic retinopathy		2,026		https://gwas.mrcieu.ac.uk/datasets/finn-b-DM_BCKGRND_RETINA/
	Proliferative diabetic retinopathy		8,681		https://gwas.mrcieu.ac.uk/datasets/finn-b-DM_RETINA_PROLIF/
**GSCAN**	Cigarettes per day	Liu M (11)	337,334	2019	https://www.nature.com/articles/s41588-018-0307-5
	Age Of Smoking Initiation		341,427		
	Alcoholic drinks per week		335,394		
**MRC-IEU**	Ever smoked	Ben Elsworth	461,066	2018	https://gwas.mrcieu.ac.uk/datasets/ukb-b-20261/
	Alcohol intake frequency		462,346		https://gwas.mrcieu.ac.uk/datasets/ukb-b-5779/
**Neale Lab**	Body mass index	Neale	336,107	2017	http://www.nealelab.is/blog/2017/9/11/details-and-considerations-of-the-uk-biobank-gwas
**GIANT**	Waist-to-hip ratio	Shungin D (10)	210,082	2015	https://www.nature.com/articles/nature14132
	Hip circumference		211,114		
	Waist circumference		231,353		

MRC-IEU, MRC Integrative Epidemiology Unit; GSCAN, GWAS & Sequencing Consortium of Alcohol and Nicotine use; GIANT, Genetic Investigation of ANthropometric Traits.

### 2.2 GWAS summary data on diabetic retinopathy

Published GWAS summary data on DR were retrieved from the MRC-IEU (14,584 cases and 176,010 controls, European ancestry) (https://gwas.mrcieu.ac.uk/) (13). To evaluate potential heterogeneity in the causal effects, we further stratified the DR type for background DR (BDR) and PDR. BDR is an early stage of DR known as non-PDR. We used GWAS summary data of 2026 BDR cases and 8681 PDR cases from the MRC-IEU with 204,208 common controls (**Table 1**). For each selected SNP, we retrieved summary data (the effects on DR, effect sizes, standard errors, and effect alleles) for the MRC-IEU using the MR-Base platform (12, 14).

### 2.3 Statistical analyses

**Figure 1:**
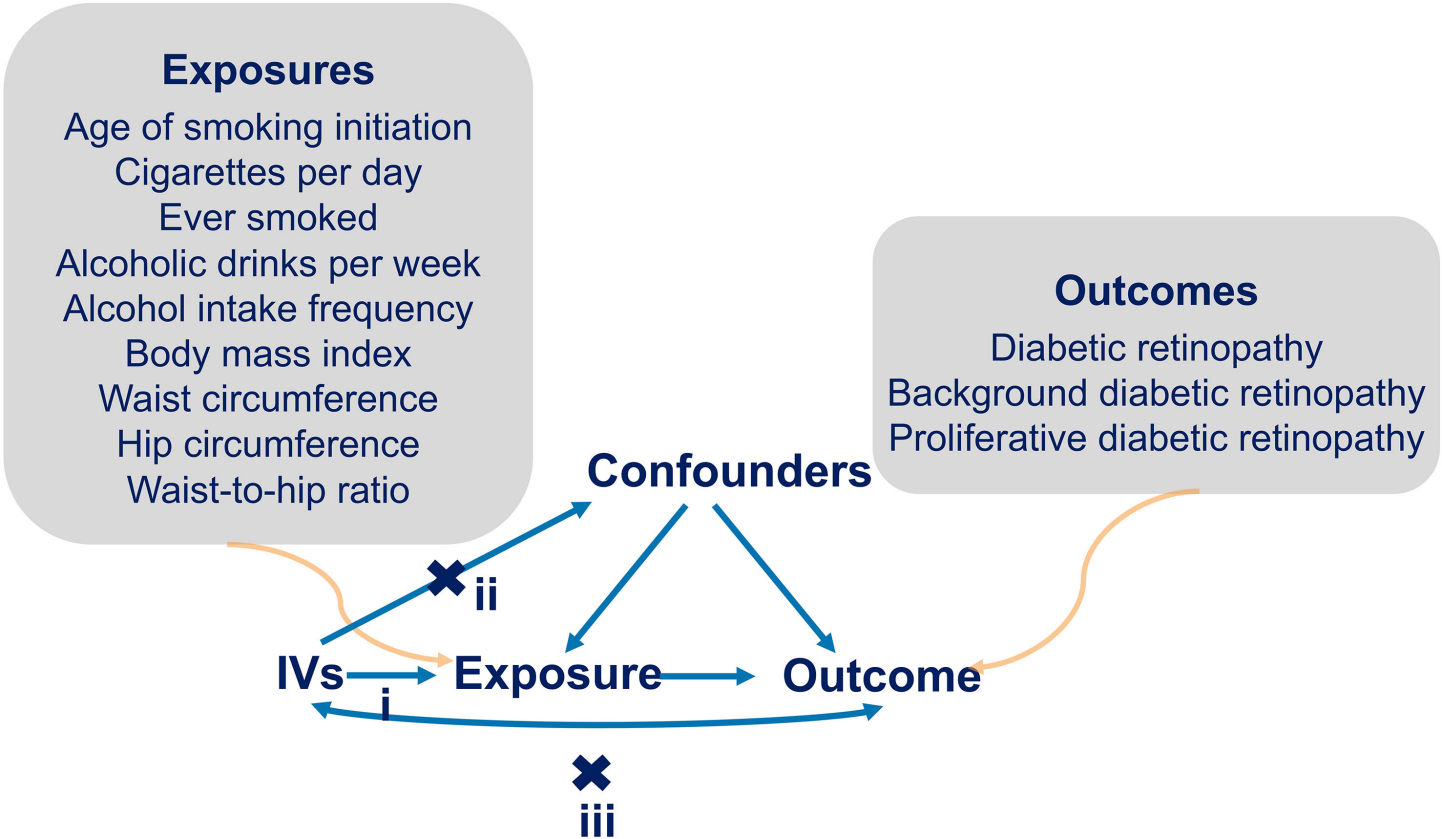
Diagram of MR assumptions. i): represents the IVs need to be related with the exposures robustly. ii): represents the IVs influence the outcome only through the exposures of interest. iii): represents the IVs are independent of any confounders of the exposure-outcome relation. Arrows interpret the causal relationships among variables.

**Figure 2:**
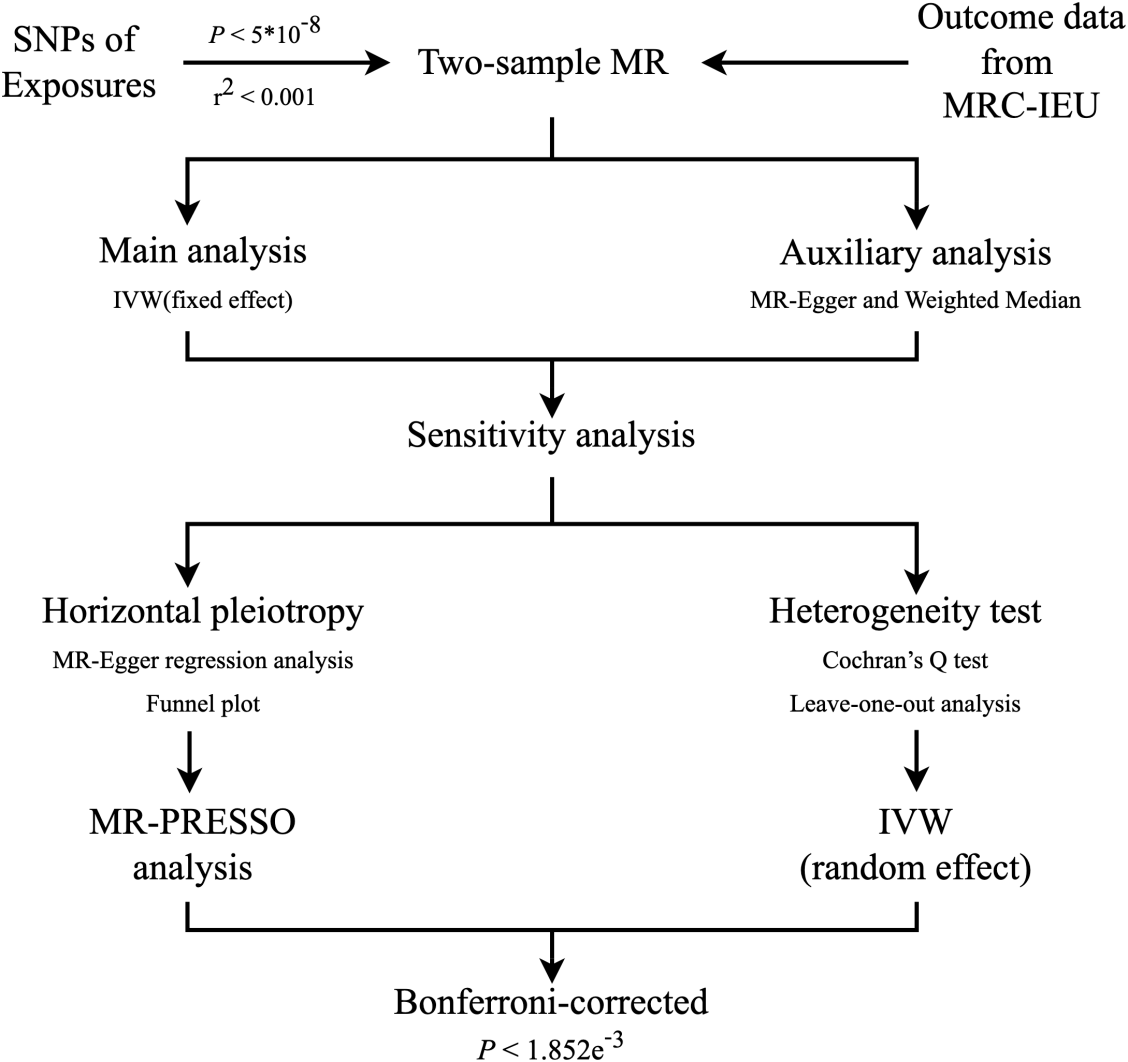
Flowchart of the statistical analyses, outlining the different analyses performed at each stage of the study. SNP, single-nucleotide polymorphism; MR, Mendelian randomization; MRC-IEU, MRC Integrative Epidemiology Unit; IVW, inverse‐variance weighted.

We used a two-sample MR approach to examine the potential causal association between unhealthy lifestyle and DR incidence. The MR method must satisfy three assumptions: (i) the IVs need to be robustly related to the exposures; (ii) the instruments influence the outcome only through the exposures of interest; and (iii) the IVs are independent of any confounders of the exposure–outcome association (**Figure 1**) (15). A flowchart of the statistical analyses is shown in **Figure 2**.

In the MR analyses, the inverse-variance weighted (IVW) model was the main method used in this study. The MR-Egger regression and weighted median (WM) models were also used to test the coherence of the causal estimates. The IVW method treated each SNP as a valid natural experiment, assessing the causal effects of each SNP on the outcome, and used the outcome as weights for meta-analysis to evaluate the combined causal effect. The IVW (fixed-effect) method provides an unbiased estimate in the absence of horizontal pleiotropy or when horizontal pleiotropy is balanced (16). If heterogeneity was present, the multiplicative random-effects IVW model was used and provided valid estimates under the assumption of balanced pleiotropy (17, 18).

In sensitivity analyses, the MR-Egger-intercept test was used to determine pleiotropy. We also applied the MR pleiotropy residual sum and outlier (MR-PRESSO) method to identify outlying SNPs and examine whether the causal effect would change after removing these outliers (19). Cochran’s Q statistic was used to determine the heterogeneity test between individual genetic variants (20). Leave-one-out analysis was performed to evaluate whether MR estimates were driven or biased by a single SNP and to calculate the meta-effect of the remaining SNPs. The asymmetry in funnel plots may indicate that the second assumption is violated by horizontal pleiotropy.

The results were shown as odds ratios (ORs) with their corresponding 95% confidence intervals (CIs). Because we included analyses of nine exposures with three outcomes, using a conservative approach, a Bonferroni-corrected *P* value < 0.05 divided by 27 (i.e., 1.852e^–3^) was considered a significant causal association to adjust for multiple testing. A *P*-value between 0.05 and 1.852e^–3^ was considered suggestive of a potential association. All statistical analyses were performed in R (version 4.1.0) using the R package “Two sample MR” (version 0.5.6) and “MR- PRESSO” (version 1.0) (12).

## 3 Results

### 3.1 Mendelian randomization analysis

#### 3.1.1 Overview of the results

**Figure 3:**
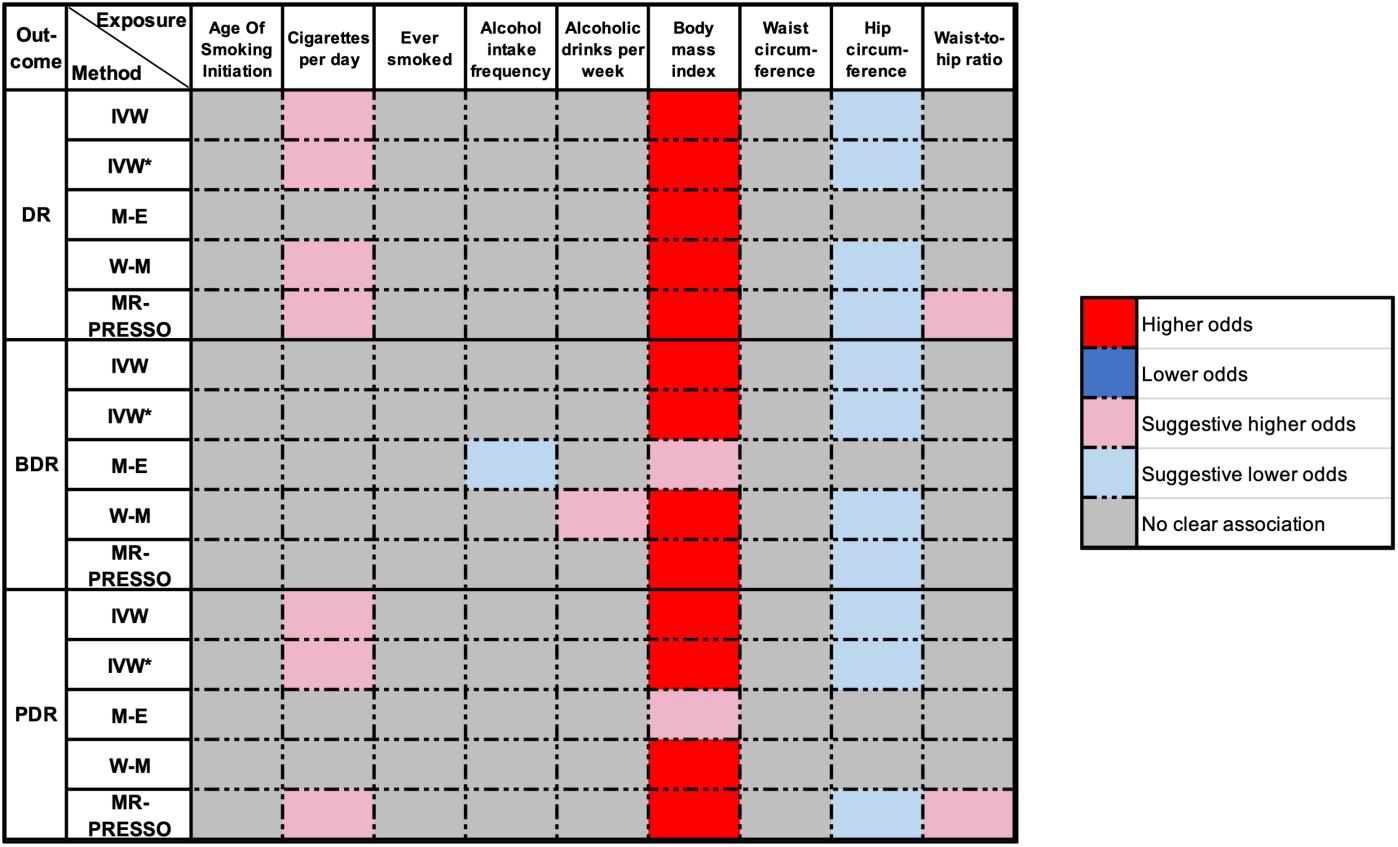
Overview of the main results of MR analyze. DR, diabetic retinopathy; BDR, background diabetic retinopathy; PDR, proliferative diabetic retinopathy; IVW, inverse‐variance weighted (fixed-effect); IVW*, Inverse variance weighted (multiplicative random-effects); MR, Mendelian randomization; M-E, MR Egger; W-M, weighted median; MR-PRESSO, MR Pleiotropy Residual Sum and Outlier. P-value < 1.852e^-03^ was regarded as a significant causal association, the P-value between 0.05 and 1.852e^-03^ was considered suggestive of a potential association.

**Figure 4:**
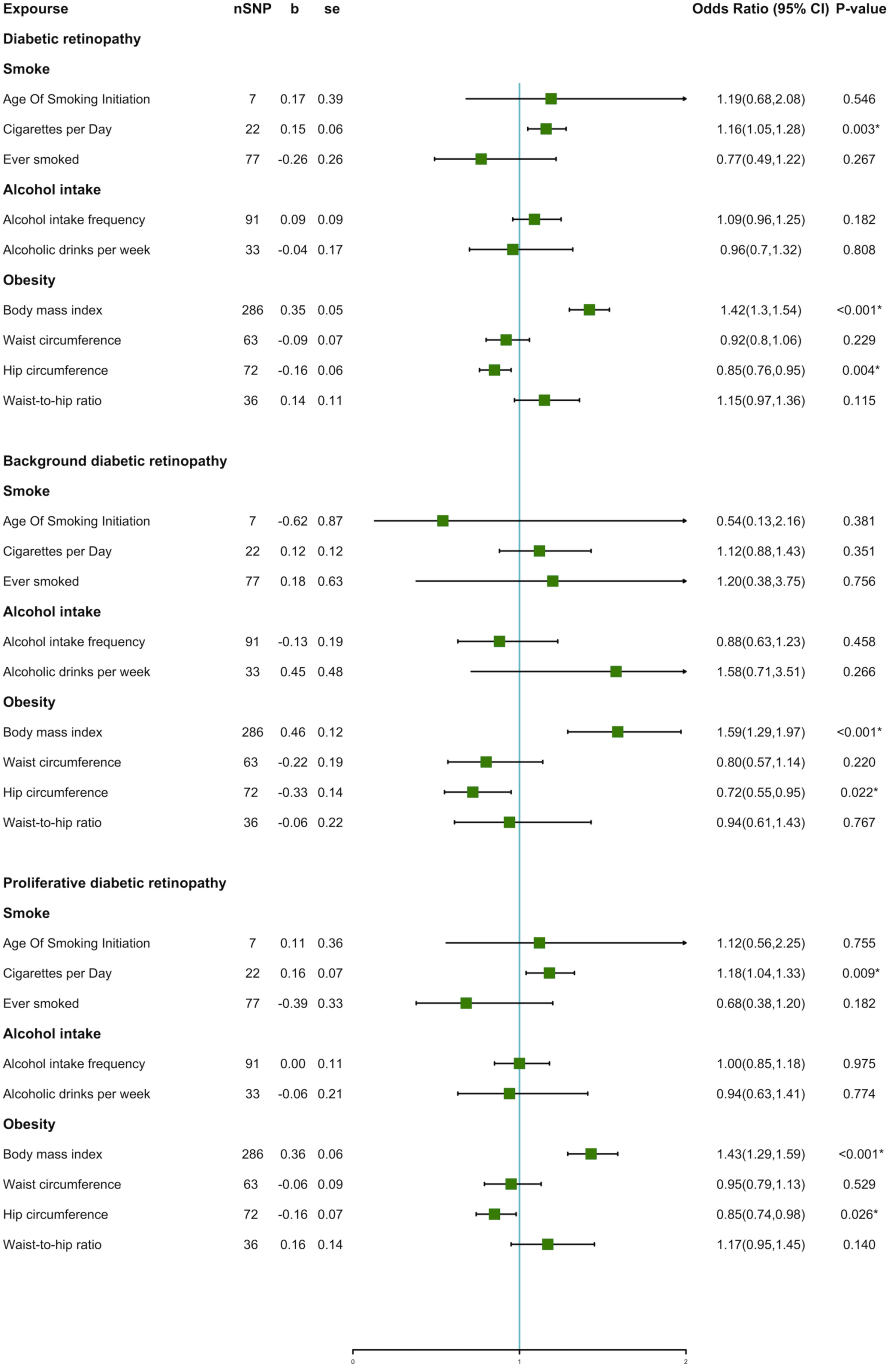
Forest plot of unhealthy lifestyle factors associated with the risk of DR and its subtypes.

An overview of the MR analysis results is shown in **Figure 3**. More specific results from the MR analyses are summarized in **Figure 4**, **Table 2**, and **Tables S2, S3**. Detailed information on each SNP is provided in **Table S1**.

**Table 2:** Mendelian randomization estimates of the associations between unhealthy lifestyle and risk of DR.

Exposure	IVW method (fixed-effect)		MR-Egger		Weighted median method	
	OR (95% CI)	*P*-value	OR (95% CI)	*P*-value	OR (95% CI)	*P*-value
Smoking						
Age Of Smoking Initiation	1.19(0.68,2.08)	0.546	0.43(0.04,5.00)	0.532	1.17(0.54,2.55)	0.691
Cigarettes per day	1.16(1.05,1.28)	0.003*	1.15(0.93,1.42)	0.221	1.20(1.04,1.38)	0.010*
Ever smoked	0.77(0.49,1.22)	0.267	0.24(0.02,2.73)	0.251	0.61(0.30,1.25)	0.177
Alcohol intake						
Alcohol intake frequency	1.09(0.96,1.25)	0.182	0.73(0.43,1.23)	0.242	1.05(0.85,1.29)	0.681
Alcoholic drinks per week	0.96(0.7,1.32)	0.808	0.93(0.43,2.00)	0.854	0.92(0.56,1.50)	0.729
Obesity						
Body mass index	1.42(1.3,1.54)	<0.001*	1.65 (1.25,2.18)	0.0005*	1.40 (1.22,1.61)	<0.001*
Waist circumference	0.92(0.8,1.06)	0.229	0.96(0.50,1.84)	0.904	0.93(0.76,1.15)	0.508
Hip circumference	0.85(0.76,0.95)	0.004*	0.94(0.62,1.43)	0.775	0.83(0.70,0.98)	0.033*
Waist-to-hip ratio	1.15(0.97,1.36)	0.115	0.99(0.36,2.70)	0.990	1.18(0.92,1.52)	0.192

IVW, inverse‐variance weighted; OR, odds ratio; CI, confidence interval.

#### 3.1.2 Results of DR

The IVW method indicated an over 40% increased risk of DR incidence per standard deviation (SD) increase in BMI (OR = 1.42; 95% CI, 1.30–1.54; *P* < 0.001*) and an approximately 20% increase in the risk of cigarettes per day (OR = 1.16; 95% CI, 1.05–1.28; *P* = 0.003*). Interestingly, higher HC decreased 15% lower risk in DR incidence per SD (OR = 0.85; 95% CI, 0.76–0.95; *P* = 0.004*). A forest plot of SNPs associated with cigarettes per day, BMI, and HC is presented in **Figure S1**. The results obtained using the WM method were similar to those obtained using the IVW method (**Table 2**). In the MR-Egger model, BMI also showed a significant causal effect. However, the MR-Egger model showed a consistent effect direction in cigarettes per day, and HC was not statistically significant (**Table 2**). We did not observe any association between alcohol intake and DR (**Figure 4**). We further proceeded with Bonferroni correction, which indicated significant causal effects between BMI and DR risk (*P* = 9.389e^–16^).

#### 3.1.3 Results of DR subtypes

In DR subtypes, a genetically predicted higher BMI increased the risk of BDR (OR = 1.59; 95% CI, 1.29–1.97; *P* < 0.001*) and PDR (OR = 1.43; 95% CI, 1.29–1.59; *P* < 0.001*). In the WM method and MR-Egger model, BMI also indicated an analogous causal effect. Higher HC was a protective factor in BDR (OR = 0.72; 95% CI, 0.55–0.95; *P* = 0.022*) and PDR (OR = 0.85; 95% CI, 0.74–0.98; *P* = 0.026*). The result of cigarettes per day indicated an increased risk of PDR (OR = 1.18; 95% CI, 1.04–1.33; *P* = 0.009*). The forest plot of SNPs associated with the above exposures and the risks of BDR and PDR are presented in **Figure S2**. The HC in the BDR using the WM method was also significant. The results of the MR-Egger model and other results of the WM method are presented in **Tables S2, S3**. We did not find causality in the other factors with BDR and PDR. After Bonferroni correction, a higher BMI still had a significant causal association with BDR and PDR risks (BDR, *P* = 1.687e^–5;^ PDR, *P* = 4.085e^–11^).

### 3.2 Sensitivity analyses

#### 3.2.1 Sensitivity analyses of DR

We assessed heterogeneity using Cochran’s Q test and horizontal pleiotropy from the MR-Egger regression analysis. There was no evidence of directional pleiotropy using MR-Egger regression (**Table 3**). Cochran’s Q test for BMI was statistically significant (*P* < 0.05). Hence, we used a multiplicative random-effects model to re-estimate the MR effect of BMI. The results showed a causal association and positive correlation between BMI and DR risk (*P* = 4.24e^–13^*).

**Table 3:** Sensitivity test of Mendelian randomization analyze of the associations between unhealthy lifestyle and risk of DR.

Exposure	MR-Egger regression analysis		Cochran’s Q test		IVW method (multiplicative random-effects)	
	*Intercept*	*P-value*	*Q statistic*	*P-value*	OR (95% CI)	*P-value*
Smoke						
Age Of Smoking Initiation	0.023	0.432	9.79	0.081	1.19(0.55,2.55)	0.659
Cigarettes per day	0.001	0.888	30.92	0.056	1.16(1.03,1.31)	0.013*
Ever smoked	0.008	0.335	92.89	0.079	0.77(0.46,1.28)	0.318
Alcohol intake						
Alcohol intake frequency	0.010	0.112	147.44	<0.001*	1.09(0.92,1.30)	0.304
Alcoholic drinks per week	0.001	0.927	34.24	0.315	0.96(0.69,1.34)	0.814
Obesity						
Body mass index	-0.003	0.251	348.61	0.005*	1.42 (1.29,1.56)	<0.001*
Waist circumference	-0.001	0.886	66.64	0.289	0.92(0.79,1.06)	0.246
Hip circumference	-0.003	0.622	72.67	0.390	0.85(0.76,0.95)	0.005*
Waist-to-hip ratio	0.004	0.776	52.13	0.024*	1.15(0.93,1.41)	0.197

IVW, inverse‐variance weighted; MR, Mendelian randomization.

#### 3.2.2 Sensitivity analyses of DR subtypes

In DR subtypes, the heterogeneity analysis showed a similar result for BMI (*P* < 0.05). The results of multiplicative random effects showed that BMI was a causal risk factor for BDR (*P* = 1.06e^–04^) and PDR (*P* = 6.05e^–09^). Directional pleiotropy was found in alcohol intake frequency in PDR (*P* = 0.024*) using MR-Egger regression. No evidence of directional pleiotropy was found in the other exposures (**Tables S4, S5**).

#### 3.2.3 MR-PRESSO analysis

We applied MR-PRESSO to recognize outlying SNPs that might cause horizontal pleiotropy effects. Several outliers were identified during the MR-PRESSO analysis. After removing these outliers, we found that a higher WHR indicated a higher risk of DR (OR = 1.24; 95% CI, 1.02–1.50; *P* = 0.041) and PDR (OR = 1.32; 95% CI, 1.01–1.73; *P* = 0.049). In the remaining cases, the results remained consistent with the original results after the removal of these outliers. The corrected results of the MR-PRESSO analysis are listed in **Table S6**.

#### 3.2.4 Leave-one-out analysis and funnel plot

The leave-one-out analysis suggested that the risk estimates of cigarettes per day, BMI, and HC for DR, BDR, and PDR generally remained consistent after eliminating each single SNP at a time (**Figures S3, S4**, **Table S7**). In addition, the points were roughly symmetrical on both sides of the vertical line in funnel plot, which meant no horizontal pleiotropy in the SNPs for shown exposures (**Figures S5, S6**).

## 4 Discussion

The present study demonstrated a causal association among cigarettes per day, BMI, and WHR and an increased risk of DR, whereas HC was found to have a lower risk of DR. In subgroup analyses, we found that higher BMI increased the risk of all types of DR, especially BDR. Cigarettes per day was only associated with PDR. Additionally, HC decreased the risks of BDR and PDR. These results were contrary to those of several previous conventional observational studies (3–7). A possible explanation is that the small sample sizes and confounders might interfere with the results of traditional observational epidemiological studies. Here, MR analysis demonstrated an unbiased estimation of whether certain risk factors played a causal role in diseases, using data from published large-scale GWAS data, which made our results more reliable and convincing.

The mechanisms underlying the associations among cigarette smoking, BMI, HC, WHR, and DR remain unclear. In smoking patients, nicotine causes a vasoconstrictive effect. This reduces the blood flow of the retina and makes it difficult for the retinal blood vessels to autoregulate hyperoxia (21, 22). Additionally, smoking may increase carboxyhemoglobin levels (23), thereby reducing the retinal oxygen delivery and oxygen-carrying capacity of the blood. Moreover, mainstream cigarette smoke contains certain components (24) that can interact with plasma and extracellular matrix proteins. This leads to the formation of covalent adducts similar to advanced glycation end products (25, 26) and involved in diabetic end-organ complications.

For obesity, a higher BMI is often associated with dyslipidemia – a disease that may be responsible for the development of DR (27). Moreover, obese individuals with hyperleptinemia (28) are more likely to have higher blood pressure and oxidative stress levels which consequently, are possibly correlated with an increased risk of DR. Additionally, higher levels of the vascular endothelial growth factor found in obese individuals (29) were related to the pathogenesis of PDR (30). For WHR, abdominal obesity played a role in insulin resistance (31) and inflammation (32). Both of these have an association with the pathogenesis of DR (33–35). For HC, the protective effects of a higher HC might result from a larger muscle mass in the gluteofemoral region (36). Different fat deposits exhibit different metabolic properties; lower body fat could somewhat reverse the impact of abdominal fat and protect against insulin resistance (37). As suggested by previous research, for a certain amount of abdominal fat, higher peripheral fat accumulation in the hips and thighs might relate to a better metabolic state (38–42). Additionally, a larger HC might reduce the risk of DR by the contribution of active lipoprotein lipase and the low turnover of fatty acid in gluteofemoral adipose tissues.

The present study has several strengths. First, very few studies have comprehensively investigated the association between unhealthy lifestyles and DR incidence. To the best of our knowledge, our study is the first to investigate the potential causal association between unhealthy lifestyles and the risk of DR employing the MR approach and a large GWASs data. Second, due to the use of MR analysis, our findings were less likely to cause confounding and reverse causality, when compared to those of conventional observational studies. Third, the accuracy of our findings might be higher as we applied analysis of subtypes stratified by clinical classification to investigate the consistency of the pooled effects. Finally, the sample overlap only could account for up to 6.94%, and the exposure and outcome data were obtained from different databases, thereby reducing the bias of the estimate in the direction of observational association. These strengths of MR analysis, as a result, might increase the reliability of our findings.

However, certain limitations were found in our study. The SNPs were obtained from five large European populations-based consortia, which, therefore, might affect the generalization of our findings in other populations and regions. Otherwise, our study only performed MR analysis with summarized statistics. This could only allow us to make a preliminary conclusion on the causal association between unhealthy lifestyles and DR but fail to further investigate this association in terms of age, sex, type of diabetes, and DR severity. Additionally, it is hardly possible to remove all pleiotropy in our studies, especially the correlated horizontal pleiotropy. Consequently, some undetected confounders between exposures and outcomes may bias our results. Therefore, our findings should be cautiously interpreted and confirmed through further studies.

In conclusion, we demonstrate that smoking, BMI, and WHR are risk factors for DR. For future studies, informed by the work reported here, individual-level data and basic science approaches are required to investigate the further mechanism underlying the association between cigarette intake and obesity and DR development. In addition, due to the contrary results of WHR and HC, more emphasis should be placed on how different fat distributions relate to DR, which can disclose the underlying association between obesity and DR and strengthen the understanding of obesity. For clinical practice and public health strategies, the present study highlights the importance of lifestyle management in patients with diabetes and provides a reference for the future refined identification of high-risk individuals for DR. Health education can be strengthened to suggest avoiding unhealthy lifestyles. In addition, more frequent ocular examinations may be essential in patients with diabetes with such risk factors.

## Data availability statement

The original contributions presented in the study are included in the article/**Supplementary Material**. Further inquiries can be directed to the corresponding authors.

## Author contributions

FJ and XW were responsible for the concept and design of the study, interpretation of data, and drafting and writing of the article. ZS and ZW were contributed equally to the manuscript as joint first authors. The other authors were responsible for interpretation of data and revision of the intellectual content. All authors contributed to the article and approved the submitted version.
