# Supplementary material for: Diabetic retinopathy risk in patients with unhealthy lifestyle: A Mendelian randomization study
Source: Front Endocrinol (Lausanne). 2023 Jan 17;13:1087965. doi: 10.3389/fendo.2022.1087965 (PMC9887126; doi:10.3389/fendo.2022.1087965)
Supplement: Supplementary file 1 [file Image_1.pdf]

Figure S1. Forest plot and funnel plot of single nucleotide polymorphisms (SNPs) associated with cigarettes per day, body mass index (BMI), hip circumference (HC) and their risk of diabetic retinopathy (DR).

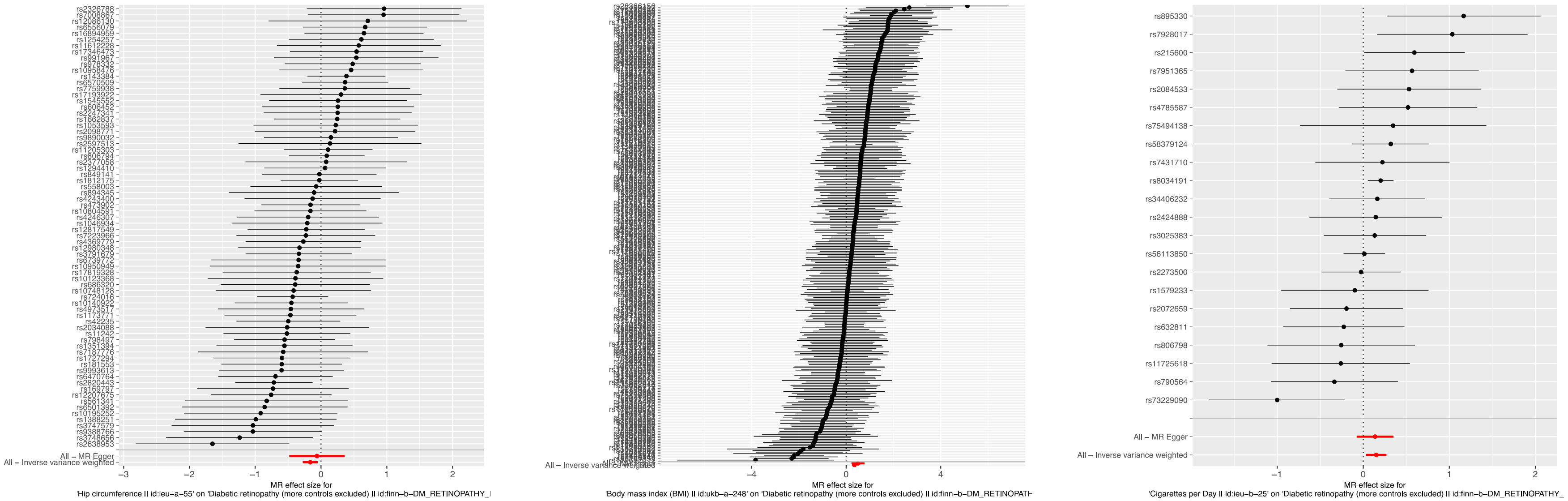

Figure S2. Forest plot and funnel plot of SNPs associated with BMI, HC and their risk of background diabetic retinopathy (BDR) and proliferative diabetic retinopathy (PDR).

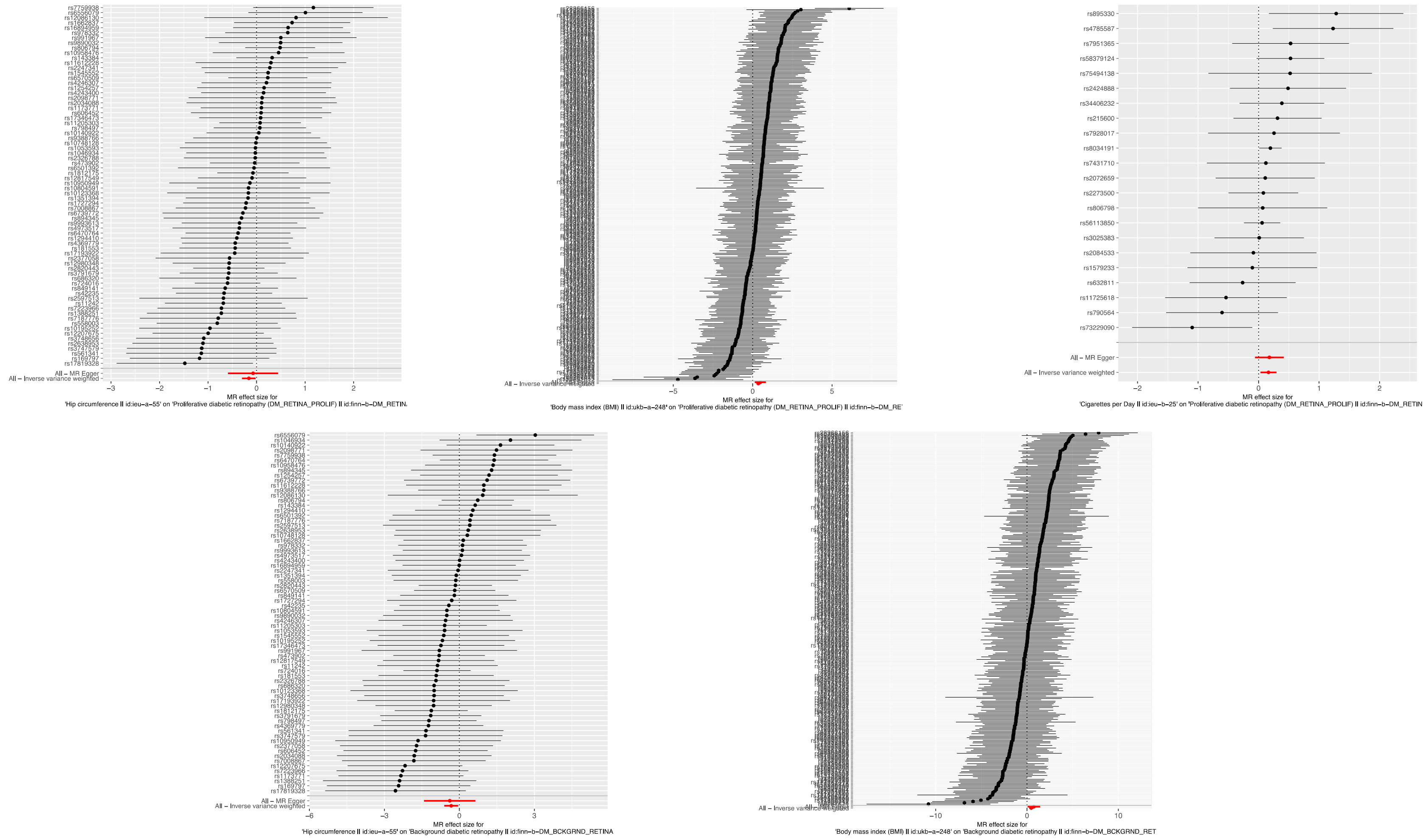

Figure S3. Leave one out sensitivity analysis for cigarettes per day, BMI, HC on DR.

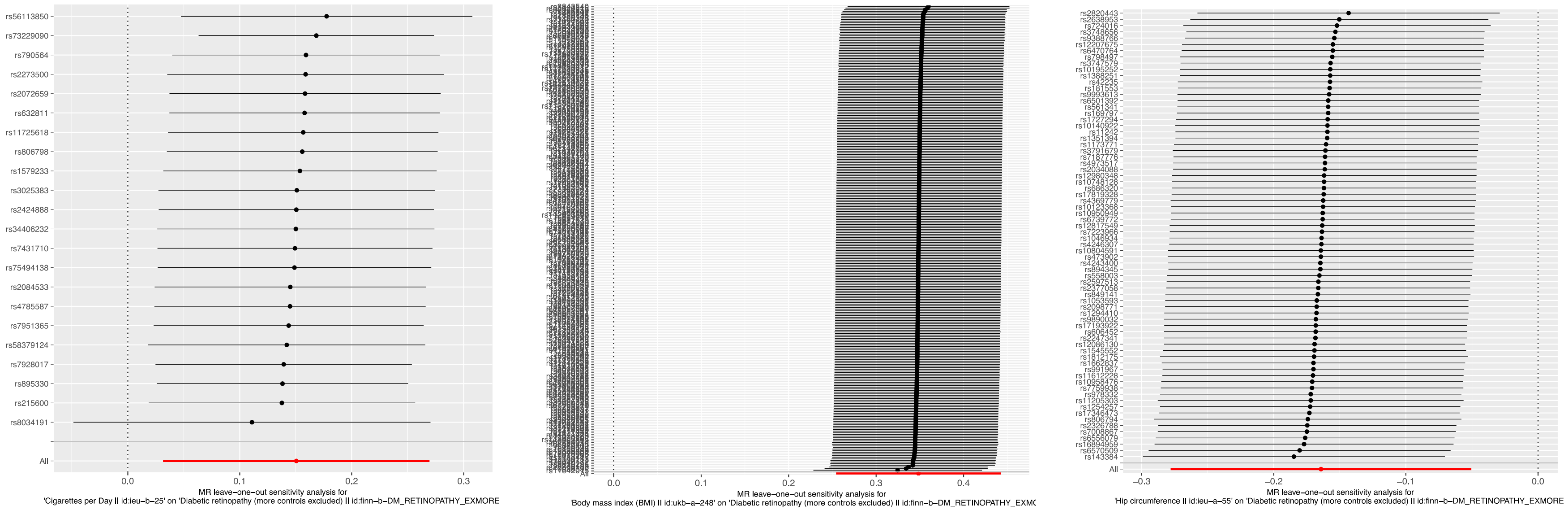

Figure S4. Leave one out sensitivity analysis for cigarettes per day, BMI, HC on BDR and PDR.

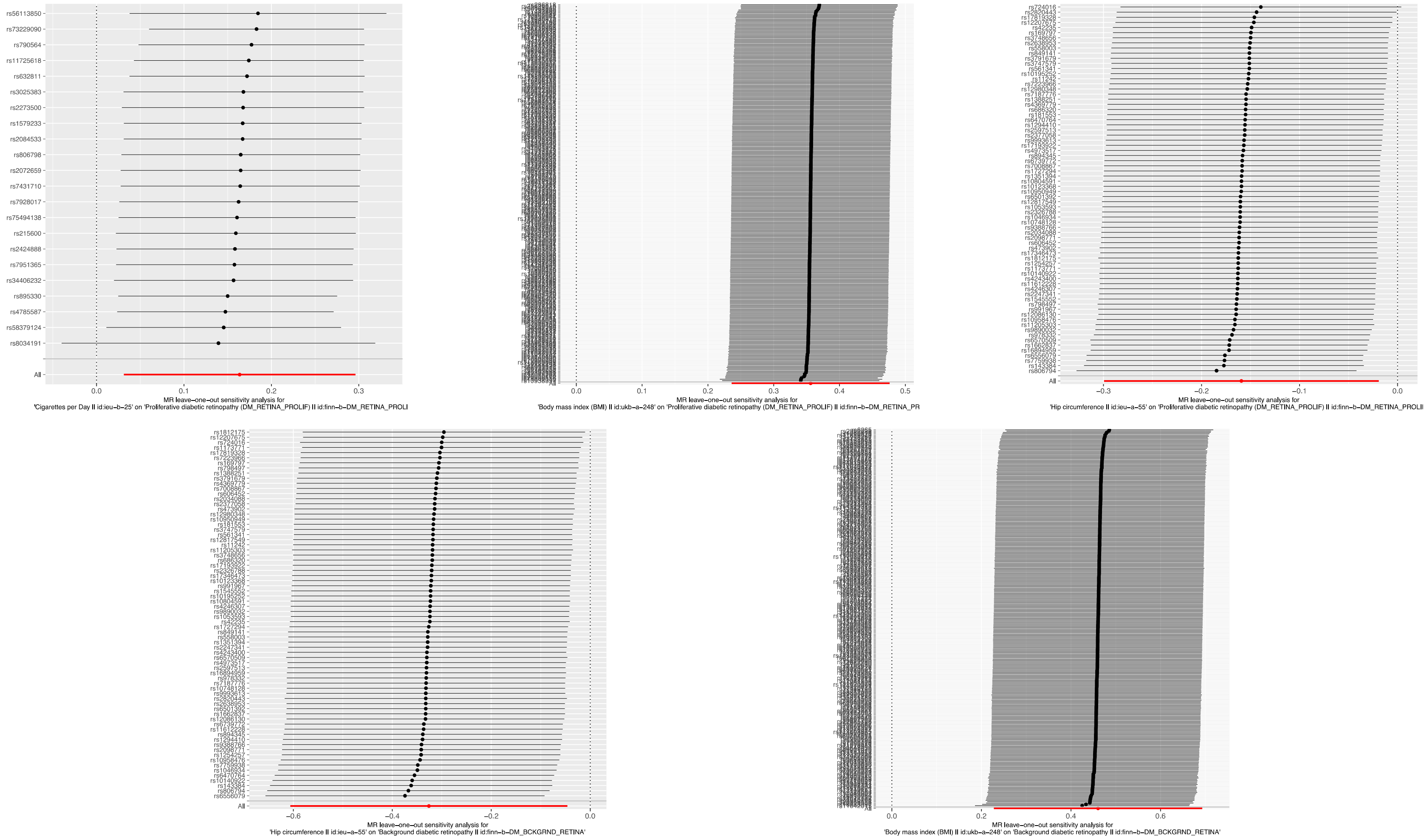

Figure S5. Funnel plot of SNPs associated with cigarettes per day, BMI, HC and their risk of DR.

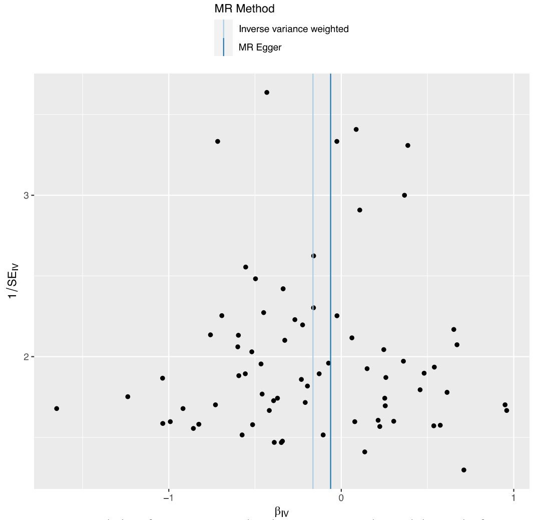

Funnel plot of SNPs associated with cigarettes per day and their risk of DR

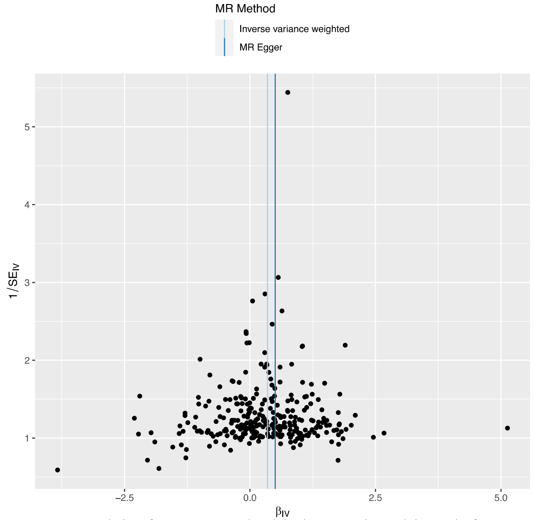

Funnel plot of SNPs associated with body mass index and their risk of DR

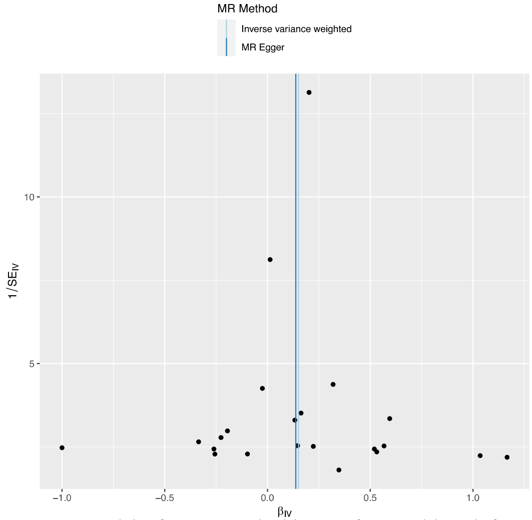

Funnel plot of SNPs associated with hip circumference and their risk of DR

Figure S6. Funnel plot of SNPs associated with cigarettes per day, BMI, HC and their risk of BDR and PDR.

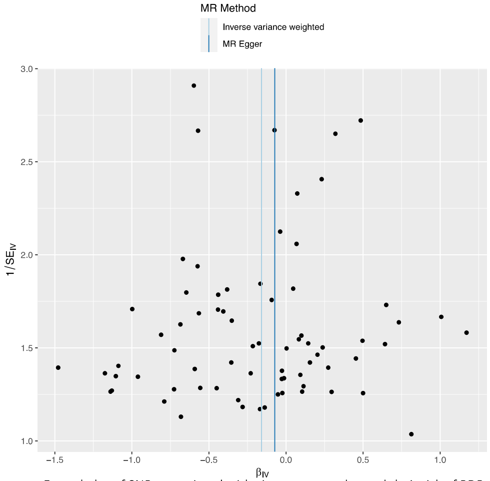

Funnel plot of SNPs associated with cigarettes per day and their risk of PDR

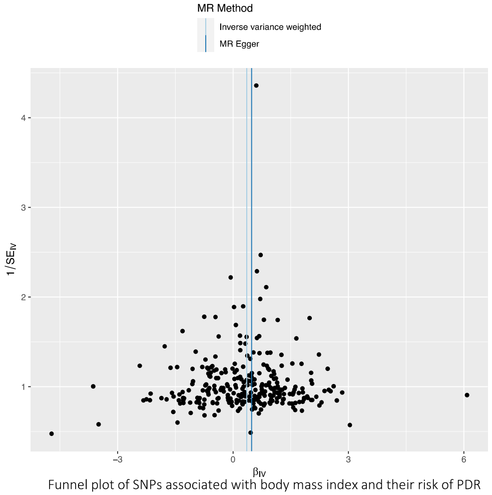

Funnel plot of SNPs associated with body mass index and their risk of PDR

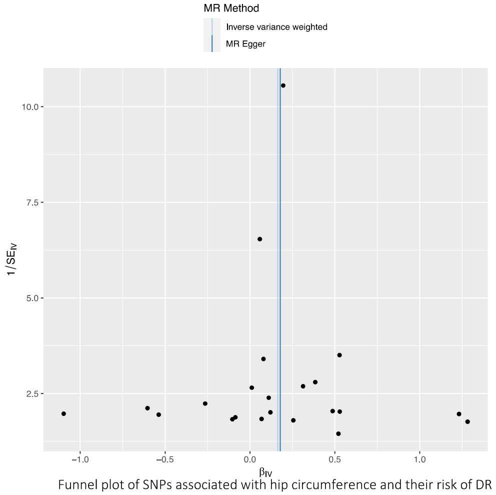

Funnel plot of SNPs associated with hip circumference and their risk of DR

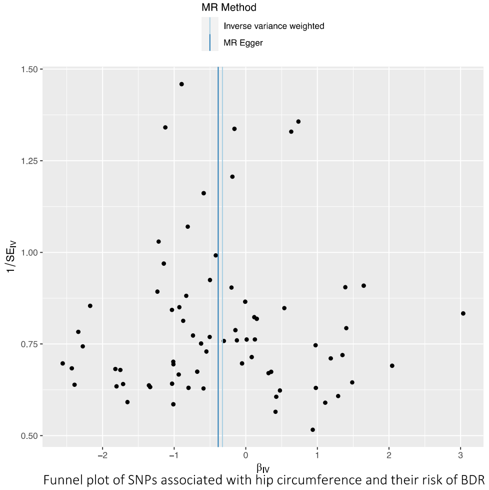

Funnel plot of SNPs associated with hip circumference and their risk of BDR

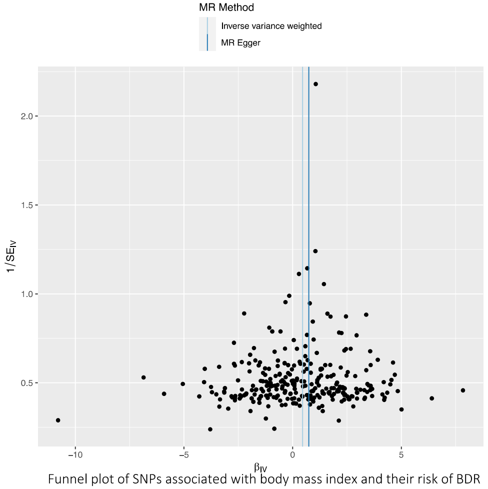

Funnel plot of SNPs associated with body mass index and their risk of BDR
