# Supplementary material for: Diabetic retinopathy risk in patients with unhealthy lifestyle: A Mendelian randomization study
Source: Front Endocrinol (Lausanne). 2023 Jan 17;13:1087965. doi: 10.3389/fendo.2022.1087965 (PMC9887126; doi:10.3389/fendo.2022.1087965)
Supplement: Supplementary file 2 [file Table_1.pdf]

Table S1. The detailed information of each single-nucleotide polymorphism

SNP	exposure	effect sizes	standard errors	effect allele	outcome	effect sizes	standard errors	effect allele
rs10200107	Age Of Smoking Initiation	-0.0196	0.0028	A	Background diabetic retinopathy	-0.0003	0.0339	A
rs11780471	Age Of Smoking Initiation	0.0379	0.0058	A	Background diabetic retinopathy	-0.1323	0.0666	A
rs11915747	Age Of Smoking Initiation	0.0210	0.0029	G	Background diabetic retinopathy	0.0096	0.0337	G
rs140485736	Age Of Smoking Initiation	0.0655	0.0115	A	Background diabetic retinopathy	-0.0133	0.1368	A
rs319748	Age Of Smoking Initiation	-0.0170	0.0031	A	Background diabetic retinopathy	-0.0597	0.0370	A
rs3768886	Age Of Smoking Initiation	0.0171	0.0030	C	Background diabetic retinopathy	-0.0605	0.0356	C
rs624833	Age Of Smoking Initiation	0.0173	0.0030	G	Background diabetic retinopathy	-0.0111	0.0344	G
rs10200107	Age Of Smoking Initiation	-0.0196	0.0028	A	Diabetic retinopathy	-0.0397	0.0136	A
rs11780471	Age Of Smoking Initiation	0.0379	0.0058	A	Diabetic retinopathy	-0.0315	0.0265	A
rs11915747	Age Of Smoking Initiation	0.0210	0.0029	G	Diabetic retinopathy	0.0068	0.0135	G
rs140485736	Age Of Smoking Initiation	0.0655	0.0115	A	Diabetic retinopathy	-0.0207	0.0552	A
rs319748	Age Of Smoking Initiation	-0.0170	0.0031	A	Diabetic retinopathy	-0.0018	0.0148	A
rs3768886	Age Of Smoking Initiation	0.0171	0.0030	C	Diabetic retinopathy	-0.0152	0.0143	C
rs624833	Age Of Smoking Initiation	0.0173	0.0030	G	Diabetic retinopathy	0.0045	0.0138	G
rs10200107	Age Of Smoking Initiation	-0.0196	0.0028	A	Proliferative diabetic retinopathy	-0.0227	0.0170	A
rs11780471	Age Of Smoking Initiation	0.0379	0.0058	A	Proliferative diabetic retinopathy	-0.0224	0.0332	A
rs11915747	Age Of Smoking Initiation	0.0210	0.0029	G	Proliferative diabetic retinopathy	0.0178	0.0168	G
rs140485736	Age Of Smoking Initiation	0.0655	0.0115	A	Proliferative diabetic retinopathy	-0.0441	0.0687	A
rs319748	Age Of Smoking Initiation	-0.0170	0.0031	A	Proliferative diabetic retinopathy	-0.0137	0.0185	A
rs3768886	Age Of Smoking Initiation	0.0171	0.0030	C	Proliferative diabetic retinopathy	-0.0243	0.0178	C
rs624833	Age Of Smoking Initiation	0.0173	0.0030	G	Proliferative diabetic retinopathy	0.0005	0.0172	G
rs10188314	Alcohol intake frequency.	-0.0198	0.0030	T	Background diabetic retinopathy	-0.0174	0.0328	T
rs10792669	Alcohol intake frequency.	0.0174	0.0030	G	Background diabetic retinopathy	0.0645	0.0329	G
rs11039429	Alcohol intake frequency.	-0.0236	0.0030	T	Background diabetic retinopathy	-0.0335	0.0343	T
rs1104608	Alcohol intake frequency.	0.0174	0.0031	C	Background diabetic retinopathy	0.0277	0.0334	C
rs11223617	Alcohol intake frequency.	0.0251	0.0038	A	Background diabetic retinopathy	0.0183	0.0360	A
rs11700855	Alcohol intake frequency.	-0.0298	0.0052	G	Background diabetic retinopathy	0.0171	0.0510	G
rs11750777	Alcohol intake frequency.	-0.0205	0.0037	A	Background diabetic retinopathy	0.0115	0.0458	A
rs11787216	Alcohol intake frequency.	0.0244	0.0032	T	Background diabetic retinopathy	0.0095	0.0341	T

rs11940694	Alcohol intake frequency.	-0.0437	0.0031	G	Background diabetic retinopathy	0.0421	0.0344	G
rs12153855	Alcohol intake frequency.	0.0294	0.0049	C	Background diabetic retinopathy	-0.2915	0.0719	C
rs1228589	Alcohol intake frequency.	0.0211	0.0035	A	Background diabetic retinopathy	-0.0016	0.0380	A
rs1229984	Alcohol intake frequency.	-0.2617	0.0092	C	Background diabetic retinopathy	0.2733	0.2271	C
rs12312693	Alcohol intake frequency.	-0.0177	0.0031	C	Background diabetic retinopathy	0.0167	0.0330	C
rs13102973	Alcohol intake frequency.	-0.0194	0.0031	C	Background diabetic retinopathy	0.0008	0.0330	C
rs13135092	Alcohol intake frequency.	0.0438	0.0055	G	Background diabetic retinopathy	-0.1330	0.1247	G
rs13178443	Alcohol intake frequency.	-0.0187	0.0034	T	Background diabetic retinopathy	-0.0626	0.0355	T
rs13390019	Alcohol intake frequency.	0.0296	0.0045	C	Background diabetic retinopathy	-0.0043	0.0676	C
rs1421085	Alcohol intake frequency.	0.0199	0.0031	C	Background diabetic retinopathy	0.0759	0.0332	C
rs1515591	Alcohol intake frequency.	0.0182	0.0031	G	Background diabetic retinopathy	0.0126	0.0329	G
rs1666658	Alcohol intake frequency.	0.0180	0.0031	C	Background diabetic retinopathy	0.0110	0.0337	C
rs17662759	Alcohol intake frequency.	0.0301	0.0055	C	Background diabetic retinopathy	0.0365	0.0530	C
rs17690703	Alcohol intake frequency.	0.0250	0.0034	T	Background diabetic retinopathy	0.0389	0.0510	T
rs186347	Alcohol intake frequency.	0.0179	0.0031	T	Background diabetic retinopathy	-0.0296	0.0331	T
rs1894544	Alcohol intake frequency.	0.0174	0.0030	C	Background diabetic retinopathy	0.0011	0.0330	C
rs1937522	Alcohol intake frequency.	0.0169	0.0030	G	Background diabetic retinopathy	-0.0108	0.0328	G
rs1991083	Alcohol intake frequency.	-0.0224	0.0033	T	Background diabetic retinopathy	-0.0082	0.0378	T
rs2043677	Alcohol intake frequency.	0.0261	0.0043	T	Background diabetic retinopathy	-0.0437	0.0458	T
rs2159935	Alcohol intake frequency.	-0.0186	0.0030	A	Background diabetic retinopathy	0.0396	0.0329	A
rs2160935	Alcohol intake frequency.	-0.0187	0.0031	T	Background diabetic retinopathy	-0.0541	0.0344	T
rs2244598	Alcohol intake frequency.	-0.0184	0.0031	C	Background diabetic retinopathy	-0.0377	0.0338	C
rs2411453	Alcohol intake frequency.	-0.0351	0.0031	G	Background diabetic retinopathy	0.0312	0.0328	G
rs2535911	Alcohol intake frequency.	-0.0188	0.0032	T	Background diabetic retinopathy	0.0303	0.0331	T
rs2622167	Alcohol intake frequency.	-0.0191	0.0031	A	Background diabetic retinopathy	-0.0127	0.0351	A
rs262240	Alcohol intake frequency.	-0.0172	0.0030	T	Background diabetic retinopathy	0.0499	0.0336	T
rs2717063	Alcohol intake frequency.	-0.0204	0.0031	A	Background diabetic retinopathy	-0.0396	0.0337	A
rs28768122	Alcohol intake frequency.	0.0207	0.0036	C	Background diabetic retinopathy	0.0149	0.0386	C
rs28787109	Alcohol intake frequency.	0.0178	0.0031	A	Background diabetic retinopathy	0.0342	0.0380	A
rs2924321	Alcohol intake frequency.	-0.0195	0.0031	A	Background diabetic retinopathy	0.0004	0.0341	A
rs2977454	Alcohol intake frequency.	-0.0259	0.0046	G	Background diabetic retinopathy	0.0659	0.0439	G

rs34440851	Alcohol intake frequency.	-0.0227	0.0042	T	Background diabetic retinopathy	-0.0192	0.0391	T
rs34473884	Alcohol intake frequency.	-0.0204	0.0035	A	Background diabetic retinopathy	-0.0409	0.0391	A
rs34631026	Alcohol intake frequency.	-0.0169	0.0030	T	Background diabetic retinopathy	-0.0490	0.0346	T
rs34811474	Alcohol intake frequency.	-0.0202	0.0036	A	Background diabetic retinopathy	0.0171	0.0388	A
rs35105141	Alcohol intake frequency.	0.0263	0.0031	T	Background diabetic retinopathy	0.0153	0.0338	T
rs362307	Alcohol intake frequency.	0.0433	0.0058	T	Background diabetic retinopathy	-0.0147	0.0653	T
rs4241258	Alcohol intake frequency.	0.0251	0.0044	T	Background diabetic retinopathy	-0.0031	0.0547	T
rs4242715	Alcohol intake frequency.	-0.0187	0.0032	A	Background diabetic retinopathy	-0.0207	0.0332	A
rs4417025	Alcohol intake frequency.	-0.0188	0.0032	A	Background diabetic retinopathy	-0.0418	0.0389	A
rs4503294	Alcohol intake frequency.	0.0181	0.0031	T	Background diabetic retinopathy	-0.0310	0.0333	T
rs461599	Alcohol intake frequency.	-0.0192	0.0030	C	Background diabetic retinopathy	-0.0401	0.0328	C
rs4726481	Alcohol intake frequency.	0.0218	0.0031	T	Background diabetic retinopathy	-0.0796	0.0346	T
rs473098	Alcohol intake frequency.	-0.0217	0.0030	T	Background diabetic retinopathy	-0.0451	0.0341	T
rs489062	Alcohol intake frequency.	0.0166	0.0031	A	Background diabetic retinopathy	-0.0310	0.0331	A
rs4916723	Alcohol intake frequency.	0.0239	0.0031	C	Background diabetic retinopathy	-0.0399	0.0328	C
rs4940926	Alcohol intake frequency.	-0.0191	0.0034	C	Background diabetic retinopathy	-0.0730	0.0410	C
rs4968391	Alcohol intake frequency.	-0.0193	0.0032	T	Background diabetic retinopathy	0.0379	0.0336	T
rs5022348	Alcohol intake frequency.	0.0203	0.0036	T	Background diabetic retinopathy	0.0224	0.0329	T
rs550942	Alcohol intake frequency.	0.0224	0.0040	T	Background diabetic retinopathy	-0.0643	0.0545	T
rs56194430	Alcohol intake frequency.	0.0225	0.0041	T	Background diabetic retinopathy	0.0185	0.0496	T
rs58905411	Alcohol intake frequency.	-0.0266	0.0031	A	Background diabetic retinopathy	-0.0057	0.0332	A
rs6030200	Alcohol intake frequency.	-0.0195	0.0033	A	Background diabetic retinopathy	0.0058	0.0333	A
rs61873510	Alcohol intake frequency.	0.0204	0.0033	T	Background diabetic retinopathy	-0.0012	0.0359	T
rs62097995	Alcohol intake frequency.	0.0200	0.0031	A	Background diabetic retinopathy	0.0277	0.0333	A
rs62305780	Alcohol intake frequency.	-0.0485	0.0051	G	Background diabetic retinopathy	0.0137	0.0474	G
rs62339673	Alcohol intake frequency.	0.0183	0.0032	A	Background diabetic retinopathy	0.0734	0.0345	A
rs62466318	Alcohol intake frequency.	-0.0255	0.0038	T	Background diabetic retinopathy	0.0470	0.0415	T
rs650558	Alcohol intake frequency.	0.0207	0.0035	T	Background diabetic retinopathy	-0.0228	0.0397	T
rs6727281	Alcohol intake frequency.	-0.0243	0.0039	T	Background diabetic retinopathy	-0.0286	0.0374	T
rs6943160	Alcohol intake frequency.	0.0206	0.0037	C	Background diabetic retinopathy	-0.0166	0.0395	C
rs71651683	Alcohol intake frequency.	-0.0705	0.0128	T	Background diabetic retinopathy	-0.1706	0.1960	T

rs72769229	Alcohol intake frequency.	-0.0231	0.0042	T	Background diabetic retinopathy	0.0333	0.0510	T
rs72787062	Alcohol intake frequency.	-0.0282	0.0041	A	Background diabetic retinopathy	0.0660	0.0462	A
rs728538	Alcohol intake frequency.	0.0229	0.0041	G	Background diabetic retinopathy	0.0641	0.0416	G
rs7298932	Alcohol intake frequency.	-0.0237	0.0043	G	Background diabetic retinopathy	-0.0556	0.0501	G
rs7302200	Alcohol intake frequency.	-0.0184	0.0032	A	Background diabetic retinopathy	0.0896	0.0358	A
rs73050128	Alcohol intake frequency.	-0.0260	0.0041	A	Background diabetic retinopathy	-0.0191	0.0382	A
rs7330939	Alcohol intake frequency.	-0.0213	0.0034	T	Background diabetic retinopathy	0.0054	0.0348	T
rs74679146	Alcohol intake frequency.	-0.0321	0.0058	C	Background diabetic retinopathy	-0.0116	0.0633	C
rs7514579	Alcohol intake frequency.	0.0197	0.0036	C	Background diabetic retinopathy	-0.0124	0.0406	C
rs76082653	Alcohol intake frequency.	0.0464	0.0067	T	Background diabetic retinopathy	-0.0054	0.0934	T
rs7610856	Alcohol intake frequency.	-0.0239	0.0031	A	Background diabetic retinopathy	-0.0597	0.0334	A
rs780094	Alcohol intake frequency.	-0.0510	0.0031	C	Background diabetic retinopathy	0.0371	0.0342	C
rs780569	Alcohol intake frequency.	0.0198	0.0034	A	Background diabetic retinopathy	-0.0383	0.0385	A
rs80292319	Alcohol intake frequency.	-0.0394	0.0065	C	Background diabetic retinopathy	0.0523	0.0602	C
rs8043563	Alcohol intake frequency.	0.0234	0.0035	C	Background diabetic retinopathy	-0.0424	0.0369	C
rs838145	Alcohol intake frequency.	0.0220	0.0031	A	Background diabetic retinopathy	-0.0826	0.0345	A
rs8614	Alcohol intake frequency.	0.0248	0.0039	A	Background diabetic retinopathy	-0.0845	0.0453	A
rs9349379	Alcohol intake frequency.	-0.0193	0.0031	G	Background diabetic retinopathy	0.0335	0.0328	G
rs9372625	Alcohol intake frequency.	-0.0256	0.0031	A	Background diabetic retinopathy	-0.0394	0.0351	A
rs9403297	Alcohol intake frequency.	0.0188	0.0031	A	Background diabetic retinopathy	0.0588	0.0357	A
rs9648478	Alcohol intake frequency.	0.0169	0.0030	A	Background diabetic retinopathy	0.0271	0.0327	A
rs9814516	Alcohol intake frequency.	-0.0251	0.0036	T	Background diabetic retinopathy	0.0391	0.0396	T
rs9829192	Alcohol intake frequency.	0.0169	0.0031	T	Background diabetic retinopathy	-0.0023	0.0329	T
rs9906502	Alcohol intake frequency.	0.0238	0.0040	A	Background diabetic retinopathy	0.0427	0.0415	A
rs10188314	Alcohol intake frequency.	-0.0198	0.0030	T	Diabetic retinopathy	-0.0147	0.0132	T
rs10792669	Alcohol intake frequency.	0.0174	0.0030	G	Diabetic retinopathy	0.0145	0.0132	G
rs11039429	Alcohol intake frequency.	-0.0236	0.0030	T	Diabetic retinopathy	0.0015	0.0138	T
rs1104608	Alcohol intake frequency.	0.0174	0.0031	C	Diabetic retinopathy	-0.0023	0.0134	C
rs11223617	Alcohol intake frequency.	0.0251	0.0038	A	Diabetic retinopathy	0.0069	0.0144	A
rs11700855	Alcohol intake frequency.	-0.0298	0.0052	G	Diabetic retinopathy	-0.0046	0.0203	G
rs11750777	Alcohol intake frequency.	-0.0205	0.0037	A	Diabetic retinopathy	-0.0322	0.0184	A

rs11787216	Alcohol intake frequency.	0.0244	0.0032	T	Diabetic retinopathy	0.0090	0.0137	T
rs11940694	Alcohol intake frequency.	-0.0437	0.0031	G	Diabetic retinopathy	0.0100	0.0138	G
rs12153855	Alcohol intake frequency.	0.0294	0.0049	C	Diabetic retinopathy	-0.1042	0.0283	C
rs1228589	Alcohol intake frequency.	0.0211	0.0035	A	Diabetic retinopathy	0.0156	0.0152	A
rs1229984	Alcohol intake frequency.	-0.2617	0.0092	C	Diabetic retinopathy	-0.1272	0.0897	C
rs12312693	Alcohol intake frequency.	-0.0177	0.0031	C	Diabetic retinopathy	0.0092	0.0132	C
rs13102973	Alcohol intake frequency.	-0.0194	0.0031	C	Diabetic retinopathy	0.0045	0.0132	C
rs13135092	Alcohol intake frequency.	0.0438	0.0055	G	Diabetic retinopathy	0.0473	0.0504	G
rs13178443	Alcohol intake frequency.	-0.0187	0.0034	T	Diabetic retinopathy	-0.0221	0.0142	T
rs13390019	Alcohol intake frequency.	0.0296	0.0045	C	Diabetic retinopathy	0.0194	0.0272	C
rs1421085	Alcohol intake frequency.	0.0199	0.0031	C	Diabetic retinopathy	0.0543	0.0133	C
rs1515591	Alcohol intake frequency.	0.0182	0.0031	G	Diabetic retinopathy	-0.0007	0.0132	G
rs1666658	Alcohol intake frequency.	0.0180	0.0031	C	Diabetic retinopathy	-0.0260	0.0135	C
rs17662759	Alcohol intake frequency.	0.0301	0.0055	C	Diabetic retinopathy	0.0190	0.0213	C
rs17690703	Alcohol intake frequency.	0.0250	0.0034	T	Diabetic retinopathy	0.0101	0.0204	T
rs186347	Alcohol intake frequency.	0.0179	0.0031	T	Diabetic retinopathy	-0.0232	0.0133	T
rs1894544	Alcohol intake frequency.	0.0174	0.0030	C	Diabetic retinopathy	0.0149	0.0132	C
rs1937522	Alcohol intake frequency.	0.0169	0.0030	G	Diabetic retinopathy	0.0040	0.0132	G
rs1991083	Alcohol intake frequency.	-0.0224	0.0033	T	Diabetic retinopathy	0.0173	0.0152	T
rs2043677	Alcohol intake frequency.	0.0261	0.0043	T	Diabetic retinopathy	0.0119	0.0184	T
rs2159935	Alcohol intake frequency.	-0.0186	0.0030	A	Diabetic retinopathy	-0.0019	0.0132	A
rs2160935	Alcohol intake frequency.	-0.0187	0.0031	T	Diabetic retinopathy	-0.0243	0.0138	T
rs2244598	Alcohol intake frequency.	-0.0184	0.0031	C	Diabetic retinopathy	-0.0182	0.0135	C
rs2411453	Alcohol intake frequency.	-0.0351	0.0031	G	Diabetic retinopathy	0.0255	0.0132	G
rs2535911	Alcohol intake frequency.	-0.0188	0.0032	T	Diabetic retinopathy	0.0249	0.0133	T
rs2622167	Alcohol intake frequency.	-0.0191	0.0031	A	Diabetic retinopathy	-0.0082	0.0141	A
rs262240	Alcohol intake frequency.	-0.0172	0.0030	T	Diabetic retinopathy	-0.0033	0.0135	T
rs2717063	Alcohol intake frequency.	-0.0204	0.0031	A	Diabetic retinopathy	-0.0080	0.0135	A
rs28768122	Alcohol intake frequency.	0.0207	0.0036	C	Diabetic retinopathy	0.0166	0.0155	C
rs28787109	Alcohol intake frequency.	0.0178	0.0031	A	Diabetic retinopathy	0.0008	0.0153	A
rs2924321	Alcohol intake frequency.	-0.0195	0.0031	A	Diabetic retinopathy	0.0076	0.0137	A

rs2977454	Alcohol intake frequency.	-0.0259	0.0046	G	Diabetic retinopathy	-0.0208	0.0176	G
rs34440851	Alcohol intake frequency.	-0.0227	0.0042	T	Diabetic retinopathy	0.0085	0.0156	T
rs34473884	Alcohol intake frequency.	-0.0204	0.0035	A	Diabetic retinopathy	-0.0187	0.0157	A
rs34631026	Alcohol intake frequency.	-0.0169	0.0030	T	Diabetic retinopathy	-0.0338	0.0139	T
rs34811474	Alcohol intake frequency.	-0.0202	0.0036	A	Diabetic retinopathy	-0.0254	0.0157	A
rs35105141	Alcohol intake frequency.	0.0263	0.0031	T	Diabetic retinopathy	-0.0073	0.0136	T
rs362307	Alcohol intake frequency.	0.0433	0.0058	T	Diabetic retinopathy	0.0215	0.0262	T
rs4241258	Alcohol intake frequency.	0.0251	0.0044	T	Diabetic retinopathy	-0.0099	0.0220	T
rs4242715	Alcohol intake frequency.	-0.0187	0.0032	A	Diabetic retinopathy	0.0217	0.0134	A
rs4417025	Alcohol intake frequency.	-0.0188	0.0032	A	Diabetic retinopathy	-0.0087	0.0156	A
rs4503294	Alcohol intake frequency.	0.0181	0.0031	T	Diabetic retinopathy	0.0004	0.0133	T
rs461599	Alcohol intake frequency.	-0.0192	0.0030	C	Diabetic retinopathy	0.0019	0.0132	C
rs4726481	Alcohol intake frequency.	0.0218	0.0031	T	Diabetic retinopathy	-0.0024	0.0139	T
rs473098	Alcohol intake frequency.	-0.0217	0.0030	T	Diabetic retinopathy	-0.0141	0.0136	T
rs489062	Alcohol intake frequency.	0.0166	0.0031	A	Diabetic retinopathy	0.0166	0.0133	A
rs4916723	Alcohol intake frequency.	0.0239	0.0031	C	Diabetic retinopathy	-0.0089	0.0132	C
rs4940926	Alcohol intake frequency.	-0.0191	0.0034	C	Diabetic retinopathy	-0.0351	0.0166	C
rs4968391	Alcohol intake frequency.	-0.0193	0.0032	T	Diabetic retinopathy	0.0171	0.0135	T
rs5022348	Alcohol intake frequency.	0.0203	0.0036	T	Diabetic retinopathy	0.0239	0.0132	T
rs550942	Alcohol intake frequency.	0.0224	0.0040	T	Diabetic retinopathy	-0.0366	0.0219	T
rs56194430	Alcohol intake frequency.	0.0225	0.0041	T	Diabetic retinopathy	0.0138	0.0200	T
rs58905411	Alcohol intake frequency.	-0.0266	0.0031	A	Diabetic retinopathy	-0.0276	0.0133	A
rs6030200	Alcohol intake frequency.	-0.0195	0.0033	A	Diabetic retinopathy	0.0099	0.0134	A
rs61873510	Alcohol intake frequency.	0.0204	0.0033	T	Diabetic retinopathy	0.0202	0.0145	T
rs62097995	Alcohol intake frequency.	0.0200	0.0031	A	Diabetic retinopathy	0.0274	0.0133	A
rs62305780	Alcohol intake frequency.	-0.0485	0.0051	G	Diabetic retinopathy	0.0188	0.0191	G
rs62339673	Alcohol intake frequency.	0.0183	0.0032	A	Diabetic retinopathy	0.0212	0.0138	A
rs62466318	Alcohol intake frequency.	-0.0255	0.0038	T	Diabetic retinopathy	0.0288	0.0167	T
rs650558	Alcohol intake frequency.	0.0207	0.0035	T	Diabetic retinopathy	0.0117	0.0160	T
rs6727281	Alcohol intake frequency.	-0.0243	0.0039	T	Diabetic retinopathy	-0.0076	0.0150	T
rs6943160	Alcohol intake frequency.	0.0206	0.0037	C	Diabetic retinopathy	0.0316	0.0159	C

rs71651683	Alcohol intake frequency.	-0.0705	0.0128	T	Diabetic retinopathy	0.0208	0.0780	T
rs72769229	Alcohol intake frequency.	-0.0231	0.0042	T	Diabetic retinopathy	0.0065	0.0203	T
rs72787062	Alcohol intake frequency.	-0.0282	0.0041	A	Diabetic retinopathy	0.0353	0.0185	A
rs728538	Alcohol intake frequency.	0.0229	0.0041	G	Diabetic retinopathy	0.0010	0.0168	G
rs7298932	Alcohol intake frequency.	-0.0237	0.0043	G	Diabetic retinopathy	-0.0081	0.0201	G
rs7302200	Alcohol intake frequency.	-0.0184	0.0032	A	Diabetic retinopathy	0.0549	0.0144	A
rs73050128	Alcohol intake frequency.	-0.0260	0.0041	A	Diabetic retinopathy	-0.0110	0.0153	A
rs7330939	Alcohol intake frequency.	-0.0213	0.0034	T	Diabetic retinopathy	0.0015	0.0139	T
rs74679146	Alcohol intake frequency.	-0.0321	0.0058	C	Diabetic retinopathy	-0.0162	0.0254	C
rs7514579	Alcohol intake frequency.	0.0197	0.0036	C	Diabetic retinopathy	0.0093	0.0162	C
rs76082653	Alcohol intake frequency.	0.0464	0.0067	T	Diabetic retinopathy	-0.0012	0.0377	T
rs7610856	Alcohol intake frequency.	-0.0239	0.0031	A	Diabetic retinopathy	-0.0260	0.0135	A
rs780094	Alcohol intake frequency.	-0.0510	0.0031	C	Diabetic retinopathy	0.0239	0.0137	C
rs780569	Alcohol intake frequency.	0.0198	0.0034	A	Diabetic retinopathy	0.0197	0.0155	A
rs80292319	Alcohol intake frequency.	-0.0394	0.0065	C	Diabetic retinopathy	0.0109	0.0241	C
rs8043563	Alcohol intake frequency.	0.0234	0.0035	C	Diabetic retinopathy	-0.0089	0.0148	C
rs838145	Alcohol intake frequency.	0.0220	0.0031	A	Diabetic retinopathy	-0.0203	0.0138	A
rs8614	Alcohol intake frequency.	0.0248	0.0039	A	Diabetic retinopathy	-0.0162	0.0182	A
rs9349379	Alcohol intake frequency.	-0.0193	0.0031	G	Diabetic retinopathy	-0.0062	0.0132	G
rs9372625	Alcohol intake frequency.	-0.0256	0.0031	A	Diabetic retinopathy	-0.0094	0.0141	A
rs9403297	Alcohol intake frequency.	0.0188	0.0031	A	Diabetic retinopathy	0.0170	0.0143	A
rs9648478	Alcohol intake frequency.	0.0169	0.0030	A	Diabetic retinopathy	0.0168	0.0131	A
rs9814516	Alcohol intake frequency.	-0.0251	0.0036	T	Diabetic retinopathy	-0.0050	0.0159	T
rs9829192	Alcohol intake frequency.	0.0169	0.0031	T	Diabetic retinopathy	-0.0054	0.0132	T
rs9906502	Alcohol intake frequency.	0.0238	0.0040	A	Diabetic retinopathy	-0.0032	0.0167	A
rs10188314	Alcohol intake frequency.	-0.0198	0.0030	T	Proliferative diabetic retinopathy	-0.0126	0.0164	T
rs10792669	Alcohol intake frequency.	0.0174	0.0030	G	Proliferative diabetic retinopathy	0.0209	0.0164	G
rs11039429	Alcohol intake frequency.	-0.0236	0.0030	T	Proliferative diabetic retinopathy	0.0074	0.0171	T
rs1104608	Alcohol intake frequency.	0.0174	0.0031	C	Proliferative diabetic retinopathy	-0.0007	0.0167	C
rs11223617	Alcohol intake frequency.	0.0251	0.0038	A	Proliferative diabetic retinopathy	0.0282	0.0180	A
rs11700855	Alcohol intake frequency.	-0.0298	0.0052	G	Proliferative diabetic retinopathy	0.0021	0.0255	G

rs11750777	Alcohol intake frequency.	-0.0205	0.0037	A	Proliferative diabetic retinopathy	-0.0094	0.0229	A
rs11787216	Alcohol intake frequency.	0.0244	0.0032	T	Proliferative diabetic retinopathy	0.0108	0.0171	T
rs11940694	Alcohol intake frequency.	-0.0437	0.0031	G	Proliferative diabetic retinopathy	0.0151	0.0172	G
rs12153855	Alcohol intake frequency.	0.0294	0.0049	C	Proliferative diabetic retinopathy	-0.1729	0.0355	C
rs1228589	Alcohol intake frequency.	0.0211	0.0035	A	Proliferative diabetic retinopathy	0.0063	0.0190	A
rs1229984	Alcohol intake frequency.	-0.2617	0.0092	C	Proliferative diabetic retinopathy	-0.1707	0.1131	C
rs12312693	Alcohol intake frequency.	-0.0177	0.0031	C	Proliferative diabetic retinopathy	0.0030	0.0165	C
rs13102973	Alcohol intake frequency.	-0.0194	0.0031	C	Proliferative diabetic retinopathy	0.0175	0.0165	C
rs13135092	Alcohol intake frequency.	0.0438	0.0055	G	Proliferative diabetic retinopathy	0.0125	0.0623	G
rs13178443	Alcohol intake frequency.	-0.0187	0.0034	T	Proliferative diabetic retinopathy	-0.0256	0.0178	T
rs13390019	Alcohol intake frequency.	0.0296	0.0045	C	Proliferative diabetic retinopathy	0.0252	0.0338	C
rs1421085	Alcohol intake frequency.	0.0199	0.0031	C	Proliferative diabetic retinopathy	0.0427	0.0166	C
rs1515591	Alcohol intake frequency.	0.0182	0.0031	G	Proliferative diabetic retinopathy	0.0127	0.0165	G
rs1666658	Alcohol intake frequency.	0.0180	0.0031	C	Proliferative diabetic retinopathy	0.0025	0.0168	C
rs17662759	Alcohol intake frequency.	0.0301	0.0055	C	Proliferative diabetic retinopathy	0.0493	0.0265	C
rs17690703	Alcohol intake frequency.	0.0250	0.0034	T	Proliferative diabetic retinopathy	0.0088	0.0254	T
rs186347	Alcohol intake frequency.	0.0179	0.0031	T	Proliferative diabetic retinopathy	-0.0315	0.0166	T
rs1894544	Alcohol intake frequency.	0.0174	0.0030	C	Proliferative diabetic retinopathy	0.0288	0.0165	C
rs1937522	Alcohol intake frequency.	0.0169	0.0030	G	Proliferative diabetic retinopathy	-0.0061	0.0164	G
rs1991083	Alcohol intake frequency.	-0.0224	0.0033	T	Proliferative diabetic retinopathy	0.0236	0.0189	T
rs2043677	Alcohol intake frequency.	0.0261	0.0043	T	Proliferative diabetic retinopathy	0.0010	0.0229	T
rs2159935	Alcohol intake frequency.	-0.0186	0.0030	A	Proliferative diabetic retinopathy	-0.0099	0.0165	A
rs2160935	Alcohol intake frequency.	-0.0187	0.0031	T	Proliferative diabetic retinopathy	-0.0264	0.0172	T
rs2244598	Alcohol intake frequency.	-0.0184	0.0031	C	Proliferative diabetic retinopathy	-0.0068	0.0169	C
rs2411453	Alcohol intake frequency.	-0.0351	0.0031	G	Proliferative diabetic retinopathy	0.0329	0.0164	G
rs2535911	Alcohol intake frequency.	-0.0188	0.0032	T	Proliferative diabetic retinopathy	0.0248	0.0166	T
rs2622167	Alcohol intake frequency.	-0.0191	0.0031	A	Proliferative diabetic retinopathy	-0.0037	0.0176	A
rs262240	Alcohol intake frequency.	-0.0172	0.0030	T	Proliferative diabetic retinopathy	0.0109	0.0168	T
rs2717063	Alcohol intake frequency.	-0.0204	0.0031	A	Proliferative diabetic retinopathy	-0.0120	0.0168	A
rs28768122	Alcohol intake frequency.	0.0207	0.0036	C	Proliferative diabetic retinopathy	0.0202	0.0193	C
rs28787109	Alcohol intake frequency.	0.0178	0.0031	A	Proliferative diabetic retinopathy	0.0167	0.0190	A

rs2924321	Alcohol intake frequency.	-0.0195	0.0031	A	Proliferative diabetic retinopathy	0.0140	0.0171	A
rs2977454	Alcohol intake frequency.	-0.0259	0.0046	G	Proliferative diabetic retinopathy	0.0088	0.0220	G
rs34440851	Alcohol intake frequency.	-0.0227	0.0042	T	Proliferative diabetic retinopathy	0.0254	0.0195	T
rs34473884	Alcohol intake frequency.	-0.0204	0.0035	A	Proliferative diabetic retinopathy	-0.0179	0.0195	A
rs34631026	Alcohol intake frequency.	-0.0169	0.0030	T	Proliferative diabetic retinopathy	-0.0350	0.0173	T
rs34811474	Alcohol intake frequency.	-0.0202	0.0036	A	Proliferative diabetic retinopathy	-0.0055	0.0195	A
rs35105141	Alcohol intake frequency.	0.0263	0.0031	T	Proliferative diabetic retinopathy	-0.0265	0.0169	T
rs362307	Alcohol intake frequency.	0.0433	0.0058	T	Proliferative diabetic retinopathy	0.0332	0.0325	T
rs4241258	Alcohol intake frequency.	0.0251	0.0044	T	Proliferative diabetic retinopathy	-0.0233	0.0274	T
rs4242715	Alcohol intake frequency.	-0.0187	0.0032	A	Proliferative diabetic retinopathy	0.0117	0.0166	A
rs4417025	Alcohol intake frequency.	-0.0188	0.0032	A	Proliferative diabetic retinopathy	-0.0180	0.0194	A
rs4503294	Alcohol intake frequency.	0.0181	0.0031	T	Proliferative diabetic retinopathy	0.0093	0.0167	T
rs461599	Alcohol intake frequency.	-0.0192	0.0030	C	Proliferative diabetic retinopathy	0.0074	0.0164	C
rs4726481	Alcohol intake frequency.	0.0218	0.0031	T	Proliferative diabetic retinopathy	-0.0043	0.0173	T
rs473098	Alcohol intake frequency.	-0.0217	0.0030	T	Proliferative diabetic retinopathy	-0.0113	0.0170	T
rs489062	Alcohol intake frequency.	0.0166	0.0031	A	Proliferative diabetic retinopathy	0.0148	0.0166	A
rs4916723	Alcohol intake frequency.	0.0239	0.0031	C	Proliferative diabetic retinopathy	-0.0202	0.0164	C
rs4940926	Alcohol intake frequency.	-0.0191	0.0034	C	Proliferative diabetic retinopathy	-0.0460	0.0206	C
rs4968391	Alcohol intake frequency.	-0.0193	0.0032	T	Proliferative diabetic retinopathy	0.0223	0.0168	T
rs5022348	Alcohol intake frequency.	0.0203	0.0036	T	Proliferative diabetic retinopathy	0.0305	0.0165	T
rs550942	Alcohol intake frequency.	0.0224	0.0040	T	Proliferative diabetic retinopathy	-0.0369	0.0273	T
rs56194430	Alcohol intake frequency.	0.0225	0.0041	T	Proliferative diabetic retinopathy	-0.0007	0.0249	T
rs58905411	Alcohol intake frequency.	-0.0266	0.0031	A	Proliferative diabetic retinopathy	-0.0260	0.0166	A
rs6030200	Alcohol intake frequency.	-0.0195	0.0033	A	Proliferative diabetic retinopathy	0.0082	0.0167	A
rs61873510	Alcohol intake frequency.	0.0204	0.0033	T	Proliferative diabetic retinopathy	0.0213	0.0180	T
rs62097995	Alcohol intake frequency.	0.0200	0.0031	A	Proliferative diabetic retinopathy	0.0310	0.0166	A
rs62305780	Alcohol intake frequency.	-0.0485	0.0051	G	Proliferative diabetic retinopathy	0.0119	0.0238	G
rs62339673	Alcohol intake frequency.	0.0183	0.0032	A	Proliferative diabetic retinopathy	0.0222	0.0173	A
rs62466318	Alcohol intake frequency.	-0.0255	0.0038	T	Proliferative diabetic retinopathy	0.0387	0.0208	T
rs650558	Alcohol intake frequency.	0.0207	0.0035	T	Proliferative diabetic retinopathy	0.0191	0.0199	T
rs6727281	Alcohol intake frequency.	-0.0243	0.0039	T	Proliferative diabetic retinopathy	-0.0183	0.0187	T

rs6943160	Alcohol intake frequency.	0.0206	0.0037	C	Proliferative diabetic retinopathy	0.0278	0.0198	C
rs71651683	Alcohol intake frequency.	-0.0705	0.0128	T	Proliferative diabetic retinopathy	-0.0094	0.0983	T
rs72769229	Alcohol intake frequency.	-0.0231	0.0042	T	Proliferative diabetic retinopathy	-0.0077	0.0255	T
rs72787062	Alcohol intake frequency.	-0.0282	0.0041	A	Proliferative diabetic retinopathy	0.0485	0.0231	A
rs728538	Alcohol intake frequency.	0.0229	0.0041	G	Proliferative diabetic retinopathy	-0.0272	0.0209	G
rs7298932	Alcohol intake frequency.	-0.0237	0.0043	G	Proliferative diabetic retinopathy	-0.0142	0.0251	G
rs7302200	Alcohol intake frequency.	-0.0184	0.0032	A	Proliferative diabetic retinopathy	0.0493	0.0179	A
rs73050128	Alcohol intake frequency.	-0.0260	0.0041	A	Proliferative diabetic retinopathy	0.0063	0.0191	A
rs7330939	Alcohol intake frequency.	-0.0213	0.0034	T	Proliferative diabetic retinopathy	0.0184	0.0174	T
rs74679146	Alcohol intake frequency.	-0.0321	0.0058	C	Proliferative diabetic retinopathy	-0.0374	0.0317	C
rs7514579	Alcohol intake frequency.	0.0197	0.0036	C	Proliferative diabetic retinopathy	0.0322	0.0202	C
rs76082653	Alcohol intake frequency.	0.0464	0.0067	T	Proliferative diabetic retinopathy	-0.0125	0.0468	T
rs7610856	Alcohol intake frequency.	-0.0239	0.0031	A	Proliferative diabetic retinopathy	-0.0348	0.0168	A
rs780094	Alcohol intake frequency.	-0.0510	0.0031	C	Proliferative diabetic retinopathy	0.0243	0.0171	C
rs780569	Alcohol intake frequency.	0.0198	0.0034	A	Proliferative diabetic retinopathy	-0.0037	0.0193	A
rs80292319	Alcohol intake frequency.	-0.0394	0.0065	C	Proliferative diabetic retinopathy	0.0428	0.0302	C
rs8043563	Alcohol intake frequency.	0.0234	0.0035	C	Proliferative diabetic retinopathy	-0.0299	0.0185	C
rs838145	Alcohol intake frequency.	0.0220	0.0031	A	Proliferative diabetic retinopathy	-0.0230	0.0172	A
rs8614	Alcohol intake frequency.	0.0248	0.0039	A	Proliferative diabetic retinopathy	-0.0270	0.0227	A
rs9349379	Alcohol intake frequency.	-0.0193	0.0031	G	Proliferative diabetic retinopathy	-0.0021	0.0164	G
rs9372625	Alcohol intake frequency.	-0.0256	0.0031	A	Proliferative diabetic retinopathy	-0.0068	0.0176	A
rs9403297	Alcohol intake frequency.	0.0188	0.0031	A	Proliferative diabetic retinopathy	0.0240	0.0178	A
rs9648478	Alcohol intake frequency.	0.0169	0.0030	A	Proliferative diabetic retinopathy	0.0304	0.0164	A
rs9814516	Alcohol intake frequency.	-0.0251	0.0036	T	Proliferative diabetic retinopathy	-0.0047	0.0198	T
rs9829192	Alcohol intake frequency.	0.0169	0.0031	T	Proliferative diabetic retinopathy	-0.0090	0.0165	T
rs9906502	Alcohol intake frequency.	0.0238	0.0040	A	Proliferative diabetic retinopathy	-0.0036	0.0208	A
rs10085696	Alcoholic drinks per week	-0.0161	0.0025	G	Background diabetic retinopathy	-0.0370	0.0402	G
rs11860773	Alcoholic drinks per week	-0.0150	0.0024	C	Background diabetic retinopathy	0.0081	0.0415	C
rs1229984	Alcoholic drinks per week	0.1881	0.0062	C	Background diabetic retinopathy	0.2733	0.2271	C
rs1260326	Alcoholic drinks per week	0.0238	0.0020	C	Background diabetic retinopathy	0.0350	0.0344	C
rs13107325	Alcoholic drinks per week	-0.0365	0.0039	T	Background diabetic retinopathy	-0.1379	0.1391	T

rs13332432	Alcoholic drinks per week	0.0140	0.0021	G	Background diabetic retinopathy	0.0376	0.0378	G
rs1387766	Alcoholic drinks per week	-0.0108	0.0020	A	Background diabetic retinopathy	0.0777	0.0351	A
rs153106	Alcoholic drinks per week	-0.0136	0.0020	C	Background diabetic retinopathy	-0.0159	0.0327	C
rs16854020	Alcoholic drinks per week	0.0181	0.0029	A	Background diabetic retinopathy	0.0264	0.0579	A
rs17542254	Alcoholic drinks per week	0.0131	0.0021	G	Background diabetic retinopathy	0.0208	0.0392	G
rs2049045	Alcoholic drinks per week	-0.0138	0.0025	C	Background diabetic retinopathy	0.0993	0.0455	C
rs2299409	Alcoholic drinks per week	-0.0106	0.0019	A	Background diabetic retinopathy	-0.0464	0.0328	A
rs28601761	Alcoholic drinks per week	0.0113	0.0020	G	Background diabetic retinopathy	-0.0106	0.0332	G
rs28680958	Alcoholic drinks per week	-0.0136	0.0024	A	Background diabetic retinopathy	-0.0017	0.0414	A
rs28712821	Alcoholic drinks per week	0.0283	0.0020	A	Background diabetic retinopathy	0.0448	0.0344	A
rs28732378	Alcoholic drinks per week	-0.0167	0.0022	G	Background diabetic retinopathy	-0.0500	0.0382	G
rs28929474	Alcoholic drinks per week	-0.0477	0.0071	T	Background diabetic retinopathy	-0.1423	0.1136	T
rs331939	Alcoholic drinks per week	-0.0119	0.0020	A	Background diabetic retinopathy	0.0493	0.0362	A
rs34121753	Alcoholic drinks per week	0.0111	0.0020	G	Background diabetic retinopathy	-0.0535	0.0332	G
rs4309187	Alcoholic drinks per week	0.0148	0.0021	C	Background diabetic retinopathy	-0.0424	0.0436	C
rs4752999	Alcoholic drinks per week	-0.0146	0.0021	T	Background diabetic retinopathy	0.0269	0.0378	T
rs494904	Alcoholic drinks per week	0.0151	0.0020	C	Background diabetic retinopathy	-0.0482	0.0329	C
rs55872084	Alcoholic drinks per week	0.0127	0.0023	T	Background diabetic retinopathy	0.0279	0.0451	T
rs55932213	Alcoholic drinks per week	0.0125	0.0022	G	Background diabetic retinopathy	-0.0140	0.0371	G
rs6106989	Alcoholic drinks per week	0.0109	0.0020	A	Background diabetic retinopathy	0.0183	0.0350	A
rs6739804	Alcoholic drinks per week	-0.0130	0.0021	C	Background diabetic retinopathy	0.0093	0.0346	C
rs676388	Alcoholic drinks per week	0.0151	0.0019	C	Background diabetic retinopathy	0.1089	0.0334	C
rs6969458	Alcoholic drinks per week	0.0127	0.0019	A	Background diabetic retinopathy	-0.0255	0.0336	A
rs75120545	Alcoholic drinks per week	-0.0328	0.0057	T	Background diabetic retinopathy	0.1006	0.0784	T
rs76640332	Alcoholic drinks per week	-0.0210	0.0024	A	Background diabetic retinopathy	0.0423	0.0602	A
rs78234152	Alcoholic drinks per week	0.0277	0.0031	A	Background diabetic retinopathy	0.0084	0.0471	A
rs79616692	Alcoholic drinks per week	0.0188	0.0032	C	Background diabetic retinopathy	0.0187	0.0500	C
rs962961	Alcoholic drinks per week	-0.0122	0.0021	T	Background diabetic retinopathy	0.0112	0.0344	T
rs10085696	Alcoholic drinks per week	-0.0161	0.0025	G	Diabetic retinopathy	0.0167	0.0161	G
rs11860773	Alcoholic drinks per week	-0.0150	0.0024	C	Diabetic retinopathy	0.0023	0.0166	C
rs1229984	Alcoholic drinks per week	0.1881	0.0062	C	Diabetic retinopathy	-0.1272	0.0897	C

rs1260326	Alcoholic drinks per week	0.0238	0.0020	C	Diabetic retinopathy	0.0265	0.0138	C
rs13107325	Alcoholic drinks per week	-0.0365	0.0039	T	Diabetic retinopathy	0.0681	0.0561	T
rs13332432	Alcoholic drinks per week	0.0140	0.0021	G	Diabetic retinopathy	-0.0178	0.0152	G
rs1387766	Alcoholic drinks per week	-0.0108	0.0020	A	Diabetic retinopathy	-0.0028	0.0140	A
rs153106	Alcoholic drinks per week	-0.0136	0.0020	C	Diabetic retinopathy	-0.0225	0.0131	C
rs16854020	Alcoholic drinks per week	0.0181	0.0029	A	Diabetic retinopathy	0.0328	0.0234	A
rs17542254	Alcoholic drinks per week	0.0131	0.0021	G	Diabetic retinopathy	-0.0048	0.0158	G
rs2049045	Alcoholic drinks per week	-0.0138	0.0025	C	Diabetic retinopathy	0.0020	0.0183	C
rs2299409	Alcoholic drinks per week	-0.0106	0.0019	A	Diabetic retinopathy	-0.0100	0.0132	A
rs28601761	Alcoholic drinks per week	0.0113	0.0020	G	Diabetic retinopathy	-0.0204	0.0133	G
rs28680958	Alcoholic drinks per week	-0.0136	0.0024	A	Diabetic retinopathy	-0.0002	0.0165	A
rs28712821	Alcoholic drinks per week	0.0283	0.0020	A	Diabetic retinopathy	0.0104	0.0138	A
rs28732378	Alcoholic drinks per week	-0.0167	0.0022	G	Diabetic retinopathy	0.0042	0.0154	G
rs28929474	Alcoholic drinks per week	-0.0477	0.0071	T	Diabetic retinopathy	-0.0497	0.0464	T
rs331939	Alcoholic drinks per week	-0.0119	0.0020	A	Diabetic retinopathy	0.0125	0.0145	A
rs34121753	Alcoholic drinks per week	0.0111	0.0020	G	Diabetic retinopathy	-0.0150	0.0133	G
rs4309187	Alcoholic drinks per week	0.0148	0.0021	C	Diabetic retinopathy	0.0296	0.0174	C
rs4752999	Alcoholic drinks per week	-0.0146	0.0021	T	Diabetic retinopathy	0.0081	0.0152	T
rs494904	Alcoholic drinks per week	0.0151	0.0020	C	Diabetic retinopathy	-0.0148	0.0132	C
rs55872084	Alcoholic drinks per week	0.0127	0.0023	T	Diabetic retinopathy	0.0045	0.0180	T
rs55932213	Alcoholic drinks per week	0.0125	0.0022	G	Diabetic retinopathy	0.0034	0.0149	G
rs6106989	Alcoholic drinks per week	0.0109	0.0020	A	Diabetic retinopathy	0.0089	0.0140	A
rs6739804	Alcoholic drinks per week	-0.0130	0.0021	C	Diabetic retinopathy	0.0174	0.0139	C
rs676388	Alcoholic drinks per week	0.0151	0.0019	C	Diabetic retinopathy	0.0141	0.0133	C
rs6969458	Alcoholic drinks per week	0.0127	0.0019	A	Diabetic retinopathy	-0.0083	0.0135	A
rs75120545	Alcoholic drinks per week	-0.0328	0.0057	T	Diabetic retinopathy	0.0565	0.0317	T
rs76640332	Alcoholic drinks per week	-0.0210	0.0024	A	Diabetic retinopathy	0.0162	0.0241	A
rs78234152	Alcoholic drinks per week	0.0277	0.0031	A	Diabetic retinopathy	0.0171	0.0190	A
rs79616692	Alcoholic drinks per week	0.0188	0.0032	C	Diabetic retinopathy	-0.0120	0.0200	C
rs962961	Alcoholic drinks per week	-0.0122	0.0021	T	Diabetic retinopathy	-0.0032	0.0138	T
rs10085696	Alcoholic drinks per week	-0.0161	0.0025	G	Proliferative diabetic retinopathy	0.0211	0.0201	G

rs11860773	Alcoholic drinks per week	-0.0150	0.0024	C	Proliferative diabetic retinopathy	-0.0062	0.0208	C
rs1229984	Alcoholic drinks per week	0.1881	0.0062	C	Proliferative diabetic retinopathy	-0.1707	0.1131	C
rs1260326	Alcoholic drinks per week	0.0238	0.0020	C	Proliferative diabetic retinopathy	0.0263	0.0172	C
rs13107325	Alcoholic drinks per week	-0.0365	0.0039	T	Proliferative diabetic retinopathy	0.0350	0.0692	T
rs13332432	Alcoholic drinks per week	0.0140	0.0021	G	Proliferative diabetic retinopathy	-0.0140	0.0189	G
rs1387766	Alcoholic drinks per week	-0.0108	0.0020	A	Proliferative diabetic retinopathy	0.0025	0.0175	A
rs153106	Alcoholic drinks per week	-0.0136	0.0020	C	Proliferative diabetic retinopathy	-0.0371	0.0164	C
rs16854020	Alcoholic drinks per week	0.0181	0.0029	A	Proliferative diabetic retinopathy	0.0433	0.0291	A
rs17542254	Alcoholic drinks per week	0.0131	0.0021	G	Proliferative diabetic retinopathy	-0.0062	0.0197	G
rs2049045	Alcoholic drinks per week	-0.0138	0.0025	C	Proliferative diabetic retinopathy	0.0356	0.0228	C
rs2299409	Alcoholic drinks per week	-0.0106	0.0019	A	Proliferative diabetic retinopathy	-0.0174	0.0164	A
rs28601761	Alcoholic drinks per week	0.0113	0.0020	G	Proliferative diabetic retinopathy	-0.0382	0.0167	G
rs28680958	Alcoholic drinks per week	-0.0136	0.0024	A	Proliferative diabetic retinopathy	0.0197	0.0207	A
rs28712821	Alcoholic drinks per week	0.0283	0.0020	A	Proliferative diabetic retinopathy	0.0168	0.0172	A
rs28732378	Alcoholic drinks per week	-0.0167	0.0022	G	Proliferative diabetic retinopathy	0.0013	0.0192	G
rs28929474	Alcoholic drinks per week	-0.0477	0.0071	T	Proliferative diabetic retinopathy	0.0310	0.0579	T
rs331939	Alcoholic drinks per week	-0.0119	0.0020	A	Proliferative diabetic retinopathy	0.0063	0.0181	A
rs34121753	Alcoholic drinks per week	0.0111	0.0020	G	Proliferative diabetic retinopathy	-0.0347	0.0166	G
rs4309187	Alcoholic drinks per week	0.0148	0.0021	C	Proliferative diabetic retinopathy	0.0240	0.0218	C
rs4752999	Alcoholic drinks per week	-0.0146	0.0021	T	Proliferative diabetic retinopathy	0.0058	0.0189	T
rs494904	Alcoholic drinks per week	0.0151	0.0020	C	Proliferative diabetic retinopathy	-0.0060	0.0165	C
rs55872084	Alcoholic drinks per week	0.0127	0.0023	T	Proliferative diabetic retinopathy	0.0014	0.0224	T
rs55932213	Alcoholic drinks per week	0.0125	0.0022	G	Proliferative diabetic retinopathy	-0.0019	0.0186	G
rs6106989	Alcoholic drinks per week	0.0109	0.0020	A	Proliferative diabetic retinopathy	0.0134	0.0175	A
rs6739804	Alcoholic drinks per week	-0.0130	0.0021	C	Proliferative diabetic retinopathy	0.0046	0.0173	C
rs676388	Alcoholic drinks per week	0.0151	0.0019	C	Proliferative diabetic retinopathy	0.0091	0.0167	C
rs6969458	Alcoholic drinks per week	0.0127	0.0019	A	Proliferative diabetic retinopathy	-0.0043	0.0168	A
rs75120545	Alcoholic drinks per week	-0.0328	0.0057	T	Proliferative diabetic retinopathy	0.0330	0.0394	T
rs76640332	Alcoholic drinks per week	-0.0210	0.0024	A	Proliferative diabetic retinopathy	0.0104	0.0300	A
rs78234152	Alcoholic drinks per week	0.0277	0.0031	A	Proliferative diabetic retinopathy	0.0120	0.0236	A
rs79616692	Alcoholic drinks per week	0.0188	0.0032	C	Proliferative diabetic retinopathy	0.0199	0.0250	C

rs962961	Alcoholic drinks per week	-0.0122	0.0021	T	Proliferative diabetic retinopathy	0.0026	0.0172	T
rs10100245	Body mass index	0.0206	0.0024	A	Background diabetic retinopathy	0.0142	0.0329	A
rs10187101	Body mass index	-0.0157	0.0025	T	Background diabetic retinopathy	-0.0363	0.0331	T
rs10404726	Body mass index	-0.0199	0.0024	T	Background diabetic retinopathy	-0.0165	0.0327	T
rs10465231	Body mass index	0.0171	0.0024	T	Background diabetic retinopathy	-0.0176	0.0329	T
rs1064213	Body mass index	0.0162	0.0024	A	Background diabetic retinopathy	-0.0152	0.0335	A
rs10788493	Body mass index	0.0137	0.0024	T	Background diabetic retinopathy	0.0877	0.0332	T
rs10803762	Body mass index	0.0155	0.0026	A	Background diabetic retinopathy	-0.0373	0.0360	A
rs10805383	Body mass index	0.0169	0.0024	A	Background diabetic retinopathy	0.0090	0.0334	A
rs10865612	Body mass index	-0.0231	0.0025	C	Background diabetic retinopathy	0.0411	0.0332	C
rs10898330	Body mass index	-0.0143	0.0024	T	Background diabetic retinopathy	-0.0097	0.0332	T
rs10938397	Body mass index	0.0290	0.0024	G	Background diabetic retinopathy	0.0980	0.0328	G
rs10995427	Body mass index	-0.0165	0.0025	A	Background diabetic retinopathy	0.0038	0.0338	A
rs11012732	Body mass index	0.0238	0.0026	G	Background diabetic retinopathy	0.0851	0.0352	G
rs11078883	Body mass index	0.0157	0.0025	G	Background diabetic retinopathy	0.0466	0.0353	G
rs11084554	Body mass index	-0.0216	0.0033	A	Background diabetic retinopathy	-0.0357	0.0485	A
rs11099020	Body mass index	-0.0153	0.0025	T	Background diabetic retinopathy	-0.0741	0.0338	T
rs11150745	Body mass index	-0.0210	0.0026	G	Background diabetic retinopathy	-0.0270	0.0366	G
rs111640872	Body mass index	0.0206	0.0026	C	Background diabetic retinopathy	-0.0322	0.0345	C
rs11223641	Body mass index	-0.0191	0.0034	C	Background diabetic retinopathy	-0.0406	0.0665	C
rs112520079	Body mass index	0.0205	0.0030	G	Background diabetic retinopathy	0.0370	0.0481	G
rs11264489	Body mass index	0.0141	0.0025	G	Background diabetic retinopathy	0.0013	0.0366	G
rs112693590	Body mass index	-0.0329	0.0057	A	Background diabetic retinopathy	0.0011	0.0572	A
rs1127100	Body mass index	0.0165	0.0025	C	Background diabetic retinopathy	-0.0619	0.0346	C
rs113182412	Body mass index	-0.0194	0.0033	A	Background diabetic retinopathy	0.0574	0.0546	A
rs113230003	Body mass index	-0.0198	0.0028	A	Background diabetic retinopathy	-0.0173	0.0402	A
rs113603865	Body mass index	0.0197	0.0030	T	Background diabetic retinopathy	0.0449	0.0416	T
rs11515071	Body mass index	-0.0228	0.0025	T	Background diabetic retinopathy	-0.0609	0.0330	T
rs11642015	Body mass index	0.0724	0.0024	T	Background diabetic retinopathy	0.0769	0.0332	T
rs11650012	Body mass index	0.0186	0.0032	A	Background diabetic retinopathy	0.0172	0.0458	A
rs11655587	Body mass index	-0.0196	0.0025	T	Background diabetic retinopathy	-0.0665	0.0344	T

rs11666480	Body mass index	0.0185	0.0024	G	Background diabetic retinopathy	0.0281	0.0333	G
rs11742930	Body mass index	0.0143	0.0024	T	Background diabetic retinopathy	0.0591	0.0329	T
rs11757278	Body mass index	-0.0158	0.0026	C	Background diabetic retinopathy	0.0492	0.0336	C
rs11761411	Body mass index	-0.0185	0.0033	T	Background diabetic retinopathy	-0.0502	0.0435	T
rs117632017	Body mass index	0.0364	0.0064	A	Background diabetic retinopathy	-0.1380	0.1526	A
rs11782074	Body mass index	0.0145	0.0025	T	Background diabetic retinopathy	0.0062	0.0340	T
rs11856579	Body mass index	-0.0198	0.0027	A	Background diabetic retinopathy	0.1000	0.0401	A
rs12024554	Body mass index	-0.0163	0.0028	T	Background diabetic retinopathy	0.0431	0.0393	T
rs12042959	Body mass index	-0.0206	0.0034	G	Background diabetic retinopathy	-0.0735	0.0448	G
rs12049202	Body mass index	0.0218	0.0030	T	Background diabetic retinopathy	0.0784	0.0364	T
rs12140153	Body mass index	-0.0318	0.0042	T	Background diabetic retinopathy	-0.1447	0.0617	T
rs12144626	Body mass index	-0.0172	0.0024	C	Background diabetic retinopathy	-0.0070	0.0338	C
rs12477385	Body mass index	-0.0173	0.0029	T	Background diabetic retinopathy	-0.0569	0.0376	T
rs12479357	Body mass index	0.0179	0.0025	G	Background diabetic retinopathy	0.0036	0.0341	G
rs12614861	Body mass index	0.0152	0.0025	T	Background diabetic retinopathy	-0.0535	0.0350	T
rs12622280	Body mass index	-0.0187	0.0033	G	Background diabetic retinopathy	-0.0255	0.0506	G
rs12662900	Body mass index	-0.0169	0.0027	A	Background diabetic retinopathy	-0.0367	0.0353	A
rs12679106	Body mass index	-0.0220	0.0027	T	Background diabetic retinopathy	-0.0015	0.0369	T
rs1286138	Body mass index	0.0181	0.0026	G	Background diabetic retinopathy	0.0002	0.0365	G
rs12877270	Body mass index	0.0169	0.0024	A	Background diabetic retinopathy	-0.0425	0.0329	A
rs12881629	Body mass index	0.0240	0.0044	G	Background diabetic retinopathy	0.0006	0.0623	G
rs12885458	Body mass index	-0.0156	0.0024	G	Background diabetic retinopathy	-0.0336	0.0333	G
rs1296328	Body mass index	-0.0190	0.0024	C	Background diabetic retinopathy	0.0767	0.0328	C
rs12977259	Body mass index	0.0186	0.0032	G	Background diabetic retinopathy	0.0030	0.0486	G
rs12992672	Body mass index	0.0508	0.0032	A	Background diabetic retinopathy	0.0342	0.0444	A
rs13047416	Body mass index	-0.0136	0.0025	G	Background diabetic retinopathy	0.0287	0.0329	G
rs13062093	Body mass index	0.0171	0.0025	G	Background diabetic retinopathy	0.0587	0.0346	G
rs13076052	Body mass index	0.0161	0.0027	G	Background diabetic retinopathy	0.0370	0.0342	G
rs13135092	Body mass index	0.0501	0.0044	G	Background diabetic retinopathy	-0.1330	0.1247	G
rs13174863	Body mass index	0.0250	0.0034	G	Background diabetic retinopathy	0.0619	0.0462	G
rs1320903	Body mass index	0.0220	0.0026	A	Background diabetic retinopathy	-0.0081	0.0360	A

rs1327259	Body mass index	-0.0140	0.0025	G	Background diabetic retinopathy	0.0394	0.0330	G
rs1342391	Body mass index	0.0159	0.0026	T	Background diabetic retinopathy	0.0269	0.0354	T
rs13427822	Body mass index	-0.0199	0.0027	G	Background diabetic retinopathy	0.0271	0.0358	G
rs1411432	Body mass index	0.0249	0.0031	C	Background diabetic retinopathy	0.0322	0.0431	C
rs1412239	Body mass index	0.0239	0.0026	G	Background diabetic retinopathy	0.0140	0.0339	G
rs1441264	Body mass index	0.0206	0.0025	A	Background diabetic retinopathy	-0.0698	0.0350	A
rs1446585	Body mass index	-0.0171	0.0029	G	Background diabetic retinopathy	0.0057	0.0346	G
rs1454687	Body mass index	-0.0225	0.0024	G	Background diabetic retinopathy	-0.0522	0.0330	G
rs1458156	Body mass index	0.0138	0.0024	T	Background diabetic retinopathy	-0.0120	0.0329	T
rs1477290	Body mass index	0.0335	0.0035	C	Background diabetic retinopathy	0.0373	0.0502	C
rs147730268	Body mass index	-0.0361	0.0043	T	Background diabetic retinopathy	-0.0129	0.0595	T
rs1582931	Body mass index	-0.0149	0.0024	A	Background diabetic retinopathy	-0.0139	0.0328	A
rs16846140	Body mass index	0.0162	0.0025	G	Background diabetic retinopathy	-0.0374	0.0348	G
rs16916303	Body mass index	-0.0207	0.0037	G	Background diabetic retinopathy	-0.0164	0.0490	G
rs16975459	Body mass index	0.0240	0.0037	C	Background diabetic retinopathy	0.0122	0.0426	C
rs17014332	Body mass index	0.0180	0.0029	C	Background diabetic retinopathy	-0.0315	0.0415	C
rs17024393	Body mass index	0.0675	0.0076	C	Background diabetic retinopathy	-0.0223	0.0708	C
rs17058884	Body mass index	-0.0318	0.0058	G	Background diabetic retinopathy	0.0115	0.0716	G
rs17085463	Body mass index	-0.0148	0.0026	A	Background diabetic retinopathy	0.0352	0.0351	A
rs17149254	Body mass index	-0.0238	0.0031	C	Background diabetic retinopathy	0.0553	0.0401	C
rs17342242	Body mass index	-0.0162	0.0029	G	Background diabetic retinopathy	0.0181	0.0439	G
rs17399739	Body mass index	0.0262	0.0047	G	Background diabetic retinopathy	0.0228	0.0580	G
rs17716502	Body mass index	-0.0223	0.0030	T	Background diabetic retinopathy	0.0175	0.0414	T
rs1788808	Body mass index	-0.0183	0.0024	G	Background diabetic retinopathy	-0.0153	0.0329	G
rs1805123	Body mass index	-0.0180	0.0028	G	Background diabetic retinopathy	-0.0278	0.0385	G
rs1840661	Body mass index	0.0137	0.0024	A	Background diabetic retinopathy	0.0084	0.0333	A
rs1884897	Body mass index	0.0213	0.0025	G	Background diabetic retinopathy	-0.0428	0.0350	G
rs1901241	Body mass index	0.0193	0.0033	G	Background diabetic retinopathy	-0.0143	0.0416	G
rs1919243	Body mass index	0.0139	0.0024	C	Background diabetic retinopathy	0.0138	0.0333	C
rs1941706	Body mass index	0.0139	0.0024	G	Background diabetic retinopathy	-0.0202	0.0328	G
rs1949204	Body mass index	0.0167	0.0028	G	Background diabetic retinopathy	0.0228	0.0368	G

rs2035806	Body mass index	-0.0166	0.0024	A	Background diabetic retinopathy	0.0089	0.0328	A
rs2046002	Body mass index	-0.0146	0.0025	C	Background diabetic retinopathy	-0.0634	0.0328	C
rs2121058	Body mass index	-0.0238	0.0029	C	Background diabetic retinopathy	-0.1100	0.0388	C
rs2135745	Body mass index	-0.0168	0.0028	G	Background diabetic retinopathy	0.0444	0.0395	G
rs2155869	Body mass index	-0.0184	0.0031	C	Background diabetic retinopathy	0.0346	0.0402	C
rs215634	Body mass index	-0.0148	0.0025	G	Background diabetic retinopathy	-0.0130	0.0363	G
rs2192649	Body mass index	0.0137	0.0024	G	Background diabetic retinopathy	0.0169	0.0327	G
rs2234458	Body mass index	-0.0206	0.0025	T	Background diabetic retinopathy	-0.0134	0.0357	T
rs2253310	Body mass index	0.0173	0.0025	G	Background diabetic retinopathy	0.0285	0.0332	G
rs2292238	Body mass index	-0.0177	0.0024	C	Background diabetic retinopathy	0.1214	0.0334	C
rs2307111	Body mass index	-0.0290	0.0025	C	Background diabetic retinopathy	-0.0505	0.0332	C
rs2318543	Body mass index	-0.0194	0.0029	G	Background diabetic retinopathy	-0.0872	0.0395	G
rs2384054	Body mass index	0.0350	0.0024	C	Background diabetic retinopathy	0.0504	0.0332	C
rs2398861	Body mass index	0.0221	0.0028	G	Background diabetic retinopathy	-0.0379	0.0353	G
rs241460	Body mass index	-0.0205	0.0026	G	Background diabetic retinopathy	0.0076	0.0337	G
rs2425857	Body mass index	-0.0136	0.0024	G	Background diabetic retinopathy	-0.0307	0.0344	G
rs2439823	Body mass index	0.0216	0.0024	G	Background diabetic retinopathy	-0.0423	0.0329	G
rs2450254	Body mass index	-0.0141	0.0024	T	Background diabetic retinopathy	-0.0367	0.0345	T
rs2450445	Body mass index	-0.0154	0.0026	A	Background diabetic retinopathy	-0.0371	0.0354	A
rs245775	Body mass index	0.0203	0.0027	G	Background diabetic retinopathy	-0.0258	0.0350	G
rs2470392	Body mass index	0.0145	0.0027	C	Background diabetic retinopathy	-0.0177	0.0340	C
rs2474898	Body mass index	0.0146	0.0025	T	Background diabetic retinopathy	0.0202	0.0331	T
rs2482704	Body mass index	-0.0138	0.0024	T	Background diabetic retinopathy	0.0462	0.0340	T
rs2606228	Body mass index	-0.0156	0.0025	C	Background diabetic retinopathy	-0.0373	0.0333	C
rs2616192	Body mass index	0.0141	0.0026	T	Background diabetic retinopathy	0.0596	0.0338	T
rs2678204	Body mass index	0.0281	0.0025	G	Background diabetic retinopathy	0.0600	0.0359	G
rs2711111	Body mass index	-0.0147	0.0025	G	Background diabetic retinopathy	0.0014	0.0335	G
rs2725371	Body mass index	-0.0181	0.0026	G	Background diabetic retinopathy	-0.0649	0.0374	G
rs273505	Body mass index	0.0189	0.0024	C	Background diabetic retinopathy	0.0254	0.0326	C
rs28366156	Body mass index	-0.0290	0.0036	C	Background diabetic retinopathy	-0.2273	0.0634	C
rs28447555	Body mass index	0.0185	0.0031	T	Background diabetic retinopathy	0.0176	0.0469	T

rs28489620	Body mass index	-0.0147	0.0027	A	Background diabetic retinopathy	0.0225	0.0360	A
rs2861685	Body mass index	-0.0176	0.0024	C	Background diabetic retinopathy	0.0057	0.0332	C
rs286818	Body mass index	-0.0291	0.0032	A	Background diabetic retinopathy	0.0787	0.0402	A
rs2962082	Body mass index	-0.0135	0.0024	A	Background diabetic retinopathy	-0.0401	0.0329	A
rs2975693	Body mass index	0.0238	0.0039	C	Background diabetic retinopathy	0.0078	0.0642	C
rs34045288	Body mass index	0.0260	0.0025	T	Background diabetic retinopathy	0.0765	0.0339	T
rs34095326	Body mass index	-0.0244	0.0038	A	Background diabetic retinopathy	0.0146	0.0444	A
rs34236292	Body mass index	-0.0141	0.0026	T	Background diabetic retinopathy	0.0070	0.0377	T
rs34361149	Body mass index	-0.0310	0.0030	A	Background diabetic retinopathy	-0.0180	0.0477	A
rs34373881	Body mass index	-0.0169	0.0027	A	Background diabetic retinopathy	-0.0358	0.0373	A
rs34774377	Body mass index	-0.0208	0.0037	C	Background diabetic retinopathy	0.0377	0.0530	C
rs34811474	Body mass index	-0.0306	0.0028	A	Background diabetic retinopathy	0.0171	0.0388	A
rs34966008	Body mass index	-0.0199	0.0024	T	Background diabetic retinopathy	-0.0441	0.0340	T
rs35025195	Body mass index	-0.0230	0.0032	A	Background diabetic retinopathy	-0.0807	0.0475	A
rs35193668	Body mass index	-0.0167	0.0025	T	Background diabetic retinopathy	-0.0699	0.0331	T
rs35483388	Body mass index	0.0141	0.0025	T	Background diabetic retinopathy	0.0064	0.0333	T
rs35626515	Body mass index	0.0266	0.0024	A	Background diabetic retinopathy	-0.0286	0.0328	A
rs35722922	Body mass index	-0.0165	0.0025	G	Background diabetic retinopathy	-0.0595	0.0350	G
rs357501	Body mass index	0.0154	0.0025	A	Background diabetic retinopathy	-0.0258	0.0333	A
rs35851183	Body mass index	0.0168	0.0025	G	Background diabetic retinopathy	0.0300	0.0357	G
rs35882248	Body mass index	0.0192	0.0026	T	Background diabetic retinopathy	-0.0243	0.0353	T
rs36007635	Body mass index	-0.0200	0.0035	A	Background diabetic retinopathy	-0.1002	0.0571	A
rs362307	Body mass index	0.0314	0.0046	T	Background diabetic retinopathy	-0.0147	0.0653	T
rs3759584	Body mass index	-0.0166	0.0025	C	Background diabetic retinopathy	-0.0314	0.0353	C
rs3802858	Body mass index	-0.0165	0.0024	C	Background diabetic retinopathy	0.0167	0.0330	C
rs3803286	Body mass index	-0.0208	0.0025	G	Background diabetic retinopathy	-0.0107	0.0345	G
rs3810291	Body mass index	0.0297	0.0026	A	Background diabetic retinopathy	0.0728	0.0340	A
rs3843540	Body mass index	-0.0242	0.0034	C	Background diabetic retinopathy	0.0565	0.0392	C
rs3844598	Body mass index	0.0137	0.0024	G	Background diabetic retinopathy	-0.0103	0.0330	G
rs3861879	Body mass index	0.0137	0.0024	G	Background diabetic retinopathy	-0.0259	0.0333	G
rs3897102	Body mass index	0.0147	0.0025	T	Background diabetic retinopathy	0.0344	0.0331	T

rs390192	Body mass index	-0.0154	0.0024	G	Background diabetic retinopathy	-0.0369	0.0329	G
rs39330	Body mass index	-0.0189	0.0024	T	Background diabetic retinopathy	-0.0165	0.0330	T
rs4246657	Body mass index	0.0170	0.0025	T	Background diabetic retinopathy	-0.0202	0.0352	T
rs4261944	Body mass index	0.0155	0.0025	G	Background diabetic retinopathy	-0.0160	0.0357	G
rs4402589	Body mass index	0.0292	0.0024	G	Background diabetic retinopathy	0.0467	0.0328	G
rs4467770	Body mass index	0.0157	0.0027	A	Background diabetic retinopathy	0.0179	0.0364	A
rs4474229	Body mass index	-0.0159	0.0025	A	Background diabetic retinopathy	0.0174	0.0346	A
rs4482463	Body mass index	-0.0341	0.0045	A	Background diabetic retinopathy	0.0237	0.0698	A
rs4502882	Body mass index	-0.0147	0.0025	T	Background diabetic retinopathy	0.0004	0.0359	T
rs4595495	Body mass index	0.0137	0.0024	G	Background diabetic retinopathy	-0.0381	0.0329	G
rs4648450	Body mass index	-0.0154	0.0024	A	Background diabetic retinopathy	-0.0452	0.0331	A
rs4687770	Body mass index	-0.0193	0.0035	C	Background diabetic retinopathy	0.0431	0.0450	C
rs4718964	Body mass index	0.0146	0.0024	T	Background diabetic retinopathy	-0.0313	0.0335	T
rs4757144	Body mass index	0.0165	0.0024	A	Background diabetic retinopathy	-0.0067	0.0327	A
rs4776970	Body mass index	-0.0255	0.0025	T	Background diabetic retinopathy	-0.0013	0.0344	T
rs4777541	Body mass index	0.0197	0.0028	T	Background diabetic retinopathy	0.0654	0.0472	T
rs4911382	Body mass index	0.0156	0.0024	T	Background diabetic retinopathy	-0.0053	0.0343	T
rs491711	Body mass index	-0.0158	0.0026	C	Background diabetic retinopathy	-0.0292	0.0391	C
rs4921301	Body mass index	-0.0182	0.0030	T	Background diabetic retinopathy	0.0248	0.0403	T
rs4929923	Body mass index	0.0174	0.0025	C	Background diabetic retinopathy	-0.0212	0.0343	C
rs525101	Body mass index	0.0165	0.0025	C	Background diabetic retinopathy	-0.0176	0.0330	C
rs539515	Body mass index	0.0475	0.0030	C	Background diabetic retinopathy	0.0138	0.0427	C
rs550974	Body mass index	0.0159	0.0024	T	Background diabetic retinopathy	-0.0339	0.0361	T
rs55689274	Body mass index	-0.0148	0.0027	A	Background diabetic retinopathy	0.0027	0.0370	A
rs55726687	Body mass index	0.0239	0.0029	A	Background diabetic retinopathy	0.0358	0.0398	A
rs55886426	Body mass index	-0.0316	0.0055	G	Background diabetic retinopathy	0.0484	0.0817	G
rs55938344	Body mass index	-0.0168	0.0028	C	Background diabetic retinopathy	0.0529	0.0378	C
rs56067609	Body mass index	-0.0194	0.0035	T	Background diabetic retinopathy	-0.0181	0.0477	T
rs56161855	Body mass index	0.0230	0.0035	T	Background diabetic retinopathy	-0.0936	0.0456	T
rs56212061	Body mass index	-0.0211	0.0034	T	Background diabetic retinopathy	-0.0169	0.0558	T
rs56773984	Body mass index	-0.0179	0.0032	T	Background diabetic retinopathy	0.0153	0.0740	T

rs56803094	Body mass index	-0.0189	0.0029	G	Background diabetic retinopathy	-0.0004	0.0368	G
rs57636386	Body mass index	-0.0428	0.0044	C	Background diabetic retinopathy	0.0848	0.0758	C
rs58862095	Body mass index	-0.0244	0.0024	T	Background diabetic retinopathy	-0.0235	0.0328	T
rs588660	Body mass index	0.0183	0.0024	A	Background diabetic retinopathy	-0.0023	0.0335	A
rs59104534	Body mass index	0.0150	0.0026	T	Background diabetic retinopathy	-0.0102	0.0350	T
rs5995843	Body mass index	-0.0174	0.0025	G	Background diabetic retinopathy	0.0082	0.0339	G
rs6050446	Body mass index	0.0421	0.0068	G	Background diabetic retinopathy	-0.0138	0.1118	G
rs60654199	Body mass index	0.0313	0.0048	A	Background diabetic retinopathy	-0.0483	0.0764	A
rs60764613	Body mass index	0.0236	0.0034	T	Background diabetic retinopathy	0.0444	0.0433	T
rs61813324	Body mass index	0.0279	0.0036	T	Background diabetic retinopathy	-0.0420	0.0454	T
rs61826867	Body mass index	0.0251	0.0038	G	Background diabetic retinopathy	0.0692	0.0594	G
rs61871615	Body mass index	-0.0280	0.0044	T	Background diabetic retinopathy	-0.0874	0.0608	T
rs61903695	Body mass index	0.0164	0.0028	G	Background diabetic retinopathy	0.0107	0.0380	G
rs61969510	Body mass index	0.0155	0.0027	C	Background diabetic retinopathy	0.0147	0.0340	C
rs62106258	Body mass index	-0.0907	0.0056	C	Background diabetic retinopathy	0.0851	0.1150	C
rs62147189	Body mass index	-0.0170	0.0025	G	Background diabetic retinopathy	0.0249	0.0333	G
rs62246314	Body mass index	0.0228	0.0040	A	Background diabetic retinopathy	0.0316	0.0669	A
rs62407562	Body mass index	0.0171	0.0027	A	Background diabetic retinopathy	0.0441	0.0419	A
rs62543438	Body mass index	-0.0148	0.0027	C	Background diabetic retinopathy	-0.0413	0.0403	C
rs6265	Body mass index	-0.0402	0.0031	T	Background diabetic retinopathy	0.0897	0.0452	T
rs6536575	Body mass index	0.0141	0.0024	C	Background diabetic retinopathy	-0.0102	0.0332	C
rs6575340	Body mass index	0.0229	0.0025	A	Background diabetic retinopathy	-0.0190	0.0339	A
rs6601527	Body mass index	-0.0217	0.0024	A	Background diabetic retinopathy	-0.0450	0.0473	A
rs66679256	Body mass index	0.0163	0.0024	T	Background diabetic retinopathy	-0.0165	0.0340	T
rs6687953	Body mass index	0.0159	0.0025	G	Background diabetic retinopathy	-0.0290	0.0329	G
rs66922415	Body mass index	0.0521	0.0028	G	Background diabetic retinopathy	0.0546	0.0420	G
rs6705567	Body mass index	-0.0136	0.0025	C	Background diabetic retinopathy	-0.0574	0.0337	C
rs6722241	Body mass index	-0.0202	0.0027	C	Background diabetic retinopathy	0.0161	0.0418	C
rs6739755	Body mass index	-0.0210	0.0025	G	Background diabetic retinopathy	-0.0821	0.0333	G
rs67609008	Body mass index	0.0146	0.0027	C	Background diabetic retinopathy	-0.0283	0.0428	C
rs6780459	Body mass index	0.0164	0.0028	T	Background diabetic retinopathy	-0.0038	0.0399	T

rs67844506	Body mass index	-0.0259	0.0031	G	Background diabetic retinopathy	0.0282	0.0448	G
rs6789488	Body mass index	0.0200	0.0028	C	Background diabetic retinopathy	-0.0021	0.0458	C
rs6809307	Body mass index	0.0157	0.0028	T	Background diabetic retinopathy	-0.0530	0.0429	T
rs6831020	Body mass index	-0.0159	0.0026	A	Background diabetic retinopathy	0.0388	0.0387	A
rs6861649	Body mass index	0.0143	0.0025	C	Background diabetic retinopathy	0.0361	0.0339	C
rs6950388	Body mass index	0.0172	0.0030	A	Background diabetic retinopathy	0.0248	0.0393	A
rs7006178	Body mass index	0.0155	0.0027	C	Background diabetic retinopathy	0.0512	0.0379	C
rs7030732	Body mass index	-0.0152	0.0025	A	Background diabetic retinopathy	-0.0520	0.0338	A
rs704061	Body mass index	0.0159	0.0024	C	Background diabetic retinopathy	-0.0077	0.0339	C
rs7094644	Body mass index	0.0153	0.0026	A	Background diabetic retinopathy	0.0126	0.0357	A
rs7116641	Body mass index	0.0244	0.0026	G	Background diabetic retinopathy	0.0599	0.0354	G
rs7124681	Body mass index	0.0267	0.0024	A	Background diabetic retinopathy	0.0601	0.0343	A
rs7132908	Body mass index	0.0285	0.0025	A	Background diabetic retinopathy	0.0265	0.0338	A
rs7138383	Body mass index	-0.0214	0.0028	A	Background diabetic retinopathy	-0.0370	0.0356	A
rs7141420	Body mass index	0.0208	0.0024	T	Background diabetic retinopathy	-0.0141	0.0333	T
rs71495049	Body mass index	0.0265	0.0043	A	Background diabetic retinopathy	0.0049	0.0516	A
rs7163362	Body mass index	0.0132	0.0024	A	Background diabetic retinopathy	-0.0114	0.0340	A
rs7183417	Body mass index	0.0149	0.0024	T	Background diabetic retinopathy	0.0545	0.0350	T
rs7189149	Body mass index	0.0213	0.0036	G	Background diabetic retinopathy	-0.0917	0.0504	G
rs7195386	Body mass index	-0.0159	0.0024	C	Background diabetic retinopathy	0.0127	0.0328	C
rs7201895	Body mass index	-0.0173	0.0025	A	Background diabetic retinopathy	-0.0014	0.0359	A
rs7218014	Body mass index	0.0207	0.0030	C	Background diabetic retinopathy	0.0596	0.0385	C
rs72697614	Body mass index	0.0150	0.0026	A	Background diabetic retinopathy	0.0011	0.0362	A
rs72820274	Body mass index	0.0160	0.0024	A	Background diabetic retinopathy	0.0094	0.0331	A
rs72892910	Body mass index	0.0402	0.0032	T	Background diabetic retinopathy	-0.0063	0.0406	T
rs72976986	Body mass index	-0.0240	0.0031	A	Background diabetic retinopathy	-0.0426	0.0448	A
rs73050254	Body mass index	0.0193	0.0035	A	Background diabetic retinopathy	-0.0238	0.0646	A
rs73144053	Body mass index	-0.0154	0.0026	A	Background diabetic retinopathy	-0.0365	0.0356	A
rs73169730	Body mass index	0.0195	0.0027	G	Background diabetic retinopathy	0.0221	0.0378	G
rs7321331	Body mass index	0.0176	0.0028	A	Background diabetic retinopathy	0.0359	0.0350	A
rs73213484	Body mass index	-0.0212	0.0035	T	Background diabetic retinopathy	0.0360	0.0461	T

rs7331420	Body mass index	-0.0147	0.0027	A	Background diabetic retinopathy	-0.0106	0.0362	A
rs7442885	Body mass index	-0.0254	0.0030	G	Background diabetic retinopathy	0.0686	0.0419	G
rs7498044	Body mass index	-0.0176	0.0029	A	Background diabetic retinopathy	-0.0017	0.0379	A
rs750090	Body mass index	-0.0156	0.0025	C	Background diabetic retinopathy	-0.0162	0.0333	C
rs752179	Body mass index	-0.0144	0.0026	A	Background diabetic retinopathy	0.0165	0.0346	A
rs75499503	Body mass index	-0.0198	0.0029	T	Background diabetic retinopathy	0.0271	0.0389	T
rs7553158	Body mass index	-0.0174	0.0024	A	Background diabetic retinopathy	-0.0279	0.0326	A
rs75557510	Body mass index	-0.0378	0.0051	G	Background diabetic retinopathy	0.0409	0.0764	G
rs756717	Body mass index	-0.0147	0.0025	A	Background diabetic retinopathy	0.0868	0.0335	A
rs7568228	Body mass index	-0.0140	0.0024	C	Background diabetic retinopathy	0.0315	0.0329	C
rs76040172	Body mass index	-0.0408	0.0053	A	Background diabetic retinopathy	-0.0970	0.0598	A
rs7701777	Body mass index	-0.0170	0.0027	G	Background diabetic retinopathy	0.0632	0.0381	G
rs7719067	Body mass index	-0.0162	0.0024	G	Background diabetic retinopathy	-0.0195	0.0334	G
rs7723426	Body mass index	0.0147	0.0026	C	Background diabetic retinopathy	0.0340	0.0331	C
rs7755574	Body mass index	0.0155	0.0027	T	Background diabetic retinopathy	0.0462	0.0348	T
rs7774	Body mass index	0.0166	0.0026	A	Background diabetic retinopathy	0.0014	0.0336	A
rs778094	Body mass index	-0.0149	0.0024	A	Background diabetic retinopathy	-0.0304	0.0329	A
rs7852189	Body mass index	0.0166	0.0026	G	Background diabetic retinopathy	0.0333	0.0337	G
rs78565420	Body mass index	0.0337	0.0055	T	Background diabetic retinopathy	0.0800	0.0833	T
rs79113395	Body mass index	-0.0209	0.0027	A	Background diabetic retinopathy	-0.0251	0.0396	A
rs7933085	Body mass index	0.0157	0.0024	G	Background diabetic retinopathy	0.0076	0.0328	G
rs7941828	Body mass index	-0.0156	0.0025	T	Background diabetic retinopathy	-0.0263	0.0355	T
rs7952102	Body mass index	-0.0152	0.0025	C	Background diabetic retinopathy	-0.0023	0.0343	C
rs7992832	Body mass index	-0.0176	0.0027	T	Background diabetic retinopathy	-0.0642	0.0377	T
rs799449	Body mass index	0.0199	0.0024	T	Background diabetic retinopathy	-0.0041	0.0332	T
rs8015400	Body mass index	0.0211	0.0026	A	Background diabetic retinopathy	0.0068	0.0341	A
rs80330591	Body mass index	-0.0214	0.0034	A	Background diabetic retinopathy	-0.0240	0.0426	A
rs8078135	Body mass index	-0.0195	0.0024	T	Background diabetic retinopathy	0.0134	0.0328	T
rs8134638	Body mass index	0.0152	0.0025	C	Background diabetic retinopathy	-0.0354	0.0328	C
rs815163	Body mass index	-0.0182	0.0024	C	Background diabetic retinopathy	0.0038	0.0329	C
rs815715	Body mass index	-0.0163	0.0024	G	Background diabetic retinopathy	0.0523	0.0329	G

rs845084	Body mass index	0.0203	0.0028	A	Background diabetic retinopathy	0.0956	0.0373	A
rs862320	Body mass index	-0.0228	0.0024	T	Background diabetic retinopathy	-0.0045	0.0330	T
rs869400	Body mass index	0.0307	0.0031	G	Background diabetic retinopathy	-0.0108	0.0444	G
rs879620	Body mass index	0.0256	0.0025	T	Background diabetic retinopathy	0.0169	0.0333	T
rs9267671	Body mass index	0.0324	0.0050	A	Background diabetic retinopathy	-0.3494	0.1119	A
rs9291822	Body mass index	-0.0136	0.0024	T	Background diabetic retinopathy	-0.0093	0.0336	T
rs9320823	Body mass index	0.0189	0.0025	C	Background diabetic retinopathy	0.0248	0.0350	C
rs9342196	Body mass index	0.0167	0.0031	T	Background diabetic retinopathy	0.0202	0.0465	T
rs935166	Body mass index	-0.0155	0.0024	A	Background diabetic retinopathy	0.0222	0.0333	A
rs9402104	Body mass index	0.0139	0.0025	A	Background diabetic retinopathy	0.0115	0.0338	A
rs946185	Body mass index	-0.0147	0.0025	G	Background diabetic retinopathy	-0.0279	0.0336	G
rs9489620	Body mass index	0.0148	0.0024	C	Background diabetic retinopathy	0.0528	0.0333	C
rs9515455	Body mass index	0.0180	0.0024	A	Background diabetic retinopathy	-0.0108	0.0329	A
rs9527906	Body mass index	-0.0162	0.0028	A	Background diabetic retinopathy	0.0238	0.0382	A
rs9536449	Body mass index	-0.0136	0.0024	G	Background diabetic retinopathy	-0.0264	0.0330	G
rs9641499	Body mass index	-0.0171	0.0024	A	Background diabetic retinopathy	0.0007	0.0335	A
rs9688977	Body mass index	0.0240	0.0034	C	Background diabetic retinopathy	0.0608	0.0428	C
rs9843653	Body mass index	0.0317	0.0024	C	Background diabetic retinopathy	0.0251	0.0335	C
rs9847186	Body mass index	-0.0143	0.0024	A	Background diabetic retinopathy	0.0169	0.0334	A
rs10100245	Body mass index	0.0206	0.0024	A	Diabetic retinopathy	0.0256	0.0132	A
rs10187101	Body mass index	-0.0157	0.0025	T	Diabetic retinopathy	-0.0072	0.0133	T
rs10404726	Body mass index	-0.0199	0.0024	T	Diabetic retinopathy	0.0018	0.0132	T
rs10465231	Body mass index	0.0171	0.0024	T	Diabetic retinopathy	0.0095	0.0132	T
rs1064213	Body mass index	0.0162	0.0024	A	Diabetic retinopathy	0.0016	0.0134	A
rs10788493	Body mass index	0.0137	0.0024	T	Diabetic retinopathy	0.0185	0.0133	T
rs10803762	Body mass index	0.0155	0.0026	A	Diabetic retinopathy	-0.0132	0.0144	A
rs10805383	Body mass index	0.0169	0.0024	A	Diabetic retinopathy	0.0104	0.0134	A
rs10865612	Body mass index	-0.0231	0.0025	C	Diabetic retinopathy	0.0082	0.0133	C
rs10898330	Body mass index	-0.0143	0.0024	T	Diabetic retinopathy	0.0138	0.0133	T
rs10938397	Body mass index	0.0290	0.0024	G	Diabetic retinopathy	0.0549	0.0132	G
rs10995427	Body mass index	-0.0165	0.0025	A	Diabetic retinopathy	-0.0055	0.0136	A

rs11012732	Body mass index	0.0238	0.0026	G	Diabetic retinopathy	0.0292	0.0141	G
rs11078883	Body mass index	0.0157	0.0025	G	Diabetic retinopathy	0.0300	0.0141	G
rs11084554	Body mass index	-0.0216	0.0033	A	Diabetic retinopathy	-0.0373	0.0195	A
rs11099020	Body mass index	-0.0153	0.0025	T	Diabetic retinopathy	-0.0084	0.0135	T
rs11150745	Body mass index	-0.0210	0.0026	G	Diabetic retinopathy	0.0011	0.0146	G
rs111640872	Body mass index	0.0206	0.0026	C	Diabetic retinopathy	-0.0082	0.0138	C
rs11223641	Body mass index	-0.0191	0.0034	C	Diabetic retinopathy	-0.0335	0.0267	C
rs112520079	Body mass index	0.0205	0.0030	G	Diabetic retinopathy	-0.0158	0.0194	G
rs11264489	Body mass index	0.0141	0.0025	G	Diabetic retinopathy	0.0127	0.0147	G
rs112693590	Body mass index	-0.0329	0.0057	A	Diabetic retinopathy	-0.0081	0.0229	A
rs1127100	Body mass index	0.0165	0.0025	C	Diabetic retinopathy	-0.0010	0.0138	C
rs113182412	Body mass index	-0.0194	0.0033	A	Diabetic retinopathy	0.0298	0.0219	A
rs113230003	Body mass index	-0.0198	0.0028	A	Diabetic retinopathy	0.0061	0.0161	A
rs113603865	Body mass index	0.0197	0.0030	T	Diabetic retinopathy	0.0352	0.0167	T
rs11515071	Body mass index	-0.0228	0.0025	T	Diabetic retinopathy	0.0077	0.0132	T
rs11642015	Body mass index	0.0724	0.0024	T	Diabetic retinopathy	0.0545	0.0133	T
rs11650012	Body mass index	0.0186	0.0032	A	Diabetic retinopathy	0.0043	0.0183	A
rs11655587	Body mass index	-0.0196	0.0025	T	Diabetic retinopathy	-0.0107	0.0138	T
rs11666480	Body mass index	0.0185	0.0024	G	Diabetic retinopathy	0.0025	0.0133	G
rs11742930	Body mass index	0.0143	0.0024	T	Diabetic retinopathy	-0.0073	0.0131	T
rs11757278	Body mass index	-0.0158	0.0026	C	Diabetic retinopathy	0.0027	0.0135	C
rs11761411	Body mass index	-0.0185	0.0033	T	Diabetic retinopathy	-0.0087	0.0174	T
rs117632017	Body mass index	0.0364	0.0064	A	Diabetic retinopathy	-0.1395	0.0616	A
rs11782074	Body mass index	0.0145	0.0025	T	Diabetic retinopathy	0.0074	0.0137	T
rs11856579	Body mass index	-0.0198	0.0027	A	Diabetic retinopathy	0.0071	0.0161	A
rs12024554	Body mass index	-0.0163	0.0028	T	Diabetic retinopathy	-0.0162	0.0158	T
rs12042959	Body mass index	-0.0206	0.0034	G	Diabetic retinopathy	-0.0112	0.0180	G
rs12049202	Body mass index	0.0218	0.0030	T	Diabetic retinopathy	0.0297	0.0146	T
rs12140153	Body mass index	-0.0318	0.0042	T	Diabetic retinopathy	-0.0668	0.0246	T
rs12144626	Body mass index	-0.0172	0.0024	C	Diabetic retinopathy	-0.0140	0.0135	C
rs12477385	Body mass index	-0.0173	0.0029	T	Diabetic retinopathy	-0.0121	0.0151	T

rs12479357	Body mass index	0.0179	0.0025	G	Diabetic retinopathy	0.0034	0.0137	G
rs12614861	Body mass index	0.0152	0.0025	T	Diabetic retinopathy	-0.0160	0.0140	T
rs12622280	Body mass index	-0.0187	0.0033	G	Diabetic retinopathy	-0.0330	0.0203	G
rs12662900	Body mass index	-0.0169	0.0027	A	Diabetic retinopathy	-0.0133	0.0141	A
rs12679106	Body mass index	-0.0220	0.0027	T	Diabetic retinopathy	-0.0065	0.0148	T
rs1286138	Body mass index	0.0181	0.0026	G	Diabetic retinopathy	-0.0044	0.0147	G
rs12877270	Body mass index	0.0169	0.0024	A	Diabetic retinopathy	0.0043	0.0132	A
rs12881629	Body mass index	0.0240	0.0044	G	Diabetic retinopathy	0.0041	0.0250	G
rs12885458	Body mass index	-0.0156	0.0024	G	Diabetic retinopathy	-0.0315	0.0134	G
rs1296328	Body mass index	-0.0190	0.0024	C	Diabetic retinopathy	0.0194	0.0132	C
rs12977259	Body mass index	0.0186	0.0032	G	Diabetic retinopathy	-0.0353	0.0196	G
rs12992672	Body mass index	0.0508	0.0032	A	Diabetic retinopathy	0.0152	0.0178	A
rs13047416	Body mass index	-0.0136	0.0025	G	Diabetic retinopathy	0.0099	0.0132	G
rs13062093	Body mass index	0.0171	0.0025	G	Diabetic retinopathy	0.0176	0.0139	G
rs13076052	Body mass index	0.0161	0.0027	G	Diabetic retinopathy	0.0070	0.0138	G
rs13135092	Body mass index	0.0501	0.0044	G	Diabetic retinopathy	0.0473	0.0504	G
rs13174863	Body mass index	0.0250	0.0034	G	Diabetic retinopathy	-0.0045	0.0185	G
rs1320903	Body mass index	0.0220	0.0026	A	Diabetic retinopathy	0.0240	0.0144	A
rs1327259	Body mass index	-0.0140	0.0025	G	Diabetic retinopathy	-0.0141	0.0132	G
rs1342391	Body mass index	0.0159	0.0026	T	Diabetic retinopathy	0.0058	0.0142	T
rs13427822	Body mass index	-0.0199	0.0027	G	Diabetic retinopathy	-0.0081	0.0144	G
rs1411432	Body mass index	0.0249	0.0031	C	Diabetic retinopathy	0.0302	0.0173	C
rs1412239	Body mass index	0.0239	0.0026	G	Diabetic retinopathy	0.0099	0.0136	G
rs1441264	Body mass index	0.0206	0.0025	A	Diabetic retinopathy	-0.0169	0.0140	A
rs1446585	Body mass index	-0.0171	0.0029	G	Diabetic retinopathy	-0.0049	0.0138	G
rs1454687	Body mass index	-0.0225	0.0024	G	Diabetic retinopathy	-0.0011	0.0133	G
rs1458156	Body mass index	0.0138	0.0024	T	Diabetic retinopathy	0.0116	0.0132	T
rs1477290	Body mass index	0.0335	0.0035	C	Diabetic retinopathy	-0.0201	0.0202	C
rs147730268	Body mass index	-0.0361	0.0043	T	Diabetic retinopathy	0.0061	0.0238	T
rs1582931	Body mass index	-0.0149	0.0024	A	Diabetic retinopathy	-0.0010	0.0131	A
rs16846140	Body mass index	0.0162	0.0025	G	Diabetic retinopathy	-0.0226	0.0140	G

rs16916303	Body mass index	-0.0207	0.0037	G	Diabetic retinopathy	-0.0162	0.0197	G
rs16975459	Body mass index	0.0240	0.0037	C	Diabetic retinopathy	-0.0149	0.0172	C
rs17014332	Body mass index	0.0180	0.0029	C	Diabetic retinopathy	0.0019	0.0167	C
rs17024393	Body mass index	0.0675	0.0076	C	Diabetic retinopathy	-0.0050	0.0288	C
rs17058884	Body mass index	-0.0318	0.0058	G	Diabetic retinopathy	-0.0394	0.0287	G
rs17085463	Body mass index	-0.0148	0.0026	A	Diabetic retinopathy	0.0209	0.0140	A
rs17149254	Body mass index	-0.0238	0.0031	C	Diabetic retinopathy	-0.0105	0.0162	C
rs17342242	Body mass index	-0.0162	0.0029	G	Diabetic retinopathy	0.0221	0.0177	G
rs17399739	Body mass index	0.0262	0.0047	G	Diabetic retinopathy	0.0244	0.0233	G
rs17716502	Body mass index	-0.0223	0.0030	T	Diabetic retinopathy	0.0002	0.0167	T
rs1788808	Body mass index	-0.0183	0.0024	G	Diabetic retinopathy	-0.0001	0.0132	G
rs1805123	Body mass index	-0.0180	0.0028	G	Diabetic retinopathy	-0.0145	0.0155	G
rs1840661	Body mass index	0.0137	0.0024	A	Diabetic retinopathy	-0.0005	0.0133	A
rs1884897	Body mass index	0.0213	0.0025	G	Diabetic retinopathy	-0.0219	0.0140	G
rs1901241	Body mass index	0.0193	0.0033	G	Diabetic retinopathy	0.0025	0.0167	G
rs1919243	Body mass index	0.0139	0.0024	C	Diabetic retinopathy	0.0156	0.0134	C
rs1941706	Body mass index	0.0139	0.0024	G	Diabetic retinopathy	0.0049	0.0132	G
rs1949204	Body mass index	0.0167	0.0028	G	Diabetic retinopathy	-0.0019	0.0148	G
rs2035806	Body mass index	-0.0166	0.0024	A	Diabetic retinopathy	0.0180	0.0131	A
rs2046002	Body mass index	-0.0146	0.0025	C	Diabetic retinopathy	-0.0135	0.0132	C
rs2121058	Body mass index	-0.0238	0.0029	C	Diabetic retinopathy	-0.0268	0.0155	C
rs2135745	Body mass index	-0.0168	0.0028	G	Diabetic retinopathy	0.0022	0.0159	G
rs2155869	Body mass index	-0.0184	0.0031	C	Diabetic retinopathy	0.0203	0.0162	C
rs215634	Body mass index	-0.0148	0.0025	G	Diabetic retinopathy	-0.0363	0.0146	G
rs2192649	Body mass index	0.0137	0.0024	G	Diabetic retinopathy	-0.0088	0.0131	G
rs2234458	Body mass index	-0.0206	0.0025	T	Diabetic retinopathy	0.0032	0.0143	T
rs2253310	Body mass index	0.0173	0.0025	G	Diabetic retinopathy	0.0113	0.0133	G
rs2292238	Body mass index	-0.0177	0.0024	C	Diabetic retinopathy	0.0229	0.0134	C
rs2307111	Body mass index	-0.0290	0.0025	C	Diabetic retinopathy	-0.0301	0.0133	C
rs2318543	Body mass index	-0.0194	0.0029	G	Diabetic retinopathy	-0.0297	0.0158	G
rs2384054	Body mass index	0.0350	0.0024	C	Diabetic retinopathy	0.0223	0.0133	C

rs2398861	Body mass index	0.0221	0.0028	G	Diabetic retinopathy	0.0029	0.0141	G
rs241460	Body mass index	-0.0205	0.0026	G	Diabetic retinopathy	-0.0014	0.0136	G
rs2425857	Body mass index	-0.0136	0.0024	G	Diabetic retinopathy	-0.0252	0.0137	G
rs2439823	Body mass index	0.0216	0.0024	G	Diabetic retinopathy	0.0107	0.0132	G
rs2450254	Body mass index	-0.0141	0.0024	T	Diabetic retinopathy	-0.0084	0.0138	T
rs2450445	Body mass index	-0.0154	0.0026	A	Diabetic retinopathy	0.0051	0.0142	A
rs245775	Body mass index	0.0203	0.0027	G	Diabetic retinopathy	-0.0001	0.0140	G
rs2470392	Body mass index	0.0145	0.0027	C	Diabetic retinopathy	0.0140	0.0136	C
rs2474898	Body mass index	0.0146	0.0025	T	Diabetic retinopathy	0.0087	0.0133	T
rs2482704	Body mass index	-0.0138	0.0024	T	Diabetic retinopathy	0.0021	0.0136	T
rs2606228	Body mass index	-0.0156	0.0025	C	Diabetic retinopathy	-0.0211	0.0134	C
rs2616192	Body mass index	0.0141	0.0026	T	Diabetic retinopathy	0.0248	0.0135	T
rs2678204	Body mass index	0.0281	0.0025	G	Diabetic retinopathy	0.0062	0.0144	G
rs2711111	Body mass index	-0.0147	0.0025	G	Diabetic retinopathy	0.0123	0.0134	G
rs2725371	Body mass index	-0.0181	0.0026	G	Diabetic retinopathy	-0.0247	0.0150	G
rs273505	Body mass index	0.0189	0.0024	C	Diabetic retinopathy	-0.0050	0.0131	C
rs28366156	Body mass index	-0.0290	0.0036	C	Diabetic retinopathy	-0.1488	0.0257	C
rs28447555	Body mass index	0.0185	0.0031	T	Diabetic retinopathy	0.0191	0.0188	T
rs28489620	Body mass index	-0.0147	0.0027	A	Diabetic retinopathy	0.0108	0.0144	A
rs2861685	Body mass index	-0.0176	0.0024	C	Diabetic retinopathy	-0.0017	0.0133	C
rs286818	Body mass index	-0.0291	0.0032	A	Diabetic retinopathy	0.0233	0.0161	A
rs2962082	Body mass index	-0.0135	0.0024	A	Diabetic retinopathy	-0.0142	0.0132	A
rs2975693	Body mass index	0.0238	0.0039	C	Diabetic retinopathy	-0.0003	0.0256	C
rs34045288	Body mass index	0.0260	0.0025	T	Diabetic retinopathy	0.0156	0.0136	T
rs34095326	Body mass index	-0.0244	0.0038	A	Diabetic retinopathy	-0.0265	0.0180	A
rs34236292	Body mass index	-0.0141	0.0026	T	Diabetic retinopathy	-0.0086	0.0152	T
rs34361149	Body mass index	-0.0310	0.0030	A	Diabetic retinopathy	-0.0041	0.0190	A
rs34373881	Body mass index	-0.0169	0.0027	A	Diabetic retinopathy	-0.0223	0.0149	A
rs34774377	Body mass index	-0.0208	0.0037	C	Diabetic retinopathy	0.0039	0.0213	C
rs34811474	Body mass index	-0.0306	0.0028	A	Diabetic retinopathy	-0.0254	0.0157	A
rs34966008	Body mass index	-0.0199	0.0024	T	Diabetic retinopathy	-0.0186	0.0136	T

rs35025195	Body mass index	-0.0230	0.0032	A	Diabetic retinopathy	-0.0343	0.0190	A
rs35193668	Body mass index	-0.0167	0.0025	T	Diabetic retinopathy	-0.0245	0.0133	T
rs35483388	Body mass index	0.0141	0.0025	T	Diabetic retinopathy	0.0072	0.0133	T
rs35626515	Body mass index	0.0266	0.0024	A	Diabetic retinopathy	-0.0264	0.0132	A
rs35722922	Body mass index	-0.0165	0.0025	G	Diabetic retinopathy	-0.0054	0.0140	G
rs357501	Body mass index	0.0154	0.0025	A	Diabetic retinopathy	-0.0038	0.0134	A
rs35851183	Body mass index	0.0168	0.0025	G	Diabetic retinopathy	0.0032	0.0143	G
rs35882248	Body mass index	0.0192	0.0026	T	Diabetic retinopathy	-0.0083	0.0142	T
rs36007635	Body mass index	-0.0200	0.0035	A	Diabetic retinopathy	-0.0173	0.0228	A
rs362307	Body mass index	0.0314	0.0046	T	Diabetic retinopathy	0.0215	0.0262	T
rs3759584	Body mass index	-0.0166	0.0025	C	Diabetic retinopathy	-0.0263	0.0142	C
rs3802858	Body mass index	-0.0165	0.0024	C	Diabetic retinopathy	-0.0162	0.0133	C
rs3803286	Body mass index	-0.0208	0.0025	G	Diabetic retinopathy	-0.0173	0.0139	G
rs3810291	Body mass index	0.0297	0.0026	A	Diabetic retinopathy	0.0311	0.0136	A
rs3843540	Body mass index	-0.0242	0.0034	C	Diabetic retinopathy	0.0531	0.0157	C
rs3844598	Body mass index	0.0137	0.0024	G	Diabetic retinopathy	0.0159	0.0133	G
rs3861879	Body mass index	0.0137	0.0024	G	Diabetic retinopathy	0.0010	0.0133	G
rs3897102	Body mass index	0.0147	0.0025	T	Diabetic retinopathy	-0.0012	0.0133	T
rs390192	Body mass index	-0.0154	0.0024	G	Diabetic retinopathy	-0.0274	0.0132	G
rs39330	Body mass index	-0.0189	0.0024	T	Diabetic retinopathy	0.0071	0.0132	T
rs4246657	Body mass index	0.0170	0.0025	T	Diabetic retinopathy	0.0251	0.0141	T
rs4261944	Body mass index	0.0155	0.0025	G	Diabetic retinopathy	0.0282	0.0143	G
rs4402589	Body mass index	0.0292	0.0024	G	Diabetic retinopathy	-0.0004	0.0131	G
rs4467770	Body mass index	0.0157	0.0027	A	Diabetic retinopathy	0.0239	0.0146	A
rs4474229	Body mass index	-0.0159	0.0025	A	Diabetic retinopathy	-0.0102	0.0139	A
rs4482463	Body mass index	-0.0341	0.0045	A	Diabetic retinopathy	-0.0428	0.0279	A
rs4502882	Body mass index	-0.0147	0.0025	T	Diabetic retinopathy	-0.0031	0.0144	T
rs4595495	Body mass index	0.0137	0.0024	G	Diabetic retinopathy	0.0077	0.0132	G
rs4648450	Body mass index	-0.0154	0.0024	A	Diabetic retinopathy	-0.0002	0.0133	A
rs4687770	Body mass index	-0.0193	0.0035	C	Diabetic retinopathy	0.0069	0.0181	C
rs4718964	Body mass index	0.0146	0.0024	T	Diabetic retinopathy	-0.0194	0.0134	T

rs4757144	Body mass index	0.0165	0.0024	A	Diabetic retinopathy	0.0078	0.0132	A
rs4776970	Body mass index	-0.0255	0.0025	T	Diabetic retinopathy	-0.0096	0.0138	T
rs4777541	Body mass index	0.0197	0.0028	T	Diabetic retinopathy	0.0117	0.0189	T
rs4911382	Body mass index	0.0156	0.0024	T	Diabetic retinopathy	-0.0001	0.0138	T
rs491711	Body mass index	-0.0158	0.0026	C	Diabetic retinopathy	-0.0030	0.0156	C
rs4921301	Body mass index	-0.0182	0.0030	T	Diabetic retinopathy	0.0032	0.0161	T
rs4929923	Body mass index	0.0174	0.0025	C	Diabetic retinopathy	0.0157	0.0137	C
rs525101	Body mass index	0.0165	0.0025	C	Diabetic retinopathy	0.0081	0.0133	C
rs539515	Body mass index	0.0475	0.0030	C	Diabetic retinopathy	0.0024	0.0172	C
rs550974	Body mass index	0.0159	0.0024	T	Diabetic retinopathy	0.0175	0.0144	T
rs55689274	Body mass index	-0.0148	0.0027	A	Diabetic retinopathy	-0.0005	0.0148	A
rs55726687	Body mass index	0.0239	0.0029	A	Diabetic retinopathy	0.0070	0.0160	A
rs55886426	Body mass index	-0.0316	0.0055	G	Diabetic retinopathy	-0.0515	0.0332	G
rs55938344	Body mass index	-0.0168	0.0028	C	Diabetic retinopathy	0.0032	0.0151	C
rs56067609	Body mass index	-0.0194	0.0035	T	Diabetic retinopathy	-0.0072	0.0190	T
rs56161855	Body mass index	0.0230	0.0035	T	Diabetic retinopathy	-0.0529	0.0183	T
rs56212061	Body mass index	-0.0211	0.0034	T	Diabetic retinopathy	-0.0171	0.0223	T
rs56773984	Body mass index	-0.0179	0.0032	T	Diabetic retinopathy	0.0325	0.0294	T
rs56803094	Body mass index	-0.0189	0.0029	G	Diabetic retinopathy	-0.0114	0.0147	G
rs57636386	Body mass index	-0.0428	0.0044	C	Diabetic retinopathy	0.0378	0.0303	C
rs58862095	Body mass index	-0.0244	0.0024	T	Diabetic retinopathy	0.0021	0.0132	T
rs588660	Body mass index	0.0183	0.0024	A	Diabetic retinopathy	0.0041	0.0134	A
rs59104534	Body mass index	0.0150	0.0026	T	Diabetic retinopathy	0.0020	0.0140	T
rs5995843	Body mass index	-0.0174	0.0025	G	Diabetic retinopathy	0.0021	0.0136	G
rs6050446	Body mass index	0.0421	0.0068	G	Diabetic retinopathy	-0.0286	0.0441	G
rs60654199	Body mass index	0.0313	0.0048	A	Diabetic retinopathy	-0.0090	0.0306	A
rs60764613	Body mass index	0.0236	0.0034	T	Diabetic retinopathy	0.0338	0.0173	T
rs61813324	Body mass index	0.0279	0.0036	T	Diabetic retinopathy	-0.0052	0.0182	T
rs61826867	Body mass index	0.0251	0.0038	G	Diabetic retinopathy	0.0248	0.0239	G
rs61871615	Body mass index	-0.0280	0.0044	T	Diabetic retinopathy	-0.0164	0.0243	T
rs61903695	Body mass index	0.0164	0.0028	G	Diabetic retinopathy	0.0210	0.0153	G

rs61969510	Body mass index	0.0155	0.0027	C	Diabetic retinopathy	0.0218	0.0136	C
rs62106258	Body mass index	-0.0907	0.0056	C	Diabetic retinopathy	-0.0302	0.0465	C
rs62147189	Body mass index	-0.0170	0.0025	G	Diabetic retinopathy	0.0008	0.0134	G
rs62246314	Body mass index	0.0228	0.0040	A	Diabetic retinopathy	-0.0088	0.0270	A
rs62407562	Body mass index	0.0171	0.0027	A	Diabetic retinopathy	0.0012	0.0170	A
rs62543438	Body mass index	-0.0148	0.0027	C	Diabetic retinopathy	-0.0148	0.0162	C
rs6265	Body mass index	-0.0402	0.0031	T	Diabetic retinopathy	0.0025	0.0181	T
rs6536575	Body mass index	0.0141	0.0024	C	Diabetic retinopathy	-0.0013	0.0133	C
rs6575340	Body mass index	0.0229	0.0025	A	Diabetic retinopathy	0.0100	0.0136	A
rs6601527	Body mass index	-0.0217	0.0024	A	Diabetic retinopathy	-0.0145	0.0189	A
rs66679256	Body mass index	0.0163	0.0024	T	Diabetic retinopathy	-0.0202	0.0136	T
rs6687953	Body mass index	0.0159	0.0025	G	Diabetic retinopathy	0.0048	0.0132	G
rs66922415	Body mass index	0.0521	0.0028	G	Diabetic retinopathy	0.0293	0.0170	G
rs6705567	Body mass index	-0.0136	0.0025	C	Diabetic retinopathy	0.0040	0.0135	C
rs6722241	Body mass index	-0.0202	0.0027	C	Diabetic retinopathy	-0.0292	0.0168	C
rs6739755	Body mass index	-0.0210	0.0025	G	Diabetic retinopathy	-0.0375	0.0134	G
rs67609008	Body mass index	0.0146	0.0027	C	Diabetic retinopathy	-0.0185	0.0171	C
rs6780459	Body mass index	0.0164	0.0028	T	Diabetic retinopathy	0.0074	0.0161	T
rs67844506	Body mass index	-0.0259	0.0031	G	Diabetic retinopathy	0.0064	0.0180	G
rs6789488	Body mass index	0.0200	0.0028	C	Diabetic retinopathy	-0.0200	0.0183	C
rs6809307	Body mass index	0.0157	0.0028	T	Diabetic retinopathy	-0.0080	0.0172	T
rs6831020	Body mass index	-0.0159	0.0026	A	Diabetic retinopathy	-0.0033	0.0155	A
rs6861649	Body mass index	0.0143	0.0025	C	Diabetic retinopathy	0.0073	0.0136	C
rs6950388	Body mass index	0.0172	0.0030	A	Diabetic retinopathy	0.0288	0.0157	A
rs7006178	Body mass index	0.0155	0.0027	C	Diabetic retinopathy	0.0096	0.0152	C
rs7030732	Body mass index	-0.0152	0.0025	A	Diabetic retinopathy	-0.0072	0.0135	A
rs704061	Body mass index	0.0159	0.0024	C	Diabetic retinopathy	0.0005	0.0135	C
rs7094644	Body mass index	0.0153	0.0026	A	Diabetic retinopathy	-0.0301	0.0143	A
rs7116641	Body mass index	0.0244	0.0026	G	Diabetic retinopathy	0.0146	0.0142	G
rs7124681	Body mass index	0.0267	0.0024	A	Diabetic retinopathy	0.0090	0.0138	A
rs7132908	Body mass index	0.0285	0.0025	A	Diabetic retinopathy	0.0084	0.0136	A

rs7138383	Body mass index	-0.0214	0.0028	A	Diabetic retinopathy	-0.0091	0.0142	A
rs7141420	Body mass index	0.0208	0.0024	T	Diabetic retinopathy	0.0171	0.0134	T
rs71495049	Body mass index	0.0265	0.0043	A	Diabetic retinopathy	0.0180	0.0208	A
rs7163362	Body mass index	0.0132	0.0024	A	Diabetic retinopathy	0.0198	0.0136	A
rs7183417	Body mass index	0.0149	0.0024	T	Diabetic retinopathy	0.0398	0.0140	T
rs7189149	Body mass index	0.0213	0.0036	G	Diabetic retinopathy	-0.0474	0.0203	G
rs7195386	Body mass index	-0.0159	0.0024	C	Diabetic retinopathy	-0.0002	0.0131	C
rs7201895	Body mass index	-0.0173	0.0025	A	Diabetic retinopathy	-0.0088	0.0144	A
rs7218014	Body mass index	0.0207	0.0030	C	Diabetic retinopathy	0.0156	0.0154	C
rs72697614	Body mass index	0.0150	0.0026	A	Diabetic retinopathy	-0.0068	0.0145	A
rs72820274	Body mass index	0.0160	0.0024	A	Diabetic retinopathy	-0.0108	0.0132	A
rs72892910	Body mass index	0.0402	0.0032	T	Diabetic retinopathy	0.0178	0.0163	T
rs72976986	Body mass index	-0.0240	0.0031	A	Diabetic retinopathy	-0.0423	0.0181	A
rs73050254	Body mass index	0.0193	0.0035	A	Diabetic retinopathy	-0.0247	0.0259	A
rs73144053	Body mass index	-0.0154	0.0026	A	Diabetic retinopathy	-0.0007	0.0142	A
rs73169730	Body mass index	0.0195	0.0027	G	Diabetic retinopathy	-0.0252	0.0151	G
rs7321331	Body mass index	0.0176	0.0028	A	Diabetic retinopathy	0.0049	0.0140	A
rs73213484	Body mass index	-0.0212	0.0035	T	Diabetic retinopathy	-0.0055	0.0185	T
rs7331420	Body mass index	-0.0147	0.0027	A	Diabetic retinopathy	-0.0037	0.0145	A
rs7442885	Body mass index	-0.0254	0.0030	G	Diabetic retinopathy	0.0072	0.0168	G
rs7498044	Body mass index	-0.0176	0.0029	A	Diabetic retinopathy	0.0018	0.0151	A
rs750090	Body mass index	-0.0156	0.0025	C	Diabetic retinopathy	-0.0024	0.0134	C
rs752179	Body mass index	-0.0144	0.0026	A	Diabetic retinopathy	-0.0092	0.0138	A
rs75499503	Body mass index	-0.0198	0.0029	T	Diabetic retinopathy	0.0104	0.0157	T
rs7553158	Body mass index	-0.0174	0.0024	A	Diabetic retinopathy	-0.0081	0.0131	A
rs75557510	Body mass index	-0.0378	0.0051	G	Diabetic retinopathy	-0.0463	0.0307	G
rs756717	Body mass index	-0.0147	0.0025	A	Diabetic retinopathy	0.0069	0.0134	A
rs7568228	Body mass index	-0.0140	0.0024	C	Diabetic retinopathy	-0.0019	0.0132	C
rs76040172	Body mass index	-0.0408	0.0053	A	Diabetic retinopathy	-0.0606	0.0239	A
rs7701777	Body mass index	-0.0170	0.0027	G	Diabetic retinopathy	0.0082	0.0153	G
rs7719067	Body mass index	-0.0162	0.0024	G	Diabetic retinopathy	-0.0144	0.0134	G

rs7723426	Body mass index	0.0147	0.0026	C	Diabetic retinopathy	0.0086	0.0132	C
rs7755574	Body mass index	0.0155	0.0027	T	Diabetic retinopathy	0.0124	0.0140	T
rs7774	Body mass index	0.0166	0.0026	A	Diabetic retinopathy	0.0020	0.0135	A
rs778094	Body mass index	-0.0149	0.0024	A	Diabetic retinopathy	0.0008	0.0132	A
rs7852189	Body mass index	0.0166	0.0026	G	Diabetic retinopathy	0.0044	0.0135	G
rs78565420	Body mass index	0.0337	0.0055	T	Diabetic retinopathy	-0.0071	0.0338	T
rs79113395	Body mass index	-0.0209	0.0027	A	Diabetic retinopathy	0.0007	0.0160	A
rs7933085	Body mass index	0.0157	0.0024	G	Diabetic retinopathy	-0.0058	0.0132	G
rs7941828	Body mass index	-0.0156	0.0025	T	Diabetic retinopathy	-0.0142	0.0142	T
rs7952102	Body mass index	-0.0152	0.0025	C	Diabetic retinopathy	-0.0004	0.0138	C
rs7992832	Body mass index	-0.0176	0.0027	T	Diabetic retinopathy	-0.0268	0.0151	T
rs799449	Body mass index	0.0199	0.0024	T	Diabetic retinopathy	-0.0097	0.0133	T
rs8015400	Body mass index	0.0211	0.0026	A	Diabetic retinopathy	0.0103	0.0137	A
rs80330591	Body mass index	-0.0214	0.0034	A	Diabetic retinopathy	-0.0033	0.0171	A
rs8078135	Body mass index	-0.0195	0.0024	T	Diabetic retinopathy	-0.0147	0.0132	T
rs8134638	Body mass index	0.0152	0.0025	C	Diabetic retinopathy	0.0097	0.0132	C
rs815163	Body mass index	-0.0182	0.0024	C	Diabetic retinopathy	-0.0005	0.0132	C
rs815715	Body mass index	-0.0163	0.0024	G	Diabetic retinopathy	-0.0249	0.0132	G
rs845084	Body mass index	0.0203	0.0028	A	Diabetic retinopathy	0.0152	0.0150	A
rs862320	Body mass index	-0.0228	0.0024	T	Diabetic retinopathy	0.0049	0.0133	T
rs869400	Body mass index	0.0307	0.0031	G	Diabetic retinopathy	0.0322	0.0179	G
rs879620	Body mass index	0.0256	0.0025	T	Diabetic retinopathy	0.0075	0.0134	T
rs9267671	Body mass index	0.0324	0.0050	A	Diabetic retinopathy	-0.0661	0.0452	A
rs9291822	Body mass index	-0.0136	0.0024	T	Diabetic retinopathy	-0.0110	0.0135	T
rs9320823	Body mass index	0.0189	0.0025	C	Diabetic retinopathy	0.0161	0.0140	C
rs9342196	Body mass index	0.0167	0.0031	T	Diabetic retinopathy	-0.0135	0.0187	T
rs935166	Body mass index	-0.0155	0.0024	A	Diabetic retinopathy	-0.0138	0.0133	A
rs9402104	Body mass index	0.0139	0.0025	A	Diabetic retinopathy	0.0014	0.0136	A
rs946185	Body mass index	-0.0147	0.0025	G	Diabetic retinopathy	-0.0131	0.0135	G
rs9489620	Body mass index	0.0148	0.0024	C	Diabetic retinopathy	0.0252	0.0134	C
rs9515455	Body mass index	0.0180	0.0024	A	Diabetic retinopathy	-0.0004	0.0132	A

rs9527906	Body mass index	-0.0162	0.0028	A	Diabetic retinopathy	0.0096	0.0153	A
rs9536449	Body mass index	-0.0136	0.0024	G	Diabetic retinopathy	-0.0083	0.0132	G
rs9641499	Body mass index	-0.0171	0.0024	A	Diabetic retinopathy	0.0102	0.0134	A
rs9688977	Body mass index	0.0240	0.0034	C	Diabetic retinopathy	0.0271	0.0172	C
rs9843653	Body mass index	0.0317	0.0024	C	Diabetic retinopathy	-0.0025	0.0134	C
rs9847186	Body mass index	-0.0143	0.0024	A	Diabetic retinopathy	-0.0169	0.0134	A
rs10100245	Body mass index	0.0206	0.0024	A	Proliferative diabetic retinopathy	0.0318	0.0164	A
rs10187101	Body mass index	-0.0157	0.0025	T	Proliferative diabetic retinopathy	0.0038	0.0166	T
rs10404726	Body mass index	-0.0199	0.0024	T	Proliferative diabetic retinopathy	0.0133	0.0164	T
rs10465231	Body mass index	0.0171	0.0024	T	Proliferative diabetic retinopathy	0.0161	0.0164	T
rs1064213	Body mass index	0.0162	0.0024	A	Proliferative diabetic retinopathy	0.0094	0.0168	A
rs10788493	Body mass index	0.0137	0.0024	T	Proliferative diabetic retinopathy	0.0315	0.0166	T
rs10803762	Body mass index	0.0155	0.0026	A	Proliferative diabetic retinopathy	-0.0348	0.0180	A
rs10805383	Body mass index	0.0169	0.0024	A	Proliferative diabetic retinopathy	0.0145	0.0167	A
rs10865612	Body mass index	-0.0231	0.0025	C	Proliferative diabetic retinopathy	0.0224	0.0166	C
rs10898330	Body mass index	-0.0143	0.0024	T	Proliferative diabetic retinopathy	0.0127	0.0166	T
rs10938397	Body mass index	0.0290	0.0024	G	Proliferative diabetic retinopathy	0.0576	0.0164	G
rs10995427	Body mass index	-0.0165	0.0025	A	Proliferative diabetic retinopathy	-0.0165	0.0169	A
rs11012732	Body mass index	0.0238	0.0026	G	Proliferative diabetic retinopathy	0.0300	0.0176	G
rs11078883	Body mass index	0.0157	0.0025	G	Proliferative diabetic retinopathy	0.0302	0.0176	G
rs11084554	Body mass index	-0.0216	0.0033	A	Proliferative diabetic retinopathy	-0.0467	0.0243	A
rs11099020	Body mass index	-0.0153	0.0025	T	Proliferative diabetic retinopathy	-0.0148	0.0169	T
rs11150745	Body mass index	-0.0210	0.0026	G	Proliferative diabetic retinopathy	0.0135	0.0183	G
rs111640872	Body mass index	0.0206	0.0026	C	Proliferative diabetic retinopathy	-0.0217	0.0172	C
rs11223641	Body mass index	-0.0191	0.0034	C	Proliferative diabetic retinopathy	-0.0580	0.0334	C
rs112520079	Body mass index	0.0205	0.0030	G	Proliferative diabetic retinopathy	-0.0442	0.0241	G
rs11264489	Body mass index	0.0141	0.0025	G	Proliferative diabetic retinopathy	0.0078	0.0183	G
rs112693590	Body mass index	-0.0329	0.0057	A	Proliferative diabetic retinopathy	-0.0161	0.0286	A
rs1127100	Body mass index	0.0165	0.0025	C	Proliferative diabetic retinopathy	-0.0112	0.0173	C
rs113182412	Body mass index	-0.0194	0.0033	A	Proliferative diabetic retinopathy	0.0213	0.0274	A
rs113230003	Body mass index	-0.0198	0.0028	A	Proliferative diabetic retinopathy	0.0122	0.0201	A

rs113603865	Body mass index	0.0197	0.0030	T	Proliferative diabetic retinopathy	0.0501	0.0208	T
rs11515071	Body mass index	-0.0228	0.0025	T	Proliferative diabetic retinopathy	-0.0121	0.0165	T
rs11642015	Body mass index	0.0724	0.0024	T	Proliferative diabetic retinopathy	0.0437	0.0166	T
rs11650012	Body mass index	0.0186	0.0032	A	Proliferative diabetic retinopathy	-0.0256	0.0229	A
rs11655587	Body mass index	-0.0196	0.0025	T	Proliferative diabetic retinopathy	-0.0210	0.0172	T
rs11666480	Body mass index	0.0185	0.0024	G	Proliferative diabetic retinopathy	0.0054	0.0167	G
rs11742930	Body mass index	0.0143	0.0024	T	Proliferative diabetic retinopathy	-0.0054	0.0164	T
rs11757278	Body mass index	-0.0158	0.0026	C	Proliferative diabetic retinopathy	0.0207	0.0168	C
rs11761411	Body mass index	-0.0185	0.0033	T	Proliferative diabetic retinopathy	-0.0375	0.0217	T
rs117632017	Body mass index	0.0364	0.0064	A	Proliferative diabetic retinopathy	-0.1716	0.0766	A
rs11782074	Body mass index	0.0145	0.0025	T	Proliferative diabetic retinopathy	0.0076	0.0170	T
rs11856579	Body mass index	-0.0198	0.0027	A	Proliferative diabetic retinopathy	0.0050	0.0201	A
rs12024554	Body mass index	-0.0163	0.0028	T	Proliferative diabetic retinopathy	0.0154	0.0197	T
rs12042959	Body mass index	-0.0206	0.0034	G	Proliferative diabetic retinopathy	-0.0142	0.0224	G
rs12049202	Body mass index	0.0218	0.0030	T	Proliferative diabetic retinopathy	0.0537	0.0182	T
rs12140153	Body mass index	-0.0318	0.0042	T	Proliferative diabetic retinopathy	-0.0654	0.0308	T
rs12144626	Body mass index	-0.0172	0.0024	C	Proliferative diabetic retinopathy	-0.0160	0.0169	C
rs12477385	Body mass index	-0.0173	0.0029	T	Proliferative diabetic retinopathy	0.0110	0.0189	T
rs12479357	Body mass index	0.0179	0.0025	G	Proliferative diabetic retinopathy	0.0205	0.0171	G
rs12614861	Body mass index	0.0152	0.0025	T	Proliferative diabetic retinopathy	-0.0194	0.0175	T
rs12622280	Body mass index	-0.0187	0.0033	G	Proliferative diabetic retinopathy	-0.0302	0.0253	G
rs12662900	Body mass index	-0.0169	0.0027	A	Proliferative diabetic retinopathy	-0.0401	0.0177	A
rs12679106	Body mass index	-0.0220	0.0027	T	Proliferative diabetic retinopathy	0.0095	0.0185	T
rs1286138	Body mass index	0.0181	0.0026	G	Proliferative diabetic retinopathy	-0.0054	0.0183	G
rs12877270	Body mass index	0.0169	0.0024	A	Proliferative diabetic retinopathy	-0.0118	0.0165	A
rs12881629	Body mass index	0.0240	0.0044	G	Proliferative diabetic retinopathy	-0.0206	0.0312	G
rs12885458	Body mass index	-0.0156	0.0024	G	Proliferative diabetic retinopathy	-0.0443	0.0167	G
rs1296328	Body mass index	-0.0190	0.0024	C	Proliferative diabetic retinopathy	0.0110	0.0164	C
rs12977259	Body mass index	0.0186	0.0032	G	Proliferative diabetic retinopathy	-0.0145	0.0245	G
rs12992672	Body mass index	0.0508	0.0032	A	Proliferative diabetic retinopathy	0.0315	0.0222	A
rs13047416	Body mass index	-0.0136	0.0025	G	Proliferative diabetic retinopathy	-0.0028	0.0164	G

rs13062093	Body mass index	0.0171	0.0025	G	Proliferative diabetic retinopathy	0.0230	0.0173	G
rs13076052	Body mass index	0.0161	0.0027	G	Proliferative diabetic retinopathy	0.0048	0.0171	G
rs13135092	Body mass index	0.0501	0.0044	G	Proliferative diabetic retinopathy	0.0125	0.0623	G
rs13174863	Body mass index	0.0250	0.0034	G	Proliferative diabetic retinopathy	0.0139	0.0231	G
rs1320903	Body mass index	0.0220	0.0026	A	Proliferative diabetic retinopathy	0.0058	0.0180	A
rs1327259	Body mass index	-0.0140	0.0025	G	Proliferative diabetic retinopathy	-0.0074	0.0165	G
rs1342391	Body mass index	0.0159	0.0026	T	Proliferative diabetic retinopathy	0.0089	0.0177	T
rs13427822	Body mass index	-0.0199	0.0027	G	Proliferative diabetic retinopathy	-0.0095	0.0179	G
rs1411432	Body mass index	0.0249	0.0031	C	Proliferative diabetic retinopathy	0.0506	0.0216	C
rs1412239	Body mass index	0.0239	0.0026	G	Proliferative diabetic retinopathy	0.0045	0.0170	G
rs1441264	Body mass index	0.0206	0.0025	A	Proliferative diabetic retinopathy	-0.0101	0.0175	A
rs1446585	Body mass index	-0.0171	0.0029	G	Proliferative diabetic retinopathy	-0.0167	0.0173	G
rs1454687	Body mass index	-0.0225	0.0024	G	Proliferative diabetic retinopathy	0.0063	0.0165	G
rs1458156	Body mass index	0.0138	0.0024	T	Proliferative diabetic retinopathy	0.0067	0.0164	T
rs1477290	Body mass index	0.0335	0.0035	C	Proliferative diabetic retinopathy	-0.0154	0.0251	C
rs147730268	Body mass index	-0.0361	0.0043	T	Proliferative diabetic retinopathy	0.0289	0.0296	T
rs1582931	Body mass index	-0.0149	0.0024	A	Proliferative diabetic retinopathy	0.0020	0.0164	A
rs16846140	Body mass index	0.0162	0.0025	G	Proliferative diabetic retinopathy	-0.0239	0.0180	G
rs16916303	Body mass index	-0.0207	0.0037	G	Proliferative diabetic retinopathy	-0.0086	0.0245	G
rs16975459	Body mass index	0.0240	0.0037	C	Proliferative diabetic retinopathy	-0.0147	0.0214	C
rs17014332	Body mass index	0.0180	0.0029	C	Proliferative diabetic retinopathy	-0.0072	0.0208	C
rs17024393	Body mass index	0.0675	0.0076	C	Proliferative diabetic retinopathy	0.0177	0.0356	C
rs17058884	Body mass index	-0.0318	0.0058	G	Proliferative diabetic retinopathy	-0.0135	0.0358	G
rs17085463	Body mass index	-0.0148	0.0026	A	Proliferative diabetic retinopathy	0.0133	0.0175	A
rs17149254	Body mass index	-0.0238	0.0031	C	Proliferative diabetic retinopathy	-0.0048	0.0200	C
rs17342242	Body mass index	-0.0162	0.0029	G	Proliferative diabetic retinopathy	-0.0021	0.0221	G
rs17399739	Body mass index	0.0262	0.0047	G	Proliferative diabetic retinopathy	0.0275	0.0291	G
rs17716502	Body mass index	-0.0223	0.0030	T	Proliferative diabetic retinopathy	-0.0078	0.0208	T
rs1788808	Body mass index	-0.0183	0.0024	G	Proliferative diabetic retinopathy	-0.0034	0.0165	G
rs1805123	Body mass index	-0.0180	0.0028	G	Proliferative diabetic retinopathy	-0.0218	0.0192	G
rs1840661	Body mass index	0.0137	0.0024	A	Proliferative diabetic retinopathy	0.0168	0.0166	A

rs1884897	Body mass index	0.0213	0.0025	G	Proliferative diabetic retinopathy	-0.0310	0.0175	G
rs1901241	Body mass index	0.0193	0.0033	G	Proliferative diabetic retinopathy	0.0003	0.0208	G
rs1919243	Body mass index	0.0139	0.0024	C	Proliferative diabetic retinopathy	-0.0005	0.0167	C
rs1941706	Body mass index	0.0139	0.0024	G	Proliferative diabetic retinopathy	0.0013	0.0164	G
rs1949204	Body mass index	0.0167	0.0028	G	Proliferative diabetic retinopathy	0.0024	0.0184	G
rs2035806	Body mass index	-0.0166	0.0024	A	Proliferative diabetic retinopathy	0.0120	0.0164	A
rs2046002	Body mass index	-0.0146	0.0025	C	Proliferative diabetic retinopathy	-0.0098	0.0164	C
rs2121058	Body mass index	-0.0238	0.0029	C	Proliferative diabetic retinopathy	-0.0301	0.0193	C
rs2135745	Body mass index	-0.0168	0.0028	G	Proliferative diabetic retinopathy	0.0021	0.0198	G
rs2155869	Body mass index	-0.0184	0.0031	C	Proliferative diabetic retinopathy	0.0257	0.0202	C
rs215634	Body mass index	-0.0148	0.0025	G	Proliferative diabetic retinopathy	-0.0225	0.0182	G
rs2192649	Body mass index	0.0137	0.0024	G	Proliferative diabetic retinopathy	0.0035	0.0164	G
rs2234458	Body mass index	-0.0206	0.0025	T	Proliferative diabetic retinopathy	-0.0009	0.0178	T
rs2253310	Body mass index	0.0173	0.0025	G	Proliferative diabetic retinopathy	0.0001	0.0166	G
rs2292238	Body mass index	-0.0177	0.0024	C	Proliferative diabetic retinopathy	0.0184	0.0167	C
rs2307111	Body mass index	-0.0290	0.0025	C	Proliferative diabetic retinopathy	-0.0336	0.0166	C
rs2318543	Body mass index	-0.0194	0.0029	G	Proliferative diabetic retinopathy	-0.0350	0.0198	G
rs2384054	Body mass index	0.0350	0.0024	C	Proliferative diabetic retinopathy	0.0302	0.0166	C
rs2398861	Body mass index	0.0221	0.0028	G	Proliferative diabetic retinopathy	0.0010	0.0176	G
rs241460	Body mass index	-0.0205	0.0026	G	Proliferative diabetic retinopathy	0.0117	0.0169	G
rs2425857	Body mass index	-0.0136	0.0024	G	Proliferative diabetic retinopathy	-0.0305	0.0172	G
rs2439823	Body mass index	0.0216	0.0024	G	Proliferative diabetic retinopathy	0.0097	0.0165	G
rs2450254	Body mass index	-0.0141	0.0024	T	Proliferative diabetic retinopathy	-0.0206	0.0173	T
rs2450445	Body mass index	-0.0154	0.0026	A	Proliferative diabetic retinopathy	0.0010	0.0177	A
rs245775	Body mass index	0.0203	0.0027	G	Proliferative diabetic retinopathy	-0.0039	0.0175	G
rs2470392	Body mass index	0.0145	0.0027	C	Proliferative diabetic retinopathy	0.0083	0.0170	C
rs2474898	Body mass index	0.0146	0.0025	T	Proliferative diabetic retinopathy	0.0031	0.0165	T
rs2482704	Body mass index	-0.0138	0.0024	T	Proliferative diabetic retinopathy	-0.0073	0.0170	T
rs2606228	Body mass index	-0.0156	0.0025	C	Proliferative diabetic retinopathy	-0.0178	0.0167	C
rs2616192	Body mass index	0.0141	0.0026	T	Proliferative diabetic retinopathy	0.0255	0.0169	T
rs2678204	Body mass index	0.0281	0.0025	G	Proliferative diabetic retinopathy	-0.0105	0.0180	G

rs2711111	Body mass index	-0.0147	0.0025	G	Proliferative diabetic retinopathy	0.0136	0.0168	G
rs2725371	Body mass index	-0.0181	0.0026	G	Proliferative diabetic retinopathy	-0.0289	0.0187	G
rs273505	Body mass index	0.0189	0.0024	C	Proliferative diabetic retinopathy	0.0129	0.0164	C
rs28366156	Body mass index	-0.0290	0.0036	C	Proliferative diabetic retinopathy	-0.1764	0.0320	C
rs28447555	Body mass index	0.0185	0.0031	T	Proliferative diabetic retinopathy	0.0148	0.0234	T
rs28489620	Body mass index	-0.0147	0.0027	A	Proliferative diabetic retinopathy	0.0190	0.0180	A
rs2861685	Body mass index	-0.0176	0.0024	C	Proliferative diabetic retinopathy	-0.0072	0.0166	C
rs286818	Body mass index	-0.0291	0.0032	A	Proliferative diabetic retinopathy	0.0518	0.0201	A
rs2962082	Body mass index	-0.0135	0.0024	A	Proliferative diabetic retinopathy	-0.0013	0.0165	A
rs2975693	Body mass index	0.0238	0.0039	C	Proliferative diabetic retinopathy	0.0173	0.0320	C
rs34045288	Body mass index	0.0260	0.0025	T	Proliferative diabetic retinopathy	0.0428	0.0169	T
rs34095326	Body mass index	-0.0244	0.0038	A	Proliferative diabetic retinopathy	-0.0030	0.0223	A
rs34236292	Body mass index	-0.0141	0.0026	T	Proliferative diabetic retinopathy	-0.0070	0.0189	T
rs34361149	Body mass index	-0.0310	0.0030	A	Proliferative diabetic retinopathy	0.0222	0.0238	A
rs34373881	Body mass index	-0.0169	0.0027	A	Proliferative diabetic retinopathy	-0.0204	0.0186	A
rs34774377	Body mass index	-0.0208	0.0037	C	Proliferative diabetic retinopathy	0.0005	0.0265	C
rs34811474	Body mass index	-0.0306	0.0028	A	Proliferative diabetic retinopathy	-0.0055	0.0195	A
rs34966008	Body mass index	-0.0199	0.0024	T	Proliferative diabetic retinopathy	-0.0210	0.0170	T
rs35025195	Body mass index	-0.0230	0.0032	A	Proliferative diabetic retinopathy	-0.0282	0.0238	A
rs35193668	Body mass index	-0.0167	0.0025	T	Proliferative diabetic retinopathy	-0.0436	0.0166	T
rs35483388	Body mass index	0.0141	0.0025	T	Proliferative diabetic retinopathy	0.0144	0.0167	T
rs35626515	Body mass index	0.0266	0.0024	A	Proliferative diabetic retinopathy	-0.0349	0.0164	A
rs35722922	Body mass index	-0.0165	0.0025	G	Proliferative diabetic retinopathy	-0.0137	0.0174	G
rs357501	Body mass index	0.0154	0.0025	A	Proliferative diabetic retinopathy	-0.0330	0.0167	A
rs35851183	Body mass index	0.0168	0.0025	G	Proliferative diabetic retinopathy	0.0304	0.0179	G
rs35882248	Body mass index	0.0192	0.0026	T	Proliferative diabetic retinopathy	0.0007	0.0177	T
rs36007635	Body mass index	-0.0200	0.0035	A	Proliferative diabetic retinopathy	-0.0134	0.0285	A
rs362307	Body mass index	0.0314	0.0046	T	Proliferative diabetic retinopathy	0.0332	0.0325	T
rs3759584	Body mass index	-0.0166	0.0025	C	Proliferative diabetic retinopathy	-0.0261	0.0177	C
rs3802858	Body mass index	-0.0165	0.0024	C	Proliferative diabetic retinopathy	-0.0267	0.0165	C
rs3803286	Body mass index	-0.0208	0.0025	G	Proliferative diabetic retinopathy	-0.0190	0.0173	G

rs3810291	Body mass index	0.0297	0.0026	A	Proliferative diabetic retinopathy	0.0238	0.0170	A
rs3843540	Body mass index	-0.0242	0.0034	C	Proliferative diabetic retinopathy	0.0586	0.0196	C
rs3844598	Body mass index	0.0137	0.0024	G	Proliferative diabetic retinopathy	0.0156	0.0165	G
rs3861879	Body mass index	0.0137	0.0024	G	Proliferative diabetic retinopathy	0.0131	0.0167	G
rs3897102	Body mass index	0.0147	0.0025	T	Proliferative diabetic retinopathy	0.0178	0.0166	T
rs390192	Body mass index	-0.0154	0.0024	G	Proliferative diabetic retinopathy	-0.0167	0.0165	G
rs39330	Body mass index	-0.0189	0.0024	T	Proliferative diabetic retinopathy	0.0058	0.0165	T
rs4246657	Body mass index	0.0170	0.0025	T	Proliferative diabetic retinopathy	0.0217	0.0176	T
rs4261944	Body mass index	0.0155	0.0025	G	Proliferative diabetic retinopathy	0.0177	0.0178	G
rs4402589	Body mass index	0.0292	0.0024	G	Proliferative diabetic retinopathy	-0.0133	0.0164	G
rs4467770	Body mass index	0.0157	0.0027	A	Proliferative diabetic retinopathy	0.0289	0.0182	A
rs4474229	Body mass index	-0.0159	0.0025	A	Proliferative diabetic retinopathy	-0.0210	0.0173	A
rs4482463	Body mass index	-0.0341	0.0045	A	Proliferative diabetic retinopathy	-0.0083	0.0349	A
rs4502882	Body mass index	-0.0147	0.0025	T	Proliferative diabetic retinopathy	0.0164	0.0179	T
rs4595495	Body mass index	0.0137	0.0024	G	Proliferative diabetic retinopathy	0.0092	0.0165	G
rs4648450	Body mass index	-0.0154	0.0024	A	Proliferative diabetic retinopathy	0.0100	0.0165	A
rs4687770	Body mass index	-0.0193	0.0035	C	Proliferative diabetic retinopathy	0.0144	0.0226	C
rs4718964	Body mass index	0.0146	0.0024	T	Proliferative diabetic retinopathy	-0.0271	0.0167	T
rs4757144	Body mass index	0.0165	0.0024	A	Proliferative diabetic retinopathy	-0.0023	0.0164	A
rs4776970	Body mass index	-0.0255	0.0025	T	Proliferative diabetic retinopathy	-0.0082	0.0172	T
rs4777541	Body mass index	0.0197	0.0028	T	Proliferative diabetic retinopathy	0.0339	0.0237	T
rs4911382	Body mass index	0.0156	0.0024	T	Proliferative diabetic retinopathy	0.0048	0.0172	T
rs491711	Body mass index	-0.0158	0.0026	C	Proliferative diabetic retinopathy	-0.0038	0.0195	C
rs4921301	Body mass index	-0.0182	0.0030	T	Proliferative diabetic retinopathy	-0.0044	0.0201	T
rs4929923	Body mass index	0.0174	0.0025	C	Proliferative diabetic retinopathy	0.0011	0.0171	C
rs525101	Body mass index	0.0165	0.0025	C	Proliferative diabetic retinopathy	0.0045	0.0165	C
rs539515	Body mass index	0.0475	0.0030	C	Proliferative diabetic retinopathy	-0.0029	0.0214	C
rs550974	Body mass index	0.0159	0.0024	T	Proliferative diabetic retinopathy	0.0099	0.0180	T
rs55689274	Body mass index	-0.0148	0.0027	A	Proliferative diabetic retinopathy	0.0103	0.0186	A
rs55726687	Body mass index	0.0239	0.0029	A	Proliferative diabetic retinopathy	-0.0049	0.0200	A
rs55886426	Body mass index	-0.0316	0.0055	G	Proliferative diabetic retinopathy	-0.0495	0.0412	G

rs55938344	Body mass index	-0.0168	0.0028	C	Proliferative diabetic retinopathy	0.0260	0.0189	C
rs56067609	Body mass index	-0.0194	0.0035	T	Proliferative diabetic retinopathy	-0.0140	0.0239	T
rs56161855	Body mass index	0.0230	0.0035	T	Proliferative diabetic retinopathy	-0.0835	0.0229	T
rs56212061	Body mass index	-0.0211	0.0034	T	Proliferative diabetic retinopathy	-0.0122	0.0278	T
rs56773984	Body mass index	-0.0179	0.0032	T	Proliferative diabetic retinopathy	-0.0074	0.0368	T
rs56803094	Body mass index	-0.0189	0.0029	G	Proliferative diabetic retinopathy	-0.0143	0.0184	G
rs57636386	Body mass index	-0.0428	0.0044	C	Proliferative diabetic retinopathy	-0.0175	0.0380	C
rs58862095	Body mass index	-0.0244	0.0024	T	Proliferative diabetic retinopathy	-0.0046	0.0164	T
rs588660	Body mass index	0.0183	0.0024	A	Proliferative diabetic retinopathy	-0.0080	0.0167	A
rs59104534	Body mass index	0.0150	0.0026	T	Proliferative diabetic retinopathy	0.0148	0.0175	T
rs5995843	Body mass index	-0.0174	0.0025	G	Proliferative diabetic retinopathy	0.0027	0.0170	G
rs6050446	Body mass index	0.0421	0.0068	G	Proliferative diabetic retinopathy	0.0075	0.0556	G
rs60654199	Body mass index	0.0313	0.0048	A	Proliferative diabetic retinopathy	-0.0274	0.0382	A
rs60764613	Body mass index	0.0236	0.0034	T	Proliferative diabetic retinopathy	0.0263	0.0216	T
rs61813324	Body mass index	0.0279	0.0036	T	Proliferative diabetic retinopathy	-0.0150	0.0227	T
rs61826867	Body mass index	0.0251	0.0038	G	Proliferative diabetic retinopathy	0.0678	0.0297	G
rs61871615	Body mass index	-0.0280	0.0044	T	Proliferative diabetic retinopathy	-0.0414	0.0305	T
rs61903695	Body mass index	0.0164	0.0028	G	Proliferative diabetic retinopathy	0.0104	0.0191	G
rs61969510	Body mass index	0.0155	0.0027	C	Proliferative diabetic retinopathy	0.0241	0.0170	C
rs62106258	Body mass index	-0.0907	0.0056	C	Proliferative diabetic retinopathy	-0.0616	0.0580	C
rs62147189	Body mass index	-0.0170	0.0025	G	Proliferative diabetic retinopathy	-0.0086	0.0167	G
rs62246314	Body mass index	0.0228	0.0040	A	Proliferative diabetic retinopathy	-0.0169	0.0335	A
rs62407562	Body mass index	0.0171	0.0027	A	Proliferative diabetic retinopathy	0.0264	0.0211	A
rs62543438	Body mass index	-0.0148	0.0027	C	Proliferative diabetic retinopathy	-0.0265	0.0202	C
rs6265	Body mass index	-0.0402	0.0031	T	Proliferative diabetic retinopathy	0.0301	0.0226	T
rs6536575	Body mass index	0.0141	0.0024	C	Proliferative diabetic retinopathy	-0.0182	0.0166	C
rs6575340	Body mass index	0.0229	0.0025	A	Proliferative diabetic retinopathy	0.0085	0.0170	A
rs6601527	Body mass index	-0.0217	0.0024	A	Proliferative diabetic retinopathy	-0.0050	0.0237	A
rs66679256	Body mass index	0.0163	0.0024	T	Proliferative diabetic retinopathy	-0.0128	0.0170	T
rs6687953	Body mass index	0.0159	0.0025	G	Proliferative diabetic retinopathy	-0.0012	0.0164	G
rs66922415	Body mass index	0.0521	0.0028	G	Proliferative diabetic retinopathy	0.0374	0.0211	G

rs6705567	Body mass index	-0.0136	0.0025	C	Proliferative diabetic retinopathy	-0.0132	0.0168	C
rs6722241	Body mass index	-0.0202	0.0027	C	Proliferative diabetic retinopathy	-0.0502	0.0209	C
rs6739755	Body mass index	-0.0210	0.0025	G	Proliferative diabetic retinopathy	-0.0373	0.0167	G
rs67609008	Body mass index	0.0146	0.0027	C	Proliferative diabetic retinopathy	-0.0070	0.0214	C
rs6780459	Body mass index	0.0164	0.0028	T	Proliferative diabetic retinopathy	0.0265	0.0200	T
rs67844506	Body mass index	-0.0259	0.0031	G	Proliferative diabetic retinopathy	0.0078	0.0225	G
rs6789488	Body mass index	0.0200	0.0028	C	Proliferative diabetic retinopathy	-0.0260	0.0229	C
rs6809307	Body mass index	0.0157	0.0028	T	Proliferative diabetic retinopathy	-0.0059	0.0214	T
rs6831020	Body mass index	-0.0159	0.0026	A	Proliferative diabetic retinopathy	0.0016	0.0194	A
rs6861649	Body mass index	0.0143	0.0025	C	Proliferative diabetic retinopathy	0.0022	0.0170	C
rs6950388	Body mass index	0.0172	0.0030	A	Proliferative diabetic retinopathy	0.0335	0.0197	A
rs7006178	Body mass index	0.0155	0.0027	C	Proliferative diabetic retinopathy	0.0199	0.0190	C
rs7030732	Body mass index	-0.0152	0.0025	A	Proliferative diabetic retinopathy	-0.0027	0.0169	A
rs704061	Body mass index	0.0159	0.0024	C	Proliferative diabetic retinopathy	-0.0071	0.0169	C
rs7094644	Body mass index	0.0153	0.0026	A	Proliferative diabetic retinopathy	-0.0264	0.0178	A
rs7116641	Body mass index	0.0244	0.0026	G	Proliferative diabetic retinopathy	0.0237	0.0177	G
rs7124681	Body mass index	0.0267	0.0024	A	Proliferative diabetic retinopathy	0.0094	0.0172	A
rs7132908	Body mass index	0.0285	0.0025	A	Proliferative diabetic retinopathy	0.0021	0.0169	A
rs7138383	Body mass index	-0.0214	0.0028	A	Proliferative diabetic retinopathy	0.0003	0.0178	A
rs7141420	Body mass index	0.0208	0.0024	T	Proliferative diabetic retinopathy	0.0165	0.0167	T
rs71495049	Body mass index	0.0265	0.0043	A	Proliferative diabetic retinopathy	0.0148	0.0258	A
rs7163362	Body mass index	0.0132	0.0024	A	Proliferative diabetic retinopathy	0.0098	0.0170	A
rs7183417	Body mass index	0.0149	0.0024	T	Proliferative diabetic retinopathy	0.0307	0.0175	T
rs7189149	Body mass index	0.0213	0.0036	G	Proliferative diabetic retinopathy	-0.0497	0.0252	G
rs7195386	Body mass index	-0.0159	0.0024	C	Proliferative diabetic retinopathy	0.0077	0.0164	C
rs7201895	Body mass index	-0.0173	0.0025	A	Proliferative diabetic retinopathy	0.0056	0.0180	A
rs7218014	Body mass index	0.0207	0.0030	C	Proliferative diabetic retinopathy	0.0293	0.0192	C
rs72697614	Body mass index	0.0150	0.0026	A	Proliferative diabetic retinopathy	-0.0079	0.0181	A
rs72820274	Body mass index	0.0160	0.0024	A	Proliferative diabetic retinopathy	-0.0102	0.0165	A
rs72892910	Body mass index	0.0402	0.0032	T	Proliferative diabetic retinopathy	0.0284	0.0203	T
rs72976986	Body mass index	-0.0240	0.0031	A	Proliferative diabetic retinopathy	-0.0489	0.0225	A

rs73050254	Body mass index	0.0193	0.0035	A	Proliferative diabetic retinopathy	-0.0279	0.0322	A
rs73144053	Body mass index	-0.0154	0.0026	A	Proliferative diabetic retinopathy	-0.0202	0.0178	A
rs73169730	Body mass index	0.0195	0.0027	G	Proliferative diabetic retinopathy	-0.0206	0.0189	G
rs7321331	Body mass index	0.0176	0.0028	A	Proliferative diabetic retinopathy	0.0130	0.0175	A
rs73213484	Body mass index	-0.0212	0.0035	T	Proliferative diabetic retinopathy	-0.0067	0.0231	T
rs7331420	Body mass index	-0.0147	0.0027	A	Proliferative diabetic retinopathy	0.0061	0.0181	A
rs7442885	Body mass index	-0.0254	0.0030	G	Proliferative diabetic retinopathy	0.0413	0.0210	G
rs7498044	Body mass index	-0.0176	0.0029	A	Proliferative diabetic retinopathy	-0.0141	0.0189	A
rs750090	Body mass index	-0.0156	0.0025	C	Proliferative diabetic retinopathy	-0.0117	0.0167	C
rs752179	Body mass index	-0.0144	0.0026	A	Proliferative diabetic retinopathy	-0.0043	0.0173	A
rs75499503	Body mass index	-0.0198	0.0029	T	Proliferative diabetic retinopathy	0.0096	0.0196	T
rs7553158	Body mass index	-0.0174	0.0024	A	Proliferative diabetic retinopathy	-0.0082	0.0164	A
rs75557510	Body mass index	-0.0378	0.0051	G	Proliferative diabetic retinopathy	-0.0435	0.0383	G
rs756717	Body mass index	-0.0147	0.0025	A	Proliferative diabetic retinopathy	0.0127	0.0168	A
rs7568228	Body mass index	-0.0140	0.0024	C	Proliferative diabetic retinopathy	0.0008	0.0164	C
rs76040172	Body mass index	-0.0408	0.0053	A	Proliferative diabetic retinopathy	-0.0910	0.0300	A
rs7701777	Body mass index	-0.0170	0.0027	G	Proliferative diabetic retinopathy	-0.0054	0.0191	G
rs7719067	Body mass index	-0.0162	0.0024	G	Proliferative diabetic retinopathy	-0.0121	0.0167	G
rs7723426	Body mass index	0.0147	0.0026	C	Proliferative diabetic retinopathy	0.0146	0.0165	C
rs7755574	Body mass index	0.0155	0.0027	T	Proliferative diabetic retinopathy	0.0101	0.0174	T
rs7774	Body mass index	0.0166	0.0026	A	Proliferative diabetic retinopathy	0.0013	0.0168	A
rs778094	Body mass index	-0.0149	0.0024	A	Proliferative diabetic retinopathy	-0.0122	0.0165	A
rs7852189	Body mass index	0.0166	0.0026	G	Proliferative diabetic retinopathy	0.0179	0.0168	G
rs78565420	Body mass index	0.0337	0.0055	T	Proliferative diabetic retinopathy	0.0521	0.0417	T
rs79113395	Body mass index	-0.0209	0.0027	A	Proliferative diabetic retinopathy	-0.0058	0.0199	A
rs7933085	Body mass index	0.0157	0.0024	G	Proliferative diabetic retinopathy	-0.0176	0.0164	G
rs7941828	Body mass index	-0.0156	0.0025	T	Proliferative diabetic retinopathy	0.0021	0.0178	T
rs7952102	Body mass index	-0.0152	0.0025	C	Proliferative diabetic retinopathy	-0.0104	0.0172	C
rs7992832	Body mass index	-0.0176	0.0027	T	Proliferative diabetic retinopathy	-0.0300	0.0189	T
rs799449	Body mass index	0.0199	0.0024	T	Proliferative diabetic retinopathy	-0.0128	0.0166	T
rs8015400	Body mass index	0.0211	0.0026	A	Proliferative diabetic retinopathy	0.0185	0.0171	A

rs80330591	Body mass index	-0.0214	0.0034	A	Proliferative diabetic retinopathy	-0.0248	0.0213	A
rs8078135	Body mass index	-0.0195	0.0024	T	Proliferative diabetic retinopathy	-0.0183	0.0164	T
rs8134638	Body mass index	0.0152	0.0025	C	Proliferative diabetic retinopathy	0.0115	0.0164	C
rs815163	Body mass index	-0.0182	0.0024	C	Proliferative diabetic retinopathy	0.0108	0.0164	C
rs815715	Body mass index	-0.0163	0.0024	G	Proliferative diabetic retinopathy	-0.0062	0.0164	G
rs845084	Body mass index	0.0203	0.0028	A	Proliferative diabetic retinopathy	0.0217	0.0187	A
rs862320	Body mass index	-0.0228	0.0024	T	Proliferative diabetic retinopathy	-0.0157	0.0166	T
rs869400	Body mass index	0.0307	0.0031	G	Proliferative diabetic retinopathy	0.0275	0.0223	G
rs879620	Body mass index	0.0256	0.0025	T	Proliferative diabetic retinopathy	0.0159	0.0166	T
rs9267671	Body mass index	0.0324	0.0050	A	Proliferative diabetic retinopathy	-0.1131	0.0558	A
rs9291822	Body mass index	-0.0136	0.0024	T	Proliferative diabetic retinopathy	-0.0101	0.0168	T
rs9320823	Body mass index	0.0189	0.0025	C	Proliferative diabetic retinopathy	0.0041	0.0175	C
rs9342196	Body mass index	0.0167	0.0031	T	Proliferative diabetic retinopathy	-0.0260	0.0233	T
rs935166	Body mass index	-0.0155	0.0024	A	Proliferative diabetic retinopathy	-0.0072	0.0166	A
rs9402104	Body mass index	0.0139	0.0025	A	Proliferative diabetic retinopathy	-0.0073	0.0170	A
rs946185	Body mass index	-0.0147	0.0025	G	Proliferative diabetic retinopathy	-0.0271	0.0168	G
rs9489620	Body mass index	0.0148	0.0024	C	Proliferative diabetic retinopathy	0.0325	0.0167	C
rs9515455	Body mass index	0.0180	0.0024	A	Proliferative diabetic retinopathy	-0.0070	0.0164	A
rs9527906	Body mass index	-0.0162	0.0028	A	Proliferative diabetic retinopathy	0.0228	0.0191	A
rs9536449	Body mass index	-0.0136	0.0024	G	Proliferative diabetic retinopathy	-0.0156	0.0165	G
rs9641499	Body mass index	-0.0171	0.0024	A	Proliferative diabetic retinopathy	-0.0065	0.0168	A
rs9688977	Body mass index	0.0240	0.0034	C	Proliferative diabetic retinopathy	0.0239	0.0214	C
rs9843653	Body mass index	0.0317	0.0024	C	Proliferative diabetic retinopathy	0.0008	0.0168	C
rs9847186	Body mass index	-0.0143	0.0024	A	Proliferative diabetic retinopathy	-0.0186	0.0167	A
rs11725618	Cigarettes per Day	0.0361	0.0062	C	Background diabetic retinopathy	0.0152	0.0370	C
rs1579233	Cigarettes per Day	-0.0318	0.0056	G	Background diabetic retinopathy	0.0346	0.0349	G
rs2072659	Cigarettes per Day	-0.0653	0.0092	G	Background diabetic retinopathy	-0.0416	0.0544	G
rs2084533	Cigarettes per Day	0.0336	0.0059	T	Background diabetic retinopathy	0.0159	0.0357	T
rs215600	Cigarettes per Day	-0.0493	0.0058	A	Background diabetic retinopathy	-0.0045	0.0366	A
rs2273500	Cigarettes per Day	0.0681	0.0078	C	Background diabetic retinopathy	0.0226	0.0400	C
rs2424888	Cigarettes per Day	0.0335	0.0056	A	Background diabetic retinopathy	0.0287	0.0329	A

rs3025383	Cigarettes per Day	-0.0578	0.0070	C	Background diabetic retinopathy	0.0321	0.0436	C
rs34406232	Cigarettes per Day	-0.1470	0.0167	A	Background diabetic retinopathy	0.0810	0.1051	A
rs4785587	Cigarettes per Day	-0.0336	0.0055	A	Background diabetic retinopathy	-0.0655	0.0343	A
rs56113850	Cigarettes per Day	0.1072	0.0056	C	Background diabetic retinopathy	0.0025	0.0328	C
rs58379124	Cigarettes per Day	0.0669	0.0065	C	Background diabetic retinopathy	-0.0057	0.0381	C
rs632811	Cigarettes per Day	-0.0367	0.0064	G	Background diabetic retinopathy	0.0136	0.0329	G
rs73229090	Cigarettes per Day	0.0555	0.0088	A	Background diabetic retinopathy	-0.0648	0.0560	A
rs7431710	Cigarettes per Day	-0.0350	0.0058	A	Background diabetic retinopathy	-0.0153	0.0347	A
rs75494138	Cigarettes per Day	0.0599	0.0106	T	Background diabetic retinopathy	0.0319	0.0823	T
rs787362	Cigarettes per Day	0.0305	0.0056	A	Background diabetic retinopathy	-0.0174	0.0328	A
rs790564	Cigarettes per Day	-0.0409	0.0062	C	Background diabetic retinopathy	0.0289	0.0384	C
rs7928017	Cigarettes per Day	-0.0329	0.0056	A	Background diabetic retinopathy	-0.0148	0.0366	A
rs7951365	Cigarettes per Day	0.0390	0.0060	C	Background diabetic retinopathy	-0.0439	0.0385	C
rs8034191	Cigarettes per Day	0.1826	0.0059	C	Background diabetic retinopathy	0.0429	0.0346	C
rs806798	Cigarettes per Day	-0.0309	0.0055	C	Background diabetic retinopathy	0.0019	0.0336	C
rs895330	Cigarettes per Day	-0.0390	0.0070	G	Background diabetic retinopathy	-0.0411	0.0441	G
rs11725618	Cigarettes per Day	0.0361	0.0062	C	Diabetic retinopathy	-0.0094	0.0148	C
rs1579233	Cigarettes per Day	-0.0318	0.0056	G	Diabetic retinopathy	0.0031	0.0139	G
rs2072659	Cigarettes per Day	-0.0653	0.0092	G	Diabetic retinopathy	0.0127	0.0219	G
rs2084533	Cigarettes per Day	0.0336	0.0059	T	Diabetic retinopathy	0.0179	0.0143	T
rs215600	Cigarettes per Day	-0.0493	0.0058	A	Diabetic retinopathy	-0.0293	0.0147	A
rs2273500	Cigarettes per Day	0.0681	0.0078	C	Diabetic retinopathy	-0.0017	0.0160	C
rs2424888	Cigarettes per Day	0.0335	0.0056	A	Diabetic retinopathy	0.0049	0.0132	A
rs3025383	Cigarettes per Day	-0.0578	0.0070	C	Diabetic retinopathy	-0.0077	0.0175	C
rs34406232	Cigarettes per Day	-0.1470	0.0167	A	Diabetic retinopathy	-0.0240	0.0418	A
rs4785587	Cigarettes per Day	-0.0336	0.0055	A	Diabetic retinopathy	-0.0175	0.0138	A
rs56113850	Cigarettes per Day	0.1072	0.0056	C	Diabetic retinopathy	0.0014	0.0132	C
rs58379124	Cigarettes per Day	0.0669	0.0065	C	Diabetic retinopathy	0.0214	0.0153	C
rs632811	Cigarettes per Day	-0.0367	0.0064	G	Diabetic retinopathy	0.0083	0.0132	G
rs73229090	Cigarettes per Day	0.0555	0.0088	A	Diabetic retinopathy	-0.0555	0.0224	A
rs7431710	Cigarettes per Day	-0.0350	0.0058	A	Diabetic retinopathy	-0.0078	0.0139	A

rs75494138	Cigarettes per Day	0.0599	0.0106	T	Diabetic retinopathy	0.0208	0.0331	T
rs787362	Cigarettes per Day	0.0305	0.0056	A	Diabetic retinopathy	-0.0004	0.0132	A
rs790564	Cigarettes per Day	-0.0409	0.0062	C	Diabetic retinopathy	0.0137	0.0154	C
rs7928017	Cigarettes per Day	-0.0329	0.0056	A	Diabetic retinopathy	-0.0341	0.0147	A
rs7951365	Cigarettes per Day	0.0390	0.0060	C	Diabetic retinopathy	0.0221	0.0154	C
rs8034191	Cigarettes per Day	0.1826	0.0059	C	Diabetic retinopathy	0.0370	0.0139	C
rs806798	Cigarettes per Day	-0.0309	0.0055	C	Diabetic retinopathy	0.0079	0.0135	C
rs895330	Cigarettes per Day	-0.0390	0.0070	G	Diabetic retinopathy	-0.0455	0.0178	G
rs11725618	Cigarettes per Day	0.0361	0.0062	C	Proliferative diabetic retinopathy	-0.0194	0.0185	C
rs1579233	Cigarettes per Day	-0.0318	0.0056	G	Proliferative diabetic retinopathy	0.0033	0.0174	G
rs2072659	Cigarettes per Day	-0.0653	0.0092	G	Proliferative diabetic retinopathy	-0.0072	0.0273	G
rs2084533	Cigarettes per Day	0.0336	0.0059	T	Proliferative diabetic retinopathy	-0.0029	0.0179	T
rs215600	Cigarettes per Day	-0.0493	0.0058	A	Proliferative diabetic retinopathy	-0.0154	0.0183	A
rs2273500	Cigarettes per Day	0.0681	0.0078	C	Proliferative diabetic retinopathy	0.0054	0.0200	C
rs2424888	Cigarettes per Day	0.0335	0.0056	A	Proliferative diabetic retinopathy	0.0163	0.0164	A
rs3025383	Cigarettes per Day	-0.0578	0.0070	C	Proliferative diabetic retinopathy	-0.0006	0.0218	C
rs34406232	Cigarettes per Day	-0.1470	0.0167	A	Proliferative diabetic retinopathy	-0.0565	0.0525	A
rs4785587	Cigarettes per Day	-0.0336	0.0055	A	Proliferative diabetic retinopathy	-0.0414	0.0171	A
rs56113850	Cigarettes per Day	0.1072	0.0056	C	Proliferative diabetic retinopathy	0.0062	0.0164	C
rs58379124	Cigarettes per Day	0.0669	0.0065	C	Proliferative diabetic retinopathy	0.0353	0.0191	C
rs632811	Cigarettes per Day	-0.0367	0.0064	G	Proliferative diabetic retinopathy	0.0097	0.0164	G
rs73229090	Cigarettes per Day	0.0555	0.0088	A	Proliferative diabetic retinopathy	-0.0609	0.0281	A
rs7431710	Cigarettes per Day	-0.0350	0.0058	A	Proliferative diabetic retinopathy	-0.0042	0.0174	A
rs75494138	Cigarettes per Day	0.0599	0.0106	T	Proliferative diabetic retinopathy	0.0312	0.0413	T
rs787362	Cigarettes per Day	0.0305	0.0056	A	Proliferative diabetic retinopathy	-0.0022	0.0164	A
rs790564	Cigarettes per Day	-0.0409	0.0062	C	Proliferative diabetic retinopathy	0.0247	0.0193	C
rs7928017	Cigarettes per Day	-0.0329	0.0056	A	Proliferative diabetic retinopathy	-0.0084	0.0183	A
rs7951365	Cigarettes per Day	0.0390	0.0060	C	Proliferative diabetic retinopathy	0.0206	0.0192	C
rs8034191	Cigarettes per Day	0.1826	0.0059	C	Proliferative diabetic retinopathy	0.0357	0.0173	C
rs806798	Cigarettes per Day	-0.0309	0.0055	C	Proliferative diabetic retinopathy	-0.0021	0.0168	C
rs895330	Cigarettes per Day	-0.0390	0.0070	G	Proliferative diabetic retinopathy	-0.0500	0.0221	G

rs10179482	Ever smoked	0.0058	0.0010	A	Background diabetic retinopathy	-0.0131	0.0330	A
rs10212155	Ever smoked	0.0104	0.0014	A	Background diabetic retinopathy	-0.0069	0.0399	A
rs10233018	Ever smoked	0.0078	0.0010	G	Background diabetic retinopathy	0.0447	0.0335	G
rs1040070	Ever smoked	-0.0059	0.0010	C	Background diabetic retinopathy	0.0242	0.0327	C
rs10774625	Ever smoked	-0.0066	0.0010	G	Background diabetic retinopathy	-0.1429	0.0331	G
rs10863714	Ever smoked	-0.0058	0.0010	G	Background diabetic retinopathy	0.0317	0.0335	G
rs10952199	Ever smoked	-0.0069	0.0010	T	Background diabetic retinopathy	-0.0434	0.0329	T
rs10956808	Ever smoked	-0.0067	0.0010	G	Background diabetic retinopathy	-0.0089	0.0327	G
rs10988799	Ever smoked	0.0059	0.0010	T	Background diabetic retinopathy	-0.0068	0.0328	T
rs11165623	Ever smoked	0.0055	0.0010	A	Background diabetic retinopathy	-0.0066	0.0331	A
rs1124639	Ever smoked	0.0066	0.0010	C	Background diabetic retinopathy	0.0387	0.0328	C
rs1150023	Ever smoked	-0.0065	0.0011	C	Background diabetic retinopathy	-0.0638	0.0380	C
rs1174864	Ever smoked	0.0062	0.0010	A	Background diabetic retinopathy	0.0254	0.0334	A
rs12209519	Ever smoked	0.0057	0.0010	G	Background diabetic retinopathy	-0.0228	0.0341	G
rs12244388	Ever smoked	0.0086	0.0011	A	Background diabetic retinopathy	0.0254	0.0337	A
rs12272735	Ever smoked	0.0058	0.0010	G	Background diabetic retinopathy	-0.0201	0.0338	G
rs12333760	Ever smoked	-0.0083	0.0014	C	Background diabetic retinopathy	0.0479	0.0390	C
rs12450028	Ever smoked	0.0061	0.0011	T	Background diabetic retinopathy	-0.0460	0.0347	T
rs1246265	Ever smoked	0.0061	0.0011	C	Background diabetic retinopathy	-0.0440	0.0366	C
rs12902636	Ever smoked	-0.0059	0.0010	T	Background diabetic retinopathy	-0.0172	0.0336	T
rs13001103	Ever smoked	-0.0057	0.0010	G	Background diabetic retinopathy	0.0360	0.0327	G
rs13162305	Ever smoked	0.0060	0.0011	T	Background diabetic retinopathy	0.0305	0.0346	T
rs1322525	Ever smoked	0.0064	0.0010	G	Background diabetic retinopathy	-0.0223	0.0365	G
rs1324481	Ever smoked	0.0069	0.0011	G	Background diabetic retinopathy	0.0587	0.0357	G
rs13246563	Ever smoked	-0.0066	0.0010	G	Background diabetic retinopathy	0.0061	0.0337	G
rs13246563	Ever smoked	-0.0066	0.0010	G	Background diabetic retinopathy	0.0061	0.3202	G
rs13246563	Ever smoked	-0.0066	0.0010	G	Background diabetic retinopathy	0.2825	0.0337	A
rs13246563	Ever smoked	-0.0066	0.0010	G	Background diabetic retinopathy	0.2825	0.3202	A
rs1363101	Ever smoked	-0.0058	0.0010	G	Background diabetic retinopathy	-0.0093	0.0329	G
rs1373178	Ever smoked	-0.0062	0.0010	G	Background diabetic retinopathy	-0.0008	0.0341	G
rs150294	Ever smoked	-0.0076	0.0010	G	Background diabetic retinopathy	-0.0286	0.0330	G

rs1549212	Ever smoked	-0.0066	0.0010	T	Background diabetic retinopathy	-0.0239	0.0344	T
rs1718705	Ever smoked	0.0065	0.0011	C	Background diabetic retinopathy	-0.0217	0.0358	C
rs17584022	Ever smoked	-0.0066	0.0011	A	Background diabetic retinopathy	-0.0219	0.0363	A
rs1876066	Ever smoked	-0.0057	0.0010	C	Background diabetic retinopathy	-0.0026	0.0334	C
rs1899896	Ever smoked	0.0072	0.0011	T	Background diabetic retinopathy	-0.0107	0.0372	T
rs2155292	Ever smoked	0.0147	0.0010	G	Background diabetic retinopathy	-0.0463	0.0341	G
rs2175207	Ever smoked	0.0076	0.0014	G	Background diabetic retinopathy	0.0178	0.0403	G
rs2183573	Ever smoked	-0.0058	0.0010	G	Background diabetic retinopathy	-0.0597	0.0354	G
rs2516436	Ever smoked	-0.0057	0.0010	G	Background diabetic retinopathy	-0.0927	0.0333	G
rs28809490	Ever smoked	-0.0063	0.0011	A	Background diabetic retinopathy	-0.0194	0.0387	A
rs303753	Ever smoked	-0.0061	0.0011	A	Background diabetic retinopathy	0.0181	0.0343	A
rs34335016	Ever smoked	-0.0086	0.0015	T	Background diabetic retinopathy	0.0030	0.0432	T
rs35498642	Ever smoked	-0.0062	0.0010	T	Background diabetic retinopathy	0.0389	0.0335	T
rs35892365	Ever smoked	-0.0067	0.0012	T	Background diabetic retinopathy	-0.0421	0.0341	T
rs3783177	Ever smoked	-0.0073	0.0012	G	Background diabetic retinopathy	0.0008	0.0396	G
rs3790286	Ever smoked	0.0056	0.0010	C	Background diabetic retinopathy	-0.0375	0.0331	C
rs41513151	Ever smoked	0.0073	0.0012	A	Background diabetic retinopathy	0.0591	0.0409	A
rs4422110	Ever smoked	-0.0088	0.0010	T	Background diabetic retinopathy	0.0040	0.0330	T
rs465646	Ever smoked	-0.0117	0.0014	A	Background diabetic retinopathy	0.0152	0.0411	A
rs4680392	Ever smoked	0.0062	0.0011	C	Background diabetic retinopathy	0.0277	0.0355	C
rs4856598	Ever smoked	-0.0084	0.0010	C	Background diabetic retinopathy	-0.0599	0.0364	C
rs528301	Ever smoked	0.0076	0.0010	A	Background diabetic retinopathy	-0.0465	0.0343	A
rs529206	Ever smoked	-0.0059	0.0010	T	Background diabetic retinopathy	0.0101	0.0334	T
rs55864295	Ever smoked	-0.0074	0.0013	G	Background diabetic retinopathy	-0.0059	0.0422	G
rs56166763	Ever smoked	-0.0058	0.0010	C	Background diabetic retinopathy	0.0325	0.0342	C
rs58400863	Ever smoked	-0.0063	0.0011	A	Background diabetic retinopathy	0.0260	0.0344	A
rs61785503	Ever smoked	0.0070	0.0012	T	Background diabetic retinopathy	-0.0210	0.0395	T
rs6265	Ever smoked	-0.0084	0.0013	T	Background diabetic retinopathy	0.0897	0.0452	T
rs6438208	Ever smoked	-0.0066	0.0012	A	Background diabetic retinopathy	0.0076	0.0370	A
rs6499595	Ever smoked	-0.0065	0.0010	C	Background diabetic retinopathy	-0.0267	0.0344	C
rs67716713	Ever smoked	-0.0073	0.0010	A	Background diabetic retinopathy	0.0352	0.0329	A

rs7014143	Ever smoked	0.0060	0.0011	C	Background diabetic retinopathy	0.0653	0.0338	C
rs7024687	Ever smoked	-0.0058	0.0010	G	Background diabetic retinopathy	-0.0464	0.0337	G
rs71580759	Ever smoked	-0.0068	0.0012	C	Background diabetic retinopathy	0.0186	0.0382	C
rs7162423	Ever smoked	-0.0056	0.0010	T	Background diabetic retinopathy	0.0231	0.0330	T
rs7216173	Ever smoked	-0.0072	0.0012	T	Background diabetic retinopathy	-0.0038	0.0434	T
rs72706955	Ever smoked	-0.0077	0.0014	T	Background diabetic retinopathy	-0.0801	0.0391	T
rs73058737	Ever smoked	0.0062	0.0011	T	Background diabetic retinopathy	-0.0368	0.0354	T
rs7572027	Ever smoked	0.0071	0.0013	T	Background diabetic retinopathy	-0.0002	0.0442	T
rs7585579	Ever smoked	0.0065	0.0010	G	Background diabetic retinopathy	0.0069	0.0329	G
rs75919030	Ever smoked	-0.0068	0.0011	C	Background diabetic retinopathy	0.0249	0.0374	C
rs763053	Ever smoked	-0.0084	0.0012	C	Background diabetic retinopathy	0.0029	0.0346	C
rs7758291	Ever smoked	0.0060	0.0011	C	Background diabetic retinopathy	0.0234	0.0364	C
rs77878475	Ever smoked	-0.0117	0.0019	A	Background diabetic retinopathy	-0.0396	0.0564	A
rs7870475	Ever smoked	0.0057	0.0010	C	Background diabetic retinopathy	-0.0190	0.0329	C
rs7901348	Ever smoked	-0.0060	0.0010	G	Background diabetic retinopathy	0.0096	0.0327	G
rs7969559	Ever smoked	-0.0062	0.0011	G	Background diabetic retinopathy	0.0080	0.0341	G
rs899632	Ever smoked	-0.0070	0.0010	C	Background diabetic retinopathy	0.0136	0.0343	C
rs904592	Ever smoked	0.0057	0.0010	T	Background diabetic retinopathy	-0.0221	0.0335	T
rs905871	Ever smoked	-0.0058	0.0011	G	Background diabetic retinopathy	-0.0114	0.0351	G
rs9375371	Ever smoked	0.0071	0.0011	A	Background diabetic retinopathy	0.0174	0.0350	A
rs9423279	Ever smoked	-0.0063	0.0011	G	Background diabetic retinopathy	0.0372	0.0337	G
rs9597810	Ever smoked	-0.0059	0.0011	G	Background diabetic retinopathy	0.0491	0.0350	G
rs9845144	Ever smoked	0.0066	0.0011	A	Background diabetic retinopathy	-0.1173	0.0435	A
rs10179482	Ever smoked	0.0058	0.0010	A	Diabetic retinopathy	0.0057	0.0132	A
rs10212155	Ever smoked	0.0104	0.0014	A	Diabetic retinopathy	-0.0154	0.0160	A
rs10233018	Ever smoked	0.0078	0.0010	G	Diabetic retinopathy	-0.0105	0.0134	G
rs1040070	Ever smoked	-0.0059	0.0010	C	Diabetic retinopathy	0.0065	0.0131	C
rs10774625	Ever smoked	-0.0066	0.0010	G	Diabetic retinopathy	-0.0481	0.0133	G
rs10863714	Ever smoked	-0.0058	0.0010	G	Diabetic retinopathy	-0.0004	0.0134	G
rs10952199	Ever smoked	-0.0069	0.0010	T	Diabetic retinopathy	-0.0269	0.0132	T
rs10956808	Ever smoked	-0.0067	0.0010	G	Diabetic retinopathy	-0.0025	0.0132	G

rs10988799	Ever smoked	0.0059	0.0010	T	Diabetic retinopathy	-0.0118	0.0131	T
rs11165623	Ever smoked	0.0055	0.0010	A	Diabetic retinopathy	-0.0042	0.0133	A
rs1124639	Ever smoked	0.0066	0.0010	C	Diabetic retinopathy	0.0206	0.0132	C
rs1150023	Ever smoked	-0.0065	0.0011	C	Diabetic retinopathy	-0.0259	0.0153	C
rs1174864	Ever smoked	0.0062	0.0010	A	Diabetic retinopathy	-0.0032	0.0134	A
rs12209519	Ever smoked	0.0057	0.0010	G	Diabetic retinopathy	-0.0009	0.0137	G
rs12244388	Ever smoked	0.0086	0.0011	A	Diabetic retinopathy	0.0035	0.0135	A
rs12272735	Ever smoked	0.0058	0.0010	G	Diabetic retinopathy	0.0090	0.0135	G
rs12333760	Ever smoked	-0.0083	0.0014	C	Diabetic retinopathy	0.0468	0.0156	C
rs12450028	Ever smoked	0.0061	0.0011	T	Diabetic retinopathy	-0.0227	0.0139	T
rs1246265	Ever smoked	0.0061	0.0011	C	Diabetic retinopathy	-0.0163	0.0146	C
rs12902636	Ever smoked	-0.0059	0.0010	T	Diabetic retinopathy	-0.0084	0.0134	T
rs13001103	Ever smoked	-0.0057	0.0010	G	Diabetic retinopathy	-0.0026	0.0131	G
rs13162305	Ever smoked	0.0060	0.0011	T	Diabetic retinopathy	-0.0201	0.0139	T
rs1322525	Ever smoked	0.0064	0.0010	G	Diabetic retinopathy	-0.0108	0.0147	G
rs1324481	Ever smoked	0.0069	0.0011	G	Diabetic retinopathy	0.0230	0.0143	G
rs13246563	Ever smoked	-0.0066	0.0010	G	Diabetic retinopathy	-0.0012	0.0135	G
rs13246563	Ever smoked	-0.0066	0.0010	G	Diabetic retinopathy	-0.0012	0.1240	G
rs13246563	Ever smoked	-0.0066	0.0010	G	Diabetic retinopathy	0.1871	0.0135	A
rs13246563	Ever smoked	-0.0066	0.0010	G	Diabetic retinopathy	0.1871	0.1240	A
rs1363101	Ever smoked	-0.0058	0.0010	G	Diabetic retinopathy	-0.0070	0.0132	G
rs1373178	Ever smoked	-0.0062	0.0010	G	Diabetic retinopathy	-0.0078	0.0136	G
rs150294	Ever smoked	-0.0076	0.0010	G	Diabetic retinopathy	0.0048	0.0132	G
rs1549212	Ever smoked	-0.0066	0.0010	T	Diabetic retinopathy	0.0075	0.0138	T
rs1718705	Ever smoked	0.0065	0.0011	C	Diabetic retinopathy	-0.0311	0.0144	C
rs17584022	Ever smoked	-0.0066	0.0011	A	Diabetic retinopathy	0.0177	0.0145	A
rs1876066	Ever smoked	-0.0057	0.0010	C	Diabetic retinopathy	-0.0107	0.0134	C
rs1899896	Ever smoked	0.0072	0.0011	T	Diabetic retinopathy	0.0169	0.0149	T
rs2155292	Ever smoked	0.0147	0.0010	G	Diabetic retinopathy	-0.0184	0.0137	G
rs2175207	Ever smoked	0.0076	0.0014	G	Diabetic retinopathy	-0.0048	0.0162	G
rs2183573	Ever smoked	-0.0058	0.0010	G	Diabetic retinopathy	-0.0077	0.0142	G

rs2516436	Ever smoked	-0.0057	0.0010	G	Diabetic retinopathy	-0.0500	0.0135	G
rs28809490	Ever smoked	-0.0063	0.0011	A	Diabetic retinopathy	-0.0019	0.0155	A
rs303753	Ever smoked	-0.0061	0.0011	A	Diabetic retinopathy	0.0038	0.0138	A
rs34335016	Ever smoked	-0.0086	0.0015	T	Diabetic retinopathy	0.0200	0.0174	T
rs35498642	Ever smoked	-0.0062	0.0010	T	Diabetic retinopathy	0.0006	0.0134	T
rs35892365	Ever smoked	-0.0067	0.0012	T	Diabetic retinopathy	0.0011	0.0137	T
rs3783177	Ever smoked	-0.0073	0.0012	G	Diabetic retinopathy	0.0076	0.0159	G
rs3790286	Ever smoked	0.0056	0.0010	C	Diabetic retinopathy	-0.0010	0.0133	C
rs41513151	Ever smoked	0.0073	0.0012	A	Diabetic retinopathy	0.0097	0.0164	A
rs4422110	Ever smoked	-0.0088	0.0010	T	Diabetic retinopathy	-0.0007	0.0133	T
rs465646	Ever smoked	-0.0117	0.0014	A	Diabetic retinopathy	0.0118	0.0165	A
rs4680392	Ever smoked	0.0062	0.0011	C	Diabetic retinopathy	0.0014	0.0142	C
rs4856598	Ever smoked	-0.0084	0.0010	C	Diabetic retinopathy	-0.0018	0.0146	C
rs528301	Ever smoked	0.0076	0.0010	A	Diabetic retinopathy	-0.0173	0.0137	A
rs529206	Ever smoked	-0.0059	0.0010	T	Diabetic retinopathy	-0.0226	0.0134	T
rs55864295	Ever smoked	-0.0074	0.0013	G	Diabetic retinopathy	-0.0066	0.0170	G
rs56166763	Ever smoked	-0.0058	0.0010	C	Diabetic retinopathy	-0.0149	0.0137	C
rs58400863	Ever smoked	-0.0063	0.0011	A	Diabetic retinopathy	0.0233	0.0138	A
rs61785503	Ever smoked	0.0070	0.0012	T	Diabetic retinopathy	-0.0081	0.0159	T
rs6265	Ever smoked	-0.0084	0.0013	T	Diabetic retinopathy	0.0025	0.0181	T
rs6438208	Ever smoked	-0.0066	0.0012	A	Diabetic retinopathy	0.0121	0.0149	A
rs6499595	Ever smoked	-0.0065	0.0010	C	Diabetic retinopathy	0.0073	0.0138	C
rs67716713	Ever smoked	-0.0073	0.0010	A	Diabetic retinopathy	0.0188	0.0131	A
rs7014143	Ever smoked	0.0060	0.0011	C	Diabetic retinopathy	0.0163	0.0135	C
rs7024687	Ever smoked	-0.0058	0.0010	G	Diabetic retinopathy	0.0124	0.0135	G
rs71580759	Ever smoked	-0.0068	0.0012	C	Diabetic retinopathy	0.0206	0.0153	C
rs7162423	Ever smoked	-0.0056	0.0010	T	Diabetic retinopathy	-0.0063	0.0132	T
rs7216173	Ever smoked	-0.0072	0.0012	T	Diabetic retinopathy	-0.0314	0.0174	T
rs72706955	Ever smoked	-0.0077	0.0014	T	Diabetic retinopathy	-0.0233	0.0157	T
rs73058737	Ever smoked	0.0062	0.0011	T	Diabetic retinopathy	0.0213	0.0142	T
rs7572027	Ever smoked	0.0071	0.0013	T	Diabetic retinopathy	-0.0037	0.0178	T

rs7585579	Ever smoked	0.0065	0.0010	G	Diabetic retinopathy	-0.0008	0.0132	G
rs75919030	Ever smoked	-0.0068	0.0011	C	Diabetic retinopathy	0.0228	0.0150	C
rs763053	Ever smoked	-0.0084	0.0012	C	Diabetic retinopathy	0.0036	0.0138	C
rs7758291	Ever smoked	0.0060	0.0011	C	Diabetic retinopathy	0.0029	0.0146	C
rs77878475	Ever smoked	-0.0117	0.0019	A	Diabetic retinopathy	-0.0314	0.0226	A
rs7870475	Ever smoked	0.0057	0.0010	C	Diabetic retinopathy	-0.0221	0.0132	C
rs7901348	Ever smoked	-0.0060	0.0010	G	Diabetic retinopathy	0.0125	0.0132	G
rs7969559	Ever smoked	-0.0062	0.0011	G	Diabetic retinopathy	-0.0107	0.0137	G
rs899632	Ever smoked	-0.0070	0.0010	C	Diabetic retinopathy	0.0019	0.0138	C
rs904592	Ever smoked	0.0057	0.0010	T	Diabetic retinopathy	-0.0139	0.0135	T
rs905871	Ever smoked	-0.0058	0.0011	G	Diabetic retinopathy	0.0145	0.0141	G
rs9375371	Ever smoked	0.0071	0.0011	A	Diabetic retinopathy	-0.0032	0.0140	A
rs9423279	Ever smoked	-0.0063	0.0011	G	Diabetic retinopathy	0.0071	0.0135	G
rs9597810	Ever smoked	-0.0059	0.0011	G	Diabetic retinopathy	0.0174	0.0140	G
rs9845144	Ever smoked	0.0066	0.0011	A	Diabetic retinopathy	0.0120	0.0174	A
rs10179482	Ever smoked	0.0058	0.0010	A	Proliferative diabetic retinopathy	-0.0080	0.0165	A
rs10212155	Ever smoked	0.0104	0.0014	A	Proliferative diabetic retinopathy	-0.0121	0.0199	A
rs10233018	Ever smoked	0.0078	0.0010	G	Proliferative diabetic retinopathy	-0.0067	0.0167	G
rs1040070	Ever smoked	-0.0059	0.0010	C	Proliferative diabetic retinopathy	0.0063	0.0164	C
rs10774625	Ever smoked	-0.0066	0.0010	G	Proliferative diabetic retinopathy	-0.0621	0.0165	G
rs10863714	Ever smoked	-0.0058	0.0010	G	Proliferative diabetic retinopathy	0.0249	0.0168	G
rs10952199	Ever smoked	-0.0069	0.0010	T	Proliferative diabetic retinopathy	-0.0445	0.0165	T
rs10956808	Ever smoked	-0.0067	0.0010	G	Proliferative diabetic retinopathy	0.0024	0.0164	G
rs10988799	Ever smoked	0.0059	0.0010	T	Proliferative diabetic retinopathy	-0.0026	0.0164	T
rs11165623	Ever smoked	0.0055	0.0010	A	Proliferative diabetic retinopathy	-0.0037	0.0166	A
rs1124639	Ever smoked	0.0066	0.0010	C	Proliferative diabetic retinopathy	0.0130	0.0164	C
rs1150023	Ever smoked	-0.0065	0.0011	C	Proliferative diabetic retinopathy	-0.0330	0.0191	C
rs1174864	Ever smoked	0.0062	0.0010	A	Proliferative diabetic retinopathy	-0.0190	0.0167	A
rs12209519	Ever smoked	0.0057	0.0010	G	Proliferative diabetic retinopathy	0.0044	0.0171	G
rs12244388	Ever smoked	0.0086	0.0011	A	Proliferative diabetic retinopathy	-0.0027	0.0169	A
rs12272735	Ever smoked	0.0058	0.0010	G	Proliferative diabetic retinopathy	0.0095	0.0169	G

rs12333760	Ever smoked	-0.0083	0.0014	C	Proliferative diabetic retinopathy	0.0507	0.0195	C
rs12450028	Ever smoked	0.0061	0.0011	T	Proliferative diabetic retinopathy	-0.0197	0.0173	T
rs1246265	Ever smoked	0.0061	0.0011	C	Proliferative diabetic retinopathy	-0.0159	0.0183	C
rs12902636	Ever smoked	-0.0059	0.0010	T	Proliferative diabetic retinopathy	-0.0063	0.0168	T
rs13001103	Ever smoked	-0.0057	0.0010	G	Proliferative diabetic retinopathy	-0.0043	0.0164	G
rs13162305	Ever smoked	0.0060	0.0011	T	Proliferative diabetic retinopathy	-0.0143	0.0174	T
rs1322525	Ever smoked	0.0064	0.0010	G	Proliferative diabetic retinopathy	-0.0070	0.0183	G
rs1324481	Ever smoked	0.0069	0.0011	G	Proliferative diabetic retinopathy	0.0219	0.0178	G
rs13246563	Ever smoked	-0.0066	0.0010	G	Proliferative diabetic retinopathy	0.1950	0.1546	A
rs13246563	Ever smoked	-0.0066	0.0010	G	Proliferative diabetic retinopathy	0.1950	0.0169	A
rs13246563	Ever smoked	-0.0066	0.0010	G	Proliferative diabetic retinopathy	0.0064	0.1546	G
rs13246563	Ever smoked	-0.0066	0.0010	G	Proliferative diabetic retinopathy	0.0064	0.0169	G
rs1363101	Ever smoked	-0.0058	0.0010	G	Proliferative diabetic retinopathy	-0.0297	0.0165	G
rs1373178	Ever smoked	-0.0062	0.0010	G	Proliferative diabetic retinopathy	-0.0114	0.0170	G
rs150294	Ever smoked	-0.0076	0.0010	G	Proliferative diabetic retinopathy	0.0099	0.0165	G
rs1549212	Ever smoked	-0.0066	0.0010	T	Proliferative diabetic retinopathy	0.0147	0.0172	T
rs1718705	Ever smoked	0.0065	0.0011	C	Proliferative diabetic retinopathy	-0.0252	0.0179	C
rs17584022	Ever smoked	-0.0066	0.0011	A	Proliferative diabetic retinopathy	0.0133	0.0181	A
rs1876066	Ever smoked	-0.0057	0.0010	C	Proliferative diabetic retinopathy	-0.0175	0.0167	C
rs1899896	Ever smoked	0.0072	0.0011	T	Proliferative diabetic retinopathy	0.0191	0.0186	T
rs2155292	Ever smoked	0.0147	0.0010	G	Proliferative diabetic retinopathy	-0.0217	0.0171	G
rs2175207	Ever smoked	0.0076	0.0014	G	Proliferative diabetic retinopathy	-0.0256	0.0202	G
rs2183573	Ever smoked	-0.0058	0.0010	G	Proliferative diabetic retinopathy	0.0019	0.0177	G
rs2516436	Ever smoked	-0.0057	0.0010	G	Proliferative diabetic retinopathy	-0.0531	0.0168	G
rs28809490	Ever smoked	-0.0063	0.0011	A	Proliferative diabetic retinopathy	-0.0099	0.0194	A
rs303753	Ever smoked	-0.0061	0.0011	A	Proliferative diabetic retinopathy	0.0026	0.0172	A
rs34335016	Ever smoked	-0.0086	0.0015	T	Proliferative diabetic retinopathy	0.0157	0.0217	T
rs35498642	Ever smoked	-0.0062	0.0010	T	Proliferative diabetic retinopathy	0.0101	0.0167	T
rs35892365	Ever smoked	-0.0067	0.0012	T	Proliferative diabetic retinopathy	-0.0050	0.0171	T
rs3783177	Ever smoked	-0.0073	0.0012	G	Proliferative diabetic retinopathy	0.0460	0.0198	G
rs3790286	Ever smoked	0.0056	0.0010	C	Proliferative diabetic retinopathy	-0.0002	0.0166	C

rs41513151	Ever smoked	0.0073	0.0012	A	Proliferative diabetic retinopathy	0.0288	0.0204	A
rs4422110	Ever smoked	-0.0088	0.0010	T	Proliferative diabetic retinopathy	0.0179	0.0165	T
rs465646	Ever smoked	-0.0117	0.0014	A	Proliferative diabetic retinopathy	0.0028	0.0206	A
rs4680392	Ever smoked	0.0062	0.0011	C	Proliferative diabetic retinopathy	0.0008	0.0177	C
rs4856598	Ever smoked	-0.0084	0.0010	C	Proliferative diabetic retinopathy	0.0011	0.0182	C
rs528301	Ever smoked	0.0076	0.0010	A	Proliferative diabetic retinopathy	-0.0099	0.0171	A
rs529206	Ever smoked	-0.0059	0.0010	T	Proliferative diabetic retinopathy	-0.0274	0.0167	T
rs55864295	Ever smoked	-0.0074	0.0013	G	Proliferative diabetic retinopathy	0.0150	0.0211	G
rs56166763	Ever smoked	-0.0058	0.0010	C	Proliferative diabetic retinopathy	-0.0109	0.0171	C
rs58400863	Ever smoked	-0.0063	0.0011	A	Proliferative diabetic retinopathy	0.0355	0.0172	A
rs61785503	Ever smoked	0.0070	0.0012	T	Proliferative diabetic retinopathy	-0.0324	0.0198	T
rs6265	Ever smoked	-0.0084	0.0013	T	Proliferative diabetic retinopathy	0.0301	0.0226	T
rs6438208	Ever smoked	-0.0066	0.0012	A	Proliferative diabetic retinopathy	0.0094	0.0185	A
rs6499595	Ever smoked	-0.0065	0.0010	C	Proliferative diabetic retinopathy	-0.0084	0.0172	C
rs67716713	Ever smoked	-0.0073	0.0010	A	Proliferative diabetic retinopathy	0.0108	0.0164	A
rs7014143	Ever smoked	0.0060	0.0011	C	Proliferative diabetic retinopathy	0.0149	0.0169	C
rs7024687	Ever smoked	-0.0058	0.0010	G	Proliferative diabetic retinopathy	0.0120	0.0169	G
rs71580759	Ever smoked	-0.0068	0.0012	C	Proliferative diabetic retinopathy	0.0154	0.0191	C
rs7162423	Ever smoked	-0.0056	0.0010	T	Proliferative diabetic retinopathy	-0.0017	0.0165	T
rs7216173	Ever smoked	-0.0072	0.0012	T	Proliferative diabetic retinopathy	-0.0373	0.0217	T
rs72706955	Ever smoked	-0.0077	0.0014	T	Proliferative diabetic retinopathy	-0.0267	0.0195	T
rs73058737	Ever smoked	0.0062	0.0011	T	Proliferative diabetic retinopathy	0.0078	0.0177	T
rs7572027	Ever smoked	0.0071	0.0013	T	Proliferative diabetic retinopathy	0.0089	0.0221	T
rs7585579	Ever smoked	0.0065	0.0010	G	Proliferative diabetic retinopathy	0.0099	0.0165	G
rs75919030	Ever smoked	-0.0068	0.0011	C	Proliferative diabetic retinopathy	0.0450	0.0187	C
rs763053	Ever smoked	-0.0084	0.0012	C	Proliferative diabetic retinopathy	0.0116	0.0173	C
rs7758291	Ever smoked	0.0060	0.0011	C	Proliferative diabetic retinopathy	0.0137	0.0182	C
rs77878475	Ever smoked	-0.0117	0.0019	A	Proliferative diabetic retinopathy	-0.0488	0.0282	A
rs7870475	Ever smoked	0.0057	0.0010	C	Proliferative diabetic retinopathy	-0.0136	0.0165	C
rs7901348	Ever smoked	-0.0060	0.0010	G	Proliferative diabetic retinopathy	0.0138	0.0164	G
rs7969559	Ever smoked	-0.0062	0.0011	G	Proliferative diabetic retinopathy	-0.0188	0.0171	G

rs899632	Ever smoked	-0.0070	0.0010	C	Proliferative diabetic retinopathy	0.0031	0.0172	C
rs904592	Ever smoked	0.0057	0.0010	T	Proliferative diabetic retinopathy	-0.0173	0.0168	T
rs905871	Ever smoked	-0.0058	0.0011	G	Proliferative diabetic retinopathy	0.0102	0.0176	G
rs9375371	Ever smoked	0.0071	0.0011	A	Proliferative diabetic retinopathy	-0.0027	0.0175	A
rs9423279	Ever smoked	-0.0063	0.0011	G	Proliferative diabetic retinopathy	0.0112	0.0169	G
rs9597810	Ever smoked	-0.0059	0.0011	G	Proliferative diabetic retinopathy	0.0167	0.0175	G
rs9845144	Ever smoked	0.0066	0.0011	A	Proliferative diabetic retinopathy	0.0043	0.0217	A
rs10123368	Hip circumference	0.0260	0.0044	C	Background diabetic retinopathy	-0.0263	0.0444	C
rs10140922	Hip circumference	-0.0300	0.0046	T	Background diabetic retinopathy	-0.0494	0.0330	T
rs10195252	Hip circumference	0.0230	0.0036	C	Background diabetic retinopathy	-0.0156	0.0341	C
rs1046934	Hip circumference	0.0230	0.0037	C	Background diabetic retinopathy	0.0470	0.0333	C
rs1053593	Hip circumference	0.0210	0.0038	T	Background diabetic retinopathy	-0.0124	0.0334	T
rs10748128	Hip circumference	0.0230	0.0040	T	Background diabetic retinopathy	0.0073	0.0343	T
rs10804591	Hip circumference	-0.0380	0.0044	A	Background diabetic retinopathy	0.0190	0.0411	A
rs10950949	Hip circumference	-0.0210	0.0038	C	Background diabetic retinopathy	0.0347	0.0355	C
rs10958476	Hip circumference	0.0280	0.0046	C	Background diabetic retinopathy	0.0378	0.0389	C
rs11205303	Hip circumference	0.0410	0.0040	C	Background diabetic retinopathy	-0.0241	0.0353	C
rs11242	Hip circumference	-0.0270	0.0036	C	Background diabetic retinopathy	0.0236	0.0332	C
rs11612228	Hip circumference	0.0230	0.0041	T	Background diabetic retinopathy	0.0225	0.0365	T
rs1173771	Hip circumference	-0.0260	0.0036	G	Background diabetic retinopathy	0.0608	0.0332	G
rs12086130	Hip circumference	0.0370	0.0063	T	Background diabetic retinopathy	0.0346	0.0717	T
rs12207675	Hip circumference	0.0410	0.0055	C	Background diabetic retinopathy	-0.0891	0.0480	C
rs1254257	Hip circumference	0.0290	0.0048	C	Background diabetic retinopathy	0.0344	0.0408	C
rs12817549	Hip circumference	-0.0290	0.0036	C	Background diabetic retinopathy	0.0241	0.0329	C
rs1294410	Hip circumference	-0.0290	0.0037	C	Background diabetic retinopathy	-0.0156	0.0342	C
rs12980348	Hip circumference	0.0290	0.0036	G	Background diabetic retinopathy	-0.0299	0.0344	G
rs1351394	Hip circumference	-0.0250	0.0035	C	Background diabetic retinopathy	0.0031	0.0329	C
rs1388251	Hip circumference	-0.0230	0.0040	G	Background diabetic retinopathy	0.0550	0.0360	G
rs143384	Hip circumference	0.0440	0.0038	G	Background diabetic retinopathy	0.0279	0.0331	G
rs1545552	Hip circumference	0.0290	0.0040	G	Background diabetic retinopathy	-0.0181	0.0386	G
rs1662837	Hip circumference	0.0280	0.0038	C	Background diabetic retinopathy	0.0043	0.0342	C

rs16894959	Hip circumference	0.0360	0.0050	C	Background diabetic retinopathy	-0.0003	0.0416	C
rs169797	Hip circumference	0.0240	0.0040	A	Background diabetic retinopathy	-0.0583	0.0351	A
rs17193922	Hip circumference	0.0240	0.0040	G	Background diabetic retinopathy	-0.0247	0.0374	G
rs1727294	Hip circumference	-0.0320	0.0043	G	Background diabetic retinopathy	0.0098	0.0422	G
rs17346473	Hip circumference	0.0300	0.0040	G	Background diabetic retinopathy	-0.0221	0.0388	G
rs17819328	Hip circumference	-0.0230	0.0037	G	Background diabetic retinopathy	0.0588	0.0330	G
rs1812175	Hip circumference	0.0590	0.0048	G	Background diabetic retinopathy	-0.0663	0.0440	G
rs181553	Hip circumference	0.0290	0.0038	A	Background diabetic retinopathy	-0.0269	0.0341	A
rs2034088	Hip circumference	-0.0210	0.0036	C	Background diabetic retinopathy	0.0379	0.0331	C
rs2098771	Hip circumference	-0.0220	0.0041	A	Background diabetic retinopathy	-0.0327	0.0341	A
rs2247341	Hip circumference	0.0230	0.0037	A	Background diabetic retinopathy	-0.0012	0.0330	A
rs2326788	Hip circumference	-0.0220	0.0036	A	Background diabetic retinopathy	0.0206	0.0330	A
rs2377058	Hip circumference	0.0230	0.0038	G	Background diabetic retinopathy	-0.0394	0.0359	G
rs2597513	Hip circumference	-0.0330	0.0059	T	Background diabetic retinopathy	-0.0137	0.0584	T
rs2638953	Hip circumference	0.0240	0.0038	C	Background diabetic retinopathy	0.0085	0.0356	C
rs2820443	Hip circumference	0.0480	0.0039	C	Background diabetic retinopathy	-0.0076	0.0359	C
rs3747579	Hip circumference	0.0230	0.0040	T	Background diabetic retinopathy	-0.0311	0.0361	T
rs3748656	Hip circumference	-0.0240	0.0042	T	Background diabetic retinopathy	0.0243	0.0342	T
rs3791679	Hip circumference	-0.0380	0.0041	G	Background diabetic retinopathy	0.0435	0.0392	G
rs42235	Hip circumference	0.0360	0.0039	T	Background diabetic retinopathy	-0.0151	0.0363	T
rs4243400	Hip circumference	0.0250	0.0036	G	Background diabetic retinopathy	0.0003	0.0328	G
rs4246307	Hip circumference	-0.0240	0.0043	A	Background diabetic retinopathy	0.0132	0.0329	A
rs4369779	Hip circumference	0.0350	0.0044	C	Background diabetic retinopathy	-0.0432	0.0392	C
rs473902	Hip circumference	-0.0580	0.0075	G	Background diabetic retinopathy	0.0470	0.0542	G
rs4973517	Hip circumference	-0.0290	0.0045	C	Background diabetic retinopathy	-0.0024	0.0406	C
rs558003	Hip circumference	0.0490	0.0063	A	Background diabetic retinopathy	-0.0071	0.0622	A
rs561341	Hip circumference	0.0310	0.0050	G	Background diabetic retinopathy	-0.0414	0.0490	G
rs606452	Hip circumference	-0.0290	0.0051	C	Background diabetic retinopathy	0.0508	0.0427	C
rs6470764	Hip circumference	-0.0390	0.0045	T	Background diabetic retinopathy	-0.0542	0.0431	T
rs6501392	Hip circumference	0.0210	0.0035	G	Background diabetic retinopathy	0.0100	0.0337	G
rs6556079	Hip circumference	-0.0280	0.0045	A	Background diabetic retinopathy	-0.0850	0.0336	A

rs6570509	Hip circumference	-0.0450	0.0040	T	Background diabetic retinopathy	0.0084	0.0373	T
rs6657613	Hip circumference	0.0310	0.0036	T	Background diabetic retinopathy	0.0272	0.0328	T
rs6739772	Hip circumference	0.0220	0.0039	G	Background diabetic retinopathy	0.0244	0.0373	G
rs686320	Hip circumference	-0.0380	0.0055	C	Background diabetic retinopathy	0.0384	0.0547	C
rs7008867	Hip circumference	0.0240	0.0041	A	Background diabetic retinopathy	-0.0437	0.0352	A
rs7162542	Hip circumference	0.0410	0.0036	G	Background diabetic retinopathy	-0.0159	0.0330	G
rs7187776	Hip circumference	0.0200	0.0036	G	Background diabetic retinopathy	0.0085	0.0330	G
rs7223966	Hip circumference	0.0290	0.0039	A	Background diabetic retinopathy	-0.0660	0.0390	A
rs724016	Hip circumference	0.0480	0.0035	G	Background diabetic retinopathy	-0.0430	0.0329	G
rs7759938	Hip circumference	-0.0280	0.0038	T	Background diabetic retinopathy	-0.0393	0.0353	T
rs798497	Hip circumference	-0.0350	0.0038	G	Background diabetic retinopathy	0.0426	0.0340	G
rs806794	Hip circumference	-0.0460	0.0040	G	Background diabetic retinopathy	-0.0338	0.0339	G
rs849141	Hip circumference	-0.0320	0.0039	G	Background diabetic retinopathy	0.0064	0.0354	G
rs894345	Hip circumference	-0.0200	0.0036	T	Background diabetic retinopathy	-0.0258	0.0329	T
rs9388766	Hip circumference	-0.0280	0.0039	C	Background diabetic retinopathy	-0.0273	0.0375	C
rs978332	Hip circumference	-0.0260	0.0039	T	Background diabetic retinopathy	-0.0033	0.0341	T
rs9890032	Hip circumference	-0.0260	0.0037	G	Background diabetic retinopathy	0.0131	0.0338	G
rs991967	Hip circumference	0.0220	0.0038	C	Background diabetic retinopathy	-0.0176	0.0349	C
rs9993613	Hip circumference	-0.0270	0.0045	G	Background diabetic retinopathy	-0.0032	0.0328	G
rs10123368	Hip circumference	0.0260	0.0044	C	Diabetic retinopathy	-0.0101	0.0177	C
rs10140922	Hip circumference	-0.0300	0.0046	T	Diabetic retinopathy	0.0135	0.0132	T
rs10195252	Hip circumference	0.0230	0.0036	C	Diabetic retinopathy	-0.0211	0.0137	C
rs1046934	Hip circumference	0.0230	0.0037	C	Diabetic retinopathy	-0.0048	0.0134	C
rs1053593	Hip circumference	0.0210	0.0038	T	Diabetic retinopathy	0.0047	0.0134	T
rs10748128	Hip circumference	0.0230	0.0040	T	Diabetic retinopathy	-0.0096	0.0138	T
rs10804591	Hip circumference	-0.0380	0.0044	A	Diabetic retinopathy	0.0061	0.0165	A
rs10950949	Hip circumference	-0.0210	0.0038	C	Diabetic retinopathy	0.0073	0.0143	C
rs10958476	Hip circumference	0.0280	0.0046	C	Diabetic retinopathy	0.0128	0.0156	C
rs11205303	Hip circumference	0.0410	0.0040	C	Diabetic retinopathy	0.0044	0.0141	C
rs11242	Hip circumference	-0.0270	0.0036	C	Diabetic retinopathy	0.0140	0.0133	C
rs11612228	Hip circumference	0.0230	0.0041	T	Diabetic retinopathy	0.0132	0.0146	T

rs1173771	Hip circumference	-0.0260	0.0036	G	Diabetic retinopathy	0.0121	0.0133	G
rs12086130	Hip circumference	0.0370	0.0063	T	Diabetic retinopathy	0.0263	0.0285	T
rs12207675	Hip circumference	0.0410	0.0055	C	Diabetic retinopathy	-0.0311	0.0192	C
rs1254257	Hip circumference	0.0290	0.0048	C	Diabetic retinopathy	0.0178	0.0163	C
rs12817549	Hip circumference	-0.0290	0.0036	C	Diabetic retinopathy	0.0065	0.0132	C
rs1294410	Hip circumference	-0.0290	0.0037	C	Diabetic retinopathy	-0.0018	0.0137	C
rs12980348	Hip circumference	0.0290	0.0036	G	Diabetic retinopathy	-0.0095	0.0138	G
rs1351394	Hip circumference	-0.0250	0.0035	C	Diabetic retinopathy	0.0139	0.0132	C
rs1388251	Hip circumference	-0.0230	0.0040	G	Diabetic retinopathy	0.0228	0.0144	G
rs143384	Hip circumference	0.0440	0.0038	G	Diabetic retinopathy	0.0170	0.0133	G
rs1545552	Hip circumference	0.0290	0.0040	G	Diabetic retinopathy	0.0075	0.0155	G
rs1662837	Hip circumference	0.0280	0.0038	C	Diabetic retinopathy	0.0069	0.0137	C
rs16894959	Hip circumference	0.0360	0.0050	C	Diabetic retinopathy	0.0235	0.0166	C
rs169797	Hip circumference	0.0240	0.0040	A	Diabetic retinopathy	-0.0175	0.0141	A
rs17193922	Hip circumference	0.0240	0.0040	G	Diabetic retinopathy	0.0073	0.0150	G
rs1727294	Hip circumference	-0.0320	0.0043	G	Diabetic retinopathy	0.0190	0.0170	G
rs17346473	Hip circumference	0.0300	0.0040	G	Diabetic retinopathy	0.0162	0.0155	G
rs17819328	Hip circumference	-0.0230	0.0037	G	Diabetic retinopathy	0.0085	0.0132	G
rs1812175	Hip circumference	0.0590	0.0048	G	Diabetic retinopathy	-0.0015	0.0177	G
rs181553	Hip circumference	0.0290	0.0038	A	Diabetic retinopathy	-0.0173	0.0136	A
rs2034088	Hip circumference	-0.0210	0.0036	C	Diabetic retinopathy	0.0108	0.0133	C
rs2098771	Hip circumference	-0.0220	0.0041	A	Diabetic retinopathy	-0.0047	0.0137	A
rs2247341	Hip circumference	0.0230	0.0037	A	Diabetic retinopathy	0.0058	0.0132	A
rs2326788	Hip circumference	-0.0220	0.0036	A	Diabetic retinopathy	-0.0211	0.0132	A
rs2377058	Hip circumference	0.0230	0.0038	G	Diabetic retinopathy	0.0018	0.0144	G
rs2597513	Hip circumference	-0.0330	0.0059	T	Diabetic retinopathy	-0.0045	0.0234	T
rs2638953	Hip circumference	0.0240	0.0038	C	Diabetic retinopathy	-0.0396	0.0143	C
rs2820443	Hip circumference	0.0480	0.0039	C	Diabetic retinopathy	-0.0344	0.0144	C
rs3747579	Hip circumference	0.0230	0.0040	T	Diabetic retinopathy	-0.0238	0.0145	T
rs3748656	Hip circumference	-0.0240	0.0042	T	Diabetic retinopathy	0.0297	0.0137	T
rs3791679	Hip circumference	-0.0380	0.0041	G	Diabetic retinopathy	0.0128	0.0157	G

rs42235	Hip circumference	0.0360	0.0039	T	Diabetic retinopathy	-0.0179	0.0145	T
rs4243400	Hip circumference	0.0250	0.0036	G	Diabetic retinopathy	-0.0032	0.0132	G
rs4246307	Hip circumference	-0.0240	0.0043	A	Diabetic retinopathy	0.0047	0.0132	A
rs4369779	Hip circumference	0.0350	0.0044	C	Diabetic retinopathy	-0.0094	0.0157	C
rs473902	Hip circumference	-0.0580	0.0075	G	Diabetic retinopathy	0.0093	0.0221	G
rs4973517	Hip circumference	-0.0290	0.0045	C	Diabetic retinopathy	0.0133	0.0164	C
rs558003	Hip circumference	0.0490	0.0063	A	Diabetic retinopathy	-0.0036	0.0250	A
rs561341	Hip circumference	0.0310	0.0050	G	Diabetic retinopathy	-0.0256	0.0196	G
rs606452	Hip circumference	-0.0290	0.0051	C	Diabetic retinopathy	-0.0074	0.0171	C
rs6470764	Hip circumference	-0.0390	0.0045	T	Diabetic retinopathy	0.0270	0.0173	T
rs6501392	Hip circumference	0.0210	0.0035	G	Diabetic retinopathy	-0.0180	0.0135	G
rs6556079	Hip circumference	-0.0280	0.0045	A	Diabetic retinopathy	-0.0188	0.0135	A
rs6570509	Hip circumference	-0.0450	0.0040	T	Diabetic retinopathy	-0.0165	0.0150	T
rs6657613	Hip circumference	0.0310	0.0036	T	Diabetic retinopathy	0.0017	0.0132	T
rs6739772	Hip circumference	0.0220	0.0039	G	Diabetic retinopathy	-0.0075	0.0149	G
rs686320	Hip circumference	-0.0380	0.0055	C	Diabetic retinopathy	0.0149	0.0220	C
rs7008867	Hip circumference	0.0240	0.0041	A	Diabetic retinopathy	0.0228	0.0141	A
rs7162542	Hip circumference	0.0410	0.0036	G	Diabetic retinopathy	0.0061	0.0132	G
rs7187776	Hip circumference	0.0200	0.0036	G	Diabetic retinopathy	-0.0115	0.0132	G
rs7223966	Hip circumference	0.0290	0.0039	A	Diabetic retinopathy	-0.0067	0.0156	A
rs724016	Hip circumference	0.0480	0.0035	G	Diabetic retinopathy	-0.0207	0.0132	G
rs7759938	Hip circumference	-0.0280	0.0038	T	Diabetic retinopathy	-0.0101	0.0142	T
rs798497	Hip circumference	-0.0350	0.0038	G	Diabetic retinopathy	0.0194	0.0137	G
rs806794	Hip circumference	-0.0460	0.0040	G	Diabetic retinopathy	-0.0040	0.0135	G
rs849141	Hip circumference	-0.0320	0.0039	G	Diabetic retinopathy	0.0008	0.0142	G
rs894345	Hip circumference	-0.0200	0.0036	T	Diabetic retinopathy	0.0021	0.0132	T
rs9388766	Hip circumference	-0.0280	0.0039	C	Diabetic retinopathy	0.0290	0.0150	C
rs978332	Hip circumference	-0.0260	0.0039	T	Diabetic retinopathy	-0.0125	0.0137	T
rs9890032	Hip circumference	-0.0260	0.0037	G	Diabetic retinopathy	-0.0039	0.0135	G
rs991967	Hip circumference	0.0220	0.0038	C	Diabetic retinopathy	0.0118	0.0140	C
rs9993613	Hip circumference	-0.0270	0.0045	G	Diabetic retinopathy	0.0162	0.0131	G

rs10123368	Hip circumference	0.0260	0.0044	C	Proliferative diabetic retinopathy	-0.0044	0.0222	C
rs10140922	Hip circumference	-0.0300	0.0046	T	Proliferative diabetic retinopathy	-0.0014	0.0165	T
rs10195252	Hip circumference	0.0230	0.0036	C	Proliferative diabetic retinopathy	-0.0221	0.0171	C
rs1046934	Hip circumference	0.0230	0.0037	C	Proliferative diabetic retinopathy	-0.0006	0.0167	C
rs1053593	Hip circumference	0.0210	0.0038	T	Proliferative diabetic retinopathy	-0.0005	0.0167	T
rs10748128	Hip circumference	0.0230	0.0040	T	Proliferative diabetic retinopathy	-0.0003	0.0172	T
rs10804591	Hip circumference	-0.0380	0.0044	A	Proliferative diabetic retinopathy	0.0063	0.0206	A
rs10950949	Hip circumference	-0.0210	0.0038	C	Proliferative diabetic retinopathy	0.0029	0.0178	C
rs10958476	Hip circumference	0.0280	0.0046	C	Proliferative diabetic retinopathy	0.0127	0.0194	C
rs11205303	Hip circumference	0.0410	0.0040	C	Proliferative diabetic retinopathy	0.0030	0.0176	C
rs11242	Hip circumference	-0.0270	0.0036	C	Proliferative diabetic retinopathy	0.0185	0.0166	C
rs11612228	Hip circumference	0.0230	0.0041	T	Proliferative diabetic retinopathy	0.0068	0.0182	T
rs1173771	Hip circumference	-0.0260	0.0036	G	Proliferative diabetic retinopathy	-0.0026	0.0166	G
rs12086130	Hip circumference	0.0370	0.0063	T	Proliferative diabetic retinopathy	0.0301	0.0357	T
rs12207675	Hip circumference	0.0410	0.0055	C	Proliferative diabetic retinopathy	-0.0409	0.0240	C
rs1254257	Hip circumference	0.0290	0.0048	C	Proliferative diabetic retinopathy	0.0045	0.0204	C
rs12817549	Hip circumference	-0.0290	0.0036	C	Proliferative diabetic retinopathy	0.0027	0.0165	C
rs1294410	Hip circumference	-0.0290	0.0037	C	Proliferative diabetic retinopathy	0.0118	0.0171	C
rs12980348	Hip circumference	0.0290	0.0036	G	Proliferative diabetic retinopathy	-0.0164	0.0172	G
rs1351394	Hip circumference	-0.0250	0.0035	C	Proliferative diabetic retinopathy	0.0044	0.0164	C
rs1388251	Hip circumference	-0.0230	0.0040	G	Proliferative diabetic retinopathy	0.0167	0.0180	G
rs143384	Hip circumference	0.0440	0.0038	G	Proliferative diabetic retinopathy	0.0141	0.0166	G
rs1545552	Hip circumference	0.0290	0.0040	G	Proliferative diabetic retinopathy	0.0069	0.0193	G
rs1662837	Hip circumference	0.0280	0.0038	C	Proliferative diabetic retinopathy	0.0205	0.0171	C
rs16894959	Hip circumference	0.0360	0.0050	C	Proliferative diabetic retinopathy	0.0234	0.0208	C
rs169797	Hip circumference	0.0240	0.0040	A	Proliferative diabetic retinopathy	-0.0282	0.0176	A
rs17193922	Hip circumference	0.0240	0.0040	G	Proliferative diabetic retinopathy	-0.0108	0.0187	G
rs1727294	Hip circumference	-0.0320	0.0043	G	Proliferative diabetic retinopathy	0.0069	0.0212	G
rs17346473	Hip circumference	0.0300	0.0040	G	Proliferative diabetic retinopathy	0.0025	0.0194	G
rs17819328	Hip circumference	-0.0230	0.0037	G	Proliferative diabetic retinopathy	0.0340	0.0165	G
rs1812175	Hip circumference	0.0590	0.0048	G	Proliferative diabetic retinopathy	-0.0044	0.0221	G

rs181553	Hip circumference	0.0290	0.0038	A	Proliferative diabetic retinopathy	-0.0128	0.0170	A
rs2034088	Hip circumference	-0.0210	0.0036	C	Proliferative diabetic retinopathy	-0.0022	0.0166	C
rs2098771	Hip circumference	-0.0220	0.0041	A	Proliferative diabetic retinopathy	-0.0025	0.0170	A
rs2247341	Hip circumference	0.0230	0.0037	A	Proliferative diabetic retinopathy	0.0063	0.0165	A
rs2326788	Hip circumference	-0.0220	0.0036	A	Proliferative diabetic retinopathy	0.0006	0.0165	A
rs2377058	Hip circumference	0.0230	0.0038	G	Proliferative diabetic retinopathy	-0.0128	0.0179	G
rs2597513	Hip circumference	-0.0330	0.0059	T	Proliferative diabetic retinopathy	0.0225	0.0292	T
rs2638953	Hip circumference	0.0240	0.0038	C	Proliferative diabetic retinopathy	-0.0265	0.0178	C
rs2820443	Hip circumference	0.0480	0.0039	C	Proliferative diabetic retinopathy	-0.0274	0.0180	C
rs3747579	Hip circumference	0.0230	0.0040	T	Proliferative diabetic retinopathy	-0.0260	0.0181	T
rs3748656	Hip circumference	-0.0240	0.0042	T	Proliferative diabetic retinopathy	0.0261	0.0171	T
rs3791679	Hip circumference	-0.0380	0.0041	G	Proliferative diabetic retinopathy	0.0218	0.0196	G
rs42235	Hip circumference	0.0360	0.0039	T	Proliferative diabetic retinopathy	-0.0241	0.0182	T
rs4243400	Hip circumference	0.0250	0.0036	G	Proliferative diabetic retinopathy	0.0036	0.0164	G
rs4246307	Hip circumference	-0.0240	0.0043	A	Proliferative diabetic retinopathy	-0.0049	0.0164	A
rs4369779	Hip circumference	0.0350	0.0044	C	Proliferative diabetic retinopathy	-0.0154	0.0196	C
rs473902	Hip circumference	-0.0580	0.0075	G	Proliferative diabetic retinopathy	0.0022	0.0273	G
rs4973517	Hip circumference	-0.0290	0.0045	C	Proliferative diabetic retinopathy	0.0103	0.0204	C
rs558003	Hip circumference	0.0490	0.0063	A	Proliferative diabetic retinopathy	-0.0397	0.0312	A
rs561341	Hip circumference	0.0310	0.0050	G	Proliferative diabetic retinopathy	-0.0353	0.0245	G
rs606452	Hip circumference	-0.0290	0.0051	C	Proliferative diabetic retinopathy	-0.0027	0.0214	C
rs6470764	Hip circumference	-0.0390	0.0045	T	Proliferative diabetic retinopathy	0.0149	0.0215	T
rs6501392	Hip circumference	0.0210	0.0035	G	Proliferative diabetic retinopathy	-0.0011	0.0168	G
rs6556079	Hip circumference	-0.0280	0.0045	A	Proliferative diabetic retinopathy	-0.0282	0.0168	A
rs6570509	Hip circumference	-0.0450	0.0040	T	Proliferative diabetic retinopathy	-0.0104	0.0187	T
rs6657613	Hip circumference	0.0310	0.0036	T	Proliferative diabetic retinopathy	0.0176	0.0164	T
rs6739772	Hip circumference	0.0220	0.0039	G	Proliferative diabetic retinopathy	-0.0062	0.0186	G
rs686320	Hip circumference	-0.0380	0.0055	C	Proliferative diabetic retinopathy	0.0225	0.0274	C
rs7008867	Hip circumference	0.0240	0.0041	A	Proliferative diabetic retinopathy	-0.0055	0.0176	A
rs7162542	Hip circumference	0.0410	0.0036	G	Proliferative diabetic retinopathy	0.0133	0.0165	G
rs7187776	Hip circumference	0.0200	0.0036	G	Proliferative diabetic retinopathy	-0.0158	0.0165	G

rs7223966	Hip circumference	0.0290	0.0039	A	Proliferative diabetic retinopathy	-0.0210	0.0195	A
rs724016	Hip circumference	0.0480	0.0035	G	Proliferative diabetic retinopathy	-0.0287	0.0165	G
rs7759938	Hip circumference	-0.0280	0.0038	T	Proliferative diabetic retinopathy	-0.0328	0.0177	T
rs798497	Hip circumference	-0.0350	0.0038	G	Proliferative diabetic retinopathy	-0.0024	0.0170	G
rs806794	Hip circumference	-0.0460	0.0040	G	Proliferative diabetic retinopathy	-0.0223	0.0169	G
rs849141	Hip circumference	-0.0320	0.0039	G	Proliferative diabetic retinopathy	0.0207	0.0178	G
rs894345	Hip circumference	-0.0200	0.0036	T	Proliferative diabetic retinopathy	0.0062	0.0164	T
rs9388766	Hip circumference	-0.0280	0.0039	C	Proliferative diabetic retinopathy	-0.0001	0.0187	C
rs978332	Hip circumference	-0.0260	0.0039	T	Proliferative diabetic retinopathy	-0.0167	0.0171	T
rs9890032	Hip circumference	-0.0260	0.0037	G	Proliferative diabetic retinopathy	-0.0129	0.0169	G
rs991967	Hip circumference	0.0220	0.0038	C	Proliferative diabetic retinopathy	0.0110	0.0175	C
rs9993613	Hip circumference	-0.0270	0.0045	G	Proliferative diabetic retinopathy	0.0095	0.0164	G
rs10041657	Waist circumference	0.0250	0.0040	A	Background diabetic retinopathy	0.0429	0.0413	A
rs10748826	Waist circumference	-0.0230	0.0037	C	Background diabetic retinopathy	-0.0299	0.0330	C
rs11144688	Waist circumference	-0.0340	0.0060	A	Background diabetic retinopathy	-0.0463	0.0469	A
rs11205277	Waist circumference	0.0270	0.0036	G	Background diabetic retinopathy	-0.0224	0.0349	G
rs12207675	Waist circumference	0.0310	0.0052	C	Background diabetic retinopathy	-0.0891	0.0480	C
rs12317176	Waist circumference	-0.0200	0.0035	C	Background diabetic retinopathy	-0.0156	0.0358	C
rs12330322	Waist circumference	-0.0220	0.0040	T	Background diabetic retinopathy	-0.0686	0.0414	T
rs12493901	Waist circumference	-0.0210	0.0034	A	Background diabetic retinopathy	0.0050	0.0328	A
rs12608504	Waist circumference	-0.0200	0.0036	G	Background diabetic retinopathy	0.0440	0.0347	G
rs12656497	Waist circumference	-0.0220	0.0034	C	Background diabetic retinopathy	0.0617	0.0332	C
rs12679556	Waist circumference	0.0260	0.0039	G	Background diabetic retinopathy	0.0201	0.0383	G
rs12700664	Waist circumference	-0.0190	0.0034	T	Background diabetic retinopathy	-0.0132	0.0327	T
rs12991495	Waist circumference	-0.0280	0.0037	C	Background diabetic retinopathy	0.0004	0.0369	C
rs13083798	Waist circumference	-0.0200	0.0034	G	Background diabetic retinopathy	-0.0623	0.0328	G
rs13210323	Waist circumference	-0.0220	0.0038	C	Background diabetic retinopathy	-0.0370	0.0409	C
rs1344674	Waist circumference	0.0240	0.0033	G	Background diabetic retinopathy	-0.0437	0.0329	G
rs16957304	Waist circumference	-0.0590	0.0110	G	Background diabetic retinopathy	0.0991	0.0844	G
rs17451107	Waist circumference	-0.0260	0.0036	C	Background diabetic retinopathy	-0.0050	0.0353	C
rs1776897	Waist circumference	-0.0610	0.0067	T	Background diabetic retinopathy	0.1090	0.0736	T

rs1812175	Waist circumference	0.0330	0.0045	G	Background diabetic retinopathy	-0.0663	0.0440	G
rs2047937	Waist circumference	-0.0190	0.0034	T	Background diabetic retinopathy	-0.0176	0.0330	T
rs2052670	Waist circumference	0.0200	0.0035	G	Background diabetic retinopathy	-0.0370	0.0339	G
rs2071449	Waist circumference	0.0320	0.0036	A	Background diabetic retinopathy	0.0475	0.0331	A
rs2124969	Waist circumference	0.0200	0.0034	C	Background diabetic retinopathy	0.0062	0.0330	C
rs2160077	Waist circumference	-0.0180	0.0033	A	Background diabetic retinopathy	-0.0193	0.0334	A
rs2179129	Waist circumference	-0.0190	0.0034	G	Background diabetic retinopathy	-0.0284	0.0340	G
rs2214442	Waist circumference	0.0260	0.0045	G	Background diabetic retinopathy	-0.0587	0.0333	G
rs2274432	Waist circumference	0.0250	0.0036	A	Background diabetic retinopathy	0.0469	0.0333	A
rs2638953	Waist circumference	0.0240	0.0036	C	Background diabetic retinopathy	0.0085	0.0356	C
rs272869	Waist circumference	0.0210	0.0034	G	Background diabetic retinopathy	-0.0301	0.0328	G
rs2745353	Waist circumference	0.0290	0.0033	T	Background diabetic retinopathy	-0.0565	0.0327	T
rs3760318	Waist circumference	-0.0210	0.0035	A	Background diabetic retinopathy	0.0179	0.0339	A
rs3786897	Waist circumference	0.0200	0.0035	G	Background diabetic retinopathy	-0.0229	0.0334	G
rs3791679	Waist circumference	-0.0350	0.0039	G	Background diabetic retinopathy	0.0435	0.0392	G
rs395962	Waist circumference	-0.0290	0.0036	G	Background diabetic retinopathy	-0.0396	0.0353	G
rs4239436	Waist circumference	0.0400	0.0041	G	Background diabetic retinopathy	-0.0432	0.0392	G
rs4246302	Waist circumference	0.0220	0.0037	G	Background diabetic retinopathy	-0.0255	0.0339	G
rs4542783	Waist circumference	-0.0230	0.0040	C	Background diabetic retinopathy	-0.0469	0.0332	C
rs4567683	Waist circumference	-0.0220	0.0038	G	Background diabetic retinopathy	0.0062	0.0403	G
rs459193	Waist circumference	-0.0250	0.0038	G	Background diabetic retinopathy	0.0431	0.0353	G
rs473902	Waist circumference	-0.0490	0.0071	G	Background diabetic retinopathy	0.0470	0.0542	G
rs4886782	Waist circumference	-0.0240	0.0036	A	Background diabetic retinopathy	0.0336	0.0342	A
rs606452	Waist circumference	-0.0280	0.0048	C	Background diabetic retinopathy	0.0508	0.0427	C
rs6556301	Waist circumference	0.0280	0.0039	T	Background diabetic retinopathy	-0.0074	0.0341	T
rs6751657	Waist circumference	0.0180	0.0033	C	Background diabetic retinopathy	-0.0117	0.0330	C
rs6905288	Waist circumference	0.0290	0.0038	A	Background diabetic retinopathy	0.0069	0.0332	A
rs710841	Waist circumference	0.0290	0.0038	T	Background diabetic retinopathy	0.0022	0.0385	T
rs7162542	Waist circumference	0.0380	0.0034	G	Background diabetic retinopathy	-0.0159	0.0330	G
rs7430034	Waist circumference	0.0210	0.0035	T	Background diabetic retinopathy	0.0061	0.0365	T
rs757608	Waist circumference	-0.0270	0.0036	G	Background diabetic retinopathy	-0.0986	0.0366	G

rs7684221	Waist circumference	-0.0260	0.0047	A	Background diabetic retinopathy	0.0248	0.0564	A
rs7801581	Waist circumference	0.0270	0.0042	T	Background diabetic retinopathy	-0.0126	0.0382	T
rs780159	Waist circumference	0.0210	0.0035	G	Background diabetic retinopathy	-0.0180	0.0332	G
rs7970350	Waist circumference	-0.0190	0.0034	T	Background diabetic retinopathy	0.0054	0.0329	T
rs798489	Waist circumference	-0.0250	0.0037	T	Background diabetic retinopathy	0.0577	0.0345	T
rs806794	Waist circumference	-0.0300	0.0037	G	Background diabetic retinopathy	-0.0338	0.0339	G
rs822531	Waist circumference	0.0240	0.0044	T	Background diabetic retinopathy	-0.0381	0.0439	T
rs849140	Waist circumference	-0.0290	0.0034	C	Background diabetic retinopathy	0.0109	0.0336	C
rs9389986	Waist circumference	-0.0240	0.0037	A	Background diabetic retinopathy	0.0062	0.0373	A
rs9435732	Waist circumference	-0.0310	0.0038	T	Background diabetic retinopathy	-0.0229	0.0352	T
rs979012	Waist circumference	-0.0330	0.0036	C	Background diabetic retinopathy	-0.0449	0.0358	C
rs984222	Waist circumference	0.0360	0.0035	G	Background diabetic retinopathy	-0.0567	0.0365	G
rs9864077	Waist circumference	-0.0220	0.0037	C	Background diabetic retinopathy	0.0142	0.0358	C
rs991967	Waist circumference	0.0260	0.0037	C	Background diabetic retinopathy	-0.0176	0.0349	C
rs9977276	Waist circumference	0.0220	0.0040	G	Background diabetic retinopathy	-0.0288	0.0400	G
rs10041657	Waist circumference	0.0250	0.0040	A	Diabetic retinopathy	0.0132	0.0166	A
rs10748826	Waist circumference	-0.0230	0.0037	C	Diabetic retinopathy	-0.0103	0.0132	C
rs11144688	Waist circumference	-0.0340	0.0060	A	Diabetic retinopathy	-0.0217	0.0188	A
rs11205277	Waist circumference	0.0270	0.0036	G	Diabetic retinopathy	0.0027	0.0140	G
rs12207675	Waist circumference	0.0310	0.0052	C	Diabetic retinopathy	-0.0311	0.0192	C
rs12317176	Waist circumference	-0.0200	0.0035	C	Diabetic retinopathy	-0.0214	0.0143	C
rs12330322	Waist circumference	-0.0220	0.0040	T	Diabetic retinopathy	0.0186	0.0166	T
rs12493901	Waist circumference	-0.0210	0.0034	A	Diabetic retinopathy	0.0032	0.0132	A
rs12608504	Waist circumference	-0.0200	0.0036	G	Diabetic retinopathy	0.0225	0.0139	G
rs12656497	Waist circumference	-0.0220	0.0034	C	Diabetic retinopathy	0.0125	0.0133	C
rs12679556	Waist circumference	0.0260	0.0039	G	Diabetic retinopathy	0.0190	0.0154	G
rs12700664	Waist circumference	-0.0190	0.0034	T	Diabetic retinopathy	-0.0023	0.0131	T
rs12991495	Waist circumference	-0.0280	0.0037	C	Diabetic retinopathy	-0.0150	0.0148	C
rs13083798	Waist circumference	-0.0200	0.0034	G	Diabetic retinopathy	-0.0290	0.0131	G
rs13210323	Waist circumference	-0.0220	0.0038	C	Diabetic retinopathy	0.0096	0.0164	C
rs1344674	Waist circumference	0.0240	0.0033	G	Diabetic retinopathy	-0.0207	0.0132	G

rs16957304	Waist circumference	-0.0590	0.0110	G	Diabetic retinopathy	0.0266	0.0337	G
rs17451107	Waist circumference	-0.0260	0.0036	C	Diabetic retinopathy	0.0129	0.0142	C
rs1776897	Waist circumference	-0.0610	0.0067	T	Diabetic retinopathy	0.0166	0.0295	T
rs1812175	Waist circumference	0.0330	0.0045	G	Diabetic retinopathy	-0.0015	0.0177	G
rs2047937	Waist circumference	-0.0190	0.0034	T	Diabetic retinopathy	0.0027	0.0132	T
rs2052670	Waist circumference	0.0200	0.0035	G	Diabetic retinopathy	-0.0068	0.0136	G
rs2071449	Waist circumference	0.0320	0.0036	A	Diabetic retinopathy	0.0047	0.0133	A
rs2124969	Waist circumference	0.0200	0.0034	C	Diabetic retinopathy	0.0090	0.0132	C
rs2160077	Waist circumference	-0.0180	0.0033	A	Diabetic retinopathy	0.0182	0.0134	A
rs2179129	Waist circumference	-0.0190	0.0034	G	Diabetic retinopathy	-0.0026	0.0136	G
rs2214442	Waist circumference	0.0260	0.0045	G	Diabetic retinopathy	-0.0046	0.0133	G
rs2274432	Waist circumference	0.0250	0.0036	A	Diabetic retinopathy	-0.0041	0.0134	A
rs2638953	Waist circumference	0.0240	0.0036	C	Diabetic retinopathy	-0.0396	0.0143	C
rs272869	Waist circumference	0.0210	0.0034	G	Diabetic retinopathy	-0.0146	0.0132	G
rs2745353	Waist circumference	0.0290	0.0033	T	Diabetic retinopathy	-0.0170	0.0132	T
rs3760318	Waist circumference	-0.0210	0.0035	A	Diabetic retinopathy	-0.0045	0.0136	A
rs3786897	Waist circumference	0.0200	0.0035	G	Diabetic retinopathy	-0.0129	0.0134	G
rs3791679	Waist circumference	-0.0350	0.0039	G	Diabetic retinopathy	0.0128	0.0157	G
rs395962	Waist circumference	-0.0290	0.0036	G	Diabetic retinopathy	-0.0099	0.0142	G
rs4239436	Waist circumference	0.0400	0.0041	G	Diabetic retinopathy	-0.0094	0.0157	G
rs4246302	Waist circumference	0.0220	0.0037	G	Diabetic retinopathy	-0.0128	0.0136	G
rs4542783	Waist circumference	-0.0230	0.0040	C	Diabetic retinopathy	-0.0033	0.0132	C
rs4567683	Waist circumference	-0.0220	0.0038	G	Diabetic retinopathy	-0.0001	0.0161	G
rs459193	Waist circumference	-0.0250	0.0038	G	Diabetic retinopathy	0.0415	0.0141	G
rs473902	Waist circumference	-0.0490	0.0071	G	Diabetic retinopathy	0.0093	0.0221	G
rs4886782	Waist circumference	-0.0240	0.0036	A	Diabetic retinopathy	0.0131	0.0137	A
rs606452	Waist circumference	-0.0280	0.0048	C	Diabetic retinopathy	-0.0074	0.0171	C
rs6556301	Waist circumference	0.0280	0.0039	T	Diabetic retinopathy	0.0020	0.0137	T
rs6751657	Waist circumference	0.0180	0.0033	C	Diabetic retinopathy	0.0072	0.0133	C
rs6905288	Waist circumference	0.0290	0.0038	A	Diabetic retinopathy	0.0132	0.0133	A
rs710841	Waist circumference	0.0290	0.0038	T	Diabetic retinopathy	0.0042	0.0154	T

rs7162542	Waist circumference	0.0380	0.0034	G	Diabetic retinopathy	0.0061	0.0132	G
rs7430034	Waist circumference	0.0210	0.0035	T	Diabetic retinopathy	0.0199	0.0146	T
rs757608	Waist circumference	-0.0270	0.0036	G	Diabetic retinopathy	0.0009	0.0147	G
rs7684221	Waist circumference	-0.0260	0.0047	A	Diabetic retinopathy	0.0432	0.0225	A
rs7801581	Waist circumference	0.0270	0.0042	T	Diabetic retinopathy	-0.0085	0.0153	T
rs780159	Waist circumference	0.0210	0.0035	G	Diabetic retinopathy	0.0115	0.0133	G
rs7970350	Waist circumference	-0.0190	0.0034	T	Diabetic retinopathy	0.0157	0.0132	T
rs798489	Waist circumference	-0.0250	0.0037	T	Diabetic retinopathy	0.0159	0.0138	T
rs806794	Waist circumference	-0.0300	0.0037	G	Diabetic retinopathy	-0.0040	0.0135	G
rs822531	Waist circumference	0.0240	0.0044	T	Diabetic retinopathy	-0.0122	0.0176	T
rs849140	Waist circumference	-0.0290	0.0034	C	Diabetic retinopathy	0.0044	0.0135	C
rs9389986	Waist circumference	-0.0240	0.0037	A	Diabetic retinopathy	-0.0148	0.0150	A
rs9435732	Waist circumference	-0.0310	0.0038	T	Diabetic retinopathy	-0.0056	0.0141	T
rs979012	Waist circumference	-0.0330	0.0036	C	Diabetic retinopathy	-0.0238	0.0144	C
rs984222	Waist circumference	0.0360	0.0035	G	Diabetic retinopathy	-0.0066	0.0146	G
rs9864077	Waist circumference	-0.0220	0.0037	C	Diabetic retinopathy	0.0069	0.0144	C
rs991967	Waist circumference	0.0260	0.0037	C	Diabetic retinopathy	0.0118	0.0140	C
rs9977276	Waist circumference	0.0220	0.0040	G	Diabetic retinopathy	0.0125	0.0161	G
rs10041657	Waist circumference	0.0250	0.0040	A	Proliferative diabetic retinopathy	0.0228	0.0207	A
rs10748826	Waist circumference	-0.0230	0.0037	C	Proliferative diabetic retinopathy	-0.0068	0.0165	C
rs11144688	Waist circumference	-0.0340	0.0060	A	Proliferative diabetic retinopathy	-0.0069	0.0234	A
rs11205277	Waist circumference	0.0270	0.0036	G	Proliferative diabetic retinopathy	0.0018	0.0175	G
rs12207675	Waist circumference	0.0310	0.0052	C	Proliferative diabetic retinopathy	-0.0409	0.0240	C
rs12317176	Waist circumference	-0.0200	0.0035	C	Proliferative diabetic retinopathy	-0.0069	0.0179	C
rs12330322	Waist circumference	-0.0220	0.0040	T	Proliferative diabetic retinopathy	-0.0084	0.0207	T
rs12493901	Waist circumference	-0.0210	0.0034	A	Proliferative diabetic retinopathy	-0.0022	0.0164	A
rs12608504	Waist circumference	-0.0200	0.0036	G	Proliferative diabetic retinopathy	0.0100	0.0174	G
rs12656497	Waist circumference	-0.0220	0.0034	C	Proliferative diabetic retinopathy	-0.0020	0.0166	C
rs12679556	Waist circumference	0.0260	0.0039	G	Proliferative diabetic retinopathy	0.0195	0.0192	G
rs12700664	Waist circumference	-0.0190	0.0034	T	Proliferative diabetic retinopathy	-0.0115	0.0164	T
rs12991495	Waist circumference	-0.0280	0.0037	C	Proliferative diabetic retinopathy	0.0028	0.0185	C

rs13083798	Waist circumference	-0.0200	0.0034	G	Proliferative diabetic retinopathy	-0.0255	0.0164	G
rs13210323	Waist circumference	-0.0220	0.0038	C	Proliferative diabetic retinopathy	0.0264	0.0204	C
rs1344674	Waist circumference	0.0240	0.0033	G	Proliferative diabetic retinopathy	-0.0292	0.0165	G
rs16957304	Waist circumference	-0.0590	0.0110	G	Proliferative diabetic retinopathy	0.0783	0.0422	G
rs17451107	Waist circumference	-0.0260	0.0036	C	Proliferative diabetic retinopathy	0.0198	0.0177	C
rs1776897	Waist circumference	-0.0610	0.0067	T	Proliferative diabetic retinopathy	0.0325	0.0367	T
rs1812175	Waist circumference	0.0330	0.0045	G	Proliferative diabetic retinopathy	-0.0044	0.0221	G
rs2047937	Waist circumference	-0.0190	0.0034	T	Proliferative diabetic retinopathy	-0.0038	0.0165	T
rs2052670	Waist circumference	0.0200	0.0035	G	Proliferative diabetic retinopathy	0.0015	0.0169	G
rs2071449	Waist circumference	0.0320	0.0036	A	Proliferative diabetic retinopathy	0.0135	0.0166	A
rs2124969	Waist circumference	0.0200	0.0034	C	Proliferative diabetic retinopathy	0.0067	0.0165	C
rs2160077	Waist circumference	-0.0180	0.0033	A	Proliferative diabetic retinopathy	0.0207	0.0167	A
rs2179129	Waist circumference	-0.0190	0.0034	G	Proliferative diabetic retinopathy	-0.0010	0.0170	G
rs2214442	Waist circumference	0.0260	0.0045	G	Proliferative diabetic retinopathy	-0.0076	0.0167	G
rs2274432	Waist circumference	0.0250	0.0036	A	Proliferative diabetic retinopathy	-0.0002	0.0167	A
rs2638953	Waist circumference	0.0240	0.0036	C	Proliferative diabetic retinopathy	-0.0265	0.0178	C
rs272869	Waist circumference	0.0210	0.0034	G	Proliferative diabetic retinopathy	0.0013	0.0164	G
rs2745353	Waist circumference	0.0290	0.0033	T	Proliferative diabetic retinopathy	-0.0414	0.0164	T
rs3760318	Waist circumference	-0.0210	0.0035	A	Proliferative diabetic retinopathy	-0.0086	0.0170	A
rs3786897	Waist circumference	0.0200	0.0035	G	Proliferative diabetic retinopathy	-0.0089	0.0167	G
rs3791679	Waist circumference	-0.0350	0.0039	G	Proliferative diabetic retinopathy	0.0218	0.0196	G
rs395962	Waist circumference	-0.0290	0.0036	G	Proliferative diabetic retinopathy	-0.0334	0.0177	G
rs4239436	Waist circumference	0.0400	0.0041	G	Proliferative diabetic retinopathy	-0.0152	0.0196	G
rs4246302	Waist circumference	0.0220	0.0037	G	Proliferative diabetic retinopathy	-0.0166	0.0169	G
rs4542783	Waist circumference	-0.0230	0.0040	C	Proliferative diabetic retinopathy	-0.0076	0.0166	C
rs4567683	Waist circumference	-0.0220	0.0038	G	Proliferative diabetic retinopathy	-0.0015	0.0201	G
rs459193	Waist circumference	-0.0250	0.0038	G	Proliferative diabetic retinopathy	0.0369	0.0176	G
rs473902	Waist circumference	-0.0490	0.0071	G	Proliferative diabetic retinopathy	0.0022	0.0273	G
rs4886782	Waist circumference	-0.0240	0.0036	A	Proliferative diabetic retinopathy	0.0081	0.0171	A
rs606452	Waist circumference	-0.0280	0.0048	C	Proliferative diabetic retinopathy	-0.0027	0.0214	C
rs6556301	Waist circumference	0.0280	0.0039	T	Proliferative diabetic retinopathy	0.0035	0.0170	T

rs6751657	Waist circumference	0.0180	0.0033	C	Proliferative diabetic retinopathy	0.0043	0.0165	C
rs6905288	Waist circumference	0.0290	0.0038	A	Proliferative diabetic retinopathy	0.0118	0.0166	A
rs710841	Waist circumference	0.0290	0.0038	T	Proliferative diabetic retinopathy	0.0138	0.0192	T
rs7162542	Waist circumference	0.0380	0.0034	G	Proliferative diabetic retinopathy	0.0133	0.0165	G
rs7430034	Waist circumference	0.0210	0.0035	T	Proliferative diabetic retinopathy	-0.0143	0.0182	T
rs757608	Waist circumference	-0.0270	0.0036	G	Proliferative diabetic retinopathy	-0.0120	0.0183	G
rs7684221	Waist circumference	-0.0260	0.0047	A	Proliferative diabetic retinopathy	0.0362	0.0281	A
rs7801581	Waist circumference	0.0270	0.0042	T	Proliferative diabetic retinopathy	-0.0230	0.0191	T
rs780159	Waist circumference	0.0210	0.0035	G	Proliferative diabetic retinopathy	0.0395	0.0166	G
rs7970350	Waist circumference	-0.0190	0.0034	T	Proliferative diabetic retinopathy	0.0113	0.0165	T
rs798489	Waist circumference	-0.0250	0.0037	T	Proliferative diabetic retinopathy	-0.0077	0.0173	T
rs806794	Waist circumference	-0.0300	0.0037	G	Proliferative diabetic retinopathy	-0.0223	0.0169	G
rs822531	Waist circumference	0.0240	0.0044	T	Proliferative diabetic retinopathy	-0.0151	0.0219	T
rs849140	Waist circumference	-0.0290	0.0034	C	Proliferative diabetic retinopathy	0.0178	0.0168	C
rs9389986	Waist circumference	-0.0240	0.0037	A	Proliferative diabetic retinopathy	-0.0088	0.0187	A
rs9435732	Waist circumference	-0.0310	0.0038	T	Proliferative diabetic retinopathy	0.0082	0.0176	T
rs979012	Waist circumference	-0.0330	0.0036	C	Proliferative diabetic retinopathy	-0.0413	0.0179	C
rs984222	Waist circumference	0.0360	0.0035	G	Proliferative diabetic retinopathy	-0.0451	0.0183	G
rs9864077	Waist circumference	-0.0220	0.0037	C	Proliferative diabetic retinopathy	-0.0152	0.0179	C
rs991967	Waist circumference	0.0260	0.0037	C	Proliferative diabetic retinopathy	0.0110	0.0175	C
rs9977276	Waist circumference	0.0220	0.0040	G	Proliferative diabetic retinopathy	0.0020	0.0200	G
rs10245353	Waist-to-hip ratio	0.0350	0.0043	A	Background diabetic retinopathy	-0.0246	0.0372	A
rs10804591	Waist-to-hip ratio	0.0240	0.0042	A	Background diabetic retinopathy	0.0190	0.0411	A
rs10842707	Waist-to-hip ratio	0.0320	0.0040	T	Background diabetic retinopathy	0.0169	0.0370	T
rs10991437	Waist-to-hip ratio	0.0310	0.0054	A	Background diabetic retinopathy	-0.0169	0.0605	A
rs11231693	Waist-to-hip ratio	0.0410	0.0075	A	Background diabetic retinopathy	0.0511	0.0625	A
rs1128249	Waist-to-hip ratio	-0.0280	0.0035	T	Background diabetic retinopathy	-0.0131	0.0343	T
rs12143789	Waist-to-hip ratio	0.0240	0.0042	C	Background diabetic retinopathy	0.0008	0.0369	C
rs12608504	Waist-to-hip ratio	-0.0220	0.0036	G	Background diabetic retinopathy	0.0440	0.0347	G
rs12679556	Waist-to-hip ratio	0.0270	0.0040	G	Background diabetic retinopathy	0.0201	0.0383	G
rs1294410	Waist-to-hip ratio	0.0310	0.0035	C	Background diabetic retinopathy	-0.0156	0.0342	C

rs1385167	Waist-to-hip ratio	0.0290	0.0049	G	Background diabetic retinopathy	-0.0309	0.0411	G
rs1440372	Waist-to-hip ratio	0.0240	0.0038	C	Background diabetic retinopathy	0.0564	0.0377	C
rs1569135	Waist-to-hip ratio	-0.0210	0.0034	G	Background diabetic retinopathy	-0.0213	0.0332	G
rs17451107	Waist-to-hip ratio	-0.0260	0.0036	C	Background diabetic retinopathy	-0.0050	0.0353	C
rs17819328	Waist-to-hip ratio	0.0210	0.0035	G	Background diabetic retinopathy	0.0588	0.0330	G
rs1936805	Waist-to-hip ratio	0.0420	0.0034	T	Background diabetic retinopathy	-0.0565	0.0327	T
rs2071449	Waist-to-hip ratio	0.0280	0.0036	A	Background diabetic retinopathy	0.0475	0.0331	A
rs2276824	Waist-to-hip ratio	-0.0240	0.0036	G	Background diabetic retinopathy	-0.0809	0.0331	G
rs2294239	Waist-to-hip ratio	-0.0250	0.0035	G	Background diabetic retinopathy	-0.0275	0.0336	G
rs2645294	Waist-to-hip ratio	0.0310	0.0035	T	Background diabetic retinopathy	-0.0585	0.0352	T
rs2820443	Waist-to-hip ratio	-0.0350	0.0037	C	Background diabetic retinopathy	-0.0076	0.0359	C
rs303084	Waist-to-hip ratio	0.0230	0.0042	A	Background diabetic retinopathy	-0.0086	0.0452	A
rs4081724	Waist-to-hip ratio	-0.0350	0.0051	A	Background diabetic retinopathy	0.0721	0.0632	A
rs459193	Waist-to-hip ratio	-0.0260	0.0038	G	Background diabetic retinopathy	0.0431	0.0353	G
rs4646404	Waist-to-hip ratio	-0.0270	0.0039	A	Background diabetic retinopathy	0.0119	0.0350	A
rs4765219	Waist-to-hip ratio	-0.0280	0.0036	A	Background diabetic retinopathy	-0.0156	0.0357	A
rs6090583	Waist-to-hip ratio	-0.0220	0.0034	G	Background diabetic retinopathy	0.0582	0.0330	G
rs6772129	Waist-to-hip ratio	-0.0350	0.0037	G	Background diabetic retinopathy	0.0156	0.0358	G
rs714515	Waist-to-hip ratio	-0.0270	0.0034	A	Background diabetic retinopathy	0.0128	0.0330	A
rs7705502	Waist-to-hip ratio	0.0270	0.0036	A	Background diabetic retinopathy	-0.0456	0.0332	A
rs7759742	Waist-to-hip ratio	0.0230	0.0034	A	Background diabetic retinopathy	0.5116	0.0392	A
rs8030605	Waist-to-hip ratio	0.0300	0.0053	A	Background diabetic retinopathy	0.0066	0.0593	A
rs8042543	Waist-to-hip ratio	-0.0260	0.0043	T	Background diabetic retinopathy	-0.0605	0.0376	T
rs878639	Waist-to-hip ratio	-0.0210	0.0035	G	Background diabetic retinopathy	0.0167	0.0329	G
rs905938	Waist-to-hip ratio	-0.0250	0.0040	C	Background diabetic retinopathy	-0.0110	0.0388	C
rs979012	Waist-to-hip ratio	-0.0270	0.0036	C	Background diabetic retinopathy	-0.0449	0.0358	C
rs998584	Waist-to-hip ratio	0.0430	0.0038	A	Background diabetic retinopathy	0.0226	0.0331	A
rs9991328	Waist-to-hip ratio	0.0180	0.0034	T	Background diabetic retinopathy	0.0013	0.0329	T
rs10245353	Waist-to-hip ratio	0.0350	0.0043	A	Diabetic retinopathy	0.0002	0.0150	A
rs10804591	Waist-to-hip ratio	0.0240	0.0042	A	Diabetic retinopathy	0.0061	0.0165	A
rs10842707	Waist-to-hip ratio	0.0320	0.0040	T	Diabetic retinopathy	0.0301	0.0148	T

rs10991437	Waist-to-hip ratio	0.0310	0.0054	A	Diabetic retinopathy	0.0303	0.0243	A
rs11231693	Waist-to-hip ratio	0.0410	0.0075	A	Diabetic retinopathy	0.0184	0.0251	A
rs1128249	Waist-to-hip ratio	-0.0280	0.0035	T	Diabetic retinopathy	-0.0194	0.0138	T
rs12143789	Waist-to-hip ratio	0.0240	0.0042	C	Diabetic retinopathy	-0.0007	0.0148	C
rs12608504	Waist-to-hip ratio	-0.0220	0.0036	G	Diabetic retinopathy	0.0225	0.0139	G
rs12679556	Waist-to-hip ratio	0.0270	0.0040	G	Diabetic retinopathy	0.0190	0.0154	G
rs1294410	Waist-to-hip ratio	0.0310	0.0035	C	Diabetic retinopathy	-0.0018	0.0137	C
rs1385167	Waist-to-hip ratio	0.0290	0.0049	G	Diabetic retinopathy	0.0161	0.0164	G
rs1440372	Waist-to-hip ratio	0.0240	0.0038	C	Diabetic retinopathy	-0.0155	0.0151	C
rs1569135	Waist-to-hip ratio	-0.0210	0.0034	G	Diabetic retinopathy	-0.0049	0.0133	G
rs17451107	Waist-to-hip ratio	-0.0260	0.0036	C	Diabetic retinopathy	0.0129	0.0142	C
rs17819328	Waist-to-hip ratio	0.0210	0.0035	G	Diabetic retinopathy	0.0085	0.0132	G
rs1936805	Waist-to-hip ratio	0.0420	0.0034	T	Diabetic retinopathy	-0.0171	0.0132	T
rs2071449	Waist-to-hip ratio	0.0280	0.0036	A	Diabetic retinopathy	0.0047	0.0133	A
rs2276824	Waist-to-hip ratio	-0.0240	0.0036	G	Diabetic retinopathy	-0.0338	0.0133	G
rs2294239	Waist-to-hip ratio	-0.0250	0.0035	G	Diabetic retinopathy	0.0003	0.0135	G
rs2645294	Waist-to-hip ratio	0.0310	0.0035	T	Diabetic retinopathy	-0.0047	0.0141	T
rs2820443	Waist-to-hip ratio	-0.0350	0.0037	C	Diabetic retinopathy	-0.0344	0.0144	C
rs303084	Waist-to-hip ratio	0.0230	0.0042	A	Diabetic retinopathy	0.0151	0.0181	A
rs4081724	Waist-to-hip ratio	-0.0350	0.0051	A	Diabetic retinopathy	0.0269	0.0256	A
rs459193	Waist-to-hip ratio	-0.0260	0.0038	G	Diabetic retinopathy	0.0415	0.0141	G
rs4646404	Waist-to-hip ratio	-0.0270	0.0039	A	Diabetic retinopathy	-0.0072	0.0140	A
rs4765219	Waist-to-hip ratio	-0.0280	0.0036	A	Diabetic retinopathy	-0.0205	0.0143	A
rs6090583	Waist-to-hip ratio	-0.0220	0.0034	G	Diabetic retinopathy	-0.0114	0.0132	G
rs6772129	Waist-to-hip ratio	-0.0350	0.0037	G	Diabetic retinopathy	0.0068	0.0144	G
rs714515	Waist-to-hip ratio	-0.0270	0.0034	A	Diabetic retinopathy	0.0013	0.0132	A
rs7705502	Waist-to-hip ratio	0.0270	0.0036	A	Diabetic retinopathy	0.0038	0.0134	A
rs7759742	Waist-to-hip ratio	0.0230	0.0034	A	Diabetic retinopathy	0.2034	0.0157	A
rs8030605	Waist-to-hip ratio	0.0300	0.0053	A	Diabetic retinopathy	0.0050	0.0237	A
rs8042543	Waist-to-hip ratio	-0.0260	0.0043	T	Diabetic retinopathy	-0.0203	0.0151	T
rs878639	Waist-to-hip ratio	-0.0210	0.0035	G	Diabetic retinopathy	0.0208	0.0132	G

rs905938	Waist-to-hip ratio	-0.0250	0.0040	C	Diabetic retinopathy	0.0155	0.0156	C
rs979012	Waist-to-hip ratio	-0.0270	0.0036	C	Diabetic retinopathy	-0.0238	0.0144	C
rs998584	Waist-to-hip ratio	0.0430	0.0038	A	Diabetic retinopathy	0.0099	0.0133	A
rs9991328	Waist-to-hip ratio	0.0180	0.0034	T	Diabetic retinopathy	0.0391	0.0132	T
rs10245353	Waist-to-hip ratio	0.0350	0.0043	A	Proliferative diabetic retinopathy	0.0000	0.0186	A
rs10804591	Waist-to-hip ratio	0.0240	0.0042	A	Proliferative diabetic retinopathy	0.0063	0.0206	A
rs10842707	Waist-to-hip ratio	0.0320	0.0040	T	Proliferative diabetic retinopathy	0.0337	0.0185	T
rs10991437	Waist-to-hip ratio	0.0310	0.0054	A	Proliferative diabetic retinopathy	0.0357	0.0303	A
rs11231693	Waist-to-hip ratio	0.0410	0.0075	A	Proliferative diabetic retinopathy	-0.0005	0.0313	A
rs1128249	Waist-to-hip ratio	-0.0280	0.0035	T	Proliferative diabetic retinopathy	-0.0219	0.0172	T
rs12143789	Waist-to-hip ratio	0.0240	0.0042	C	Proliferative diabetic retinopathy	-0.0105	0.0184	C
rs12608504	Waist-to-hip ratio	-0.0220	0.0036	G	Proliferative diabetic retinopathy	0.0100	0.0174	G
rs12679556	Waist-to-hip ratio	0.0270	0.0040	G	Proliferative diabetic retinopathy	0.0195	0.0192	G
rs1294410	Waist-to-hip ratio	0.0310	0.0035	C	Proliferative diabetic retinopathy	0.0118	0.0171	C
rs1385167	Waist-to-hip ratio	0.0290	0.0049	G	Proliferative diabetic retinopathy	0.0109	0.0205	G
rs1440372	Waist-to-hip ratio	0.0240	0.0038	C	Proliferative diabetic retinopathy	-0.0194	0.0189	C
rs1569135	Waist-to-hip ratio	-0.0210	0.0034	G	Proliferative diabetic retinopathy	-0.0210	0.0166	G
rs17451107	Waist-to-hip ratio	-0.0260	0.0036	C	Proliferative diabetic retinopathy	0.0198	0.0177	C
rs17819328	Waist-to-hip ratio	0.0210	0.0035	G	Proliferative diabetic retinopathy	0.0340	0.0165	G
rs1936805	Waist-to-hip ratio	0.0420	0.0034	T	Proliferative diabetic retinopathy	-0.0417	0.0164	T
rs2071449	Waist-to-hip ratio	0.0280	0.0036	A	Proliferative diabetic retinopathy	0.0135	0.0166	A
rs2276824	Waist-to-hip ratio	-0.0240	0.0036	G	Proliferative diabetic retinopathy	-0.0288	0.0165	G
rs2294239	Waist-to-hip ratio	-0.0250	0.0035	G	Proliferative diabetic retinopathy	0.0038	0.0168	G
rs2645294	Waist-to-hip ratio	0.0310	0.0035	T	Proliferative diabetic retinopathy	-0.0348	0.0176	T
rs2820443	Waist-to-hip ratio	-0.0350	0.0037	C	Proliferative diabetic retinopathy	-0.0274	0.0180	C
rs303084	Waist-to-hip ratio	0.0230	0.0042	A	Proliferative diabetic retinopathy	0.0369	0.0227	A
rs4081724	Waist-to-hip ratio	-0.0350	0.0051	A	Proliferative diabetic retinopathy	0.0411	0.0318	A
rs459193	Waist-to-hip ratio	-0.0260	0.0038	G	Proliferative diabetic retinopathy	0.0369	0.0176	G
rs4646404	Waist-to-hip ratio	-0.0270	0.0039	A	Proliferative diabetic retinopathy	-0.0244	0.0175	A
rs4765219	Waist-to-hip ratio	-0.0280	0.0036	A	Proliferative diabetic retinopathy	-0.0080	0.0178	A
rs6090583	Waist-to-hip ratio	-0.0220	0.0034	G	Proliferative diabetic retinopathy	-0.0218	0.0165	G

rs6772129	Waist-to-hip ratio	-0.0350	0.0037	G	Proliferative diabetic retinopathy	-0.0150	0.0179	G
rs714515	Waist-to-hip ratio	-0.0270	0.0034	A	Proliferative diabetic retinopathy	0.0125	0.0165	A
rs7705502	Waist-to-hip ratio	0.0270	0.0036	A	Proliferative diabetic retinopathy	-0.0167	0.0166	A
rs7759742	Waist-to-hip ratio	0.0230	0.0034	A	Proliferative diabetic retinopathy	0.2809	0.0195	A
rs8030605	Waist-to-hip ratio	0.0300	0.0053	A	Proliferative diabetic retinopathy	0.0077	0.0297	A
rs8042543	Waist-to-hip ratio	-0.0260	0.0043	T	Proliferative diabetic retinopathy	-0.0425	0.0189	T
rs878639	Waist-to-hip ratio	-0.0210	0.0035	G	Proliferative diabetic retinopathy	0.0110	0.0165	G
rs905938	Waist-to-hip ratio	-0.0250	0.0040	C	Proliferative diabetic retinopathy	0.0018	0.0194	C
rs979012	Waist-to-hip ratio	-0.0270	0.0036	C	Proliferative diabetic retinopathy	-0.0413	0.0179	C
rs998584	Waist-to-hip ratio	0.0430	0.0038	A	Proliferative diabetic retinopathy	0.0027	0.0165	A
rs9991328	Waist-to-hip ratio	0.0180	0.0034	T	Proliferative diabetic retinopathy	0.0429	0.0164	T

Table S2. Mendelian randomization (MR) estimates of the associations between unhealthy lifestyles and risk of background diabetic retinopathy (BDR)

Exposure	IVW method (fixed-effect)		MR-Egger		Weighted median method	
	OR (95% CI)	*P*-value	OR (95% CI)	*P*-value	OR (95% CI)	*P*-value
**Smoking**						
Age Of Smoking Initiation	0.54(0.13,2.16)	0.381	0.08(0.00,46.94)	0.401	0.87(0.12,6.01)	0.885
Cigarettes per day	1.12(0.88,1.43)	0.351	1.15(0.75,1.76)	0.536	1.18(0.85,1.64)	0.322
Ever smoked	1.20(0.38,3.75)	0.756	0.44(0.00,178.54)	0.792	0.55(0.10,2.86)	0.474
**Alcohol intake**						
Alcohol intake frequency	0.88(0.63,1.23)	0.458	0.25(0.08,0.78)	0.019*	0.75(0.44,1.28)	0.296
Alcoholic drinks per week	1.58(0.71,3.51)	0.266	8.41(1.06,66.89)	0.053	4.10(1.17,14.39)	0.028*
**Obesity**						
Body mass index	1.59(1.29,1.97)	<0.001*	2.10 (1.06,4.16)	0.035*	1.93(1.36,2.72)	<0.001*
Waist circumference	0.80(0.57,1.14)	0.220	0.32(0.06,1.56)	0.163	0.74(0.44,1.24)	0.258
Hip circumference	0.72(0.55,0.95)	0.022*	0.68(0.24,1.91)	0.467	0.62(0.41,0.94)	0.024*
Waist-to-hip ratio	0.94(0.61,1.43)	0.767	0.39(0.05,2.94)	0.369	1.09(0.59,2.05)	0.777

IVW = inverse-variance weighted; OR = odds ratio; CI = confidence interval.

Table S3. MR estimates of the associations between unhealthy lifestyles and risk of proliferative diabetic retinopathy (PDR)

Exposure	IVW method		MR-Egger		Weighted median method	
	**OR (95% CI)**	***P*-value**	**OR (95% CI)**	***P*-value**	**OR (95% CI)**	***P*-value**
**Smoking**						
Age Of Smoking Initiation	1.12(0.56,2.25)	0.755	0.44(0.05,4.13)	0.502	1.31(0.52,3.30)	0.563
Cigarettes per day	1.18(1.04,1.33)	0.009*	1.20(0.94,1.52)	0.157	1.18(0.99,1.39)	0.057
Ever smoked	0.68(0.38,1.20)	0.182	0.15(0.01,3.36)	0.235	0.35(0.15,0.84)	0.019*
**Alcohol intake**						
Alcohol intake frequency	1.00(0.85,1.18)	0.975	0.65(0.34,1.24)	0.199	0.85(0.65,1.12)	0.242
Alcoholic drinks per week	0.94(0.63,1.41)	0.774	0.91(0.35,2.36)	0.854	0.82(0.45,1.47)	0.503
**Obesity**						
Body mass index	1.43(1.29,1.59)	<0.001*	1.62(1.14,2.30)	0.008*	1.61(1.35,1.92)	<0.001*
Waist circumference	0.95(0.79,1.13)	0.529	0.63(0.29,1.37)	0.248	1.07(0.82,1.39)	0.616
Hip circumference	0.85(0.74,0.98)	0.026*	0.93(0.55,1.56)	0.783	0.92(0.75,0.92)	0.419
Waist-to-hip ratio	1.17(0.95,1.45)	0.140	0.35(0.10,1.55)	0.117	1.24(0.88,1.74)	0.223

IVW = inverse-variance weighted; OR = odds ratio; CI = confidence interval.

Table S4. Sensitivity test of MR analyze of the associations between unhealthy lifestyles and risk of BDR

Exposure	MR-Egger regression analysis		Cochran's Q test		IVW (Multiplicative random effects)	
	Intercept	*P-value*	*Q statistic*	*P-value*	**OR (95% CI)**	*P-value*
**Smoke**						
Age Of Smoking Initiation	0.044	0.496	8.00	0.156	0.54(0.10,2.92)	0.471
Cigarettes per day	-0.002	0.906	13.25	0.866	1.12(0.93,1.36)	0.240
Ever smoked	0.007	0.741	89.73	0.118	1.20(0.35,4.14)	0.775
**Alcohol intake**						
Alcohol intake frequency	0.032	0.024*	112.98	<0.044*	0.88(0.60,1.29)	0.520
Alcoholic drinks per week	-0.032	0.088	40.11	0.127	1.58(0.62,4.04)	0.343
**Obesity**						
Body mass index	-0.006	0.392	343.21	0.009*	1.59(1.26,2.01)	<0.001*
Waist circumference	0.025	0.245	65.24	0.332	0.80(0.56,1.16)	0.237
Hip circumference	0.002	0.908	56.93	0.870	0.72(0.56,0.93)	0.011*
Waist-to-hip ratio	0.025	0.391	31.39	0.596	0.94(0.63,1.41)	0.757

IVW = inverse-variance weighted; MR = Mendelian randomization.

Table S5. Sensitivity test of MR analyze of the associations between unhealthy lifestyles and risk of PDR

**Exposure**	**MR-Egger regression analysis**		**Cochran's Q test**		**IVW (Multiplicative random effects)**	
	Intercept	***P-value***	***Q statistic***	***P-value***	**OR (95% CI)**	***P-value***
**Smoke**						
Age Of Smoking Initiation	0.021	0.427	5.30	0.381	1.12(0.55,2.26)	0.757
Cigarettes per day	-0.001	0.884	24.71	0.213	1.18(1.03,1.34)	0.015*
Ever smoked	0.011	0.333	96.58	0.047*	0.68(0.35,1.30)	0.239
**Alcohol intake**						
Alcohol intake frequency	0.011	0.172	142.28	<0.001*	1.00(0.81,1.24)	0.980
Alcoholic drinks per week	0.001	0.944	33.62	0.342	0.94(0.63,1.42)	0.779
**Obesity**						
Body mass index	-0.003	0.464	364.51	0.001*	1.43(1.27,1.61)	<0.001*
Waist circumference	0.011	0.296	62.65	0.417	0.95(0.79,1.13)	0.535
Hip circumference	-0.003	0.733	52.55	0.941	0.85(0.76,0.96)	0.010*
Waist-to-hip ratio	0.035	0.067	55.10	0.012*	1.17(0.89,1.55)	0.264

IVW = inverse-variance weighted; MR = Mendelian randomization.

Table S6. The result of MR-PRESSO

Exposure	Outcome	beta	sd	OR(95%CI)	P-value	Corrected P-value
Age Of Smoking Initiation	Background diabetic retinopathy	NA	NA	NA	0.498	NA
Alcohol intake frequency	Background diabetic retinopathy	-0.050	0.179	0.95(0.67,1.35)	0.616	0.781
Alcoholic drinks per week	Background diabetic retinopathy	NA	NA	NA	0.350	NA
Body mass index	Background diabetic retinopathy	0.505	0.113	1.66(1.33,2.07)	0.000	0.000
Cigarettes per Day	Background diabetic retinopathy	NA	NA	NA	0.284	NA
Ever smoked	Background diabetic retinopathy	NA	NA	NA	0.731	NA
Hip circumference	Background diabetic retinopathy	NA	NA	NA	0.016	NA
Waist circumference	Background diabetic retinopathy	NA	NA	NA	0.141	NA
Waist-to-hip ratio	Background diabetic retinopathy	0.018	0.219	1.02(0.66,1.56)	0.475	0.935
Age Of Smoking Initiation	Diabetic retinopathy	NA	NA	NA	0.674	NA
Alcohol intake frequency	Diabetic retinopathy	0.122	0.075	1.13(0.97,1.31)	0.218	0.108
Alcoholic drinks per week	Diabetic retinopathy	NA	NA	NA	0.815	NA
Body mass index	Diabetic retinopathy	0.350	0.044	1.42(1.3,1.55)	0.000	0.000
Cigarettes per Day	Diabetic retinopathy	NA	NA	NA	0.020	NA

Ever smoked	Diabetic retinopathy	-0.271	0.250	0.76(0.47,1.25)	0.510	0.283
Hip circumference	Diabetic retinopathy	NA	NA	NA	0.009	NA
Waist circumference	Diabetic retinopathy	NA	NA	NA	0.268	NA
Waist-to-hip ratio	Diabetic retinopathy	0.212	0.100	1.24(1.02,1.5)	0.151	0.041
Age Of Smoking Initiation	Proliferative diabetic retinopathy	NA	NA	NA	0.767	NA
Alcohol intake frequency	Proliferative diabetic retinopathy	0.059	0.099	1.06(0.87,1.29)	0.773	0.549
Alcoholic drinks per week	Proliferative diabetic retinopathy	NA	NA	NA	0.781	NA
Body mass index	Proliferative diabetic retinopathy	0.367	0.055	1.44(1.29,1.61)	0.000	0.000
Cigarettes per Day	Proliferative diabetic retinopathy	NA	NA	NA	0.023	NA
Ever smoked	Proliferative diabetic retinopathy	-0.408	0.310	0.66(0.36,1.22)	0.394	0.191
Hip circumference	Proliferative diabetic retinopathy	NA	NA	NA	0.034	NA
Waist circumference	Proliferative diabetic retinopathy	NA	NA	NA	0.412	NA
Waist-to-hip ratio	Proliferative diabetic retinopathy	0.279	0.137	1.32(1.01,1.73)	0.195	0.049

Table S7. The result of leave-one-out analysis in each single-nucleotide polymorphism

Exposure	Outcome	id of exposure	id of outcome	SNP	b	se	p
Age Of Smoking Initiation	Background diabetic retinopathy	ieu-b-24	finn-b-DM_BCKGRND_RETINA	rs10200107	-0.755	1.031	0.464
Age Of Smoking Initiation	Background diabetic retinopathy	ieu-b-24	finn-b-DM_BCKGRND_RETINA	rs11780471	-0.062	0.831	0.941
Age Of Smoking Initiation	Background diabetic retinopathy	ieu-b-24	finn-b-DM_BCKGRND_RETINA	rs11915747	-0.890	1.024	0.385
Age Of Smoking Initiation	Background diabetic retinopathy	ieu-b-24	finn-b-DM_BCKGRND_RETINA	rs140485736	-0.680	1.007	0.499
Age Of Smoking Initiation	Background diabetic retinopathy	ieu-b-24	finn-b-DM_BCKGRND_RETINA	rs319748	-1.123	0.754	0.137
Age Of Smoking Initiation	Background diabetic retinopathy	ieu-b-24	finn-b-DM_BCKGRND_RETINA	rs3768886	-0.237	0.875	0.786
Age Of Smoking Initiation	Background diabetic retinopathy	ieu-b-24	finn-b-DM_BCKGRND_RETINA	rs624833	-0.622	1.016	0.540

Age Of Smoking Initiation	Background diabetic retinopathy	ieu-b-24	finn-b-DM_BCKGRND_RETINA	All	-0.625	0.866	0.471
Age Of Smoking Initiation	Diabetic retinopathy	ieu-b-24	finn-b-DM_RETINOPATHY_EXMORE	rs10200107	-0.204	0.313	0.514
Age Of Smoking Initiation	Diabetic retinopathy	ieu-b-24	finn-b-DM_RETINOPATHY_EXMORE	rs11780471	0.373	0.414	0.367
Age Of Smoking Initiation	Diabetic retinopathy	ieu-b-24	finn-b-DM_RETINOPATHY_EXMORE	rs11915747	0.135	0.476	0.776
Age Of Smoking Initiation	Diabetic retinopathy	ieu-b-24	finn-b-DM_RETINOPATHY_EXMORE	rs140485736	0.236	0.447	0.598
Age Of Smoking Initiation	Diabetic retinopathy	ieu-b-24	finn-b-DM_RETINOPATHY_EXMORE	rs319748	0.180	0.453	0.690
Age Of Smoking Initiation	Diabetic retinopathy	ieu-b-24	finn-b-DM_RETINOPATHY_EXMORE	rs3768886	0.313	0.416	0.453
Age Of Smoking Initiation	Diabetic retinopathy	ieu-b-24	finn-b-DM_RETINOPATHY_EXMORE	rs624833	0.159	0.458	0.728

Age Of Smoking Initiation	Diabetic retinopathy	ieu-b-24	finn-b-DM_RETINOPATHY_EXMORE	All	0.172	0.390	0.659
Age Of Smoking Initiation	Proliferative diabetic retinopathy	ieu-b-24	finn-b-DM_RETINA_PROLIF	rs10200107	-0.101	0.391	0.796
Age Of Smoking Initiation	Proliferative diabetic retinopathy	ieu-b-24	finn-b-DM_RETINA_PROLIF	rs11780471	0.250	0.402	0.534
Age Of Smoking Initiation	Proliferative diabetic retinopathy	ieu-b-24	finn-b-DM_RETINA_PROLIF	rs11915747	-0.071	0.399	0.859
Age Of Smoking Initiation	Proliferative diabetic retinopathy	ieu-b-24	finn-b-DM_RETINA_PROLIF	rs140485736	0.213	0.396	0.590
Age Of Smoking Initiation	Proliferative diabetic retinopathy	ieu-b-24	finn-b-DM_RETINA_PROLIF	rs319748	0.028	0.400	0.945
Age Of Smoking Initiation	Proliferative diabetic retinopathy	ieu-b-24	finn-b-DM_RETINA_PROLIF	rs3768886	0.315	0.379	0.406
Age Of Smoking Initiation	Proliferative diabetic retinopathy	ieu-b-24	finn-b-DM_RETINA_PROLIF	rs624833	0.123	0.421	0.770

Age Of Smoking Initiation	Proliferative diabetic retinopathy	ieu-b-24	finn-b-DM_RETINA_PROLIF	All	0.111	0.359	0.757
Alcohol intake frequency	Background diabetic retinopathy	ukb-b-5779	finn-b-DM_BCKGRND_RETINA	rs10188314	-0.136	0.197	0.489
Alcohol intake frequency	Background diabetic retinopathy	ukb-b-5779	finn-b-DM_BCKGRND_RETINA	rs10792669	-0.156	0.193	0.419
Alcohol intake frequency	Background diabetic retinopathy	ukb-b-5779	finn-b-DM_BCKGRND_RETINA	rs11039429	-0.147	0.196	0.455
Alcohol intake frequency	Background diabetic retinopathy	ukb-b-5779	finn-b-DM_BCKGRND_RETINA	rs11223617	-0.137	0.197	0.486
Alcohol intake frequency	Background diabetic retinopathy	ukb-b-5779	finn-b-DM_BCKGRND_RETINA	rs11700855	-0.121	0.197	0.539
Alcohol intake frequency	Background diabetic retinopathy	ukb-b-5779	finn-b-DM_BCKGRND_RETINA	rs11750777	-0.123	0.197	0.532
Alcohol intake frequency	Background diabetic retinopathy	ukb-b-5779	finn-b-DM_BCKGRND_RETINA	rs11787216	-0.133	0.197	0.500

Alcohol intake frequency	Background diabetic retinopathy	ukb-b-5779	finn-b-DM_BCKGRND_RETINA	rs11940694	-0.085	0.200	0.671
Alcohol intake frequency	Background diabetic retinopathy	ukb-b-5779	finn-b-DM_BCKGRND_RETINA	rs12153855	-0.078	0.183	0.668
Alcohol intake frequency	Background diabetic retinopathy	ukb-b-5779	finn-b-DM_BCKGRND_RETINA	rs1228589	-0.126	0.197	0.523
Alcohol intake frequency	Background diabetic retinopathy	ukb-b-5779	finn-b-DM_BCKGRND_RETINA	rs1229984	-0.089	0.199	0.654
Alcohol intake frequency	Background diabetic retinopathy	ukb-b-5779	finn-b-DM_BCKGRND_RETINA	rs12312693	-0.119	0.197	0.546
Alcohol intake frequency	Background diabetic retinopathy	ukb-b-5779	finn-b-DM_BCKGRND_RETINA	rs13102973	-0.126	0.197	0.522
Alcohol intake frequency	Background diabetic retinopathy	ukb-b-5779	finn-b-DM_BCKGRND_RETINA	rs13135092	-0.115	0.195	0.556
Alcohol intake frequency	Background diabetic retinopathy	ukb-b-5779	finn-b-DM_BCKGRND_RETINA	rs13178443	-0.153	0.194	0.430

Alcohol intake frequency	Background diabetic retinopathy	ukb-b-5779	finn-b-DM_BCKGRND_RETINA	rs13390019	-0.125	0.197	0.524
Alcohol intake frequency	Background diabetic retinopathy	ukb-b-5779	finn-b-DM_BCKGRND_RETINA	rs1421085	-0.166	0.192	0.387
Alcohol intake frequency	Background diabetic retinopathy	ukb-b-5779	finn-b-DM_BCKGRND_RETINA	rs1515591	-0.133	0.197	0.500
Alcohol intake frequency	Background diabetic retinopathy	ukb-b-5779	finn-b-DM_BCKGRND_RETINA	rs1666658	-0.131	0.197	0.504
Alcohol intake frequency	Background diabetic retinopathy	ukb-b-5779	finn-b-DM_BCKGRND_RETINA	rs17662759	-0.138	0.196	0.483
Alcohol intake frequency	Background diabetic retinopathy	ukb-b-5779	finn-b-DM_BCKGRND_RETINA	rs17690703	-0.137	0.196	0.485
Alcohol intake frequency	Background diabetic retinopathy	ukb-b-5779	finn-b-DM_BCKGRND_RETINA	rs186347	-0.113	0.196	0.566
Alcohol intake frequency	Background diabetic retinopathy	ukb-b-5779	finn-b-DM_BCKGRND_RETINA	rs1937522	-0.121	0.197	0.537

Alcohol intake frequency	Background diabetic retinopathy	ukb-b-5779	finn-b-DM_BCKGRND_RETINA	rs1991083	-0.130	0.197	0.508
Alcohol intake frequency	Background diabetic retinopathy	ukb-b-5779	finn-b-DM_BCKGRND_RETINA	rs2043677	-0.111	0.196	0.572
Alcohol intake frequency	Background diabetic retinopathy	ukb-b-5779	finn-b-DM_BCKGRND_RETINA	rs2159935	-0.107	0.196	0.585
Alcohol intake frequency	Background diabetic retinopathy	ukb-b-5779	finn-b-DM_BCKGRND_RETINA	rs2160935	-0.151	0.195	0.437
Alcohol intake frequency	Background diabetic retinopathy	ukb-b-5779	finn-b-DM_BCKGRND_RETINA	rs2244598	-0.144	0.196	0.462
Alcohol intake frequency	Background diabetic retinopathy	ukb-b-5779	finn-b-DM_BCKGRND_RETINA	rs2411453	-0.100	0.199	0.616
Alcohol intake frequency	Background diabetic retinopathy	ukb-b-5779	finn-b-DM_BCKGRND_RETINA	rs2535911	-0.112	0.196	0.570
Alcohol intake frequency	Background diabetic retinopathy	ukb-b-5779	finn-b-DM_BCKGRND_RETINA	rs2622167	-0.132	0.197	0.502

Alcohol intake frequency	Background diabetic retinopathy	ukb-b-5779	finn-b-DM_BCKGRND_RETINA	rs262240	-0.104	0.195	0.592
Alcohol intake frequency	Background diabetic retinopathy	ukb-b-5779	finn-b-DM_BCKGRND_RETINA	rs2717063	-0.147	0.196	0.452
Alcohol intake frequency	Background diabetic retinopathy	ukb-b-5779	finn-b-DM_BCKGRND_RETINA	rs28768122	-0.132	0.197	0.501
Alcohol intake frequency	Background diabetic retinopathy	ukb-b-5779	finn-b-DM_BCKGRND_RETINA	rs28787109	-0.138	0.196	0.480
Alcohol intake frequency	Background diabetic retinopathy	ukb-b-5779	finn-b-DM_BCKGRND_RETINA	rs2924321	-0.126	0.197	0.521
Alcohol intake frequency	Background diabetic retinopathy	ukb-b-5779	finn-b-DM_BCKGRND_RETINA	rs2977454	-0.101	0.195	0.605
Alcohol intake frequency	Background diabetic retinopathy	ukb-b-5779	finn-b-DM_BCKGRND_RETINA	rs34440851	-0.135	0.197	0.493
Alcohol intake frequency	Background diabetic retinopathy	ukb-b-5779	finn-b-DM_BCKGRND_RETINA	rs34473884	-0.142	0.196	0.468

Alcohol intake frequency	Background diabetic retinopathy	ukb-b-5779	finn-b-DM_BCKGRND_RETINA	rs34631026	-0.146	0.195	0.453
Alcohol intake frequency	Background diabetic retinopathy	ukb-b-5779	finn-b-DM_BCKGRND_RETINA	rs34811474	-0.120	0.197	0.542
Alcohol intake frequency	Background diabetic retinopathy	ukb-b-5779	finn-b-DM_BCKGRND_RETINA	rs35105141	-0.138	0.197	0.485
Alcohol intake frequency	Background diabetic retinopathy	ukb-b-5779	finn-b-DM_BCKGRND_RETINA	rs362307	-0.123	0.197	0.534
Alcohol intake frequency	Background diabetic retinopathy	ukb-b-5779	finn-b-DM_BCKGRND_RETINA	rs4241258	-0.125	0.197	0.523
Alcohol intake frequency	Background diabetic retinopathy	ukb-b-5779	finn-b-DM_BCKGRND_RETINA	rs4242715	-0.137	0.196	0.487
Alcohol intake frequency	Background diabetic retinopathy	ukb-b-5779	finn-b-DM_BCKGRND_RETINA	rs4417025	-0.141	0.196	0.470
Alcohol intake frequency	Background diabetic retinopathy	ukb-b-5779	finn-b-DM_BCKGRND_RETINA	rs4503294	-0.112	0.196	0.569

Alcohol intake frequency	Background diabetic retinopathy	ukb-b-5779	finn-b-DM_BCKGRND_RETINA	rs461599	-0.147	0.196	0.451
Alcohol intake frequency	Background diabetic retinopathy	ukb-b-5779	finn-b-DM_BCKGRND_RETINA	rs4726481	-0.085	0.193	0.659
Alcohol intake frequency	Background diabetic retinopathy	ukb-b-5779	finn-b-DM_BCKGRND_RETINA	rs473098	-0.151	0.195	0.439
Alcohol intake frequency	Background diabetic retinopathy	ukb-b-5779	finn-b-DM_BCKGRND_RETINA	rs489062	-0.113	0.196	0.565
Alcohol intake frequency	Background diabetic retinopathy	ukb-b-5779	finn-b-DM_BCKGRND_RETINA	rs4916723	-0.102	0.196	0.605
Alcohol intake frequency	Background diabetic retinopathy	ukb-b-5779	finn-b-DM_BCKGRND_RETINA	rs4940926	-0.150	0.194	0.439
Alcohol intake frequency	Background diabetic retinopathy	ukb-b-5779	finn-b-DM_BCKGRND_RETINA	rs4968391	-0.108	0.196	0.582
Alcohol intake frequency	Background diabetic retinopathy	ukb-b-5779	finn-b-DM_BCKGRND_RETINA	rs5022348	-0.139	0.197	0.480

Alcohol intake frequency	Background diabetic retinopathy	ukb-b-5779	finn-b-DM_BCKGRND_RETINA	rs550942	-0.112	0.195	0.566
Alcohol intake frequency	Background diabetic retinopathy	ukb-b-5779	finn-b-DM_BCKGRND_RETINA	rs56194430	-0.131	0.196	0.505
Alcohol intake frequency	Background diabetic retinopathy	ukb-b-5779	finn-b-DM_BCKGRND_RETINA	rs58905411	-0.132	0.198	0.505
Alcohol intake frequency	Background diabetic retinopathy	ukb-b-5779	finn-b-DM_BCKGRND_RETINA	rs6030200	-0.124	0.197	0.530
Alcohol intake frequency	Background diabetic retinopathy	ukb-b-5779	finn-b-DM_BCKGRND_RETINA	rs61873510	-0.126	0.197	0.522
Alcohol intake frequency	Background diabetic retinopathy	ukb-b-5779	finn-b-DM_BCKGRND_RETINA	rs62305780	-0.121	0.199	0.545
Alcohol intake frequency	Background diabetic retinopathy	ukb-b-5779	finn-b-DM_BCKGRND_RETINA	rs62339673	-0.159	0.193	0.410
Alcohol intake frequency	Background diabetic retinopathy	ukb-b-5779	finn-b-DM_BCKGRND_RETINA	rs62466318	-0.107	0.196	0.586

Alcohol intake frequency	Background diabetic retinopathy	ukb-b-5779	finn-b-DM_BCKGRND_RETINA	rs650558	-0.118	0.197	0.549
Alcohol intake frequency	Background diabetic retinopathy	ukb-b-5779	finn-b-DM_BCKGRND_RETINA	rs6727281	-0.141	0.197	0.472
Alcohol intake frequency	Background diabetic retinopathy	ukb-b-5779	finn-b-DM_BCKGRND_RETINA	rs6943160	-0.120	0.197	0.541
Alcohol intake frequency	Background diabetic retinopathy	ukb-b-5779	finn-b-DM_BCKGRND_RETINA	rs71651683	-0.135	0.196	0.491
Alcohol intake frequency	Background diabetic retinopathy	ukb-b-5779	finn-b-DM_BCKGRND_RETINA	rs72769229	-0.118	0.196	0.549
Alcohol intake frequency	Background diabetic retinopathy	ukb-b-5779	finn-b-DM_BCKGRND_RETINA	rs72787062	-0.102	0.196	0.603
Alcohol intake frequency	Background diabetic retinopathy	ukb-b-5779	finn-b-DM_BCKGRND_RETINA	rs728538	-0.151	0.195	0.438
Alcohol intake frequency	Background diabetic retinopathy	ukb-b-5779	finn-b-DM_BCKGRND_RETINA	rs7298932	-0.141	0.195	0.470

Alcohol intake frequency	Background diabetic retinopathy	ukb-b-5779	finn-b-DM_BCKGRND_RETINA	rs7302200	-0.089	0.192	0.641
Alcohol intake frequency	Background diabetic retinopathy	ukb-b-5779	finn-b-DM_BCKGRND_RETINA	rs73050128	-0.137	0.197	0.487
Alcohol intake frequency	Background diabetic retinopathy	ukb-b-5779	finn-b-DM_BCKGRND_RETINA	rs7330939	-0.124	0.197	0.529
Alcohol intake frequency	Background diabetic retinopathy	ukb-b-5779	finn-b-DM_BCKGRND_RETINA	rs74679146	-0.129	0.197	0.512
Alcohol intake frequency	Background diabetic retinopathy	ukb-b-5779	finn-b-DM_BCKGRND_RETINA	rs7514579	-0.122	0.197	0.535
Alcohol intake frequency	Background diabetic retinopathy	ukb-b-5779	finn-b-DM_BCKGRND_RETINA	rs76082653	-0.125	0.197	0.523
Alcohol intake frequency	Background diabetic retinopathy	ukb-b-5779	finn-b-DM_BCKGRND_RETINA	rs7610856	-0.164	0.194	0.398
Alcohol intake frequency	Background diabetic retinopathy	ukb-b-5779	finn-b-DM_BCKGRND_RETINA	rs780094	-0.085	0.202	0.675

Alcohol intake frequency	Background diabetic retinopathy	ukb-b-5779	finn-b-DM_BCKGRND_RETINA	rs780569	-0.112	0.196	0.569
Alcohol intake frequency	Background diabetic retinopathy	ukb-b-5779	finn-b-DM_BCKGRND_RETINA	rs80292319	-0.111	0.197	0.574
Alcohol intake frequency	Background diabetic retinopathy	ukb-b-5779	finn-b-DM_BCKGRND_RETINA	rs8043563	-0.106	0.196	0.590
Alcohol intake frequency	Background diabetic retinopathy	ukb-b-5779	finn-b-DM_BCKGRND_RETINA	rs838145	-0.083	0.193	0.667
Alcohol intake frequency	Background diabetic retinopathy	ukb-b-5779	finn-b-DM_BCKGRND_RETINA	rs8614	-0.097	0.194	0.617
Alcohol intake frequency	Background diabetic retinopathy	ukb-b-5779	finn-b-DM_BCKGRND_RETINA	rs9349379	-0.109	0.196	0.578
Alcohol intake frequency	Background diabetic retinopathy	ukb-b-5779	finn-b-DM_BCKGRND_RETINA	rs9372625	-0.151	0.196	0.441
Alcohol intake frequency	Background diabetic retinopathy	ukb-b-5779	finn-b-DM_BCKGRND_RETINA	rs9403297	-0.151	0.194	0.436

Alcohol intake frequency	Background diabetic retinopathy	ukb-b-5779	finn-b-DM_BCKGRND_RETINA	rs9648478	-0.139	0.196	0.479
Alcohol intake frequency	Background diabetic retinopathy	ukb-b-5779	finn-b-DM_BCKGRND_RETINA	rs9814516	-0.109	0.196	0.580
Alcohol intake frequency	Background diabetic retinopathy	ukb-b-5779	finn-b-DM_BCKGRND_RETINA	rs9829192	-0.125	0.197	0.524
Alcohol intake frequency	Background diabetic retinopathy	ukb-b-5779	finn-b-DM_BCKGRND_RETINA	rs9906502	-0.144	0.196	0.464
Alcohol intake frequency	Background diabetic retinopathy	ukb-b-5779	finn-b-DM_BCKGRND_RETINA	All	-0.125	0.195	0.520
Alcohol intake frequency	Diabetic retinopathy	ukb-b-5779	finn-b-DM_RETINOPATHY_EXMORE	rs10188314	0.084	0.089	0.345
Alcohol intake frequency	Diabetic retinopathy	ukb-b-5779	finn-b-DM_RETINOPATHY_EXMORE	rs10792669	0.085	0.089	0.340
Alcohol intake frequency	Diabetic retinopathy	ukb-b-5779	finn-b-DM_RETINOPATHY_EXMORE	rs11039429	0.093	0.089	0.299

Alcohol intake frequency	Diabetic retinopathy	ukb-b-5779	finn-b-DM_RETINOPATHY_EXMORE	rs11223617	0.088	0.089	0.324
Alcohol intake frequency	Diabetic retinopathy	ukb-b-5779	finn-b-DM_RETINOPATHY_EXMORE	rs11700855	0.090	0.089	0.312
Alcohol intake frequency	Diabetic retinopathy	ukb-b-5779	finn-b-DM_RETINOPATHY_EXMORE	rs11750777	0.082	0.088	0.351
Alcohol intake frequency	Diabetic retinopathy	ukb-b-5779	finn-b-DM_RETINOPATHY_EXMORE	rs11787216	0.086	0.089	0.332
Alcohol intake frequency	Diabetic retinopathy	ukb-b-5779	finn-b-DM_RETINOPATHY_EXMORE	rs11940694	0.106	0.090	0.241
Alcohol intake frequency	Diabetic retinopathy	ukb-b-5779	finn-b-DM_RETINOPATHY_EXMORE	rs12153855	0.109	0.084	0.198
Alcohol intake frequency	Diabetic retinopathy	ukb-b-5779	finn-b-DM_RETINOPATHY_EXMORE	rs1228589	0.085	0.089	0.339
Alcohol intake frequency	Diabetic retinopathy	ukb-b-5779	finn-b-DM_RETINOPATHY_EXMORE	rs1229984	0.074	0.090	0.408

Alcohol intake frequency	Diabetic retinopathy	ukb-b-5779	finn-b-DM_RETINOPATHY_EXMORE	rs12312693	0.096	0.089	0.281
Alcohol intake frequency	Diabetic retinopathy	ukb-b-5779	finn-b-DM_RETINOPATHY_EXMORE	rs13102973	0.094	0.089	0.292
Alcohol intake frequency	Diabetic retinopathy	ukb-b-5779	finn-b-DM_RETINOPATHY_EXMORE	rs13135092	0.087	0.088	0.325
Alcohol intake frequency	Diabetic retinopathy	ukb-b-5779	finn-b-DM_RETINOPATHY_EXMORE	rs13178443	0.082	0.088	0.354
Alcohol intake frequency	Diabetic retinopathy	ukb-b-5779	finn-b-DM_RETINOPATHY_EXMORE	rs13390019	0.087	0.089	0.324
Alcohol intake frequency	Diabetic retinopathy	ukb-b-5779	finn-b-DM_RETINOPATHY_EXMORE	rs1421085	0.063	0.084	0.454
Alcohol intake frequency	Diabetic retinopathy	ukb-b-5779	finn-b-DM_RETINOPATHY_EXMORE	rs1515591	0.092	0.089	0.303
Alcohol intake frequency	Diabetic retinopathy	ukb-b-5779	finn-b-DM_RETINOPATHY_EXMORE	rs1666658	0.103	0.088	0.239

Alcohol intake frequency	Diabetic retinopathy	ukb-b-5779	finn-b-DM_RETINOPATHY_EXMORE	rs17662759	0.085	0.089	0.336
Alcohol intake frequency	Diabetic retinopathy	ukb-b-5779	finn-b-DM_RETINOPATHY_EXMORE	rs17690703	0.088	0.089	0.320
Alcohol intake frequency	Diabetic retinopathy	ukb-b-5779	finn-b-DM_RETINOPATHY_EXMORE	rs186347	0.102	0.088	0.245
Alcohol intake frequency	Diabetic retinopathy	ukb-b-5779	finn-b-DM_RETINOPATHY_EXMORE	rs1937522	0.089	0.089	0.314
Alcohol intake frequency	Diabetic retinopathy	ukb-b-5779	finn-b-DM_RETINOPATHY_EXMORE	rs1991083	0.099	0.088	0.262
Alcohol intake frequency	Diabetic retinopathy	ukb-b-5779	finn-b-DM_RETINOPATHY_EXMORE	rs2043677	0.087	0.089	0.327
Alcohol intake frequency	Diabetic retinopathy	ukb-b-5779	finn-b-DM_RETINOPATHY_EXMORE	rs2159935	0.090	0.089	0.309
Alcohol intake frequency	Diabetic retinopathy	ukb-b-5779	finn-b-DM_RETINOPATHY_EXMORE	rs2160935	0.080	0.088	0.363

Alcohol intake frequency	Diabetic retinopathy	ukb-b-5779	finn-b-DM_RETINOPATHY_EXMORE	rs2244598	0.083	0.088	0.349
Alcohol intake frequency	Diabetic retinopathy	ukb-b-5779	finn-b-DM_RETINOPATHY_EXMORE	rs2411453	0.118	0.089	0.183
Alcohol intake frequency	Diabetic retinopathy	ukb-b-5779	finn-b-DM_RETINOPATHY_EXMORE	rs2535911	0.104	0.088	0.237
Alcohol intake frequency	Diabetic retinopathy	ukb-b-5779	finn-b-DM_RETINOPATHY_EXMORE	rs2622167	0.088	0.089	0.324
Alcohol intake frequency	Diabetic retinopathy	ukb-b-5779	finn-b-DM_RETINOPATHY_EXMORE	rs262240	0.090	0.089	0.313
Alcohol intake frequency	Diabetic retinopathy	ukb-b-5779	finn-b-DM_RETINOPATHY_EXMORE	rs2717063	0.087	0.089	0.326
Alcohol intake frequency	Diabetic retinopathy	ukb-b-5779	finn-b-DM_RETINOPATHY_EXMORE	rs28768122	0.085	0.089	0.340
Alcohol intake frequency	Diabetic retinopathy	ukb-b-5779	finn-b-DM_RETINOPATHY_EXMORE	rs28787109	0.091	0.089	0.307

Alcohol intake frequency	Diabetic retinopathy	ukb-b-5779	finn-b-DM_RETINOPATHY_EXMORE	rs2924321	0.095	0.089	0.285
Alcohol intake frequency	Diabetic retinopathy	ukb-b-5779	finn-b-DM_RETINOPATHY_EXMORE	rs2977454	0.083	0.089	0.347
Alcohol intake frequency	Diabetic retinopathy	ukb-b-5779	finn-b-DM_RETINOPATHY_EXMORE	rs34440851	0.095	0.089	0.284
Alcohol intake frequency	Diabetic retinopathy	ukb-b-5779	finn-b-DM_RETINOPATHY_EXMORE	rs34473884	0.084	0.089	0.342
Alcohol intake frequency	Diabetic retinopathy	ukb-b-5779	finn-b-DM_RETINOPATHY_EXMORE	rs34631026	0.077	0.087	0.375
Alcohol intake frequency	Diabetic retinopathy	ukb-b-5779	finn-b-DM_RETINOPATHY_EXMORE	rs34811474	0.082	0.088	0.355
Alcohol intake frequency	Diabetic retinopathy	ukb-b-5779	finn-b-DM_RETINOPATHY_EXMORE	rs35105141	0.097	0.089	0.277
Alcohol intake frequency	Diabetic retinopathy	ukb-b-5779	finn-b-DM_RETINOPATHY_EXMORE	rs362307	0.085	0.089	0.337

Alcohol intake frequency	Diabetic retinopathy	ukb-b-5779	finn-b-DM_RETINOPATHY_EXMORE	rs4241258	0.093	0.089	0.292
Alcohol intake frequency	Diabetic retinopathy	ukb-b-5779	finn-b-DM_RETINOPATHY_EXMORE	rs4242715	0.102	0.088	0.248
Alcohol intake frequency	Diabetic retinopathy	ukb-b-5779	finn-b-DM_RETINOPATHY_EXMORE	rs4417025	0.088	0.089	0.322
Alcohol intake frequency	Diabetic retinopathy	ukb-b-5779	finn-b-DM_RETINOPATHY_EXMORE	rs4503294	0.091	0.089	0.306
Alcohol intake frequency	Diabetic retinopathy	ukb-b-5779	finn-b-DM_RETINOPATHY_EXMORE	rs461599	0.092	0.089	0.299
Alcohol intake frequency	Diabetic retinopathy	ukb-b-5779	finn-b-DM_RETINOPATHY_EXMORE	rs4726481	0.093	0.089	0.297
Alcohol intake frequency	Diabetic retinopathy	ukb-b-5779	finn-b-DM_RETINOPATHY_EXMORE	rs473098	0.084	0.089	0.345
Alcohol intake frequency	Diabetic retinopathy	ukb-b-5779	finn-b-DM_RETINOPATHY_EXMORE	rs489062	0.084	0.088	0.343

Alcohol intake frequency	Diabetic retinopathy	ukb-b-5779	finn-b-DM_RETINOPATHY_EXMORE	rs4916723	0.098	0.089	0.273
Alcohol intake frequency	Diabetic retinopathy	ukb-b-5779	finn-b-DM_RETINOPATHY_EXMORE	rs4940926	0.080	0.088	0.362
Alcohol intake frequency	Diabetic retinopathy	ukb-b-5779	finn-b-DM_RETINOPATHY_EXMORE	rs4968391	0.100	0.088	0.259
Alcohol intake frequency	Diabetic retinopathy	ukb-b-5779	finn-b-DM_RETINOPATHY_EXMORE	rs5022348	0.079	0.088	0.373
Alcohol intake frequency	Diabetic retinopathy	ukb-b-5779	finn-b-DM_RETINOPATHY_EXMORE	rs550942	0.099	0.088	0.260
Alcohol intake frequency	Diabetic retinopathy	ukb-b-5779	finn-b-DM_RETINOPATHY_EXMORE	rs56194430	0.087	0.089	0.324
Alcohol intake frequency	Diabetic retinopathy	ukb-b-5779	finn-b-DM_RETINOPATHY_EXMORE	rs58905411	0.073	0.088	0.410
Alcohol intake frequency	Diabetic retinopathy	ukb-b-5779	finn-b-DM_RETINOPATHY_EXMORE	rs6030200	0.096	0.089	0.277

Alcohol intake frequency	Diabetic retinopathy	ukb-b-5779	finn-b-DM_RETINOPATHY_EXMORE	rs61873510	0.082	0.088	0.352
Alcohol intake frequency	Diabetic retinopathy	ukb-b-5779	finn-b-DM_RETINOPATHY_EXMORE	rs62305780	0.105	0.089	0.240
Alcohol intake frequency	Diabetic retinopathy	ukb-b-5779	finn-b-DM_RETINOPATHY_EXMORE	rs62339673	0.082	0.088	0.354
Alcohol intake frequency	Diabetic retinopathy	ukb-b-5779	finn-b-DM_RETINOPATHY_EXMORE	rs62466318	0.104	0.088	0.238
Alcohol intake frequency	Diabetic retinopathy	ukb-b-5779	finn-b-DM_RETINOPATHY_EXMORE	rs650558	0.087	0.089	0.328
Alcohol intake frequency	Diabetic retinopathy	ukb-b-5779	finn-b-DM_RETINOPATHY_EXMORE	rs6727281	0.088	0.089	0.324
Alcohol intake frequency	Diabetic retinopathy	ukb-b-5779	finn-b-DM_RETINOPATHY_EXMORE	rs6943160	0.079	0.088	0.367
Alcohol intake frequency	Diabetic retinopathy	ukb-b-5779	finn-b-DM_RETINOPATHY_EXMORE	rs71651683	0.092	0.089	0.300

Alcohol intake frequency	Diabetic retinopathy	ukb-b-5779	finn-b-DM_RETINOPATHY_EXMORE	rs72769229	0.093	0.089	0.296
Alcohol intake frequency	Diabetic retinopathy	ukb-b-5779	finn-b-DM_RETINOPATHY_EXMORE	rs72787062	0.105	0.088	0.232
Alcohol intake frequency	Diabetic retinopathy	ukb-b-5779	finn-b-DM_RETINOPATHY_EXMORE	rs728538	0.091	0.089	0.307
Alcohol intake frequency	Diabetic retinopathy	ukb-b-5779	finn-b-DM_RETINOPATHY_EXMORE	rs7298932	0.089	0.089	0.317
Alcohol intake frequency	Diabetic retinopathy	ukb-b-5779	finn-b-DM_RETINOPATHY_EXMORE	rs7302200	0.114	0.084	0.177
Alcohol intake frequency	Diabetic retinopathy	ukb-b-5779	finn-b-DM_RETINOPATHY_EXMORE	rs73050128	0.086	0.089	0.334
Alcohol intake frequency	Diabetic retinopathy	ukb-b-5779	finn-b-DM_RETINOPATHY_EXMORE	rs7330939	0.092	0.089	0.300
Alcohol intake frequency	Diabetic retinopathy	ukb-b-5779	finn-b-DM_RETINOPATHY_EXMORE	rs74679146	0.087	0.089	0.325

Alcohol intake frequency	Diabetic retinopathy	ukb-b-5779	finn-b-DM_RETINOPATHY_EXMORE	rs7514579	0.088	0.089	0.322
Alcohol intake frequency	Diabetic retinopathy	ukb-b-5779	finn-b-DM_RETINOPATHY_EXMORE	rs76082653	0.091	0.089	0.304
Alcohol intake frequency	Diabetic retinopathy	ukb-b-5779	finn-b-DM_RETINOPATHY_EXMORE	rs7610856	0.076	0.088	0.389
Alcohol intake frequency	Diabetic retinopathy	ukb-b-5779	finn-b-DM_RETINOPATHY_EXMORE	rs780094	0.129	0.090	0.154
Alcohol intake frequency	Diabetic retinopathy	ukb-b-5779	finn-b-DM_RETINOPATHY_EXMORE	rs780569	0.084	0.088	0.344
Alcohol intake frequency	Diabetic retinopathy	ukb-b-5779	finn-b-DM_RETINOPATHY_EXMORE	rs80292319	0.095	0.089	0.285
Alcohol intake frequency	Diabetic retinopathy	ukb-b-5779	finn-b-DM_RETINOPATHY_EXMORE	rs8043563	0.096	0.089	0.280
Alcohol intake frequency	Diabetic retinopathy	ukb-b-5779	finn-b-DM_RETINOPATHY_EXMORE	rs838145	0.102	0.088	0.246

Alcohol intake frequency	Diabetic retinopathy	ukb-b-5779	finn-b-DM_RETINOPATHY_EXMORE	rs8614	0.097	0.089	0.274
Alcohol intake frequency	Diabetic retinopathy	ukb-b-5779	finn-b-DM_RETINOPATHY_EXMORE	rs9349379	0.088	0.089	0.321
Alcohol intake frequency	Diabetic retinopathy	ukb-b-5779	finn-b-DM_RETINOPATHY_EXMORE	rs9372625	0.086	0.089	0.333
Alcohol intake frequency	Diabetic retinopathy	ukb-b-5779	finn-b-DM_RETINOPATHY_EXMORE	rs9403297	0.084	0.089	0.343
Alcohol intake frequency	Diabetic retinopathy	ukb-b-5779	finn-b-DM_RETINOPATHY_EXMORE	rs9648478	0.084	0.088	0.345
Alcohol intake frequency	Diabetic retinopathy	ukb-b-5779	finn-b-DM_RETINOPATHY_EXMORE	rs9814516	0.089	0.089	0.316
Alcohol intake frequency	Diabetic retinopathy	ukb-b-5779	finn-b-DM_RETINOPATHY_EXMORE	rs9829192	0.094	0.089	0.292
Alcohol intake frequency	Diabetic retinopathy	ukb-b-5779	finn-b-DM_RETINOPATHY_EXMORE	rs9906502	0.093	0.089	0.298

Alcohol intake frequency	Diabetic retinopathy	ukb-b-5779	finn-b-DM_RETINOPATHY_EXMORE	All	0.090	0.088	0.304
Alcohol intake frequency	Proliferative diabetic retinopathy	ukb-b-5779	finn-b-DM_RETINA_PROLIF	rs10188314	-0.004	0.108	0.970
Alcohol intake frequency	Proliferative diabetic retinopathy	ukb-b-5779	finn-b-DM_RETINA_PROLIF	rs10792669	-0.007	0.108	0.947
Alcohol intake frequency	Proliferative diabetic retinopathy	ukb-b-5779	finn-b-DM_RETINA_PROLIF	rs11039429	0.007	0.109	0.949
Alcohol intake frequency	Proliferative diabetic retinopathy	ukb-b-5779	finn-b-DM_RETINA_PROLIF	rs11223617	-0.013	0.108	0.903
Alcohol intake frequency	Proliferative diabetic retinopathy	ukb-b-5779	finn-b-DM_RETINA_PROLIF	rs11700855	0.003	0.109	0.975
Alcohol intake frequency	Proliferative diabetic retinopathy	ukb-b-5779	finn-b-DM_RETINA_PROLIF	rs11750777	0.000	0.108	1.000
Alcohol intake frequency	Proliferative diabetic retinopathy	ukb-b-5779	finn-b-DM_RETINA_PROLIF	rs11787216	-0.004	0.109	0.971

Alcohol intake frequency	Proliferative diabetic retinopathy	ukb-b-5779	finn-b-DM_RETINA_PROLIF	rs11940694	0.019	0.110	0.860
Alcohol intake frequency	Proliferative diabetic retinopathy	ukb-b-5779	finn-b-DM_RETINA_PROLIF	rs12153855	0.032	0.099	0.749
Alcohol intake frequency	Proliferative diabetic retinopathy	ukb-b-5779	finn-b-DM_RETINA_PROLIF	rs1228589	0.000	0.108	1.000
Alcohol intake frequency	Proliferative diabetic retinopathy	ukb-b-5779	finn-b-DM_RETINA_PROLIF	rs1229984	-0.023	0.109	0.832
Alcohol intake frequency	Proliferative diabetic retinopathy	ukb-b-5779	finn-b-DM_RETINA_PROLIF	rs12312693	0.004	0.108	0.970
Alcohol intake frequency	Proliferative diabetic retinopathy	ukb-b-5779	finn-b-DM_RETINA_PROLIF	rs13102973	0.012	0.108	0.914
Alcohol intake frequency	Proliferative diabetic retinopathy	ukb-b-5779	finn-b-DM_RETINA_PROLIF	rs13135092	0.002	0.108	0.988
Alcohol intake frequency	Proliferative diabetic retinopathy	ukb-b-5779	finn-b-DM_RETINA_PROLIF	rs13178443	-0.008	0.108	0.939

Alcohol intake frequency	Proliferative diabetic retinopathy	ukb-b-5779	finn-b-DM_RETINA_PROLIF	rs13390019	-0.002	0.108	0.985
Alcohol intake frequency	Proliferative diabetic retinopathy	ukb-b-5779	finn-b-DM_RETINA_PROLIF	rs1421085	-0.020	0.106	0.853
Alcohol intake frequency	Proliferative diabetic retinopathy	ukb-b-5779	finn-b-DM_RETINA_PROLIF	rs1515591	-0.003	0.108	0.974
Alcohol intake frequency	Proliferative diabetic retinopathy	ukb-b-5779	finn-b-DM_RETINA_PROLIF	rs1666658	0.001	0.108	0.989
Alcohol intake frequency	Proliferative diabetic retinopathy	ukb-b-5779	finn-b-DM_RETINA_PROLIF	rs17662759	-0.013	0.107	0.906
Alcohol intake frequency	Proliferative diabetic retinopathy	ukb-b-5779	finn-b-DM_RETINA_PROLIF	rs17690703	0.000	0.108	0.999
Alcohol intake frequency	Proliferative diabetic retinopathy	ukb-b-5779	finn-b-DM_RETINA_PROLIF	rs186347	0.017	0.107	0.871
Alcohol intake frequency	Proliferative diabetic retinopathy	ukb-b-5779	finn-b-DM_RETINA_PROLIF	rs1937522	0.005	0.108	0.960

Alcohol intake frequency	Proliferative diabetic retinopathy	ukb-b-5779	finn-b-DM_RETINA_PROLIF	rs1991083	0.013	0.108	0.902
Alcohol intake frequency	Proliferative diabetic retinopathy	ukb-b-5779	finn-b-DM_RETINA_PROLIF	rs2043677	0.002	0.109	0.983
Alcohol intake frequency	Proliferative diabetic retinopathy	ukb-b-5779	finn-b-DM_RETINA_PROLIF	rs2159935	-0.002	0.108	0.984
Alcohol intake frequency	Proliferative diabetic retinopathy	ukb-b-5779	finn-b-DM_RETINA_PROLIF	rs2160935	-0.009	0.108	0.930
Alcohol intake frequency	Proliferative diabetic retinopathy	ukb-b-5779	finn-b-DM_RETINA_PROLIF	rs2244598	-0.001	0.108	0.996
Alcohol intake frequency	Proliferative diabetic retinopathy	ukb-b-5779	finn-b-DM_RETINA_PROLIF	rs2411453	0.034	0.108	0.750
Alcohol intake frequency	Proliferative diabetic retinopathy	ukb-b-5779	finn-b-DM_RETINA_PROLIF	rs2535911	0.015	0.108	0.890
Alcohol intake frequency	Proliferative diabetic retinopathy	ukb-b-5779	finn-b-DM_RETINA_PROLIF	rs2622167	0.001	0.108	0.993

Alcohol intake frequency	Proliferative diabetic retinopathy	ukb-b-5779	finn-b-DM_RETINA_PROLIF	rs262240	0.007	0.108	0.945
Alcohol intake frequency	Proliferative diabetic retinopathy	ukb-b-5779	finn-b-DM_RETINA_PROLIF	rs2717063	-0.004	0.108	0.973
Alcohol intake frequency	Proliferative diabetic retinopathy	ukb-b-5779	finn-b-DM_RETINA_PROLIF	rs28768122	-0.005	0.108	0.960
Alcohol intake frequency	Proliferative diabetic retinopathy	ukb-b-5779	finn-b-DM_RETINA_PROLIF	rs28787109	-0.003	0.108	0.976
Alcohol intake frequency	Proliferative diabetic retinopathy	ukb-b-5779	finn-b-DM_RETINA_PROLIF	rs2924321	0.009	0.108	0.931
Alcohol intake frequency	Proliferative diabetic retinopathy	ukb-b-5779	finn-b-DM_RETINA_PROLIF	rs2977454	0.006	0.109	0.956
Alcohol intake frequency	Proliferative diabetic retinopathy	ukb-b-5779	finn-b-DM_RETINA_PROLIF	rs34440851	0.014	0.108	0.900
Alcohol intake frequency	Proliferative diabetic retinopathy	ukb-b-5779	finn-b-DM_RETINA_PROLIF	rs34473884	-0.004	0.108	0.969

Alcohol intake frequency	Proliferative diabetic retinopathy	ukb-b-5779	finn-b-DM_RETINA_PROLIF	rs34631026	-0.012	0.107	0.914
Alcohol intake frequency	Proliferative diabetic retinopathy	ukb-b-5779	finn-b-DM_RETINA_PROLIF	rs34811474	0.001	0.108	0.996
Alcohol intake frequency	Proliferative diabetic retinopathy	ukb-b-5779	finn-b-DM_RETINA_PROLIF	rs35105141	0.020	0.108	0.850
Alcohol intake frequency	Proliferative diabetic retinopathy	ukb-b-5779	finn-b-DM_RETINA_PROLIF	rs362307	-0.007	0.108	0.947
Alcohol intake frequency	Proliferative diabetic retinopathy	ukb-b-5779	finn-b-DM_RETINA_PROLIF	rs4241258	0.008	0.108	0.939
Alcohol intake frequency	Proliferative diabetic retinopathy	ukb-b-5779	finn-b-DM_RETINA_PROLIF	rs4242715	0.008	0.108	0.938
Alcohol intake frequency	Proliferative diabetic retinopathy	ukb-b-5779	finn-b-DM_RETINA_PROLIF	rs4417025	-0.004	0.108	0.972
Alcohol intake frequency	Proliferative diabetic retinopathy	ukb-b-5779	finn-b-DM_RETINA_PROLIF	rs4503294	-0.002	0.108	0.987

Alcohol intake frequency	Proliferative diabetic retinopathy	ukb-b-5779	finn-b-DM_RETINA_PROLIF	rs461599	0.006	0.108	0.953
Alcohol intake frequency	Proliferative diabetic retinopathy	ukb-b-5779	finn-b-DM_RETINA_PROLIF	rs4726481	0.005	0.109	0.964
Alcohol intake frequency	Proliferative diabetic retinopathy	ukb-b-5779	finn-b-DM_RETINA_PROLIF	rs473098	-0.003	0.109	0.974
Alcohol intake frequency	Proliferative diabetic retinopathy	ukb-b-5779	finn-b-DM_RETINA_PROLIF	rs489062	-0.004	0.108	0.972
Alcohol intake frequency	Proliferative diabetic retinopathy	ukb-b-5779	finn-b-DM_RETINA_PROLIF	rs4916723	0.016	0.108	0.885
Alcohol intake frequency	Proliferative diabetic retinopathy	ukb-b-5779	finn-b-DM_RETINA_PROLIF	rs4940926	-0.012	0.106	0.908
Alcohol intake frequency	Proliferative diabetic retinopathy	ukb-b-5779	finn-b-DM_RETINA_PROLIF	rs4968391	0.014	0.108	0.899
Alcohol intake frequency	Proliferative diabetic retinopathy	ukb-b-5779	finn-b-DM_RETINA_PROLIF	rs5022348	-0.014	0.107	0.898

Alcohol intake frequency	Proliferative diabetic retinopathy	ukb-b-5779	finn-b-DM_RETINA_PROLIF	rs550942	0.011	0.108	0.922
Alcohol intake frequency	Proliferative diabetic retinopathy	ukb-b-5779	finn-b-DM_RETINA_PROLIF	rs56194430	0.003	0.108	0.979
Alcohol intake frequency	Proliferative diabetic retinopathy	ukb-b-5779	finn-b-DM_RETINA_PROLIF	rs58905411	-0.016	0.108	0.885
Alcohol intake frequency	Proliferative diabetic retinopathy	ukb-b-5779	finn-b-DM_RETINA_PROLIF	rs6030200	0.007	0.108	0.950
Alcohol intake frequency	Proliferative diabetic retinopathy	ukb-b-5779	finn-b-DM_RETINA_PROLIF	rs61873510	-0.007	0.108	0.948
Alcohol intake frequency	Proliferative diabetic retinopathy	ukb-b-5779	finn-b-DM_RETINA_PROLIF	rs62305780	0.010	0.110	0.926
Alcohol intake frequency	Proliferative diabetic retinopathy	ukb-b-5779	finn-b-DM_RETINA_PROLIF	rs62339673	-0.007	0.108	0.947
Alcohol intake frequency	Proliferative diabetic retinopathy	ukb-b-5779	finn-b-DM_RETINA_PROLIF	rs62466318	0.019	0.107	0.858

Alcohol intake frequency	Proliferative diabetic retinopathy	ukb-b-5779	finn-b-DM_RETINA_PROLIF	rs650558	-0.005	0.108	0.966
Alcohol intake frequency	Proliferative diabetic retinopathy	ukb-b-5779	finn-b-DM_RETINA_PROLIF	rs6727281	-0.007	0.108	0.952
Alcohol intake frequency	Proliferative diabetic retinopathy	ukb-b-5779	finn-b-DM_RETINA_PROLIF	rs6943160	-0.008	0.108	0.942
Alcohol intake frequency	Proliferative diabetic retinopathy	ukb-b-5779	finn-b-DM_RETINA_PROLIF	rs71651683	0.002	0.108	0.984
Alcohol intake frequency	Proliferative diabetic retinopathy	ukb-b-5779	finn-b-DM_RETINA_PROLIF	rs72769229	0.001	0.108	0.995
Alcohol intake frequency	Proliferative diabetic retinopathy	ukb-b-5779	finn-b-DM_RETINA_PROLIF	rs72787062	0.021	0.107	0.843
Alcohol intake frequency	Proliferative diabetic retinopathy	ukb-b-5779	finn-b-DM_RETINA_PROLIF	rs728538	0.013	0.108	0.905
Alcohol intake frequency	Proliferative diabetic retinopathy	ukb-b-5779	finn-b-DM_RETINA_PROLIF	rs7298932	-0.001	0.108	0.991

Alcohol intake frequency	Proliferative diabetic retinopathy	ukb-b-5779	finn-b-DM_RETINA_PROLIF	rs7302200	0.023	0.106	0.827
Alcohol intake frequency	Proliferative diabetic retinopathy	ukb-b-5779	finn-b-DM_RETINA_PROLIF	rs73050128	0.006	0.109	0.957
Alcohol intake frequency	Proliferative diabetic retinopathy	ukb-b-5779	finn-b-DM_RETINA_PROLIF	rs7330939	0.012	0.108	0.912
Alcohol intake frequency	Proliferative diabetic retinopathy	ukb-b-5779	finn-b-DM_RETINA_PROLIF	rs74679146	-0.006	0.108	0.956
Alcohol intake frequency	Proliferative diabetic retinopathy	ukb-b-5779	finn-b-DM_RETINA_PROLIF	rs7514579	-0.009	0.107	0.937
Alcohol intake frequency	Proliferative diabetic retinopathy	ukb-b-5779	finn-b-DM_RETINA_PROLIF	rs76082653	0.005	0.108	0.967
Alcohol intake frequency	Proliferative diabetic retinopathy	ukb-b-5779	finn-b-DM_RETINA_PROLIF	rs7610856	-0.019	0.107	0.862
Alcohol intake frequency	Proliferative diabetic retinopathy	ukb-b-5779	finn-b-DM_RETINA_PROLIF	rs780094	0.035	0.111	0.751

Alcohol intake frequency	Proliferative diabetic retinopathy	ukb-b-5779	finn-b-DM_RETINA_PROLIF	rs780569	0.004	0.108	0.970
Alcohol intake frequency	Proliferative diabetic retinopathy	ukb-b-5779	finn-b-DM_RETINA_PROLIF	rs80292319	0.016	0.108	0.882
Alcohol intake frequency	Proliferative diabetic retinopathy	ukb-b-5779	finn-b-DM_RETINA_PROLIF	rs8043563	0.017	0.108	0.871
Alcohol intake frequency	Proliferative diabetic retinopathy	ukb-b-5779	finn-b-DM_RETINA_PROLIF	rs838145	0.015	0.108	0.890
Alcohol intake frequency	Proliferative diabetic retinopathy	ukb-b-5779	finn-b-DM_RETINA_PROLIF	rs8614	0.012	0.108	0.911
Alcohol intake frequency	Proliferative diabetic retinopathy	ukb-b-5779	finn-b-DM_RETINA_PROLIF	rs9349379	0.002	0.109	0.989
Alcohol intake frequency	Proliferative diabetic retinopathy	ukb-b-5779	finn-b-DM_RETINA_PROLIF	rs9372625	-0.001	0.109	0.990
Alcohol intake frequency	Proliferative diabetic retinopathy	ukb-b-5779	finn-b-DM_RETINA_PROLIF	rs9403297	-0.008	0.108	0.944

Alcohol intake frequency	Proliferative diabetic retinopathy	ukb-b-5779	finn-b-DM_RETINA_PROLIF	rs9648478	-0.011	0.107	0.918
Alcohol intake frequency	Proliferative diabetic retinopathy	ukb-b-5779	finn-b-DM_RETINA_PROLIF	rs9814516	0.000	0.109	0.997
Alcohol intake frequency	Proliferative diabetic retinopathy	ukb-b-5779	finn-b-DM_RETINA_PROLIF	rs9829192	0.007	0.108	0.951
Alcohol intake frequency	Proliferative diabetic retinopathy	ukb-b-5779	finn-b-DM_RETINA_PROLIF	rs9906502	0.004	0.109	0.970
Alcohol intake frequency	Proliferative diabetic retinopathy	ukb-b-5779	finn-b-DM_RETINA_PROLIF	All	0.003	0.107	0.981
Alcoholic drinks per week	Background diabetic retinopathy	ieu-b-73	finn-b-DM_BCKGRND_RETINA	rs10085696	0.404	0.491	0.411
Alcoholic drinks per week	Background diabetic retinopathy	ieu-b-73	finn-b-DM_BCKGRND_RETINA	rs11860773	0.477	0.492	0.333
Alcoholic drinks per week	Background diabetic retinopathy	ieu-b-73	finn-b-DM_BCKGRND_RETINA	rs1229984	0.326	0.514	0.526

Alcoholic drinks per week	Background diabetic retinopathy	ieu-b-73	finn-b-DM_BCKGRND_RETINA	rs1260326	0.366	0.505	0.468
Alcoholic drinks per week	Background diabetic retinopathy	ieu-b-73	finn-b-DM_BCKGRND_RETINA	rs13107325	0.416	0.486	0.392
Alcoholic drinks per week	Background diabetic retinopathy	ieu-b-73	finn-b-DM_BCKGRND_RETINA	rs13332432	0.402	0.489	0.411
Alcoholic drinks per week	Background diabetic retinopathy	ieu-b-73	finn-b-DM_BCKGRND_RETINA	rs1387766	0.578	0.459	0.208
Alcoholic drinks per week	Background diabetic retinopathy	ieu-b-73	finn-b-DM_BCKGRND_RETINA	rs153106	0.434	0.494	0.381
Alcoholic drinks per week	Background diabetic retinopathy	ieu-b-73	finn-b-DM_BCKGRND_RETINA	rs16854020	0.438	0.491	0.372
Alcoholic drinks per week	Background diabetic retinopathy	ieu-b-73	finn-b-DM_BCKGRND_RETINA	rs17542254	0.433	0.492	0.378
Alcoholic drinks per week	Background diabetic retinopathy	ieu-b-73	finn-b-DM_BCKGRND_RETINA	rs2049045	0.574	0.460	0.212

Alcoholic drinks per week	Background diabetic retinopathy	ieu-b-73	finn-b-DM_BCKGRND_RETINA	rs2299409	0.385	0.483	0.425
Alcoholic drinks per week	Background diabetic retinopathy	ieu-b-73	finn-b-DM_BCKGRND_RETINA	rs28601761	0.482	0.491	0.326
Alcoholic drinks per week	Background diabetic retinopathy	ieu-b-73	finn-b-DM_BCKGRND_RETINA	rs28680958	0.461	0.492	0.349
Alcoholic drinks per week	Background diabetic retinopathy	ieu-b-73	finn-b-DM_BCKGRND_RETINA	rs28712821	0.311	0.512	0.544
Alcoholic drinks per week	Background diabetic retinopathy	ieu-b-73	finn-b-DM_BCKGRND_RETINA	rs28732378	0.371	0.489	0.448
Alcoholic drinks per week	Background diabetic retinopathy	ieu-b-73	finn-b-DM_BCKGRND_RETINA	rs28929474	0.378	0.488	0.439
Alcoholic drinks per week	Background diabetic retinopathy	ieu-b-73	finn-b-DM_BCKGRND_RETINA	rs331939	0.539	0.479	0.260
Alcoholic drinks per week	Background diabetic retinopathy	ieu-b-73	finn-b-DM_BCKGRND_RETINA	rs34121753	0.555	0.474	0.242

Alcoholic drinks per week	Background diabetic retinopathy	ieu-b-73	finn-b-DM_BCKGRND_RETINA	rs4309187	0.520	0.485	0.284
Alcoholic drinks per week	Background diabetic retinopathy	ieu-b-73	finn-b-DM_BCKGRND_RETINA	rs4752999	0.513	0.489	0.294
Alcoholic drinks per week	Background diabetic retinopathy	ieu-b-73	finn-b-DM_BCKGRND_RETINA	rs494904	0.588	0.480	0.221
Alcoholic drinks per week	Background diabetic retinopathy	ieu-b-73	finn-b-DM_BCKGRND_RETINA	rs55872084	0.431	0.490	0.378
Alcoholic drinks per week	Background diabetic retinopathy	ieu-b-73	finn-b-DM_BCKGRND_RETINA	rs55932213	0.485	0.491	0.323
Alcoholic drinks per week	Background diabetic retinopathy	ieu-b-73	finn-b-DM_BCKGRND_RETINA	rs6106989	0.435	0.491	0.376
Alcoholic drinks per week	Background diabetic retinopathy	ieu-b-73	finn-b-DM_BCKGRND_RETINA	rs6739804	0.483	0.492	0.327
Alcoholic drinks per week	Background diabetic retinopathy	ieu-b-73	finn-b-DM_BCKGRND_RETINA	rs676388	0.216	0.439	0.623

Alcoholic drinks per week	Background diabetic retinopathy	ieu-b-73	finn-b-DM_BCKGRND_RETINA	rs6969458	0.515	0.489	0.292
Alcoholic drinks per week	Background diabetic retinopathy	ieu-b-73	finn-b-DM_BCKGRND_RETINA	rs75120545	0.561	0.482	0.245
Alcoholic drinks per week	Background diabetic retinopathy	ieu-b-73	finn-b-DM_BCKGRND_RETINA	rs76640332	0.506	0.488	0.300
Alcoholic drinks per week	Background diabetic retinopathy	ieu-b-73	finn-b-DM_BCKGRND_RETINA	rs78234152	0.464	0.502	0.356
Alcoholic drinks per week	Background diabetic retinopathy	ieu-b-73	finn-b-DM_BCKGRND_RETINA	rs79616692	0.442	0.493	0.371
Alcoholic drinks per week	Background diabetic retinopathy	ieu-b-73	finn-b-DM_BCKGRND_RETINA	rs962961	0.484	0.492	0.325
Alcoholic drinks per week	Background diabetic retinopathy	ieu-b-73	finn-b-DM_BCKGRND_RETINA	All	0.455	0.480	0.343
Alcoholic drinks per week	Diabetic retinopathy	ieu-b-73	finn-b-DM_RETINOPATHY_EXMORE	rs10085696	-0.012	0.172	0.942

Alcoholic drinks per week	Diabetic retinopathy	ieu-b-73	finn-b-DM_RETINOPATHY_EXMORE	rs11860773	-0.037	0.174	0.830
Alcoholic drinks per week	Diabetic retinopathy	ieu-b-73	finn-b-DM_RETINOPATHY_EXMORE	rs1229984	0.045	0.178	0.799
Alcoholic drinks per week	Diabetic retinopathy	ieu-b-73	finn-b-DM_RETINOPATHY_EXMORE	rs1260326	-0.140	0.171	0.412
Alcoholic drinks per week	Diabetic retinopathy	ieu-b-73	finn-b-DM_RETINOPATHY_EXMORE	rs13107325	-0.019	0.170	0.911
Alcoholic drinks per week	Diabetic retinopathy	ieu-b-73	finn-b-DM_RETINOPATHY_EXMORE	rs13332432	-0.011	0.171	0.948
Alcoholic drinks per week	Diabetic retinopathy	ieu-b-73	finn-b-DM_RETINOPATHY_EXMORE	rs1387766	-0.045	0.173	0.796
Alcoholic drinks per week	Diabetic retinopathy	ieu-b-73	finn-b-DM_RETINOPATHY_EXMORE	rs153106	-0.090	0.166	0.587
Alcoholic drinks per week	Diabetic retinopathy	ieu-b-73	finn-b-DM_RETINOPATHY_EXMORE	rs16854020	-0.070	0.168	0.677

Alcoholic drinks per week	Diabetic retinopathy	ieu-b-73	finn-b-DM_RETINOPATHY_EXMORE	rs17542254	-0.034	0.174	0.846
Alcoholic drinks per week	Diabetic retinopathy	ieu-b-73	finn-b-DM_RETINOPATHY_EXMORE	rs2049045	-0.038	0.173	0.825
Alcoholic drinks per week	Diabetic retinopathy	ieu-b-73	finn-b-DM_RETINOPATHY_EXMORE	rs2299409	-0.057	0.172	0.740
Alcoholic drinks per week	Diabetic retinopathy	ieu-b-73	finn-b-DM_RETINOPATHY_EXMORE	rs28601761	-0.005	0.168	0.976
Alcoholic drinks per week	Diabetic retinopathy	ieu-b-73	finn-b-DM_RETINOPATHY_EXMORE	rs28680958	-0.041	0.174	0.814
Alcoholic drinks per week	Diabetic retinopathy	ieu-b-73	finn-b-DM_RETINOPATHY_EXMORE	rs28712821	-0.092	0.181	0.611
Alcoholic drinks per week	Diabetic retinopathy	ieu-b-73	finn-b-DM_RETINOPATHY_EXMORE	rs28732378	-0.033	0.175	0.850
Alcoholic drinks per week	Diabetic retinopathy	ieu-b-73	finn-b-DM_RETINOPATHY_EXMORE	rs28929474	-0.071	0.171	0.677

Alcoholic drinks per week	Diabetic retinopathy	ieu-b-73	finn-b-DM_RETINOPATHY_EXMORE	rs331939	-0.021	0.172	0.901
Alcoholic drinks per week	Diabetic retinopathy	ieu-b-73	finn-b-DM_RETINOPATHY_EXMORE	rs34121753	-0.015	0.171	0.930
Alcoholic drinks per week	Diabetic retinopathy	ieu-b-73	finn-b-DM_RETINOPATHY_EXMORE	rs4309187	-0.080	0.166	0.628
Alcoholic drinks per week	Diabetic retinopathy	ieu-b-73	finn-b-DM_RETINOPATHY_EXMORE	rs4752999	-0.027	0.174	0.877
Alcoholic drinks per week	Diabetic retinopathy	ieu-b-73	finn-b-DM_RETINOPATHY_EXMORE	rs494904	-0.006	0.172	0.974
Alcoholic drinks per week	Diabetic retinopathy	ieu-b-73	finn-b-DM_RETINOPATHY_EXMORE	rs55872084	-0.045	0.173	0.794
Alcoholic drinks per week	Diabetic retinopathy	ieu-b-73	finn-b-DM_RETINOPATHY_EXMORE	rs55932213	-0.046	0.174	0.792
Alcoholic drinks per week	Diabetic retinopathy	ieu-b-73	finn-b-DM_RETINOPATHY_EXMORE	rs6106989	-0.054	0.172	0.754

Alcoholic drinks per week	Diabetic retinopathy	ieu-b-73	finn-b-DM_RETINOPATHY_EXMORE	rs6739804	-0.009	0.170	0.959
Alcoholic drinks per week	Diabetic retinopathy	ieu-b-73	finn-b-DM_RETINOPATHY_EXMORE	rs676388	-0.075	0.172	0.664
Alcoholic drinks per week	Diabetic retinopathy	ieu-b-73	finn-b-DM_RETINOPATHY_EXMORE	rs6969458	-0.025	0.173	0.885
Alcoholic drinks per week	Diabetic retinopathy	ieu-b-73	finn-b-DM_RETINOPATHY_EXMORE	rs75120545	0.010	0.167	0.953
Alcoholic drinks per week	Diabetic retinopathy	ieu-b-73	finn-b-DM_RETINOPATHY_EXMORE	rs76640332	-0.025	0.173	0.886
Alcoholic drinks per week	Diabetic retinopathy	ieu-b-73	finn-b-DM_RETINOPATHY_EXMORE	rs78234152	-0.080	0.175	0.649
Alcoholic drinks per week	Diabetic retinopathy	ieu-b-73	finn-b-DM_RETINOPATHY_EXMORE	rs79616692	-0.025	0.173	0.884
Alcoholic drinks per week	Diabetic retinopathy	ieu-b-73	finn-b-DM_RETINOPATHY_EXMORE	rs962961	-0.046	0.174	0.790

Alcoholic drinks per week	Diabetic retinopathy	ieu-b-73	finn-b-DM_RETINOPATHY_EXMORE	All	-0.040	0.169	0.814
Alcoholic drinks per week	Proliferative diabetic retinopathy	ieu-b-73	finn-b-DM_RETINA_PROLIF	rs10085696	-0.024	0.213	0.909
Alcoholic drinks per week	Proliferative diabetic retinopathy	ieu-b-73	finn-b-DM_RETINA_PROLIF	rs11860773	-0.069	0.215	0.747
Alcoholic drinks per week	Proliferative diabetic retinopathy	ieu-b-73	finn-b-DM_RETINA_PROLIF	rs1229984	0.052	0.219	0.811
Alcoholic drinks per week	Proliferative diabetic retinopathy	ieu-b-73	finn-b-DM_RETINA_PROLIF	rs1260326	-0.160	0.213	0.453
Alcoholic drinks per week	Proliferative diabetic retinopathy	ieu-b-73	finn-b-DM_RETINA_PROLIF	rs13107325	-0.048	0.214	0.821
Alcoholic drinks per week	Proliferative diabetic retinopathy	ieu-b-73	finn-b-DM_RETINA_PROLIF	rs13332432	-0.037	0.214	0.864
Alcoholic drinks per week	Proliferative diabetic retinopathy	ieu-b-73	finn-b-DM_RETINA_PROLIF	rs1387766	-0.056	0.215	0.794

Alcoholic drinks per week	Proliferative diabetic retinopathy	ieu-b-73	finn-b-DM_RETINA_PROLIF	rs153106	-0.141	0.208	0.496
Alcoholic drinks per week	Proliferative diabetic retinopathy	ieu-b-73	finn-b-DM_RETINA_PROLIF	rs16854020	-0.099	0.207	0.632
Alcoholic drinks per week	Proliferative diabetic retinopathy	ieu-b-73	finn-b-DM_RETINA_PROLIF	rs17542254	-0.051	0.215	0.813
Alcoholic drinks per week	Proliferative diabetic retinopathy	ieu-b-73	finn-b-DM_RETINA_PROLIF	rs2049045	-0.020	0.207	0.924
Alcoholic drinks per week	Proliferative diabetic retinopathy	ieu-b-73	finn-b-DM_RETINA_PROLIF	rs2299409	-0.089	0.211	0.673
Alcoholic drinks per week	Proliferative diabetic retinopathy	ieu-b-73	finn-b-DM_RETINA_PROLIF	rs28601761	0.006	0.207	0.976
Alcoholic drinks per week	Proliferative diabetic retinopathy	ieu-b-73	finn-b-DM_RETINA_PROLIF	rs28680958	-0.033	0.212	0.876
Alcoholic drinks per week	Proliferative diabetic retinopathy	ieu-b-73	finn-b-DM_RETINA_PROLIF	rs28712821	-0.142	0.222	0.521

Alcoholic drinks per week	Proliferative diabetic retinopathy	ieu-b-73	finn-b-DM_RETINA_PROLIF	rs28732378	-0.058	0.217	0.788
Alcoholic drinks per week	Proliferative diabetic retinopathy	ieu-b-73	finn-b-DM_RETINA_PROLIF	rs28929474	-0.042	0.215	0.847
Alcoholic drinks per week	Proliferative diabetic retinopathy	ieu-b-73	finn-b-DM_RETINA_PROLIF	rs331939	-0.050	0.215	0.815
Alcoholic drinks per week	Proliferative diabetic retinopathy	ieu-b-73	finn-b-DM_RETINA_PROLIF	rs34121753	0.000	0.207	0.998
Alcoholic drinks per week	Proliferative diabetic retinopathy	ieu-b-73	finn-b-DM_RETINA_PROLIF	rs4309187	-0.092	0.211	0.663
Alcoholic drinks per week	Proliferative diabetic retinopathy	ieu-b-73	finn-b-DM_RETINA_PROLIF	rs4752999	-0.050	0.216	0.816
Alcoholic drinks per week	Proliferative diabetic retinopathy	ieu-b-73	finn-b-DM_RETINA_PROLIF	rs494904	-0.047	0.217	0.830
Alcoholic drinks per week	Proliferative diabetic retinopathy	ieu-b-73	finn-b-DM_RETINA_PROLIF	rs55872084	-0.061	0.215	0.776

Alcoholic drinks per week	Proliferative diabetic retinopathy	ieu-b-73	finn-b-DM_RETINA_PROLIF	rs55932213	-0.057	0.215	0.791
Alcoholic drinks per week	Proliferative diabetic retinopathy	ieu-b-73	finn-b-DM_RETINA_PROLIF	rs6106989	-0.080	0.213	0.707
Alcoholic drinks per week	Proliferative diabetic retinopathy	ieu-b-73	finn-b-DM_RETINA_PROLIF	rs6739804	-0.052	0.215	0.810
Alcoholic drinks per week	Proliferative diabetic retinopathy	ieu-b-73	finn-b-DM_RETINA_PROLIF	rs676388	-0.082	0.216	0.703
Alcoholic drinks per week	Proliferative diabetic retinopathy	ieu-b-73	finn-b-DM_RETINA_PROLIF	rs6969458	-0.052	0.216	0.810
Alcoholic drinks per week	Proliferative diabetic retinopathy	ieu-b-73	finn-b-DM_RETINA_PROLIF	rs75120545	-0.031	0.214	0.887
Alcoholic drinks per week	Proliferative diabetic retinopathy	ieu-b-73	finn-b-DM_RETINA_PROLIF	rs76640332	-0.050	0.215	0.817
Alcoholic drinks per week	Proliferative diabetic retinopathy	ieu-b-73	finn-b-DM_RETINA_PROLIF	rs78234152	-0.089	0.218	0.684

Alcoholic drinks per week	Proliferative diabetic retinopathy	ieu-b-73	finn-b-DM_RETINA_PROLIF	rs79616692	-0.086	0.213	0.687
Alcoholic drinks per week	Proliferative diabetic retinopathy	ieu-b-73	finn-b-DM_RETINA_PROLIF	rs962961	-0.055	0.215	0.797
Alcoholic drinks per week	Proliferative diabetic retinopathy	ieu-b-73	finn-b-DM_RETINA_PROLIF	All	-0.059	0.210	0.779
Body mass index	Background diabetic retinopathy	ukb-a-248	finn-b-DM_BCKGRND_RETINA	rs10100245	0.464	0.119	0.000
Body mass index	Background diabetic retinopathy	ukb-a-248	finn-b-DM_BCKGRND_RETINA	rs10187101	0.460	0.119	0.000
Body mass index	Background diabetic retinopathy	ukb-a-248	finn-b-DM_BCKGRND_RETINA	rs10404726	0.464	0.119	0.000
Body mass index	Background diabetic retinopathy	ukb-a-248	finn-b-DM_BCKGRND_RETINA	rs10465231	0.470	0.119	0.000
Body mass index	Background diabetic retinopathy	ukb-a-248	finn-b-DM_BCKGRND_RETINA	rs1064213	0.469	0.119	0.000

Body mass index	Background diabetic retinopathy	ukb-a-248	finn-b-DM_BCKGRND_RETINA	rs10788493	0.453	0.118	0.000
Body mass index	Background diabetic retinopathy	ukb-a-248	finn-b-DM_BCKGRND_RETINA	rs10803762	0.471	0.119	0.000
Body mass index	Background diabetic retinopathy	ukb-a-248	finn-b-DM_BCKGRND_RETINA	rs10805383	0.465	0.119	0.000
Body mass index	Background diabetic retinopathy	ukb-a-248	finn-b-DM_BCKGRND_RETINA	rs10865612	0.478	0.119	0.000
Body mass index	Background diabetic retinopathy	ukb-a-248	finn-b-DM_BCKGRND_RETINA	rs10898330	0.465	0.119	0.000
Body mass index	Background diabetic retinopathy	ukb-a-248	finn-b-DM_BCKGRND_RETINA	rs10938397	0.438	0.118	0.000
Body mass index	Background diabetic retinopathy	ukb-a-248	finn-b-DM_BCKGRND_RETINA	rs10995427	0.467	0.119	0.000
Body mass index	Background diabetic retinopathy	ukb-a-248	finn-b-DM_BCKGRND_RETINA	rs11012732	0.448	0.118	0.000

Body mass index	Background diabetic retinopathy	ukb-a-248	finn-b-DM_BCKGRND_RETINA	rs11078883	0.459	0.119	0.000
Body mass index	Background diabetic retinopathy	ukb-a-248	finn-b-DM_BCKGRND_RETINA	rs11084554	0.462	0.119	0.000
Body mass index	Background diabetic retinopathy	ukb-a-248	finn-b-DM_BCKGRND_RETINA	rs11099020	0.455	0.118	0.000
Body mass index	Background diabetic retinopathy	ukb-a-248	finn-b-DM_BCKGRND_RETINA	rs11150745	0.462	0.119	0.000
Body mass index	Background diabetic retinopathy	ukb-a-248	finn-b-DM_BCKGRND_RETINA	rs111640872	0.474	0.119	0.000
Body mass index	Background diabetic retinopathy	ukb-a-248	finn-b-DM_BCKGRND_RETINA	rs11223641	0.463	0.119	0.000
Body mass index	Background diabetic retinopathy	ukb-a-248	finn-b-DM_BCKGRND_RETINA	rs112520079	0.462	0.119	0.000
Body mass index	Background diabetic retinopathy	ukb-a-248	finn-b-DM_BCKGRND_RETINA	rs11264489	0.466	0.119	0.000

Body mass index	Background diabetic retinopathy	ukb-a-248	finn-b-DM_BCKGRND_RETINA	rs112693590	0.467	0.119	0.000
Body mass index	Background diabetic retinopathy	ukb-a-248	finn-b-DM_BCKGRND_RETINA	rs1127100	0.476	0.118	0.000
Body mass index	Background diabetic retinopathy	ukb-a-248	finn-b-DM_BCKGRND_RETINA	rs113182412	0.470	0.119	0.000
Body mass index	Background diabetic retinopathy	ukb-a-248	finn-b-DM_BCKGRND_RETINA	rs113230003	0.464	0.119	0.000
Body mass index	Background diabetic retinopathy	ukb-a-248	finn-b-DM_BCKGRND_RETINA	rs113603865	0.460	0.119	0.000
Body mass index	Background diabetic retinopathy	ukb-a-248	finn-b-DM_BCKGRND_RETINA	rs11515071	0.453	0.119	0.000
Body mass index	Background diabetic retinopathy	ukb-a-248	finn-b-DM_BCKGRND_RETINA	rs11642015	0.430	0.122	0.000
Body mass index	Background diabetic retinopathy	ukb-a-248	finn-b-DM_BCKGRND_RETINA	rs11650012	0.464	0.119	0.000

Body mass index	Background diabetic retinopathy	ukb-a-248	finn-b-DM_BCKGRND_RETINA	rs11655587	0.454	0.118	0.000
Body mass index	Background diabetic retinopathy	ukb-a-248	finn-b-DM_BCKGRND_RETINA	rs11742930	0.457	0.118	0.000
Body mass index	Background diabetic retinopathy	ukb-a-248	finn-b-DM_BCKGRND_RETINA	rs11757278	0.474	0.118	0.000
Body mass index	Background diabetic retinopathy	ukb-a-248	finn-b-DM_BCKGRND_RETINA	rs11761411	0.460	0.119	0.000
Body mass index	Background diabetic retinopathy	ukb-a-248	finn-b-DM_BCKGRND_RETINA	rs117632017	0.468	0.119	0.000
Body mass index	Background diabetic retinopathy	ukb-a-248	finn-b-DM_BCKGRND_RETINA	rs11782074	0.465	0.119	0.000
Body mass index	Background diabetic retinopathy	ukb-a-248	finn-b-DM_BCKGRND_RETINA	rs11856579	0.481	0.118	0.000
Body mass index	Background diabetic retinopathy	ukb-a-248	finn-b-DM_BCKGRND_RETINA	rs12024554	0.471	0.119	0.000

Body mass index	Background diabetic retinopathy	ukb-a-248	finn-b-DM_BCKGRND_RETINA	rs12042959	0.457	0.118	0.000
Body mass index	Background diabetic retinopathy	ukb-a-248	finn-b-DM_BCKGRND_RETINA	rs12049202	0.452	0.118	0.000
Body mass index	Background diabetic retinopathy	ukb-a-248	finn-b-DM_BCKGRND_RETINA	rs12140153	0.452	0.118	0.000
Body mass index	Background diabetic retinopathy	ukb-a-248	finn-b-DM_BCKGRND_RETINA	rs12144626	0.465	0.119	0.000
Body mass index	Background diabetic retinopathy	ukb-a-248	finn-b-DM_BCKGRND_RETINA	rs12477385	0.458	0.119	0.000
Body mass index	Background diabetic retinopathy	ukb-a-248	finn-b-DM_BCKGRND_RETINA	rs12479357	0.466	0.119	0.000
Body mass index	Background diabetic retinopathy	ukb-a-248	finn-b-DM_BCKGRND_RETINA	rs12614861	0.474	0.118	0.000
Body mass index	Background diabetic retinopathy	ukb-a-248	finn-b-DM_BCKGRND_RETINA	rs12622280	0.464	0.119	0.000

Body mass index	Background diabetic retinopathy	ukb-a-248	finn-b-DM_BCKGRND_RETINA	rs12662900	0.461	0.119	0.000
Body mass index	Background diabetic retinopathy	ukb-a-248	finn-b-DM_BCKGRND_RETINA	rs12679106	0.467	0.119	0.000
Body mass index	Background diabetic retinopathy	ukb-a-248	finn-b-DM_BCKGRND_RETINA	rs1286138	0.466	0.119	0.000
Body mass index	Background diabetic retinopathy	ukb-a-248	finn-b-DM_BCKGRND_RETINA	rs12877270	0.474	0.118	0.000
Body mass index	Background diabetic retinopathy	ukb-a-248	finn-b-DM_BCKGRND_RETINA	rs12881629	0.466	0.119	0.000
Body mass index	Background diabetic retinopathy	ukb-a-248	finn-b-DM_BCKGRND_RETINA	rs12885458	0.461	0.119	0.000
Body mass index	Background diabetic retinopathy	ukb-a-248	finn-b-DM_BCKGRND_RETINA	rs1296328	0.483	0.118	0.000
Body mass index	Background diabetic retinopathy	ukb-a-248	finn-b-DM_BCKGRND_RETINA	rs12977259	0.466	0.119	0.000

Body mass index	Background diabetic retinopathy	ukb-a-248	finn-b-DM_BCKGRND_RETINA	rs12992672	0.462	0.120	0.000
Body mass index	Background diabetic retinopathy	ukb-a-248	finn-b-DM_BCKGRND_RETINA	rs13062093	0.457	0.118	0.000
Body mass index	Background diabetic retinopathy	ukb-a-248	finn-b-DM_BCKGRND_RETINA	rs13076052	0.460	0.119	0.000
Body mass index	Background diabetic retinopathy	ukb-a-248	finn-b-DM_BCKGRND_RETINA	rs13135092	0.471	0.119	0.000
Body mass index	Background diabetic retinopathy	ukb-a-248	finn-b-DM_BCKGRND_RETINA	rs13174863	0.458	0.119	0.000
Body mass index	Background diabetic retinopathy	ukb-a-248	finn-b-DM_BCKGRND_RETINA	rs1320903	0.469	0.119	0.000
Body mass index	Background diabetic retinopathy	ukb-a-248	finn-b-DM_BCKGRND_RETINA	rs1327259	0.472	0.118	0.000
Body mass index	Background diabetic retinopathy	ukb-a-248	finn-b-DM_BCKGRND_RETINA	rs1342391	0.462	0.119	0.000

Body mass index	Background diabetic retinopathy	ukb-a-248	finn-b-DM_BCKGRND_RETINA	rs13427822	0.472	0.119	0.000
Body mass index	Background diabetic retinopathy	ukb-a-248	finn-b-DM_BCKGRND_RETINA	rs1411432	0.462	0.119	0.000
Body mass index	Background diabetic retinopathy	ukb-a-248	finn-b-DM_BCKGRND_RETINA	rs1412239	0.464	0.119	0.000
Body mass index	Background diabetic retinopathy	ukb-a-248	finn-b-DM_BCKGRND_RETINA	rs1441264	0.481	0.118	0.000
Body mass index	Background diabetic retinopathy	ukb-a-248	finn-b-DM_BCKGRND_RETINA	rs1446585	0.467	0.119	0.000
Body mass index	Background diabetic retinopathy	ukb-a-248	finn-b-DM_BCKGRND_RETINA	rs1458156	0.468	0.119	0.000
Body mass index	Background diabetic retinopathy	ukb-a-248	finn-b-DM_BCKGRND_RETINA	rs1477290	0.462	0.119	0.000
Body mass index	Background diabetic retinopathy	ukb-a-248	finn-b-DM_BCKGRND_RETINA	rs147730268	0.466	0.119	0.000

Body mass index	Background diabetic retinopathy	ukb-a-248	finn-b-DM_BCKGRND_RETINA	rs1582931	0.464	0.119	0.000
Body mass index	Background diabetic retinopathy	ukb-a-248	finn-b-DM_BCKGRND_RETINA	rs16846140	0.472	0.119	0.000
Body mass index	Background diabetic retinopathy	ukb-a-248	finn-b-DM_BCKGRND_RETINA	rs16916303	0.464	0.119	0.000
Body mass index	Background diabetic retinopathy	ukb-a-248	finn-b-DM_BCKGRND_RETINA	rs16975459	0.465	0.119	0.000
Body mass index	Background diabetic retinopathy	ukb-a-248	finn-b-DM_BCKGRND_RETINA	rs17014332	0.470	0.119	0.000
Body mass index	Background diabetic retinopathy	ukb-a-248	finn-b-DM_BCKGRND_RETINA	rs17024393	0.474	0.119	0.000
Body mass index	Background diabetic retinopathy	ukb-a-248	finn-b-DM_BCKGRND_RETINA	rs17058884	0.467	0.119	0.000
Body mass index	Background diabetic retinopathy	ukb-a-248	finn-b-DM_BCKGRND_RETINA	rs17085463	0.471	0.119	0.000

Body mass index	Background diabetic retinopathy	ukb-a-248	finn-b-DM_BCKGRND_RETINA	rs17149254	0.477	0.118	0.000
Body mass index	Background diabetic retinopathy	ukb-a-248	finn-b-DM_BCKGRND_RETINA	rs17342242	0.468	0.119	0.000
Body mass index	Background diabetic retinopathy	ukb-a-248	finn-b-DM_BCKGRND_RETINA	rs17399739	0.464	0.119	0.000
Body mass index	Background diabetic retinopathy	ukb-a-248	finn-b-DM_BCKGRND_RETINA	rs17716502	0.469	0.119	0.000
Body mass index	Background diabetic retinopathy	ukb-a-248	finn-b-DM_BCKGRND_RETINA	rs1788808	0.464	0.119	0.000
Body mass index	Background diabetic retinopathy	ukb-a-248	finn-b-DM_BCKGRND_RETINA	rs1805123	0.462	0.119	0.000
Body mass index	Background diabetic retinopathy	ukb-a-248	finn-b-DM_BCKGRND_RETINA	rs1884897	0.476	0.119	0.000
Body mass index	Background diabetic retinopathy	ukb-a-248	finn-b-DM_BCKGRND_RETINA	rs1901241	0.468	0.119	0.000

Body mass index	Background diabetic retinopathy	ukb-a-248	finn-b-DM_BCKGRND_RETINA	rs1919243	0.464	0.119	0.000
Body mass index	Background diabetic retinopathy	ukb-a-248	finn-b-DM_BCKGRND_RETINA	rs1941706	0.469	0.119	0.000
Body mass index	Background diabetic retinopathy	ukb-a-248	finn-b-DM_BCKGRND_RETINA	rs1949204	0.463	0.119	0.000
Body mass index	Background diabetic retinopathy	ukb-a-248	finn-b-DM_BCKGRND_RETINA	rs2035806	0.468	0.119	0.000
Body mass index	Background diabetic retinopathy	ukb-a-248	finn-b-DM_BCKGRND_RETINA	rs2046002	0.456	0.118	0.000
Body mass index	Background diabetic retinopathy	ukb-a-248	finn-b-DM_BCKGRND_RETINA	rs2121058	0.447	0.118	0.000
Body mass index	Background diabetic retinopathy	ukb-a-248	finn-b-DM_BCKGRND_RETINA	rs2135745	0.472	0.118	0.000
Body mass index	Background diabetic retinopathy	ukb-a-248	finn-b-DM_BCKGRND_RETINA	rs2155869	0.471	0.119	0.000

Body mass index	Background diabetic retinopathy	ukb-a-248	finn-b-DM_BCKGRND_RETINA	rs215634	0.464	0.119	0.000
Body mass index	Background diabetic retinopathy	ukb-a-248	finn-b-DM_BCKGRND_RETINA	rs2192649	0.464	0.119	0.000
Body mass index	Background diabetic retinopathy	ukb-a-248	finn-b-DM_BCKGRND_RETINA	rs2234458	0.464	0.119	0.000
Body mass index	Background diabetic retinopathy	ukb-a-248	finn-b-DM_BCKGRND_RETINA	rs2292238	0.489	0.116	0.000
Body mass index	Background diabetic retinopathy	ukb-a-248	finn-b-DM_BCKGRND_RETINA	rs2307111	0.454	0.119	0.000
Body mass index	Background diabetic retinopathy	ukb-a-248	finn-b-DM_BCKGRND_RETINA	rs2318543	0.454	0.118	0.000
Body mass index	Background diabetic retinopathy	ukb-a-248	finn-b-DM_BCKGRND_RETINA	rs2384054	0.452	0.119	0.000
Body mass index	Background diabetic retinopathy	ukb-a-248	finn-b-DM_BCKGRND_RETINA	rs2398861	0.475	0.119	0.000

Body mass index	Background diabetic retinopathy	ukb-a-248	finn-b-DM_BCKGRND_RETINA	rs241460	0.469	0.119	0.000
Body mass index	Background diabetic retinopathy	ukb-a-248	finn-b-DM_BCKGRND_RETINA	rs2425857	0.462	0.119	0.000
Body mass index	Background diabetic retinopathy	ukb-a-248	finn-b-DM_BCKGRND_RETINA	rs2439823	0.477	0.119	0.000
Body mass index	Background diabetic retinopathy	ukb-a-248	finn-b-DM_BCKGRND_RETINA	rs2450254	0.461	0.119	0.000
Body mass index	Background diabetic retinopathy	ukb-a-248	finn-b-DM_BCKGRND_RETINA	rs2450445	0.461	0.119	0.000
Body mass index	Background diabetic retinopathy	ukb-a-248	finn-b-DM_BCKGRND_RETINA	rs245775	0.472	0.119	0.000
Body mass index	Background diabetic retinopathy	ukb-a-248	finn-b-DM_BCKGRND_RETINA	rs2470392	0.469	0.119	0.000
Body mass index	Background diabetic retinopathy	ukb-a-248	finn-b-DM_BCKGRND_RETINA	rs2474898	0.463	0.119	0.000

Body mass index	Background diabetic retinopathy	ukb-a-248	finn-b-DM_BCKGRND_RETINA	rs2482704	0.472	0.118	0.000
Body mass index	Background diabetic retinopathy	ukb-a-248	finn-b-DM_BCKGRND_RETINA	rs2606228	0.460	0.119	0.000
Body mass index	Background diabetic retinopathy	ukb-a-248	finn-b-DM_BCKGRND_RETINA	rs2616192	0.457	0.118	0.000
Body mass index	Background diabetic retinopathy	ukb-a-248	finn-b-DM_BCKGRND_RETINA	rs2678204	0.453	0.119	0.000
Body mass index	Background diabetic retinopathy	ukb-a-248	finn-b-DM_BCKGRND_RETINA	rs2711111	0.466	0.119	0.000
Body mass index	Background diabetic retinopathy	ukb-a-248	finn-b-DM_BCKGRND_RETINA	rs2725371	0.457	0.118	0.000
Body mass index	Background diabetic retinopathy	ukb-a-248	finn-b-DM_BCKGRND_RETINA	rs273505	0.462	0.119	0.000
Body mass index	Background diabetic retinopathy	ukb-a-248	finn-b-DM_BCKGRND_RETINA	rs28366156	0.447	0.117	0.000

Body mass index	Background diabetic retinopathy	ukb-a-248	finn-b-DM_BCKGRND_RETINA	rs28447555	0.464	0.119	0.000
Body mass index	Background diabetic retinopathy	ukb-a-248	finn-b-DM_BCKGRND_RETINA	rs28489620	0.469	0.119	0.000
Body mass index	Background diabetic retinopathy	ukb-a-248	finn-b-DM_BCKGRND_RETINA	rs2861685	0.468	0.119	0.000
Body mass index	Background diabetic retinopathy	ukb-a-248	finn-b-DM_BCKGRND_RETINA	rs286818	0.485	0.118	0.000
Body mass index	Background diabetic retinopathy	ukb-a-248	finn-b-DM_BCKGRND_RETINA	rs2962082	0.460	0.119	0.000
Body mass index	Background diabetic retinopathy	ukb-a-248	finn-b-DM_BCKGRND_RETINA	rs2975693	0.465	0.119	0.000
Body mass index	Background diabetic retinopathy	ukb-a-248	finn-b-DM_BCKGRND_RETINA	rs34045288	0.448	0.118	0.000
Body mass index	Background diabetic retinopathy	ukb-a-248	finn-b-DM_BCKGRND_RETINA	rs34095326	0.469	0.119	0.000

Body mass index	Background diabetic retinopathy	ukb-a-248	finn-b-DM_BCKGRND_RETINA	rs34236292	0.467	0.119	0.000
Body mass index	Background diabetic retinopathy	ukb-a-248	finn-b-DM_BCKGRND_RETINA	rs34361149	0.465	0.119	0.000
Body mass index	Background diabetic retinopathy	ukb-a-248	finn-b-DM_BCKGRND_RETINA	rs34373881	0.461	0.119	0.000
Body mass index	Background diabetic retinopathy	ukb-a-248	finn-b-DM_BCKGRND_RETINA	rs34774377	0.469	0.119	0.000
Body mass index	Background diabetic retinopathy	ukb-a-248	finn-b-DM_BCKGRND_RETINA	rs34811474	0.473	0.119	0.000
Body mass index	Background diabetic retinopathy	ukb-a-248	finn-b-DM_BCKGRND_RETINA	rs34966008	0.458	0.119	0.000
Body mass index	Background diabetic retinopathy	ukb-a-248	finn-b-DM_BCKGRND_RETINA	rs35025195	0.457	0.118	0.000
Body mass index	Background diabetic retinopathy	ukb-a-248	finn-b-DM_BCKGRND_RETINA	rs35193668	0.454	0.118	0.000

Body mass index	Background diabetic retinopathy	ukb-a-248	finn-b-DM_BCKGRND_RETINA	rs35483388	0.465	0.119	0.000
Body mass index	Background diabetic retinopathy	ukb-a-248	finn-b-DM_BCKGRND_RETINA	rs35626515	0.477	0.119	0.000
Body mass index	Background diabetic retinopathy	ukb-a-248	finn-b-DM_BCKGRND_RETINA	rs35722922	0.457	0.118	0.000
Body mass index	Background diabetic retinopathy	ukb-a-248	finn-b-DM_BCKGRND_RETINA	rs357501	0.470	0.119	0.000
Body mass index	Background diabetic retinopathy	ukb-a-248	finn-b-DM_BCKGRND_RETINA	rs35851183	0.462	0.119	0.000
Body mass index	Background diabetic retinopathy	ukb-a-248	finn-b-DM_BCKGRND_RETINA	rs35882248	0.471	0.119	0.000
Body mass index	Background diabetic retinopathy	ukb-a-248	finn-b-DM_BCKGRND_RETINA	rs36007635	0.459	0.118	0.000
Body mass index	Background diabetic retinopathy	ukb-a-248	finn-b-DM_BCKGRND_RETINA	rs362307	0.468	0.119	0.000

Body mass index	Background diabetic retinopathy	ukb-a-248	finn-b-DM_BCKGRND_RETINA	rs3759584	0.461	0.119	0.000
Body mass index	Background diabetic retinopathy	ukb-a-248	finn-b-DM_BCKGRND_RETINA	rs3802858	0.469	0.119	0.000
Body mass index	Background diabetic retinopathy	ukb-a-248	finn-b-DM_BCKGRND_RETINA	rs3803286	0.465	0.119	0.000
Body mass index	Background diabetic retinopathy	ukb-a-248	finn-b-DM_BCKGRND_RETINA	rs3810291	0.447	0.119	0.000
Body mass index	Background diabetic retinopathy	ukb-a-248	finn-b-DM_BCKGRND_RETINA	rs3843540	0.478	0.118	0.000
Body mass index	Background diabetic retinopathy	ukb-a-248	finn-b-DM_BCKGRND_RETINA	rs3844598	0.468	0.119	0.000
Body mass index	Background diabetic retinopathy	ukb-a-248	finn-b-DM_BCKGRND_RETINA	rs3861879	0.470	0.119	0.000
Body mass index	Background diabetic retinopathy	ukb-a-248	finn-b-DM_BCKGRND_RETINA	rs3897102	0.461	0.119	0.000

Body mass index	Background diabetic retinopathy	ukb-a-248	finn-b-DM_BCKGRND_RETINA	rs390192	0.460	0.119	0.000
Body mass index	Background diabetic retinopathy	ukb-a-248	finn-b-DM_BCKGRND_RETINA	rs4246657	0.470	0.119	0.000
Body mass index	Background diabetic retinopathy	ukb-a-248	finn-b-DM_BCKGRND_RETINA	rs4261944	0.468	0.119	0.000
Body mass index	Background diabetic retinopathy	ukb-a-248	finn-b-DM_BCKGRND_RETINA	rs4402589	0.455	0.119	0.000
Body mass index	Background diabetic retinopathy	ukb-a-248	finn-b-DM_BCKGRND_RETINA	rs4467770	0.464	0.119	0.000
Body mass index	Background diabetic retinopathy	ukb-a-248	finn-b-DM_BCKGRND_RETINA	rs4474229	0.469	0.119	0.000
Body mass index	Background diabetic retinopathy	ukb-a-248	finn-b-DM_BCKGRND_RETINA	rs4482463	0.468	0.119	0.000
Body mass index	Background diabetic retinopathy	ukb-a-248	finn-b-DM_BCKGRND_RETINA	rs4502882	0.466	0.119	0.000

Body mass index	Background diabetic retinopathy	ukb-a-248	finn-b-DM_BCKGRND_RETINA	rs4595495	0.472	0.118	0.000
Body mass index	Background diabetic retinopathy	ukb-a-248	finn-b-DM_BCKGRND_RETINA	rs4648450	0.459	0.119	0.000
Body mass index	Background diabetic retinopathy	ukb-a-248	finn-b-DM_BCKGRND_RETINA	rs4687770	0.471	0.119	0.000
Body mass index	Background diabetic retinopathy	ukb-a-248	finn-b-DM_BCKGRND_RETINA	rs4718964	0.471	0.119	0.000
Body mass index	Background diabetic retinopathy	ukb-a-248	finn-b-DM_BCKGRND_RETINA	rs4757144	0.468	0.119	0.000
Body mass index	Background diabetic retinopathy	ukb-a-248	finn-b-DM_BCKGRND_RETINA	rs4776970	0.468	0.119	0.000
Body mass index	Background diabetic retinopathy	ukb-a-248	finn-b-DM_BCKGRND_RETINA	rs4777541	0.459	0.119	0.000
Body mass index	Background diabetic retinopathy	ukb-a-248	finn-b-DM_BCKGRND_RETINA	rs4911382	0.467	0.119	0.000

Body mass index	Background diabetic retinopathy	ukb-a-248	finn-b-DM_BCKGRND_RETINA	rs491711	0.462	0.119	0.000
Body mass index	Background diabetic retinopathy	ukb-a-248	finn-b-DM_BCKGRND_RETINA	rs4921301	0.469	0.119	0.000
Body mass index	Background diabetic retinopathy	ukb-a-248	finn-b-DM_BCKGRND_RETINA	rs4929923	0.470	0.119	0.000
Body mass index	Background diabetic retinopathy	ukb-a-248	finn-b-DM_BCKGRND_RETINA	rs525101	0.470	0.119	0.000
Body mass index	Background diabetic retinopathy	ukb-a-248	finn-b-DM_BCKGRND_RETINA	rs539515	0.468	0.120	0.000
Body mass index	Background diabetic retinopathy	ukb-a-248	finn-b-DM_BCKGRND_RETINA	rs550974	0.471	0.119	0.000
Body mass index	Background diabetic retinopathy	ukb-a-248	finn-b-DM_BCKGRND_RETINA	rs55689274	0.466	0.119	0.000
Body mass index	Background diabetic retinopathy	ukb-a-248	finn-b-DM_BCKGRND_RETINA	rs55726687	0.461	0.119	0.000

Body mass index	Background diabetic retinopathy	ukb-a-248	finn-b-DM_BCKGRND_RETINA	rs55886426	0.469	0.119	0.000
Body mass index	Background diabetic retinopathy	ukb-a-248	finn-b-DM_BCKGRND_RETINA	rs55938344	0.473	0.118	0.000
Body mass index	Background diabetic retinopathy	ukb-a-248	finn-b-DM_BCKGRND_RETINA	rs56067609	0.464	0.119	0.000
Body mass index	Background diabetic retinopathy	ukb-a-248	finn-b-DM_BCKGRND_RETINA	rs56161855	0.479	0.118	0.000
Body mass index	Background diabetic retinopathy	ukb-a-248	finn-b-DM_BCKGRND_RETINA	rs56212061	0.465	0.119	0.000
Body mass index	Background diabetic retinopathy	ukb-a-248	finn-b-DM_BCKGRND_RETINA	rs56773984	0.466	0.119	0.000
Body mass index	Background diabetic retinopathy	ukb-a-248	finn-b-DM_BCKGRND_RETINA	rs56803094	0.466	0.119	0.000
Body mass index	Background diabetic retinopathy	ukb-a-248	finn-b-DM_BCKGRND_RETINA	rs57636386	0.474	0.119	0.000

Body mass index	Background diabetic retinopathy	ukb-a-248	finn-b-DM_BCKGRND_RETINA	rs58862095	0.462	0.119	0.000
Body mass index	Background diabetic retinopathy	ukb-a-248	finn-b-DM_BCKGRND_RETINA	rs588660	0.467	0.119	0.000
Body mass index	Background diabetic retinopathy	ukb-a-248	finn-b-DM_BCKGRND_RETINA	rs59104534	0.468	0.119	0.000
Body mass index	Background diabetic retinopathy	ukb-a-248	finn-b-DM_BCKGRND_RETINA	rs5995843	0.468	0.119	0.000
Body mass index	Background diabetic retinopathy	ukb-a-248	finn-b-DM_BCKGRND_RETINA	rs6050446	0.466	0.119	0.000
Body mass index	Background diabetic retinopathy	ukb-a-248	finn-b-DM_BCKGRND_RETINA	rs60654199	0.469	0.119	0.000
Body mass index	Background diabetic retinopathy	ukb-a-248	finn-b-DM_BCKGRND_RETINA	rs60764613	0.460	0.119	0.000
Body mass index	Background diabetic retinopathy	ukb-a-248	finn-b-DM_BCKGRND_RETINA	rs61813324	0.474	0.119	0.000

Body mass index	Background diabetic retinopathy	ukb-a-248	finn-b-DM_BCKGRND_RETINA	rs61826867	0.460	0.119	0.000
Body mass index	Background diabetic retinopathy	ukb-a-248	finn-b-DM_BCKGRND_RETINA	rs61871615	0.458	0.119	0.000
Body mass index	Background diabetic retinopathy	ukb-a-248	finn-b-DM_BCKGRND_RETINA	rs61903695	0.465	0.119	0.000
Body mass index	Background diabetic retinopathy	ukb-a-248	finn-b-DM_BCKGRND_RETINA	rs61969510	0.464	0.119	0.000
Body mass index	Background diabetic retinopathy	ukb-a-248	finn-b-DM_BCKGRND_RETINA	rs62106258	0.475	0.119	0.000
Body mass index	Background diabetic retinopathy	ukb-a-248	finn-b-DM_BCKGRND_RETINA	rs62147189	0.471	0.119	0.000
Body mass index	Background diabetic retinopathy	ukb-a-248	finn-b-DM_BCKGRND_RETINA	rs62246314	0.464	0.119	0.000
Body mass index	Background diabetic retinopathy	ukb-a-248	finn-b-DM_BCKGRND_RETINA	rs62407562	0.461	0.119	0.000

Body mass index	Background diabetic retinopathy	ukb-a-248	finn-b-DM_BCKGRND_RETINA	rs62543438	0.461	0.119	0.000
Body mass index	Background diabetic retinopathy	ukb-a-248	finn-b-DM_BCKGRND_RETINA	rs6265	0.490	0.118	0.000
Body mass index	Background diabetic retinopathy	ukb-a-248	finn-b-DM_BCKGRND_RETINA	rs6536575	0.468	0.119	0.000
Body mass index	Background diabetic retinopathy	ukb-a-248	finn-b-DM_BCKGRND_RETINA	rs6575340	0.472	0.119	0.000
Body mass index	Background diabetic retinopathy	ukb-a-248	finn-b-DM_BCKGRND_RETINA	rs6601527	0.461	0.119	0.000
Body mass index	Background diabetic retinopathy	ukb-a-248	finn-b-DM_BCKGRND_RETINA	rs66679256	0.469	0.119	0.000
Body mass index	Background diabetic retinopathy	ukb-a-248	finn-b-DM_BCKGRND_RETINA	rs6687953	0.471	0.119	0.000
Body mass index	Background diabetic retinopathy	ukb-a-248	finn-b-DM_BCKGRND_RETINA	rs66922415	0.454	0.120	0.000

Body mass index	Background diabetic retinopathy	ukb-a-248	finn-b-DM_BCKGRND_RETINA	rs6705567	0.458	0.118	0.000
Body mass index	Background diabetic retinopathy	ukb-a-248	finn-b-DM_BCKGRND_RETINA	rs6722241	0.469	0.119	0.000
Body mass index	Background diabetic retinopathy	ukb-a-248	finn-b-DM_BCKGRND_RETINA	rs6739755	0.449	0.118	0.000
Body mass index	Background diabetic retinopathy	ukb-a-248	finn-b-DM_BCKGRND_RETINA	rs67609008	0.468	0.119	0.000
Body mass index	Background diabetic retinopathy	ukb-a-248	finn-b-DM_BCKGRND_RETINA	rs6780459	0.466	0.119	0.000
Body mass index	Background diabetic retinopathy	ukb-a-248	finn-b-DM_BCKGRND_RETINA	rs67844506	0.471	0.119	0.000
Body mass index	Background diabetic retinopathy	ukb-a-248	finn-b-DM_BCKGRND_RETINA	rs6789488	0.466	0.119	0.000
Body mass index	Background diabetic retinopathy	ukb-a-248	finn-b-DM_BCKGRND_RETINA	rs6809307	0.471	0.118	0.000

Body mass index	Background diabetic retinopathy	ukb-a-248	finn-b-DM_BCKGRND_RETINA	rs6831020	0.471	0.119	0.000
Body mass index	Background diabetic retinopathy	ukb-a-248	finn-b-DM_BCKGRND_RETINA	rs6861649	0.461	0.119	0.000
Body mass index	Background diabetic retinopathy	ukb-a-248	finn-b-DM_BCKGRND_RETINA	rs6950388	0.463	0.119	0.000
Body mass index	Background diabetic retinopathy	ukb-a-248	finn-b-DM_BCKGRND_RETINA	rs7006178	0.460	0.119	0.000
Body mass index	Background diabetic retinopathy	ukb-a-248	finn-b-DM_BCKGRND_RETINA	rs7030732	0.458	0.119	0.000
Body mass index	Background diabetic retinopathy	ukb-a-248	finn-b-DM_BCKGRND_RETINA	rs704061	0.468	0.119	0.000
Body mass index	Background diabetic retinopathy	ukb-a-248	finn-b-DM_BCKGRND_RETINA	rs7094644	0.464	0.119	0.000
Body mass index	Background diabetic retinopathy	ukb-a-248	finn-b-DM_BCKGRND_RETINA	rs7116641	0.454	0.119	0.000

Body mass index	Background diabetic retinopathy	ukb-a-248	finn-b-DM_BCKGRND_RETINA	rs7124681	0.452	0.119	0.000
Body mass index	Background diabetic retinopathy	ukb-a-248	finn-b-DM_BCKGRND_RETINA	rs7132908	0.461	0.119	0.000
Body mass index	Background diabetic retinopathy	ukb-a-248	finn-b-DM_BCKGRND_RETINA	rs7138383	0.460	0.119	0.000
Body mass index	Background diabetic retinopathy	ukb-a-248	finn-b-DM_BCKGRND_RETINA	rs7141420	0.470	0.119	0.000
Body mass index	Background diabetic retinopathy	ukb-a-248	finn-b-DM_BCKGRND_RETINA	rs71495049	0.466	0.119	0.000
Body mass index	Background diabetic retinopathy	ukb-a-248	finn-b-DM_BCKGRND_RETINA	rs7183417	0.458	0.118	0.000
Body mass index	Background diabetic retinopathy	ukb-a-248	finn-b-DM_BCKGRND_RETINA	rs7189149	0.475	0.118	0.000
Body mass index	Background diabetic retinopathy	ukb-a-248	finn-b-DM_BCKGRND_RETINA	rs7195386	0.469	0.119	0.000

Body mass index	Background diabetic retinopathy	ukb-a-248	finn-b-DM_BCKGRND_RETINA	rs7201895	0.466	0.119	0.000
Body mass index	Background diabetic retinopathy	ukb-a-248	finn-b-DM_BCKGRND_RETINA	rs7218014	0.457	0.119	0.000
Body mass index	Background diabetic retinopathy	ukb-a-248	finn-b-DM_BCKGRND_RETINA	rs72697614	0.466	0.119	0.000
Body mass index	Background diabetic retinopathy	ukb-a-248	finn-b-DM_BCKGRND_RETINA	rs72820274	0.465	0.119	0.000
Body mass index	Background diabetic retinopathy	ukb-a-248	finn-b-DM_BCKGRND_RETINA	rs72892910	0.472	0.119	0.000
Body mass index	Background diabetic retinopathy	ukb-a-248	finn-b-DM_BCKGRND_RETINA	rs72976986	0.461	0.119	0.000
Body mass index	Background diabetic retinopathy	ukb-a-248	finn-b-DM_BCKGRND_RETINA	rs73050254	0.467	0.119	0.000
Body mass index	Background diabetic retinopathy	ukb-a-248	finn-b-DM_BCKGRND_RETINA	rs73144053	0.461	0.119	0.000

Body mass index	Background diabetic retinopathy	ukb-a-248	finn-b-DM_BCKGRND_RETINA	rs73169730	0.463	0.119	0.000
Body mass index	Background diabetic retinopathy	ukb-a-248	finn-b-DM_BCKGRND_RETINA	rs7321331	0.460	0.119	0.000
Body mass index	Background diabetic retinopathy	ukb-a-248	finn-b-DM_BCKGRND_RETINA	rs73213484	0.470	0.119	0.000
Body mass index	Background diabetic retinopathy	ukb-a-248	finn-b-DM_BCKGRND_RETINA	rs7331420	0.465	0.119	0.000
Body mass index	Background diabetic retinopathy	ukb-a-248	finn-b-DM_BCKGRND_RETINA	rs7442885	0.479	0.118	0.000
Body mass index	Background diabetic retinopathy	ukb-a-248	finn-b-DM_BCKGRND_RETINA	rs7498044	0.466	0.119	0.000
Body mass index	Background diabetic retinopathy	ukb-a-248	finn-b-DM_BCKGRND_RETINA	rs750090	0.464	0.119	0.000
Body mass index	Background diabetic retinopathy	ukb-a-248	finn-b-DM_BCKGRND_RETINA	rs752179	0.468	0.119	0.000

Body mass index	Background diabetic retinopathy	ukb-a-248	finn-b-DM_BCKGRND_RETINA	rs75499503	0.471	0.119	0.000
Body mass index	Background diabetic retinopathy	ukb-a-248	finn-b-DM_BCKGRND_RETINA	rs7553158	0.461	0.119	0.000
Body mass index	Background diabetic retinopathy	ukb-a-248	finn-b-DM_BCKGRND_RETINA	rs75557510	0.470	0.119	0.000
Body mass index	Background diabetic retinopathy	ukb-a-248	finn-b-DM_BCKGRND_RETINA	rs756717	0.479	0.117	0.000
Body mass index	Background diabetic retinopathy	ukb-a-248	finn-b-DM_BCKGRND_RETINA	rs76040172	0.455	0.119	0.000
Body mass index	Background diabetic retinopathy	ukb-a-248	finn-b-DM_BCKGRND_RETINA	rs7701777	0.475	0.118	0.000
Body mass index	Background diabetic retinopathy	ukb-a-248	finn-b-DM_BCKGRND_RETINA	rs7719067	0.463	0.119	0.000
Body mass index	Background diabetic retinopathy	ukb-a-248	finn-b-DM_BCKGRND_RETINA	rs7723426	0.461	0.119	0.000

Body mass index	Background diabetic retinopathy	ukb-a-248	finn-b-DM_BCKGRND_RETINA	rs7755574	0.459	0.119	0.000
Body mass index	Background diabetic retinopathy	ukb-a-248	finn-b-DM_BCKGRND_RETINA	rs7774	0.466	0.119	0.000
Body mass index	Background diabetic retinopathy	ukb-a-248	finn-b-DM_BCKGRND_RETINA	rs778094	0.461	0.119	0.000
Body mass index	Background diabetic retinopathy	ukb-a-248	finn-b-DM_BCKGRND_RETINA	rs7852189	0.461	0.119	0.000
Body mass index	Background diabetic retinopathy	ukb-a-248	finn-b-DM_BCKGRND_RETINA	rs78565420	0.461	0.119	0.000
Body mass index	Background diabetic retinopathy	ukb-a-248	finn-b-DM_BCKGRND_RETINA	rs79113395	0.463	0.119	0.000
Body mass index	Background diabetic retinopathy	ukb-a-248	finn-b-DM_BCKGRND_RETINA	rs7933085	0.465	0.119	0.000
Body mass index	Background diabetic retinopathy	ukb-a-248	finn-b-DM_BCKGRND_RETINA	rs7941828	0.462	0.119	0.000

Body mass index	Background diabetic retinopathy	ukb-a-248	finn-b-DM_BCKGRND_RETINA	rs7952102	0.466	0.119	0.000
Body mass index	Background diabetic retinopathy	ukb-a-248	finn-b-DM_BCKGRND_RETINA	rs7992832	0.457	0.118	0.000
Body mass index	Background diabetic retinopathy	ukb-a-248	finn-b-DM_BCKGRND_RETINA	rs799449	0.468	0.119	0.000
Body mass index	Background diabetic retinopathy	ukb-a-248	finn-b-DM_BCKGRND_RETINA	rs8015400	0.466	0.119	0.000
Body mass index	Background diabetic retinopathy	ukb-a-248	finn-b-DM_BCKGRND_RETINA	rs80330591	0.463	0.119	0.000
Body mass index	Background diabetic retinopathy	ukb-a-248	finn-b-DM_BCKGRND_RETINA	rs8078135	0.470	0.119	0.000
Body mass index	Background diabetic retinopathy	ukb-a-248	finn-b-DM_BCKGRND_RETINA	rs8134638	0.472	0.119	0.000
Body mass index	Background diabetic retinopathy	ukb-a-248	finn-b-DM_BCKGRND_RETINA	rs815163	0.468	0.119	0.000

Body mass index	Background diabetic retinopathy	ukb-a-248	finn-b-DM_BCKGRND_RETINA	rs845084	0.450	0.118	0.000
Body mass index	Background diabetic retinopathy	ukb-a-248	finn-b-DM_BCKGRND_RETINA	rs862320	0.467	0.119	0.000
Body mass index	Background diabetic retinopathy	ukb-a-248	finn-b-DM_BCKGRND_RETINA	rs869400	0.470	0.119	0.000
Body mass index	Background diabetic retinopathy	ukb-a-248	finn-b-DM_BCKGRND_RETINA	rs879620	0.464	0.119	0.000
Body mass index	Background diabetic retinopathy	ukb-a-248	finn-b-DM_BCKGRND_RETINA	rs9267671	0.476	0.117	0.000
Body mass index	Background diabetic retinopathy	ukb-a-248	finn-b-DM_BCKGRND_RETINA	rs9291822	0.465	0.119	0.000
Body mass index	Background diabetic retinopathy	ukb-a-248	finn-b-DM_BCKGRND_RETINA	rs9320823	0.462	0.119	0.000
Body mass index	Background diabetic retinopathy	ukb-a-248	finn-b-DM_BCKGRND_RETINA	rs9342196	0.464	0.119	0.000

Body mass index	Background diabetic retinopathy	ukb-a-248	finn-b-DM_BCKGRND_RETINA	rs935166	0.470	0.119	0.000
Body mass index	Background diabetic retinopathy	ukb-a-248	finn-b-DM_BCKGRND_RETINA	rs9402104	0.464	0.119	0.000
Body mass index	Background diabetic retinopathy	ukb-a-248	finn-b-DM_BCKGRND_RETINA	rs946185	0.462	0.119	0.000
Body mass index	Background diabetic retinopathy	ukb-a-248	finn-b-DM_BCKGRND_RETINA	rs9515455	0.469	0.119	0.000
Body mass index	Background diabetic retinopathy	ukb-a-248	finn-b-DM_BCKGRND_RETINA	rs9527906	0.469	0.119	0.000
Body mass index	Background diabetic retinopathy	ukb-a-248	finn-b-DM_BCKGRND_RETINA	rs9641499	0.467	0.119	0.000
Body mass index	Background diabetic retinopathy	ukb-a-248	finn-b-DM_BCKGRND_RETINA	rs9688977	0.457	0.119	0.000
Body mass index	Background diabetic retinopathy	ukb-a-248	finn-b-DM_BCKGRND_RETINA	rs9843653	0.462	0.119	0.000

Body mass index	Background diabetic retinopathy	ukb-a-248	finn-b-DM_BCKGRND_RETINA	rs9847186	0.469	0.119	0.000
Body mass index	Background diabetic retinopathy	ukb-a-248	finn-b-DM_BCKGRND_RETINA	All	0.465	0.118	0.000
Body mass index	Diabetic retinopathy	ukb-a-248	finn-b-DM_RETINOPATHY_EXMORE	rs10100245	0.344	0.048	0.000
Body mass index	Diabetic retinopathy	ukb-a-248	finn-b-DM_RETINOPATHY_EXMORE	rs10187101	0.348	0.048	0.000
Body mass index	Diabetic retinopathy	ukb-a-248	finn-b-DM_RETINOPATHY_EXMORE	rs10404726	0.350	0.048	0.000
Body mass index	Diabetic retinopathy	ukb-a-248	finn-b-DM_RETINOPATHY_EXMORE	rs10465231	0.348	0.048	0.000
Body mass index	Diabetic retinopathy	ukb-a-248	finn-b-DM_RETINOPATHY_EXMORE	rs1064213	0.349	0.048	0.000
Body mass index	Diabetic retinopathy	ukb-a-248	finn-b-DM_RETINOPATHY_EXMORE	rs10788493	0.347	0.048	0.000

Body mass index	Diabetic retinopathy	ukb-a-248	finn-b-DM_RETINOPATHY_EXMORE	rs10803762	0.351	0.048	0.000
Body mass index	Diabetic retinopathy	ukb-a-248	finn-b-DM_RETINOPATHY_EXMORE	rs10805383	0.348	0.048	0.000
Body mass index	Diabetic retinopathy	ukb-a-248	finn-b-DM_RETINOPATHY_EXMORE	rs10865612	0.353	0.048	0.000
Body mass index	Diabetic retinopathy	ukb-a-248	finn-b-DM_RETINOPATHY_EXMORE	rs10898330	0.351	0.048	0.000
Body mass index	Diabetic retinopathy	ukb-a-248	finn-b-DM_RETINOPATHY_EXMORE	rs10938397	0.334	0.048	0.000
Body mass index	Diabetic retinopathy	ukb-a-248	finn-b-DM_RETINOPATHY_EXMORE	rs10995427	0.349	0.048	0.000
Body mass index	Diabetic retinopathy	ukb-a-248	finn-b-DM_RETINOPATHY_EXMORE	rs11012732	0.344	0.048	0.000
Body mass index	Diabetic retinopathy	ukb-a-248	finn-b-DM_RETINOPATHY_EXMORE	rs11078883	0.345	0.048	0.000

Body mass index	Diabetic retinopathy	ukb-a-248	finn-b-DM_RETINOPATHY_EXMORE	rs11084554	0.345	0.048	0.000
Body mass index	Diabetic retinopathy	ukb-a-248	finn-b-DM_RETINOPATHY_EXMORE	rs11099020	0.348	0.048	0.000
Body mass index	Diabetic retinopathy	ukb-a-248	finn-b-DM_RETINOPATHY_EXMORE	rs11150745	0.350	0.048	0.000
Body mass index	Diabetic retinopathy	ukb-a-248	finn-b-DM_RETINOPATHY_EXMORE	rs111640872	0.352	0.048	0.000
Body mass index	Diabetic retinopathy	ukb-a-248	finn-b-DM_RETINOPATHY_EXMORE	rs11223641	0.347	0.048	0.000
Body mass index	Diabetic retinopathy	ukb-a-248	finn-b-DM_RETINOPATHY_EXMORE	rs112520079	0.351	0.048	0.000
Body mass index	Diabetic retinopathy	ukb-a-248	finn-b-DM_RETINOPATHY_EXMORE	rs11264489	0.348	0.048	0.000
Body mass index	Diabetic retinopathy	ukb-a-248	finn-b-DM_RETINOPATHY_EXMORE	rs112693590	0.349	0.048	0.000

Body mass index	Diabetic retinopathy	ukb-a-248	finn-b-DM_RETINOPATHY_EXMORE	rs1127100	0.350	0.048	0.000
Body mass index	Diabetic retinopathy	ukb-a-248	finn-b-DM_RETINOPATHY_EXMORE	rs113182412	0.351	0.048	0.000
Body mass index	Diabetic retinopathy	ukb-a-248	finn-b-DM_RETINOPATHY_EXMORE	rs113230003	0.350	0.048	0.000
Body mass index	Diabetic retinopathy	ukb-a-248	finn-b-DM_RETINOPATHY_EXMORE	rs113603865	0.345	0.048	0.000
Body mass index	Diabetic retinopathy	ukb-a-248	finn-b-DM_RETINOPATHY_EXMORE	rs11515071	0.352	0.048	0.000
Body mass index	Diabetic retinopathy	ukb-a-248	finn-b-DM_RETINOPATHY_EXMORE	rs11642015	0.325	0.049	0.000
Body mass index	Diabetic retinopathy	ukb-a-248	finn-b-DM_RETINOPATHY_EXMORE	rs11650012	0.349	0.048	0.000
Body mass index	Diabetic retinopathy	ukb-a-248	finn-b-DM_RETINOPATHY_EXMORE	rs11655587	0.348	0.048	0.000

Body mass index	Diabetic retinopathy	ukb-a-248	finn-b-DM_RETINOPATHY_EXMORE	rs11742930	0.350	0.048	0.000
Body mass index	Diabetic retinopathy	ukb-a-248	finn-b-DM_RETINOPATHY_EXMORE	rs11757278	0.350	0.048	0.000
Body mass index	Diabetic retinopathy	ukb-a-248	finn-b-DM_RETINOPATHY_EXMORE	rs11761411	0.348	0.048	0.000
Body mass index	Diabetic retinopathy	ukb-a-248	finn-b-DM_RETINOPATHY_EXMORE	rs117632017	0.351	0.048	0.000
Body mass index	Diabetic retinopathy	ukb-a-248	finn-b-DM_RETINOPATHY_EXMORE	rs11782074	0.348	0.048	0.000
Body mass index	Diabetic retinopathy	ukb-a-248	finn-b-DM_RETINOPATHY_EXMORE	rs11856579	0.351	0.048	0.000
Body mass index	Diabetic retinopathy	ukb-a-248	finn-b-DM_RETINOPATHY_EXMORE	rs12024554	0.347	0.048	0.000
Body mass index	Diabetic retinopathy	ukb-a-248	finn-b-DM_RETINOPATHY_EXMORE	rs12042959	0.348	0.048	0.000

Body mass index	Diabetic retinopathy	ukb-a-248	finn-b-DM_RETINOPATHY_EXMORE	rs12049202	0.344	0.048	0.000
Body mass index	Diabetic retinopathy	ukb-a-248	finn-b-DM_RETINOPATHY_EXMORE	rs12140153	0.343	0.048	0.000
Body mass index	Diabetic retinopathy	ukb-a-248	finn-b-DM_RETINOPATHY_EXMORE	rs12144626	0.347	0.048	0.000
Body mass index	Diabetic retinopathy	ukb-a-248	finn-b-DM_RETINOPATHY_EXMORE	rs12477385	0.348	0.048	0.000
Body mass index	Diabetic retinopathy	ukb-a-248	finn-b-DM_RETINOPATHY_EXMORE	rs12479357	0.349	0.048	0.000
Body mass index	Diabetic retinopathy	ukb-a-248	finn-b-DM_RETINOPATHY_EXMORE	rs12614861	0.352	0.048	0.000
Body mass index	Diabetic retinopathy	ukb-a-248	finn-b-DM_RETINOPATHY_EXMORE	rs12622280	0.346	0.048	0.000
Body mass index	Diabetic retinopathy	ukb-a-248	finn-b-DM_RETINOPATHY_EXMORE	rs12662900	0.347	0.048	0.000

Body mass index	Diabetic retinopathy	ukb-a-248	finn-b-DM_RETINOPATHY_EXMORE	rs12679106	0.349	0.048	0.000
Body mass index	Diabetic retinopathy	ukb-a-248	finn-b-DM_RETINOPATHY_EXMORE	rs1286138	0.350	0.048	0.000
Body mass index	Diabetic retinopathy	ukb-a-248	finn-b-DM_RETINOPATHY_EXMORE	rs12877270	0.349	0.048	0.000
Body mass index	Diabetic retinopathy	ukb-a-248	finn-b-DM_RETINOPATHY_EXMORE	rs12881629	0.349	0.048	0.000
Body mass index	Diabetic retinopathy	ukb-a-248	finn-b-DM_RETINOPATHY_EXMORE	rs12885458	0.344	0.048	0.000
Body mass index	Diabetic retinopathy	ukb-a-248	finn-b-DM_RETINOPATHY_EXMORE	rs1296328	0.354	0.048	0.000
Body mass index	Diabetic retinopathy	ukb-a-248	finn-b-DM_RETINOPATHY_EXMORE	rs12977259	0.352	0.048	0.000
Body mass index	Diabetic retinopathy	ukb-a-248	finn-b-DM_RETINOPATHY_EXMORE	rs12992672	0.349	0.049	0.000

Body mass index	Diabetic retinopathy	ukb-a-248	finn-b-DM_RETINOPATHY_EXMORE	rs13062093	0.347	0.048	0.000
Body mass index	Diabetic retinopathy	ukb-a-248	finn-b-DM_RETINOPATHY_EXMORE	rs13076052	0.348	0.048	0.000
Body mass index	Diabetic retinopathy	ukb-a-248	finn-b-DM_RETINOPATHY_EXMORE	rs13135092	0.347	0.048	0.000
Body mass index	Diabetic retinopathy	ukb-a-248	finn-b-DM_RETINOPATHY_EXMORE	rs13174863	0.350	0.048	0.000
Body mass index	Diabetic retinopathy	ukb-a-248	finn-b-DM_RETINOPATHY_EXMORE	rs1320903	0.345	0.048	0.000
Body mass index	Diabetic retinopathy	ukb-a-248	finn-b-DM_RETINOPATHY_EXMORE	rs1327259	0.347	0.048	0.000
Body mass index	Diabetic retinopathy	ukb-a-248	finn-b-DM_RETINOPATHY_EXMORE	rs1342391	0.348	0.048	0.000
Body mass index	Diabetic retinopathy	ukb-a-248	finn-b-DM_RETINOPATHY_EXMORE	rs13427822	0.348	0.048	0.000

Body mass index	Diabetic retinopathy	ukb-a-248	finn-b-DM_RETINOPATHY_EXMORE	rs1411432	0.345	0.048	0.000
Body mass index	Diabetic retinopathy	ukb-a-248	finn-b-DM_RETINOPATHY_EXMORE	rs1412239	0.348	0.048	0.000
Body mass index	Diabetic retinopathy	ukb-a-248	finn-b-DM_RETINOPATHY_EXMORE	rs1441264	0.353	0.048	0.000
Body mass index	Diabetic retinopathy	ukb-a-248	finn-b-DM_RETINOPATHY_EXMORE	rs1446585	0.349	0.048	0.000
Body mass index	Diabetic retinopathy	ukb-a-248	finn-b-DM_RETINOPATHY_EXMORE	rs1458156	0.347	0.048	0.000
Body mass index	Diabetic retinopathy	ukb-a-248	finn-b-DM_RETINOPATHY_EXMORE	rs1477290	0.353	0.048	0.000
Body mass index	Diabetic retinopathy	ukb-a-248	finn-b-DM_RETINOPATHY_EXMORE	rs147730268	0.351	0.048	0.000
Body mass index	Diabetic retinopathy	ukb-a-248	finn-b-DM_RETINOPATHY_EXMORE	rs1582931	0.349	0.048	0.000

Body mass index	Diabetic retinopathy	ukb-a-248	finn-b-DM_RETINOPATHY_EXMORE	rs16846140	0.353	0.048	0.000
Body mass index	Diabetic retinopathy	ukb-a-248	finn-b-DM_RETINOPATHY_EXMORE	rs16916303	0.348	0.048	0.000
Body mass index	Diabetic retinopathy	ukb-a-248	finn-b-DM_RETINOPATHY_EXMORE	rs16975459	0.352	0.048	0.000
Body mass index	Diabetic retinopathy	ukb-a-248	finn-b-DM_RETINOPATHY_EXMORE	rs17014332	0.349	0.048	0.000
Body mass index	Diabetic retinopathy	ukb-a-248	finn-b-DM_RETINOPATHY_EXMORE	rs17024393	0.353	0.048	0.000
Body mass index	Diabetic retinopathy	ukb-a-248	finn-b-DM_RETINOPATHY_EXMORE	rs17058884	0.346	0.048	0.000
Body mass index	Diabetic retinopathy	ukb-a-248	finn-b-DM_RETINOPATHY_EXMORE	rs17085463	0.352	0.048	0.000
Body mass index	Diabetic retinopathy	ukb-a-248	finn-b-DM_RETINOPATHY_EXMORE	rs17149254	0.348	0.048	0.000

Body mass index	Diabetic retinopathy	ukb-a-248	finn-b-DM_RETINOPATHY_EXMORE	rs17342242	0.351	0.048	0.000
Body mass index	Diabetic retinopathy	ukb-a-248	finn-b-DM_RETINOPATHY_EXMORE	rs17399739	0.347	0.048	0.000
Body mass index	Diabetic retinopathy	ukb-a-248	finn-b-DM_RETINOPATHY_EXMORE	rs17716502	0.350	0.048	0.000
Body mass index	Diabetic retinopathy	ukb-a-248	finn-b-DM_RETINOPATHY_EXMORE	rs1788808	0.350	0.048	0.000
Body mass index	Diabetic retinopathy	ukb-a-248	finn-b-DM_RETINOPATHY_EXMORE	rs1805123	0.347	0.048	0.000
Body mass index	Diabetic retinopathy	ukb-a-248	finn-b-DM_RETINOPATHY_EXMORE	rs1884897	0.355	0.048	0.000
Body mass index	Diabetic retinopathy	ukb-a-248	finn-b-DM_RETINOPATHY_EXMORE	rs1901241	0.349	0.048	0.000
Body mass index	Diabetic retinopathy	ukb-a-248	finn-b-DM_RETINOPATHY_EXMORE	rs1919243	0.347	0.048	0.000

Body mass index	Diabetic retinopathy	ukb-a-248	finn-b-DM_RETINOPATHY_EXMORE	rs1941706	0.349	0.048	0.000
Body mass index	Diabetic retinopathy	ukb-a-248	finn-b-DM_RETINOPATHY_EXMORE	rs1949204	0.350	0.048	0.000
Body mass index	Diabetic retinopathy	ukb-a-248	finn-b-DM_RETINOPATHY_EXMORE	rs2035806	0.353	0.048	0.000
Body mass index	Diabetic retinopathy	ukb-a-248	finn-b-DM_RETINOPATHY_EXMORE	rs2046002	0.347	0.048	0.000
Body mass index	Diabetic retinopathy	ukb-a-248	finn-b-DM_RETINOPATHY_EXMORE	rs2121058	0.345	0.048	0.000
Body mass index	Diabetic retinopathy	ukb-a-248	finn-b-DM_RETINOPATHY_EXMORE	rs2135745	0.350	0.048	0.000
Body mass index	Diabetic retinopathy	ukb-a-248	finn-b-DM_RETINOPATHY_EXMORE	rs2155869	0.352	0.048	0.000
Body mass index	Diabetic retinopathy	ukb-a-248	finn-b-DM_RETINOPATHY_EXMORE	rs215634	0.344	0.048	0.000

Body mass index	Diabetic retinopathy	ukb-a-248	finn-b-DM_RETINOPATHY_EXMORE	rs2192649	0.351	0.048	0.000
Body mass index	Diabetic retinopathy	ukb-a-248	finn-b-DM_RETINOPATHY_EXMORE	rs2234458	0.350	0.048	0.000
Body mass index	Diabetic retinopathy	ukb-a-248	finn-b-DM_RETINOPATHY_EXMORE	rs2292238	0.354	0.048	0.000
Body mass index	Diabetic retinopathy	ukb-a-248	finn-b-DM_RETINOPATHY_EXMORE	rs2307111	0.342	0.048	0.000
Body mass index	Diabetic retinopathy	ukb-a-248	finn-b-DM_RETINOPATHY_EXMORE	rs2318543	0.345	0.048	0.000
Body mass index	Diabetic retinopathy	ukb-a-248	finn-b-DM_RETINOPATHY_EXMORE	rs2384054	0.345	0.048	0.000
Body mass index	Diabetic retinopathy	ukb-a-248	finn-b-DM_RETINOPATHY_EXMORE	rs2398861	0.350	0.048	0.000
Body mass index	Diabetic retinopathy	ukb-a-248	finn-b-DM_RETINOPATHY_EXMORE	rs241460	0.350	0.048	0.000

Body mass index	Diabetic retinopathy	ukb-a-248	finn-b-DM_RETINOPATHY_EXMORE	rs2425857	0.346	0.048	0.000
Body mass index	Diabetic retinopathy	ukb-a-248	finn-b-DM_RETINOPATHY_EXMORE	rs2439823	0.348	0.048	0.000
Body mass index	Diabetic retinopathy	ukb-a-248	finn-b-DM_RETINOPATHY_EXMORE	rs2450254	0.348	0.048	0.000
Body mass index	Diabetic retinopathy	ukb-a-248	finn-b-DM_RETINOPATHY_EXMORE	rs2450445	0.350	0.048	0.000
Body mass index	Diabetic retinopathy	ukb-a-248	finn-b-DM_RETINOPATHY_EXMORE	rs245775	0.350	0.048	0.000
Body mass index	Diabetic retinopathy	ukb-a-248	finn-b-DM_RETINOPATHY_EXMORE	rs2470392	0.347	0.048	0.000
Body mass index	Diabetic retinopathy	ukb-a-248	finn-b-DM_RETINOPATHY_EXMORE	rs2474898	0.348	0.048	0.000
Body mass index	Diabetic retinopathy	ukb-a-248	finn-b-DM_RETINOPATHY_EXMORE	rs2482704	0.349	0.048	0.000

Body mass index	Diabetic retinopathy	ukb-a-248	finn-b-DM_RETINOPATHY_EXMORE	rs2606228	0.346	0.048	0.000
Body mass index	Diabetic retinopathy	ukb-a-248	finn-b-DM_RETINOPATHY_EXMORE	rs2616192	0.346	0.048	0.000
Body mass index	Diabetic retinopathy	ukb-a-248	finn-b-DM_RETINOPATHY_EXMORE	rs2678204	0.349	0.048	0.000
Body mass index	Diabetic retinopathy	ukb-a-248	finn-b-DM_RETINOPATHY_EXMORE	rs2711111	0.351	0.048	0.000
Body mass index	Diabetic retinopathy	ukb-a-248	finn-b-DM_RETINOPATHY_EXMORE	rs2725371	0.346	0.048	0.000
Body mass index	Diabetic retinopathy	ukb-a-248	finn-b-DM_RETINOPATHY_EXMORE	rs273505	0.351	0.048	0.000
Body mass index	Diabetic retinopathy	ukb-a-248	finn-b-DM_RETINOPATHY_EXMORE	rs28366156	0.337	0.046	0.000
Body mass index	Diabetic retinopathy	ukb-a-248	finn-b-DM_RETINOPATHY_EXMORE	rs28447555	0.347	0.048	0.000

Body mass index	Diabetic retinopathy	ukb-a-248	finn-b-DM_RETINOPATHY_EXMORE	rs28489620	0.351	0.048	0.000
Body mass index	Diabetic retinopathy	ukb-a-248	finn-b-DM_RETINOPATHY_EXMORE	rs2861685	0.349	0.048	0.000
Body mass index	Diabetic retinopathy	ukb-a-248	finn-b-DM_RETINOPATHY_EXMORE	rs286818	0.356	0.048	0.000
Body mass index	Diabetic retinopathy	ukb-a-248	finn-b-DM_RETINOPATHY_EXMORE	rs2962082	0.347	0.048	0.000
Body mass index	Diabetic retinopathy	ukb-a-248	finn-b-DM_RETINOPATHY_EXMORE	rs2975693	0.349	0.048	0.000
Body mass index	Diabetic retinopathy	ukb-a-248	finn-b-DM_RETINOPATHY_EXMORE	rs34045288	0.347	0.048	0.000
Body mass index	Diabetic retinopathy	ukb-a-248	finn-b-DM_RETINOPATHY_EXMORE	rs34095326	0.346	0.048	0.000
Body mass index	Diabetic retinopathy	ukb-a-248	finn-b-DM_RETINOPATHY_EXMORE	rs34236292	0.348	0.048	0.000

Body mass index	Diabetic retinopathy	ukb-a-248	finn-b-DM_RETINOPATHY_EXMORE	rs34361149	0.350	0.048	0.000
Body mass index	Diabetic retinopathy	ukb-a-248	finn-b-DM_RETINOPATHY_EXMORE	rs34373881	0.346	0.048	0.000
Body mass index	Diabetic retinopathy	ukb-a-248	finn-b-DM_RETINOPATHY_EXMORE	rs34774377	0.349	0.048	0.000
Body mass index	Diabetic retinopathy	ukb-a-248	finn-b-DM_RETINOPATHY_EXMORE	rs34811474	0.345	0.048	0.000
Body mass index	Diabetic retinopathy	ukb-a-248	finn-b-DM_RETINOPATHY_EXMORE	rs34966008	0.346	0.048	0.000
Body mass index	Diabetic retinopathy	ukb-a-248	finn-b-DM_RETINOPATHY_EXMORE	rs35025195	0.345	0.048	0.000
Body mass index	Diabetic retinopathy	ukb-a-248	finn-b-DM_RETINOPATHY_EXMORE	rs35193668	0.345	0.048	0.000
Body mass index	Diabetic retinopathy	ukb-a-248	finn-b-DM_RETINOPATHY_EXMORE	rs35483388	0.348	0.048	0.000

Body mass index	Diabetic retinopathy	ukb-a-248	finn-b-DM_RETINOPATHY_EXMORE	rs35626515	0.359	0.048	0.000
Body mass index	Diabetic retinopathy	ukb-a-248	finn-b-DM_RETINOPATHY_EXMORE	rs35722922	0.349	0.048	0.000
Body mass index	Diabetic retinopathy	ukb-a-248	finn-b-DM_RETINOPATHY_EXMORE	rs357501	0.350	0.048	0.000
Body mass index	Diabetic retinopathy	ukb-a-248	finn-b-DM_RETINOPATHY_EXMORE	rs35851183	0.349	0.048	0.000
Body mass index	Diabetic retinopathy	ukb-a-248	finn-b-DM_RETINOPATHY_EXMORE	rs35882248	0.351	0.048	0.000
Body mass index	Diabetic retinopathy	ukb-a-248	finn-b-DM_RETINOPATHY_EXMORE	rs36007635	0.348	0.048	0.000
Body mass index	Diabetic retinopathy	ukb-a-248	finn-b-DM_RETINOPATHY_EXMORE	rs362307	0.348	0.048	0.000
Body mass index	Diabetic retinopathy	ukb-a-248	finn-b-DM_RETINOPATHY_EXMORE	rs3759584	0.345	0.048	0.000

Body mass index	Diabetic retinopathy	ukb-a-248	finn-b-DM_RETINOPATHY_EXMORE	rs3802858	0.347	0.048	0.000
Body mass index	Diabetic retinopathy	ukb-a-248	finn-b-DM_RETINOPATHY_EXMORE	rs3803286	0.346	0.048	0.000
Body mass index	Diabetic retinopathy	ukb-a-248	finn-b-DM_RETINOPATHY_EXMORE	rs3810291	0.342	0.048	0.000
Body mass index	Diabetic retinopathy	ukb-a-248	finn-b-DM_RETINOPATHY_EXMORE	rs3843540	0.360	0.047	0.000
Body mass index	Diabetic retinopathy	ukb-a-248	finn-b-DM_RETINOPATHY_EXMORE	rs3844598	0.347	0.048	0.000
Body mass index	Diabetic retinopathy	ukb-a-248	finn-b-DM_RETINOPATHY_EXMORE	rs3861879	0.349	0.048	0.000
Body mass index	Diabetic retinopathy	ukb-a-248	finn-b-DM_RETINOPATHY_EXMORE	rs3897102	0.350	0.048	0.000
Body mass index	Diabetic retinopathy	ukb-a-248	finn-b-DM_RETINOPATHY_EXMORE	rs390192	0.345	0.048	0.000

Body mass index	Diabetic retinopathy	ukb-a-248	finn-b-DM_RETINOPATHY_EXMORE	rs4246657	0.345	0.048	0.000
Body mass index	Diabetic retinopathy	ukb-a-248	finn-b-DM_RETINOPATHY_EXMORE	rs4261944	0.345	0.048	0.000
Body mass index	Diabetic retinopathy	ukb-a-248	finn-b-DM_RETINOPATHY_EXMORE	rs4402589	0.352	0.048	0.000
Body mass index	Diabetic retinopathy	ukb-a-248	finn-b-DM_RETINOPATHY_EXMORE	rs4467770	0.346	0.048	0.000
Body mass index	Diabetic retinopathy	ukb-a-248	finn-b-DM_RETINOPATHY_EXMORE	rs4474229	0.348	0.048	0.000
Body mass index	Diabetic retinopathy	ukb-a-248	finn-b-DM_RETINOPATHY_EXMORE	rs4482463	0.346	0.048	0.000
Body mass index	Diabetic retinopathy	ukb-a-248	finn-b-DM_RETINOPATHY_EXMORE	rs4502882	0.349	0.048	0.000
Body mass index	Diabetic retinopathy	ukb-a-248	finn-b-DM_RETINOPATHY_EXMORE	rs4595495	0.348	0.048	0.000

Body mass index	Diabetic retinopathy	ukb-a-248	finn-b-DM_RETINOPATHY_EXMORE	rs4648450	0.349	0.048	0.000
Body mass index	Diabetic retinopathy	ukb-a-248	finn-b-DM_RETINOPATHY_EXMORE	rs4687770	0.350	0.048	0.000
Body mass index	Diabetic retinopathy	ukb-a-248	finn-b-DM_RETINOPATHY_EXMORE	rs4718964	0.352	0.048	0.000
Body mass index	Diabetic retinopathy	ukb-a-248	finn-b-DM_RETINOPATHY_EXMORE	rs4757144	0.348	0.048	0.000
Body mass index	Diabetic retinopathy	ukb-a-248	finn-b-DM_RETINOPATHY_EXMORE	rs4776970	0.348	0.048	0.000
Body mass index	Diabetic retinopathy	ukb-a-248	finn-b-DM_RETINOPATHY_EXMORE	rs4777541	0.348	0.048	0.000
Body mass index	Diabetic retinopathy	ukb-a-248	finn-b-DM_RETINOPATHY_EXMORE	rs4911382	0.349	0.048	0.000
Body mass index	Diabetic retinopathy	ukb-a-248	finn-b-DM_RETINOPATHY_EXMORE	rs491711	0.349	0.048	0.000

Body mass index	Diabetic retinopathy	ukb-a-248	finn-b-DM_RETINOPATHY_EXMORE	rs4921301	0.350	0.048	0.000
Body mass index	Diabetic retinopathy	ukb-a-248	finn-b-DM_RETINOPATHY_EXMORE	rs4929923	0.347	0.048	0.000
Body mass index	Diabetic retinopathy	ukb-a-248	finn-b-DM_RETINOPATHY_EXMORE	rs525101	0.348	0.048	0.000
Body mass index	Diabetic retinopathy	ukb-a-248	finn-b-DM_RETINOPATHY_EXMORE	rs539515	0.353	0.048	0.000
Body mass index	Diabetic retinopathy	ukb-a-248	finn-b-DM_RETINOPATHY_EXMORE	rs550974	0.347	0.048	0.000
Body mass index	Diabetic retinopathy	ukb-a-248	finn-b-DM_RETINOPATHY_EXMORE	rs55689274	0.349	0.048	0.000
Body mass index	Diabetic retinopathy	ukb-a-248	finn-b-DM_RETINOPATHY_EXMORE	rs55726687	0.349	0.048	0.000
Body mass index	Diabetic retinopathy	ukb-a-248	finn-b-DM_RETINOPATHY_EXMORE	rs55886426	0.346	0.048	0.000

Body mass index	Diabetic retinopathy	ukb-a-248	finn-b-DM_RETINOPATHY_EXMORE	rs55938344	0.350	0.048	0.000
Body mass index	Diabetic retinopathy	ukb-a-248	finn-b-DM_RETINOPATHY_EXMORE	rs56067609	0.348	0.048	0.000
Body mass index	Diabetic retinopathy	ukb-a-248	finn-b-DM_RETINOPATHY_EXMORE	rs56161855	0.356	0.047	0.000
Body mass index	Diabetic retinopathy	ukb-a-248	finn-b-DM_RETINOPATHY_EXMORE	rs56212061	0.348	0.048	0.000
Body mass index	Diabetic retinopathy	ukb-a-248	finn-b-DM_RETINOPATHY_EXMORE	rs56773984	0.350	0.048	0.000
Body mass index	Diabetic retinopathy	ukb-a-248	finn-b-DM_RETINOPATHY_EXMORE	rs56803094	0.348	0.048	0.000
Body mass index	Diabetic retinopathy	ukb-a-248	finn-b-DM_RETINOPATHY_EXMORE	rs57636386	0.353	0.048	0.000
Body mass index	Diabetic retinopathy	ukb-a-248	finn-b-DM_RETINOPATHY_EXMORE	rs58862095	0.351	0.048	0.000

Body mass index	Diabetic retinopathy	ukb-a-248	finn-b-DM_RETINOPATHY_EXMORE	rs588660	0.349	0.048	0.000
Body mass index	Diabetic retinopathy	ukb-a-248	finn-b-DM_RETINOPATHY_EXMORE	rs59104534	0.349	0.048	0.000
Body mass index	Diabetic retinopathy	ukb-a-248	finn-b-DM_RETINOPATHY_EXMORE	rs5995843	0.350	0.048	0.000
Body mass index	Diabetic retinopathy	ukb-a-248	finn-b-DM_RETINOPATHY_EXMORE	rs6050446	0.350	0.048	0.000
Body mass index	Diabetic retinopathy	ukb-a-248	finn-b-DM_RETINOPATHY_EXMORE	rs60654199	0.350	0.048	0.000
Body mass index	Diabetic retinopathy	ukb-a-248	finn-b-DM_RETINOPATHY_EXMORE	rs60764613	0.345	0.048	0.000
Body mass index	Diabetic retinopathy	ukb-a-248	finn-b-DM_RETINOPATHY_EXMORE	rs61813324	0.351	0.048	0.000
Body mass index	Diabetic retinopathy	ukb-a-248	finn-b-DM_RETINOPATHY_EXMORE	rs61826867	0.347	0.048	0.000

Body mass index	Diabetic retinopathy	ukb-a-248	finn-b-DM_RETINOPATHY_EXMORE	rs61871615	0.348	0.048	0.000
Body mass index	Diabetic retinopathy	ukb-a-248	finn-b-DM_RETINOPATHY_EXMORE	rs61903695	0.346	0.048	0.000
Body mass index	Diabetic retinopathy	ukb-a-248	finn-b-DM_RETINOPATHY_EXMORE	rs61969510	0.346	0.048	0.000
Body mass index	Diabetic retinopathy	ukb-a-248	finn-b-DM_RETINOPATHY_EXMORE	rs62106258	0.349	0.048	0.000
Body mass index	Diabetic retinopathy	ukb-a-248	finn-b-DM_RETINOPATHY_EXMORE	rs62147189	0.350	0.048	0.000
Body mass index	Diabetic retinopathy	ukb-a-248	finn-b-DM_RETINOPATHY_EXMORE	rs62246314	0.349	0.048	0.000
Body mass index	Diabetic retinopathy	ukb-a-248	finn-b-DM_RETINOPATHY_EXMORE	rs62407562	0.349	0.048	0.000
Body mass index	Diabetic retinopathy	ukb-a-248	finn-b-DM_RETINOPATHY_EXMORE	rs62543438	0.347	0.048	0.000

Body mass index	Diabetic retinopathy	ukb-a-248	finn-b-DM_RETINOPATHY_EXMORE	rs6265	0.352	0.048	0.000
Body mass index	Diabetic retinopathy	ukb-a-248	finn-b-DM_RETINOPATHY_EXMORE	rs6536575	0.349	0.048	0.000
Body mass index	Diabetic retinopathy	ukb-a-248	finn-b-DM_RETINOPATHY_EXMORE	rs6575340	0.348	0.048	0.000
Body mass index	Diabetic retinopathy	ukb-a-248	finn-b-DM_RETINOPATHY_EXMORE	rs6601527	0.348	0.048	0.000
Body mass index	Diabetic retinopathy	ukb-a-248	finn-b-DM_RETINOPATHY_EXMORE	rs66679256	0.353	0.048	0.000
Body mass index	Diabetic retinopathy	ukb-a-248	finn-b-DM_RETINOPATHY_EXMORE	rs6687953	0.349	0.048	0.000
Body mass index	Diabetic retinopathy	ukb-a-248	finn-b-DM_RETINOPATHY_EXMORE	rs66922415	0.345	0.049	0.000
Body mass index	Diabetic retinopathy	ukb-a-248	finn-b-DM_RETINOPATHY_EXMORE	rs6705567	0.350	0.048	0.000

Body mass index	Diabetic retinopathy	ukb-a-248	finn-b-DM_RETINOPATHY_EXMORE	rs6722241	0.346	0.048	0.000
Body mass index	Diabetic retinopathy	ukb-a-248	finn-b-DM_RETINOPATHY_EXMORE	rs6739755	0.342	0.048	0.000
Body mass index	Diabetic retinopathy	ukb-a-248	finn-b-DM_RETINOPATHY_EXMORE	rs67609008	0.351	0.048	0.000
Body mass index	Diabetic retinopathy	ukb-a-248	finn-b-DM_RETINOPATHY_EXMORE	rs6780459	0.348	0.048	0.000
Body mass index	Diabetic retinopathy	ukb-a-248	finn-b-DM_RETINOPATHY_EXMORE	rs67844506	0.351	0.048	0.000
Body mass index	Diabetic retinopathy	ukb-a-248	finn-b-DM_RETINOPATHY_EXMORE	rs6789488	0.352	0.048	0.000
Body mass index	Diabetic retinopathy	ukb-a-248	finn-b-DM_RETINOPATHY_EXMORE	rs6809307	0.350	0.048	0.000
Body mass index	Diabetic retinopathy	ukb-a-248	finn-b-DM_RETINOPATHY_EXMORE	rs6831020	0.349	0.048	0.000

Body mass index	Diabetic retinopathy	ukb-a-248	finn-b-DM_RETINOPATHY_EXMORE	rs6861649	0.348	0.048	0.000
Body mass index	Diabetic retinopathy	ukb-a-248	finn-b-DM_RETINOPATHY_EXMORE	rs6950388	0.346	0.048	0.000
Body mass index	Diabetic retinopathy	ukb-a-248	finn-b-DM_RETINOPATHY_EXMORE	rs7006178	0.348	0.048	0.000
Body mass index	Diabetic retinopathy	ukb-a-248	finn-b-DM_RETINOPATHY_EXMORE	rs7030732	0.348	0.048	0.000
Body mass index	Diabetic retinopathy	ukb-a-248	finn-b-DM_RETINOPATHY_EXMORE	rs704061	0.349	0.048	0.000
Body mass index	Diabetic retinopathy	ukb-a-248	finn-b-DM_RETINOPATHY_EXMORE	rs7094644	0.353	0.048	0.000
Body mass index	Diabetic retinopathy	ukb-a-248	finn-b-DM_RETINOPATHY_EXMORE	rs7116641	0.347	0.048	0.000
Body mass index	Diabetic retinopathy	ukb-a-248	finn-b-DM_RETINOPATHY_EXMORE	rs7124681	0.349	0.048	0.000

Body mass index	Diabetic retinopathy	ukb-a-248	finn-b-DM_RETINOPATHY_EXMORE	rs7132908	0.349	0.048	0.000
Body mass index	Diabetic retinopathy	ukb-a-248	finn-b-DM_RETINOPATHY_EXMORE	rs7138383	0.348	0.048	0.000
Body mass index	Diabetic retinopathy	ukb-a-248	finn-b-DM_RETINOPATHY_EXMORE	rs7141420	0.346	0.048	0.000
Body mass index	Diabetic retinopathy	ukb-a-248	finn-b-DM_RETINOPATHY_EXMORE	rs71495049	0.347	0.048	0.000
Body mass index	Diabetic retinopathy	ukb-a-248	finn-b-DM_RETINOPATHY_EXMORE	rs7183417	0.344	0.048	0.000
Body mass index	Diabetic retinopathy	ukb-a-248	finn-b-DM_RETINOPATHY_EXMORE	rs7189149	0.354	0.048	0.000
Body mass index	Diabetic retinopathy	ukb-a-248	finn-b-DM_RETINOPATHY_EXMORE	rs7195386	0.349	0.048	0.000
Body mass index	Diabetic retinopathy	ukb-a-248	finn-b-DM_RETINOPATHY_EXMORE	rs7201895	0.348	0.048	0.000

Body mass index	Diabetic retinopathy	ukb-a-248	finn-b-DM_RETINOPATHY_EXMORE	rs7218014	0.347	0.048	0.000
Body mass index	Diabetic retinopathy	ukb-a-248	finn-b-DM_RETINOPATHY_EXMORE	rs72697614	0.350	0.048	0.000
Body mass index	Diabetic retinopathy	ukb-a-248	finn-b-DM_RETINOPATHY_EXMORE	rs72820274	0.351	0.048	0.000
Body mass index	Diabetic retinopathy	ukb-a-248	finn-b-DM_RETINOPATHY_EXMORE	rs72892910	0.347	0.048	0.000
Body mass index	Diabetic retinopathy	ukb-a-248	finn-b-DM_RETINOPATHY_EXMORE	rs72976986	0.344	0.048	0.000
Body mass index	Diabetic retinopathy	ukb-a-248	finn-b-DM_RETINOPATHY_EXMORE	rs73050254	0.350	0.048	0.000
Body mass index	Diabetic retinopathy	ukb-a-248	finn-b-DM_RETINOPATHY_EXMORE	rs73144053	0.349	0.048	0.000
Body mass index	Diabetic retinopathy	ukb-a-248	finn-b-DM_RETINOPATHY_EXMORE	rs73169730	0.354	0.048	0.000

Body mass index	Diabetic retinopathy	ukb-a-248	finn-b-DM_RETINOPATHY_EXMORE	rs7321331	0.349	0.048	0.000
Body mass index	Diabetic retinopathy	ukb-a-248	finn-b-DM_RETINOPATHY_EXMORE	rs73213484	0.349	0.048	0.000
Body mass index	Diabetic retinopathy	ukb-a-248	finn-b-DM_RETINOPATHY_EXMORE	rs7331420	0.349	0.048	0.000
Body mass index	Diabetic retinopathy	ukb-a-248	finn-b-DM_RETINOPATHY_EXMORE	rs7442885	0.351	0.048	0.000
Body mass index	Diabetic retinopathy	ukb-a-248	finn-b-DM_RETINOPATHY_EXMORE	rs7498044	0.350	0.048	0.000
Body mass index	Diabetic retinopathy	ukb-a-248	finn-b-DM_RETINOPATHY_EXMORE	rs750090	0.349	0.048	0.000
Body mass index	Diabetic retinopathy	ukb-a-248	finn-b-DM_RETINOPATHY_EXMORE	rs752179	0.348	0.048	0.000
Body mass index	Diabetic retinopathy	ukb-a-248	finn-b-DM_RETINOPATHY_EXMORE	rs75499503	0.351	0.048	0.000

Body mass index	Diabetic retinopathy	ukb-a-248	finn-b-DM_RETINOPATHY_EXMORE	rs7553158	0.348	0.048	0.000
Body mass index	Diabetic retinopathy	ukb-a-248	finn-b-DM_RETINOPATHY_EXMORE	rs75557510	0.346	0.048	0.000
Body mass index	Diabetic retinopathy	ukb-a-248	finn-b-DM_RETINOPATHY_EXMORE	rs756717	0.350	0.048	0.000
Body mass index	Diabetic retinopathy	ukb-a-248	finn-b-DM_RETINOPATHY_EXMORE	rs76040172	0.342	0.048	0.000
Body mass index	Diabetic retinopathy	ukb-a-248	finn-b-DM_RETINOPATHY_EXMORE	rs7701777	0.350	0.048	0.000
Body mass index	Diabetic retinopathy	ukb-a-248	finn-b-DM_RETINOPATHY_EXMORE	rs7719067	0.347	0.048	0.000
Body mass index	Diabetic retinopathy	ukb-a-248	finn-b-DM_RETINOPATHY_EXMORE	rs7723426	0.348	0.048	0.000
Body mass index	Diabetic retinopathy	ukb-a-248	finn-b-DM_RETINOPATHY_EXMORE	rs7755574	0.347	0.048	0.000

Body mass index	Diabetic retinopathy	ukb-a-248	finn-b-DM_RETINOPATHY_EXMORE	rs7774	0.349	0.048	0.000
Body mass index	Diabetic retinopathy	ukb-a-248	finn-b-DM_RETINOPATHY_EXMORE	rs778094	0.349	0.048	0.000
Body mass index	Diabetic retinopathy	ukb-a-248	finn-b-DM_RETINOPATHY_EXMORE	rs7852189	0.349	0.048	0.000
Body mass index	Diabetic retinopathy	ukb-a-248	finn-b-DM_RETINOPATHY_EXMORE	rs78565420	0.350	0.048	0.000
Body mass index	Diabetic retinopathy	ukb-a-248	finn-b-DM_RETINOPATHY_EXMORE	rs79113395	0.350	0.048	0.000
Body mass index	Diabetic retinopathy	ukb-a-248	finn-b-DM_RETINOPATHY_EXMORE	rs7933085	0.350	0.048	0.000
Body mass index	Diabetic retinopathy	ukb-a-248	finn-b-DM_RETINOPATHY_EXMORE	rs7941828	0.347	0.048	0.000
Body mass index	Diabetic retinopathy	ukb-a-248	finn-b-DM_RETINOPATHY_EXMORE	rs7952102	0.349	0.048	0.000

Body mass index	Diabetic retinopathy	ukb-a-248	finn-b-DM_RETINOPATHY_EXMORE	rs7992832	0.346	0.048	0.000
Body mass index	Diabetic retinopathy	ukb-a-248	finn-b-DM_RETINOPATHY_EXMORE	rs799449	0.352	0.048	0.000
Body mass index	Diabetic retinopathy	ukb-a-248	finn-b-DM_RETINOPATHY_EXMORE	rs8015400	0.348	0.048	0.000
Body mass index	Diabetic retinopathy	ukb-a-248	finn-b-DM_RETINOPATHY_EXMORE	rs80330591	0.349	0.048	0.000
Body mass index	Diabetic retinopathy	ukb-a-248	finn-b-DM_RETINOPATHY_EXMORE	rs8078135	0.347	0.048	0.000
Body mass index	Diabetic retinopathy	ukb-a-248	finn-b-DM_RETINOPATHY_EXMORE	rs8134638	0.348	0.048	0.000
Body mass index	Diabetic retinopathy	ukb-a-248	finn-b-DM_RETINOPATHY_EXMORE	rs815163	0.350	0.048	0.000
Body mass index	Diabetic retinopathy	ukb-a-248	finn-b-DM_RETINOPATHY_EXMORE	rs845084	0.347	0.048	0.000

Body mass index	Diabetic retinopathy	ukb-a-248	finn-b-DM_RETINOPATHY_EXMORE	rs862320	0.352	0.048	0.000
Body mass index	Diabetic retinopathy	ukb-a-248	finn-b-DM_RETINOPATHY_EXMORE	rs869400	0.345	0.048	0.000
Body mass index	Diabetic retinopathy	ukb-a-248	finn-b-DM_RETINOPATHY_EXMORE	rs879620	0.349	0.048	0.000
Body mass index	Diabetic retinopathy	ukb-a-248	finn-b-DM_RETINOPATHY_EXMORE	rs9267671	0.351	0.048	0.000
Body mass index	Diabetic retinopathy	ukb-a-248	finn-b-DM_RETINOPATHY_EXMORE	rs9291822	0.348	0.048	0.000
Body mass index	Diabetic retinopathy	ukb-a-248	finn-b-DM_RETINOPATHY_EXMORE	rs9320823	0.347	0.048	0.000
Body mass index	Diabetic retinopathy	ukb-a-248	finn-b-DM_RETINOPATHY_EXMORE	rs9342196	0.350	0.048	0.000
Body mass index	Diabetic retinopathy	ukb-a-248	finn-b-DM_RETINOPATHY_EXMORE	rs935166	0.347	0.048	0.000

Body mass index	Diabetic retinopathy	ukb-a-248	finn-b-<br>DM_RETINOPATHY_EXMORE	rs9402104	0.349	0.048	0.000
Body mass index	Diabetic retinopathy	ukb-a-248	finn-b-<br>DM_RETINOPATHY_EXMORE	rs946185	0.347	0.048	0.000
Body mass index	Diabetic retinopathy	ukb-a-248	finn-b-<br>DM_RETINOPATHY_EXMORE	rs9515455	0.350	0.048	0.000
Body mass index	Diabetic retinopathy	ukb-a-248	finn-b-<br>DM_RETINOPATHY_EXMORE	rs9527906	0.350	0.048	0.000
Body mass index	Diabetic retinopathy	ukb-a-248	finn-b-<br>DM_RETINOPATHY_EXMORE	rs9641499	0.351	0.048	0.000
Body mass index	Diabetic retinopathy	ukb-a-248	finn-b-<br>DM_RETINOPATHY_EXMORE	rs9688977	0.346	0.048	0.000
Body mass index	Diabetic retinopathy	ukb-a-248	finn-b-<br>DM_RETINOPATHY_EXMORE	rs9843653	0.353	0.048	0.000
Body mass index	Diabetic retinopathy	ukb-a-248	finn-b-<br>DM_RETINOPATHY_EXMORE	rs9847186	0.347	0.048	0.000

Body mass index	Diabetic retinopathy	ukb-a-248	finn-b-DM_RETINOPATHY_EXMORE	All	0.349	0.048	0.000
Body mass index	Proliferative diabetic retinopathy	ukb-a-248	finn-b-DM_RETINA_PROLIF	rs10100245	0.352	0.061	0.000
Body mass index	Proliferative diabetic retinopathy	ukb-a-248	finn-b-DM_RETINA_PROLIF	rs10187101	0.359	0.061	0.000
Body mass index	Proliferative diabetic retinopathy	ukb-a-248	finn-b-DM_RETINA_PROLIF	rs10404726	0.362	0.061	0.000
Body mass index	Proliferative diabetic retinopathy	ukb-a-248	finn-b-DM_RETINA_PROLIF	rs10465231	0.355	0.061	0.000
Body mass index	Proliferative diabetic retinopathy	ukb-a-248	finn-b-DM_RETINA_PROLIF	rs1064213	0.357	0.061	0.000
Body mass index	Proliferative diabetic retinopathy	ukb-a-248	finn-b-DM_RETINA_PROLIF	rs10788493	0.353	0.061	0.000
Body mass index	Proliferative diabetic retinopathy	ukb-a-248	finn-b-DM_RETINA_PROLIF	rs10803762	0.363	0.061	0.000

Body mass index	Proliferative diabetic retinopathy	ukb-a-248	finn-b-DM_RETINA_PROLIF	rs10805383	0.356	0.061	0.000
Body mass index	Proliferative diabetic retinopathy	ukb-a-248	finn-b-DM_RETINA_PROLIF	rs10865612	0.365	0.061	0.000
Body mass index	Proliferative diabetic retinopathy	ukb-a-248	finn-b-DM_RETINA_PROLIF	rs10898330	0.360	0.061	0.000
Body mass index	Proliferative diabetic retinopathy	ukb-a-248	finn-b-DM_RETINA_PROLIF	rs10938397	0.342	0.061	0.000
Body mass index	Proliferative diabetic retinopathy	ukb-a-248	finn-b-DM_RETINA_PROLIF	rs10995427	0.355	0.061	0.000
Body mass index	Proliferative diabetic retinopathy	ukb-a-248	finn-b-DM_RETINA_PROLIF	rs11012732	0.352	0.061	0.000
Body mass index	Proliferative diabetic retinopathy	ukb-a-248	finn-b-DM_RETINA_PROLIF	rs11078883	0.353	0.061	0.000
Body mass index	Proliferative diabetic retinopathy	ukb-a-248	finn-b-DM_RETINA_PROLIF	rs11084554	0.353	0.061	0.000

Body mass index	Proliferative diabetic retinopathy	ukb-a-248	finn-b-DM_RETINA_PROLIF	rs11099020	0.356	0.061	0.000
Body mass index	Proliferative diabetic retinopathy	ukb-a-248	finn-b-DM_RETINA_PROLIF	rs11150745	0.361	0.061	0.000
Body mass index	Proliferative diabetic retinopathy	ukb-a-248	finn-b-DM_RETINA_PROLIF	rs111640872	0.363	0.061	0.000
Body mass index	Proliferative diabetic retinopathy	ukb-a-248	finn-b-DM_RETINA_PROLIF	rs11223641	0.355	0.061	0.000
Body mass index	Proliferative diabetic retinopathy	ukb-a-248	finn-b-DM_RETINA_PROLIF	rs112520079	0.362	0.061	0.000
Body mass index	Proliferative diabetic retinopathy	ukb-a-248	finn-b-DM_RETINA_PROLIF	rs11264489	0.357	0.061	0.000
Body mass index	Proliferative diabetic retinopathy	ukb-a-248	finn-b-DM_RETINA_PROLIF	rs112693590	0.357	0.061	0.000
Body mass index	Proliferative diabetic retinopathy	ukb-a-248	finn-b-DM_RETINA_PROLIF	rs1127100	0.360	0.061	0.000

Body mass index	Proliferative diabetic retinopathy	ukb-a-248	finn-b-DM_RETINA_PROLIF	rs113182412	0.359	0.061	0.000
Body mass index	Proliferative diabetic retinopathy	ukb-a-248	finn-b-DM_RETINA_PROLIF	rs113230003	0.360	0.061	0.000
Body mass index	Proliferative diabetic retinopathy	ukb-a-248	finn-b-DM_RETINA_PROLIF	rs113603865	0.351	0.061	0.000
Body mass index	Proliferative diabetic retinopathy	ukb-a-248	finn-b-DM_RETINA_PROLIF	rs11515071	0.356	0.061	0.000
Body mass index	Proliferative diabetic retinopathy	ukb-a-248	finn-b-DM_RETINA_PROLIF	rs11642015	0.343	0.063	0.000
Body mass index	Proliferative diabetic retinopathy	ukb-a-248	finn-b-DM_RETINA_PROLIF	rs11650012	0.360	0.061	0.000
Body mass index	Proliferative diabetic retinopathy	ukb-a-248	finn-b-DM_RETINA_PROLIF	rs11655587	0.354	0.061	0.000
Body mass index	Proliferative diabetic retinopathy	ukb-a-248	finn-b-DM_RETINA_PROLIF	rs11742930	0.359	0.061	0.000

Body mass index	Proliferative diabetic retinopathy	ukb-a-248	finn-b-DM_RETINA_PROLIF	rs11757278	0.361	0.061	0.000
Body mass index	Proliferative diabetic retinopathy	ukb-a-248	finn-b-DM_RETINA_PROLIF	rs11761411	0.354	0.061	0.000
Body mass index	Proliferative diabetic retinopathy	ukb-a-248	finn-b-DM_RETINA_PROLIF	rs117632017	0.360	0.061	0.000
Body mass index	Proliferative diabetic retinopathy	ukb-a-248	finn-b-DM_RETINA_PROLIF	rs11782074	0.357	0.061	0.000
Body mass index	Proliferative diabetic retinopathy	ukb-a-248	finn-b-DM_RETINA_PROLIF	rs11856579	0.359	0.061	0.000
Body mass index	Proliferative diabetic retinopathy	ukb-a-248	finn-b-DM_RETINA_PROLIF	rs12024554	0.360	0.061	0.000
Body mass index	Proliferative diabetic retinopathy	ukb-a-248	finn-b-DM_RETINA_PROLIF	rs12042959	0.356	0.061	0.000
Body mass index	Proliferative diabetic retinopathy	ukb-a-248	finn-b-DM_RETINA_PROLIF	rs12049202	0.348	0.061	0.000

Body mass index	Proliferative diabetic retinopathy	ukb-a-248	finn-b-DM_RETINA_PROLIF	rs12140153	0.352	0.061	0.000
Body mass index	Proliferative diabetic retinopathy	ukb-a-248	finn-b-DM_RETINA_PROLIF	rs12144626	0.355	0.061	0.000
Body mass index	Proliferative diabetic retinopathy	ukb-a-248	finn-b-DM_RETINA_PROLIF	rs12477385	0.360	0.061	0.000
Body mass index	Proliferative diabetic retinopathy	ukb-a-248	finn-b-DM_RETINA_PROLIF	rs12479357	0.355	0.061	0.000
Body mass index	Proliferative diabetic retinopathy	ukb-a-248	finn-b-DM_RETINA_PROLIF	rs12614861	0.361	0.061	0.000
Body mass index	Proliferative diabetic retinopathy	ukb-a-248	finn-b-DM_RETINA_PROLIF	rs12622280	0.355	0.061	0.000
Body mass index	Proliferative diabetic retinopathy	ukb-a-248	finn-b-DM_RETINA_PROLIF	rs12662900	0.352	0.061	0.000
Body mass index	Proliferative diabetic retinopathy	ukb-a-248	finn-b-DM_RETINA_PROLIF	rs12679106	0.360	0.061	0.000

Body mass index	Proliferative diabetic retinopathy	ukb-a-248	finn-b-DM_RETINA_PROLIF	rs1286138	0.359	0.061	0.000
Body mass index	Proliferative diabetic retinopathy	ukb-a-248	finn-b-DM_RETINA_PROLIF	rs12877270	0.360	0.061	0.000
Body mass index	Proliferative diabetic retinopathy	ukb-a-248	finn-b-DM_RETINA_PROLIF	rs12881629	0.359	0.061	0.000
Body mass index	Proliferative diabetic retinopathy	ukb-a-248	finn-b-DM_RETINA_PROLIF	rs12885458	0.351	0.061	0.000
Body mass index	Proliferative diabetic retinopathy	ukb-a-248	finn-b-DM_RETINA_PROLIF	rs1296328	0.361	0.061	0.000
Body mass index	Proliferative diabetic retinopathy	ukb-a-248	finn-b-DM_RETINA_PROLIF	rs12977259	0.359	0.061	0.000
Body mass index	Proliferative diabetic retinopathy	ukb-a-248	finn-b-DM_RETINA_PROLIF	rs12992672	0.353	0.062	0.000
Body mass index	Proliferative diabetic retinopathy	ukb-a-248	finn-b-DM_RETINA_PROLIF	rs13062093	0.354	0.061	0.000

Body mass index	Proliferative diabetic retinopathy	ukb-a-248	finn-b-DM_RETINA_PROLIF	rs13076052	0.357	0.061	0.000
Body mass index	Proliferative diabetic retinopathy	ukb-a-248	finn-b-DM_RETINA_PROLIF	rs13135092	0.357	0.061	0.000
Body mass index	Proliferative diabetic retinopathy	ukb-a-248	finn-b-DM_RETINA_PROLIF	rs13174863	0.356	0.061	0.000
Body mass index	Proliferative diabetic retinopathy	ukb-a-248	finn-b-DM_RETINA_PROLIF	rs1320903	0.358	0.061	0.000
Body mass index	Proliferative diabetic retinopathy	ukb-a-248	finn-b-DM_RETINA_PROLIF	rs1327259	0.357	0.061	0.000
Body mass index	Proliferative diabetic retinopathy	ukb-a-248	finn-b-DM_RETINA_PROLIF	rs1342391	0.357	0.061	0.000
Body mass index	Proliferative diabetic retinopathy	ukb-a-248	finn-b-DM_RETINA_PROLIF	rs13427822	0.357	0.061	0.000
Body mass index	Proliferative diabetic retinopathy	ukb-a-248	finn-b-DM_RETINA_PROLIF	rs1411432	0.351	0.061	0.000

Body mass index	Proliferative diabetic retinopathy	ukb-a-248	finn-b-DM_RETINA_PROLIF	rs1412239	0.358	0.061	0.000
Body mass index	Proliferative diabetic retinopathy	ukb-a-248	finn-b-DM_RETINA_PROLIF	rs1441264	0.361	0.061	0.000
Body mass index	Proliferative diabetic retinopathy	ukb-a-248	finn-b-DM_RETINA_PROLIF	rs1446585	0.355	0.061	0.000
Body mass index	Proliferative diabetic retinopathy	ukb-a-248	finn-b-DM_RETINA_PROLIF	rs1458156	0.357	0.061	0.000
Body mass index	Proliferative diabetic retinopathy	ukb-a-248	finn-b-DM_RETINA_PROLIF	rs1477290	0.361	0.061	0.000
Body mass index	Proliferative diabetic retinopathy	ukb-a-248	finn-b-DM_RETINA_PROLIF	rs147730268	0.362	0.061	0.000
Body mass index	Proliferative diabetic retinopathy	ukb-a-248	finn-b-DM_RETINA_PROLIF	rs1582931	0.358	0.061	0.000
Body mass index	Proliferative diabetic retinopathy	ukb-a-248	finn-b-DM_RETINA_PROLIF	rs16846140	0.361	0.061	0.000

Body mass index	Proliferative diabetic retinopathy	ukb-a-248	finn-b-DM_RETINA_PROLIF	rs16916303	0.357	0.061	0.000
Body mass index	Proliferative diabetic retinopathy	ukb-a-248	finn-b-DM_RETINA_PROLIF	rs16975459	0.361	0.061	0.000
Body mass index	Proliferative diabetic retinopathy	ukb-a-248	finn-b-DM_RETINA_PROLIF	rs17014332	0.359	0.061	0.000
Body mass index	Proliferative diabetic retinopathy	ukb-a-248	finn-b-DM_RETINA_PROLIF	rs17024393	0.358	0.062	0.000
Body mass index	Proliferative diabetic retinopathy	ukb-a-248	finn-b-DM_RETINA_PROLIF	rs17058884	0.357	0.061	0.000
Body mass index	Proliferative diabetic retinopathy	ukb-a-248	finn-b-DM_RETINA_PROLIF	rs17085463	0.360	0.061	0.000
Body mass index	Proliferative diabetic retinopathy	ukb-a-248	finn-b-DM_RETINA_PROLIF	rs17149254	0.358	0.061	0.000
Body mass index	Proliferative diabetic retinopathy	ukb-a-248	finn-b-DM_RETINA_PROLIF	rs17342242	0.357	0.061	0.000

Body mass index	Proliferative diabetic retinopathy	ukb-a-248	finn-b-DM_RETINA_PROLIF	rs17399739	0.355	0.061	0.000
Body mass index	Proliferative diabetic retinopathy	ukb-a-248	finn-b-DM_RETINA_PROLIF	rs17716502	0.357	0.061	0.000
Body mass index	Proliferative diabetic retinopathy	ukb-a-248	finn-b-DM_RETINA_PROLIF	rs1788808	0.358	0.061	0.000
Body mass index	Proliferative diabetic retinopathy	ukb-a-248	finn-b-DM_RETINA_PROLIF	rs1805123	0.355	0.061	0.000
Body mass index	Proliferative diabetic retinopathy	ukb-a-248	finn-b-DM_RETINA_PROLIF	rs1884897	0.365	0.061	0.000
Body mass index	Proliferative diabetic retinopathy	ukb-a-248	finn-b-DM_RETINA_PROLIF	rs1901241	0.358	0.061	0.000
Body mass index	Proliferative diabetic retinopathy	ukb-a-248	finn-b-DM_RETINA_PROLIF	rs1919243	0.358	0.061	0.000
Body mass index	Proliferative diabetic retinopathy	ukb-a-248	finn-b-DM_RETINA_PROLIF	rs1941706	0.358	0.061	0.000

Body mass index	Proliferative diabetic retinopathy	ukb-a-248	finn-b-DM_RETINA_PROLIF	rs1949204	0.358	0.061	0.000
Body mass index	Proliferative diabetic retinopathy	ukb-a-248	finn-b-DM_RETINA_PROLIF	rs2035806	0.360	0.061	0.000
Body mass index	Proliferative diabetic retinopathy	ukb-a-248	finn-b-DM_RETINA_PROLIF	rs2046002	0.356	0.061	0.000
Body mass index	Proliferative diabetic retinopathy	ukb-a-248	finn-b-DM_RETINA_PROLIF	rs2121058	0.353	0.061	0.000
Body mass index	Proliferative diabetic retinopathy	ukb-a-248	finn-b-DM_RETINA_PROLIF	rs2135745	0.358	0.061	0.000
Body mass index	Proliferative diabetic retinopathy	ukb-a-248	finn-b-DM_RETINA_PROLIF	rs2155869	0.361	0.061	0.000
Body mass index	Proliferative diabetic retinopathy	ukb-a-248	finn-b-DM_RETINA_PROLIF	rs215634	0.355	0.061	0.000
Body mass index	Proliferative diabetic retinopathy	ukb-a-248	finn-b-DM_RETINA_PROLIF	rs2192649	0.357	0.061	0.000

Body mass index	Proliferative diabetic retinopathy	ukb-a-248	finn-b-DM_RETINA_PROLIF	rs2234458	0.358	0.061	0.000
Body mass index	Proliferative diabetic retinopathy	ukb-a-248	finn-b-DM_RETINA_PROLIF	rs2292238	0.362	0.061	0.000
Body mass index	Proliferative diabetic retinopathy	ukb-a-248	finn-b-DM_RETINA_PROLIF	rs2307111	0.350	0.061	0.000
Body mass index	Proliferative diabetic retinopathy	ukb-a-248	finn-b-DM_RETINA_PROLIF	rs2318543	0.353	0.061	0.000
Body mass index	Proliferative diabetic retinopathy	ukb-a-248	finn-b-DM_RETINA_PROLIF	rs2384054	0.350	0.062	0.000
Body mass index	Proliferative diabetic retinopathy	ukb-a-248	finn-b-DM_RETINA_PROLIF	rs2398861	0.359	0.061	0.000
Body mass index	Proliferative diabetic retinopathy	ukb-a-248	finn-b-DM_RETINA_PROLIF	rs241460	0.361	0.061	0.000
Body mass index	Proliferative diabetic retinopathy	ukb-a-248	finn-b-DM_RETINA_PROLIF	rs2425857	0.354	0.061	0.000

Body mass index	Proliferative diabetic retinopathy	ukb-a-248	finn-b-DM_RETINA_PROLIF	rs2439823	0.357	0.061	0.000
Body mass index	Proliferative diabetic retinopathy	ukb-a-248	finn-b-DM_RETINA_PROLIF	rs2450254	0.355	0.061	0.000
Body mass index	Proliferative diabetic retinopathy	ukb-a-248	finn-b-DM_RETINA_PROLIF	rs2450445	0.358	0.061	0.000
Body mass index	Proliferative diabetic retinopathy	ukb-a-248	finn-b-DM_RETINA_PROLIF	rs245775	0.359	0.061	0.000
Body mass index	Proliferative diabetic retinopathy	ukb-a-248	finn-b-DM_RETINA_PROLIF	rs2470392	0.357	0.061	0.000
Body mass index	Proliferative diabetic retinopathy	ukb-a-248	finn-b-DM_RETINA_PROLIF	rs2474898	0.357	0.061	0.000
Body mass index	Proliferative diabetic retinopathy	ukb-a-248	finn-b-DM_RETINA_PROLIF	rs2482704	0.357	0.061	0.000
Body mass index	Proliferative diabetic retinopathy	ukb-a-248	finn-b-DM_RETINA_PROLIF	rs2606228	0.355	0.061	0.000

Body mass index	Proliferative diabetic retinopathy	ukb-a-248	finn-b-DM_RETINA_PROLIF	rs2616192	0.354	0.061	0.000
Body mass index	Proliferative diabetic retinopathy	ukb-a-248	finn-b-DM_RETINA_PROLIF	rs2678204	0.362	0.061	0.000
Body mass index	Proliferative diabetic retinopathy	ukb-a-248	finn-b-DM_RETINA_PROLIF	rs2711111	0.360	0.061	0.000
Body mass index	Proliferative diabetic retinopathy	ukb-a-248	finn-b-DM_RETINA_PROLIF	rs2725371	0.354	0.061	0.000
Body mass index	Proliferative diabetic retinopathy	ukb-a-248	finn-b-DM_RETINA_PROLIF	rs273505	0.356	0.061	0.000
Body mass index	Proliferative diabetic retinopathy	ukb-a-248	finn-b-DM_RETINA_PROLIF	rs28366156	0.343	0.059	0.000
Body mass index	Proliferative diabetic retinopathy	ukb-a-248	finn-b-DM_RETINA_PROLIF	rs28447555	0.356	0.061	0.000
Body mass index	Proliferative diabetic retinopathy	ukb-a-248	finn-b-DM_RETINA_PROLIF	rs28489620	0.360	0.061	0.000

Body mass index	Proliferative diabetic retinopathy	ukb-a-248	finn-b-DM_RETINA_PROLIF	rs2861685	0.357	0.061	0.000
Body mass index	Proliferative diabetic retinopathy	ukb-a-248	finn-b-DM_RETINA_PROLIF	rs286818	0.370	0.061	0.000
Body mass index	Proliferative diabetic retinopathy	ukb-a-248	finn-b-DM_RETINA_PROLIF	rs2962082	0.358	0.061	0.000
Body mass index	Proliferative diabetic retinopathy	ukb-a-248	finn-b-DM_RETINA_PROLIF	rs2975693	0.357	0.061	0.000
Body mass index	Proliferative diabetic retinopathy	ukb-a-248	finn-b-DM_RETINA_PROLIF	rs34045288	0.348	0.061	0.000
Body mass index	Proliferative diabetic retinopathy	ukb-a-248	finn-b-DM_RETINA_PROLIF	rs34095326	0.358	0.061	0.000
Body mass index	Proliferative diabetic retinopathy	ukb-a-248	finn-b-DM_RETINA_PROLIF	rs34236292	0.357	0.061	0.000
Body mass index	Proliferative diabetic retinopathy	ukb-a-248	finn-b-DM_RETINA_PROLIF	rs34361149	0.362	0.061	0.000

Body mass index	Proliferative diabetic retinopathy	ukb-a-248	finn-b-DM_RETINA_PROLIF	rs34373881	0.355	0.061	0.000
Body mass index	Proliferative diabetic retinopathy	ukb-a-248	finn-b-DM_RETINA_PROLIF	rs34774377	0.358	0.061	0.000
Body mass index	Proliferative diabetic retinopathy	ukb-a-248	finn-b-DM_RETINA_PROLIF	rs34811474	0.358	0.061	0.000
Body mass index	Proliferative diabetic retinopathy	ukb-a-248	finn-b-DM_RETINA_PROLIF	rs34966008	0.354	0.061	0.000
Body mass index	Proliferative diabetic retinopathy	ukb-a-248	finn-b-DM_RETINA_PROLIF	rs35025195	0.355	0.061	0.000
Body mass index	Proliferative diabetic retinopathy	ukb-a-248	finn-b-DM_RETINA_PROLIF	rs35193668	0.350	0.061	0.000
Body mass index	Proliferative diabetic retinopathy	ukb-a-248	finn-b-DM_RETINA_PROLIF	rs35483388	0.356	0.061	0.000
Body mass index	Proliferative diabetic retinopathy	ukb-a-248	finn-b-DM_RETINA_PROLIF	rs35626515	0.370	0.061	0.000

Body mass index	Proliferative diabetic retinopathy	ukb-a-248	finn-b-DM_RETINA_PROLIF	rs35722922	0.356	0.061	0.000
Body mass index	Proliferative diabetic retinopathy	ukb-a-248	finn-b-DM_RETINA_PROLIF	rs357501	0.363	0.061	0.000
Body mass index	Proliferative diabetic retinopathy	ukb-a-248	finn-b-DM_RETINA_PROLIF	rs35851183	0.353	0.061	0.000
Body mass index	Proliferative diabetic retinopathy	ukb-a-248	finn-b-DM_RETINA_PROLIF	rs35882248	0.358	0.061	0.000
Body mass index	Proliferative diabetic retinopathy	ukb-a-248	finn-b-DM_RETINA_PROLIF	rs36007635	0.357	0.061	0.000
Body mass index	Proliferative diabetic retinopathy	ukb-a-248	finn-b-DM_RETINA_PROLIF	rs362307	0.355	0.061	0.000
Body mass index	Proliferative diabetic retinopathy	ukb-a-248	finn-b-DM_RETINA_PROLIF	rs3759584	0.354	0.061	0.000
Body mass index	Proliferative diabetic retinopathy	ukb-a-248	finn-b-DM_RETINA_PROLIF	rs3802858	0.353	0.061	0.000

Body mass index	Proliferative diabetic retinopathy	ukb-a-248	finn-b-DM_RETINA_PROLIF	rs3803286	0.355	0.061	0.000
Body mass index	Proliferative diabetic retinopathy	ukb-a-248	finn-b-DM_RETINA_PROLIF	rs3810291	0.353	0.062	0.000
Body mass index	Proliferative diabetic retinopathy	ukb-a-248	finn-b-DM_RETINA_PROLIF	rs3843540	0.370	0.060	0.000
Body mass index	Proliferative diabetic retinopathy	ukb-a-248	finn-b-DM_RETINA_PROLIF	rs3844598	0.356	0.061	0.000
Body mass index	Proliferative diabetic retinopathy	ukb-a-248	finn-b-DM_RETINA_PROLIF	rs3861879	0.356	0.061	0.000
Body mass index	Proliferative diabetic retinopathy	ukb-a-248	finn-b-DM_RETINA_PROLIF	rs3897102	0.355	0.061	0.000
Body mass index	Proliferative diabetic retinopathy	ukb-a-248	finn-b-DM_RETINA_PROLIF	rs390192	0.355	0.061	0.000
Body mass index	Proliferative diabetic retinopathy	ukb-a-248	finn-b-DM_RETINA_PROLIF	rs4246657	0.355	0.061	0.000

Body mass index	Proliferative diabetic retinopathy	ukb-a-248	finn-b-DM_RETINA_PROLIF	rs4261944	0.355	0.061	0.000
Body mass index	Proliferative diabetic retinopathy	ukb-a-248	finn-b-DM_RETINA_PROLIF	rs4402589	0.365	0.061	0.000
Body mass index	Proliferative diabetic retinopathy	ukb-a-248	finn-b-DM_RETINA_PROLIF	rs4467770	0.354	0.061	0.000
Body mass index	Proliferative diabetic retinopathy	ukb-a-248	finn-b-DM_RETINA_PROLIF	rs4474229	0.355	0.061	0.000
Body mass index	Proliferative diabetic retinopathy	ukb-a-248	finn-b-DM_RETINA_PROLIF	rs4482463	0.357	0.061	0.000
Body mass index	Proliferative diabetic retinopathy	ukb-a-248	finn-b-DM_RETINA_PROLIF	rs4502882	0.360	0.061	0.000
Body mass index	Proliferative diabetic retinopathy	ukb-a-248	finn-b-DM_RETINA_PROLIF	rs4595495	0.356	0.061	0.000
Body mass index	Proliferative diabetic retinopathy	ukb-a-248	finn-b-DM_RETINA_PROLIF	rs4648450	0.360	0.061	0.000

Body mass index	Proliferative diabetic retinopathy	ukb-a-248	finn-b-DM_RETINA_PROLIF	rs4687770	0.359	0.061	0.000
Body mass index	Proliferative diabetic retinopathy	ukb-a-248	finn-b-DM_RETINA_PROLIF	rs4718964	0.362	0.061	0.000
Body mass index	Proliferative diabetic retinopathy	ukb-a-248	finn-b-DM_RETINA_PROLIF	rs4757144	0.359	0.061	0.000
Body mass index	Proliferative diabetic retinopathy	ukb-a-248	finn-b-DM_RETINA_PROLIF	rs4776970	0.357	0.061	0.000
Body mass index	Proliferative diabetic retinopathy	ukb-a-248	finn-b-DM_RETINA_PROLIF	rs4777541	0.354	0.061	0.000
Body mass index	Proliferative diabetic retinopathy	ukb-a-248	finn-b-DM_RETINA_PROLIF	rs4911382	0.357	0.061	0.000
Body mass index	Proliferative diabetic retinopathy	ukb-a-248	finn-b-DM_RETINA_PROLIF	rs491711	0.357	0.061	0.000
Body mass index	Proliferative diabetic retinopathy	ukb-a-248	finn-b-DM_RETINA_PROLIF	rs4921301	0.357	0.061	0.000

Body mass index	Proliferative diabetic retinopathy	ukb-a-248	finn-b-DM_RETINA_PROLIF	rs4929923	0.358	0.061	0.000
Body mass index	Proliferative diabetic retinopathy	ukb-a-248	finn-b-DM_RETINA_PROLIF	rs525101	0.357	0.061	0.000
Body mass index	Proliferative diabetic retinopathy	ukb-a-248	finn-b-DM_RETINA_PROLIF	rs539515	0.363	0.062	0.000
Body mass index	Proliferative diabetic retinopathy	ukb-a-248	finn-b-DM_RETINA_PROLIF	rs550974	0.357	0.061	0.000
Body mass index	Proliferative diabetic retinopathy	ukb-a-248	finn-b-DM_RETINA_PROLIF	rs55689274	0.359	0.061	0.000
Body mass index	Proliferative diabetic retinopathy	ukb-a-248	finn-b-DM_RETINA_PROLIF	rs55726687	0.359	0.061	0.000
Body mass index	Proliferative diabetic retinopathy	ukb-a-248	finn-b-DM_RETINA_PROLIF	rs55886426	0.355	0.061	0.000
Body mass index	Proliferative diabetic retinopathy	ukb-a-248	finn-b-DM_RETINA_PROLIF	rs55938344	0.362	0.061	0.000

Body mass index	Proliferative diabetic retinopathy	ukb-a-248	finn-b-DM_RETINA_PROLIF	rs56067609	0.356	0.061	0.000
Body mass index	Proliferative diabetic retinopathy	ukb-a-248	finn-b-DM_RETINA_PROLIF	rs56161855	0.369	0.060	0.000
Body mass index	Proliferative diabetic retinopathy	ukb-a-248	finn-b-DM_RETINA_PROLIF	rs56212061	0.357	0.061	0.000
Body mass index	Proliferative diabetic retinopathy	ukb-a-248	finn-b-DM_RETINA_PROLIF	rs56773984	0.357	0.061	0.000
Body mass index	Proliferative diabetic retinopathy	ukb-a-248	finn-b-DM_RETINA_PROLIF	rs56803094	0.356	0.061	0.000
Body mass index	Proliferative diabetic retinopathy	ukb-a-248	finn-b-DM_RETINA_PROLIF	rs57636386	0.357	0.061	0.000
Body mass index	Proliferative diabetic retinopathy	ukb-a-248	finn-b-DM_RETINA_PROLIF	rs58862095	0.358	0.061	0.000
Body mass index	Proliferative diabetic retinopathy	ukb-a-248	finn-b-DM_RETINA_PROLIF	rs588660	0.360	0.061	0.000

Body mass index	Proliferative diabetic retinopathy	ukb-a-248	finn-b-DM_RETINA_PROLIF	rs59104534	0.356	0.061	0.000
Body mass index	Proliferative diabetic retinopathy	ukb-a-248	finn-b-DM_RETINA_PROLIF	rs5995843	0.359	0.061	0.000
Body mass index	Proliferative diabetic retinopathy	ukb-a-248	finn-b-DM_RETINA_PROLIF	rs6050446	0.357	0.061	0.000
Body mass index	Proliferative diabetic retinopathy	ukb-a-248	finn-b-DM_RETINA_PROLIF	rs60654199	0.360	0.061	0.000
Body mass index	Proliferative diabetic retinopathy	ukb-a-248	finn-b-DM_RETINA_PROLIF	rs60764613	0.354	0.061	0.000
Body mass index	Proliferative diabetic retinopathy	ukb-a-248	finn-b-DM_RETINA_PROLIF	rs61813324	0.361	0.061	0.000
Body mass index	Proliferative diabetic retinopathy	ukb-a-248	finn-b-DM_RETINA_PROLIF	rs61826867	0.352	0.061	0.000
Body mass index	Proliferative diabetic retinopathy	ukb-a-248	finn-b-DM_RETINA_PROLIF	rs61871615	0.354	0.061	0.000

Body mass index	Proliferative diabetic retinopathy	ukb-a-248	finn-b-DM_RETINA_PROLIF	rs61903695	0.357	0.061	0.000
Body mass index	Proliferative diabetic retinopathy	ukb-a-248	finn-b-DM_RETINA_PROLIF	rs61969510	0.354	0.061	0.000
Body mass index	Proliferative diabetic retinopathy	ukb-a-248	finn-b-DM_RETINA_PROLIF	rs62106258	0.355	0.061	0.000
Body mass index	Proliferative diabetic retinopathy	ukb-a-248	finn-b-DM_RETINA_PROLIF	rs62147189	0.357	0.061	0.000
Body mass index	Proliferative diabetic retinopathy	ukb-a-248	finn-b-DM_RETINA_PROLIF	rs62246314	0.359	0.061	0.000
Body mass index	Proliferative diabetic retinopathy	ukb-a-248	finn-b-DM_RETINA_PROLIF	rs62407562	0.355	0.061	0.000
Body mass index	Proliferative diabetic retinopathy	ukb-a-248	finn-b-DM_RETINA_PROLIF	rs62543438	0.355	0.061	0.000
Body mass index	Proliferative diabetic retinopathy	ukb-a-248	finn-b-DM_RETINA_PROLIF	rs6265	0.367	0.061	0.000

Body mass index	Proliferative diabetic retinopathy	ukb-a-248	finn-b-DM_RETINA_PROLIF	rs6536575	0.361	0.061	0.000
Body mass index	Proliferative diabetic retinopathy	ukb-a-248	finn-b-DM_RETINA_PROLIF	rs6575340	0.357	0.061	0.000
Body mass index	Proliferative diabetic retinopathy	ukb-a-248	finn-b-DM_RETINA_PROLIF	rs6601527	0.357	0.061	0.000
Body mass index	Proliferative diabetic retinopathy	ukb-a-248	finn-b-DM_RETINA_PROLIF	rs66679256	0.360	0.061	0.000
Body mass index	Proliferative diabetic retinopathy	ukb-a-248	finn-b-DM_RETINA_PROLIF	rs6687953	0.358	0.061	0.000
Body mass index	Proliferative diabetic retinopathy	ukb-a-248	finn-b-DM_RETINA_PROLIF	rs66922415	0.351	0.062	0.000
Body mass index	Proliferative diabetic retinopathy	ukb-a-248	finn-b-DM_RETINA_PROLIF	rs6705567	0.356	0.061	0.000
Body mass index	Proliferative diabetic retinopathy	ukb-a-248	finn-b-DM_RETINA_PROLIF	rs6722241	0.351	0.061	0.000

Body mass index	Proliferative diabetic retinopathy	ukb-a-248	finn-b-DM_RETINA_PROLIF	rs6739755	0.351	0.061	0.000
Body mass index	Proliferative diabetic retinopathy	ukb-a-248	finn-b-DM_RETINA_PROLIF	rs67609008	0.358	0.061	0.000
Body mass index	Proliferative diabetic retinopathy	ukb-a-248	finn-b-DM_RETINA_PROLIF	rs6780459	0.355	0.061	0.000
Body mass index	Proliferative diabetic retinopathy	ukb-a-248	finn-b-DM_RETINA_PROLIF	rs67844506	0.360	0.061	0.000
Body mass index	Proliferative diabetic retinopathy	ukb-a-248	finn-b-DM_RETINA_PROLIF	rs6789488	0.361	0.061	0.000
Body mass index	Proliferative diabetic retinopathy	ukb-a-248	finn-b-DM_RETINA_PROLIF	rs6809307	0.358	0.061	0.000
Body mass index	Proliferative diabetic retinopathy	ukb-a-248	finn-b-DM_RETINA_PROLIF	rs6831020	0.358	0.061	0.000
Body mass index	Proliferative diabetic retinopathy	ukb-a-248	finn-b-DM_RETINA_PROLIF	rs6861649	0.358	0.061	0.000

Body mass index	Proliferative diabetic retinopathy	ukb-a-248	finn-b-DM_RETINA_PROLIF	rs6950388	0.354	0.061	0.000
Body mass index	Proliferative diabetic retinopathy	ukb-a-248	finn-b-DM_RETINA_PROLIF	rs7006178	0.355	0.061	0.000
Body mass index	Proliferative diabetic retinopathy	ukb-a-248	finn-b-DM_RETINA_PROLIF	rs7030732	0.358	0.061	0.000
Body mass index	Proliferative diabetic retinopathy	ukb-a-248	finn-b-DM_RETINA_PROLIF	rs704061	0.359	0.061	0.000
Body mass index	Proliferative diabetic retinopathy	ukb-a-248	finn-b-DM_RETINA_PROLIF	rs7094644	0.362	0.061	0.000
Body mass index	Proliferative diabetic retinopathy	ukb-a-248	finn-b-DM_RETINA_PROLIF	rs7116641	0.354	0.061	0.000
Body mass index	Proliferative diabetic retinopathy	ukb-a-248	finn-b-DM_RETINA_PROLIF	rs7124681	0.357	0.061	0.000
Body mass index	Proliferative diabetic retinopathy	ukb-a-248	finn-b-DM_RETINA_PROLIF	rs7132908	0.360	0.062	0.000

Body mass index	Proliferative diabetic retinopathy	ukb-a-248	finn-b-DM_RETINA_PROLIF	rs7138383	0.359	0.061	0.000
Body mass index	Proliferative diabetic retinopathy	ukb-a-248	finn-b-DM_RETINA_PROLIF	rs7141420	0.355	0.061	0.000
Body mass index	Proliferative diabetic retinopathy	ukb-a-248	finn-b-DM_RETINA_PROLIF	rs71495049	0.356	0.061	0.000
Body mass index	Proliferative diabetic retinopathy	ukb-a-248	finn-b-DM_RETINA_PROLIF	rs7183417	0.353	0.061	0.000
Body mass index	Proliferative diabetic retinopathy	ukb-a-248	finn-b-DM_RETINA_PROLIF	rs7189149	0.363	0.061	0.000
Body mass index	Proliferative diabetic retinopathy	ukb-a-248	finn-b-DM_RETINA_PROLIF	rs7195386	0.359	0.061	0.000
Body mass index	Proliferative diabetic retinopathy	ukb-a-248	finn-b-DM_RETINA_PROLIF	rs7201895	0.359	0.061	0.000
Body mass index	Proliferative diabetic retinopathy	ukb-a-248	finn-b-DM_RETINA_PROLIF	rs7218014	0.354	0.061	0.000

Body mass index	Proliferative diabetic retinopathy	ukb-a-248	finn-b-DM_RETINA_PROLIF	rs72697614	0.359	0.061	0.000
Body mass index	Proliferative diabetic retinopathy	ukb-a-248	finn-b-DM_RETINA_PROLIF	rs72820274	0.360	0.061	0.000
Body mass index	Proliferative diabetic retinopathy	ukb-a-248	finn-b-DM_RETINA_PROLIF	rs72892910	0.353	0.062	0.000
Body mass index	Proliferative diabetic retinopathy	ukb-a-248	finn-b-DM_RETINA_PROLIF	rs72976986	0.351	0.061	0.000
Body mass index	Proliferative diabetic retinopathy	ukb-a-248	finn-b-DM_RETINA_PROLIF	rs73050254	0.359	0.061	0.000
Body mass index	Proliferative diabetic retinopathy	ukb-a-248	finn-b-DM_RETINA_PROLIF	rs73144053	0.355	0.061	0.000
Body mass index	Proliferative diabetic retinopathy	ukb-a-248	finn-b-DM_RETINA_PROLIF	rs73169730	0.362	0.061	0.000
Body mass index	Proliferative diabetic retinopathy	ukb-a-248	finn-b-DM_RETINA_PROLIF	rs7321331	0.356	0.061	0.000

Body mass index	Proliferative diabetic retinopathy	ukb-a-248	finn-b-DM_RETINA_PROLIF	rs73213484	0.357	0.061	0.000
Body mass index	Proliferative diabetic retinopathy	ukb-a-248	finn-b-DM_RETINA_PROLIF	rs7331420	0.359	0.061	0.000
Body mass index	Proliferative diabetic retinopathy	ukb-a-248	finn-b-DM_RETINA_PROLIF	rs7442885	0.366	0.061	0.000
Body mass index	Proliferative diabetic retinopathy	ukb-a-248	finn-b-DM_RETINA_PROLIF	rs7498044	0.356	0.061	0.000
Body mass index	Proliferative diabetic retinopathy	ukb-a-248	finn-b-DM_RETINA_PROLIF	rs750090	0.356	0.061	0.000
Body mass index	Proliferative diabetic retinopathy	ukb-a-248	finn-b-DM_RETINA_PROLIF	rs752179	0.357	0.061	0.000
Body mass index	Proliferative diabetic retinopathy	ukb-a-248	finn-b-DM_RETINA_PROLIF	rs75499503	0.360	0.061	0.000
Body mass index	Proliferative diabetic retinopathy	ukb-a-248	finn-b-DM_RETINA_PROLIF	rs7553158	0.357	0.061	0.000

Body mass index	Proliferative diabetic retinopathy	ukb-a-248	finn-b-DM_RETINA_PROLIF	rs75557510	0.355	0.061	0.000
Body mass index	Proliferative diabetic retinopathy	ukb-a-248	finn-b-DM_RETINA_PROLIF	rs756717	0.360	0.061	0.000
Body mass index	Proliferative diabetic retinopathy	ukb-a-248	finn-b-DM_RETINA_PROLIF	rs76040172	0.347	0.061	0.000
Body mass index	Proliferative diabetic retinopathy	ukb-a-248	finn-b-DM_RETINA_PROLIF	rs7701777	0.357	0.061	0.000
Body mass index	Proliferative diabetic retinopathy	ukb-a-248	finn-b-DM_RETINA_PROLIF	rs7719067	0.356	0.061	0.000
Body mass index	Proliferative diabetic retinopathy	ukb-a-248	finn-b-DM_RETINA_PROLIF	rs7723426	0.356	0.061	0.000
Body mass index	Proliferative diabetic retinopathy	ukb-a-248	finn-b-DM_RETINA_PROLIF	rs7755574	0.356	0.061	0.000
Body mass index	Proliferative diabetic retinopathy	ukb-a-248	finn-b-DM_RETINA_PROLIF	rs7774	0.358	0.061	0.000

Body mass index	Proliferative diabetic retinopathy	ukb-a-248	finn-b-DM_RETINA_PROLIF	rs778094	0.356	0.061	0.000
Body mass index	Proliferative diabetic retinopathy	ukb-a-248	finn-b-DM_RETINA_PROLIF	rs7852189	0.355	0.061	0.000
Body mass index	Proliferative diabetic retinopathy	ukb-a-248	finn-b-DM_RETINA_PROLIF	rs78565420	0.355	0.061	0.000
Body mass index	Proliferative diabetic retinopathy	ukb-a-248	finn-b-DM_RETINA_PROLIF	rs79113395	0.357	0.061	0.000
Body mass index	Proliferative diabetic retinopathy	ukb-a-248	finn-b-DM_RETINA_PROLIF	rs7933085	0.361	0.061	0.000
Body mass index	Proliferative diabetic retinopathy	ukb-a-248	finn-b-DM_RETINA_PROLIF	rs7941828	0.358	0.061	0.000
Body mass index	Proliferative diabetic retinopathy	ukb-a-248	finn-b-DM_RETINA_PROLIF	rs7952102	0.356	0.061	0.000
Body mass index	Proliferative diabetic retinopathy	ukb-a-248	finn-b-DM_RETINA_PROLIF	rs7992832	0.354	0.061	0.000

Body mass index	Proliferative diabetic retinopathy	ukb-a-248	finn-b-DM_RETINA_PROLIF	rs799449	0.361	0.061	0.000
Body mass index	Proliferative diabetic retinopathy	ukb-a-248	finn-b-DM_RETINA_PROLIF	rs8015400	0.355	0.061	0.000
Body mass index	Proliferative diabetic retinopathy	ukb-a-248	finn-b-DM_RETINA_PROLIF	rs80330591	0.355	0.061	0.000
Body mass index	Proliferative diabetic retinopathy	ukb-a-248	finn-b-DM_RETINA_PROLIF	rs8078135	0.355	0.061	0.000
Body mass index	Proliferative diabetic retinopathy	ukb-a-248	finn-b-DM_RETINA_PROLIF	rs8134638	0.356	0.061	0.000
Body mass index	Proliferative diabetic retinopathy	ukb-a-248	finn-b-DM_RETINA_PROLIF	rs815163	0.361	0.061	0.000
Body mass index	Proliferative diabetic retinopathy	ukb-a-248	finn-b-DM_RETINA_PROLIF	rs845084	0.355	0.061	0.000
Body mass index	Proliferative diabetic retinopathy	ukb-a-248	finn-b-DM_RETINA_PROLIF	rs862320	0.355	0.061	0.000

Body mass index	Proliferative diabetic retinopathy	ukb-a-248	finn-b-DM_RETINA_PROLIF	rs869400	0.354	0.061	0.000
Body mass index	Proliferative diabetic retinopathy	ukb-a-248	finn-b-DM_RETINA_PROLIF	rs879620	0.355	0.061	0.000
Body mass index	Proliferative diabetic retinopathy	ukb-a-248	finn-b-DM_RETINA_PROLIF	rs9267671	0.361	0.061	0.000
Body mass index	Proliferative diabetic retinopathy	ukb-a-248	finn-b-DM_RETINA_PROLIF	rs9291822	0.356	0.061	0.000
Body mass index	Proliferative diabetic retinopathy	ukb-a-248	finn-b-DM_RETINA_PROLIF	rs9320823	0.358	0.061	0.000
Body mass index	Proliferative diabetic retinopathy	ukb-a-248	finn-b-DM_RETINA_PROLIF	rs9342196	0.360	0.061	0.000
Body mass index	Proliferative diabetic retinopathy	ukb-a-248	finn-b-DM_RETINA_PROLIF	rs935166	0.357	0.061	0.000
Body mass index	Proliferative diabetic retinopathy	ukb-a-248	finn-b-DM_RETINA_PROLIF	rs9402104	0.359	0.061	0.000

Body mass index	Proliferative diabetic retinopathy	ukb-a-248	finn-b-DM_RETINA_PROLIF	rs946185	0.354	0.061	0.000
Body mass index	Proliferative diabetic retinopathy	ukb-a-248	finn-b-DM_RETINA_PROLIF	rs9515455	0.360	0.061	0.000
Body mass index	Proliferative diabetic retinopathy	ukb-a-248	finn-b-DM_RETINA_PROLIF	rs9527906	0.361	0.061	0.000
Body mass index	Proliferative diabetic retinopathy	ukb-a-248	finn-b-DM_RETINA_PROLIF	rs9641499	0.357	0.061	0.000
Body mass index	Proliferative diabetic retinopathy	ukb-a-248	finn-b-DM_RETINA_PROLIF	rs9688977	0.355	0.061	0.000
Body mass index	Proliferative diabetic retinopathy	ukb-a-248	finn-b-DM_RETINA_PROLIF	rs9843653	0.361	0.062	0.000
Body mass index	Proliferative diabetic retinopathy	ukb-a-248	finn-b-DM_RETINA_PROLIF	rs9847186	0.355	0.061	0.000
Body mass index	Proliferative diabetic retinopathy	ukb-a-248	finn-b-DM_RETINA_PROLIF	All	0.357	0.061	0.000

Cigarettes per Day	Background diabetic retinopathy	ieu-b-25	finn-b-DM_BCKGRND_RETINA	rs11725618	0.112	0.125	0.374
Cigarettes per Day	Background diabetic retinopathy	ieu-b-25	finn-b-DM_BCKGRND_RETINA	rs1579233	0.132	0.125	0.293
Cigarettes per Day	Background diabetic retinopathy	ieu-b-25	finn-b-DM_BCKGRND_RETINA	rs2072659	0.104	0.126	0.408
Cigarettes per Day	Background diabetic retinopathy	ieu-b-25	finn-b-DM_BCKGRND_RETINA	rs2084533	0.111	0.125	0.375
Cigarettes per Day	Background diabetic retinopathy	ieu-b-25	finn-b-DM_BCKGRND_RETINA	rs215600	0.117	0.126	0.355
Cigarettes per Day	Background diabetic retinopathy	ieu-b-25	finn-b-DM_BCKGRND_RETINA	rs2273500	0.106	0.127	0.405
Cigarettes per Day	Background diabetic retinopathy	ieu-b-25	finn-b-DM_BCKGRND_RETINA	rs2424888	0.104	0.126	0.407
Cigarettes per Day	Background diabetic retinopathy	ieu-b-25	finn-b-DM_BCKGRND_RETINA	rs3025383	0.135	0.126	0.285

Cigarettes per Day	Background diabetic retinopathy	ieu-b-25	finn-b-DM_BCKGRND_RETINA	rs34406232	0.137	0.126	0.278
Cigarettes per Day	Background diabetic retinopathy	ieu-b-25	finn-b-DM_BCKGRND_RETINA	rs4785587	0.088	0.125	0.481
Cigarettes per Day	Background diabetic retinopathy	ieu-b-25	finn-b-DM_BCKGRND_RETINA	rs56113850	0.135	0.136	0.323
Cigarettes per Day	Background diabetic retinopathy	ieu-b-25	finn-b-DM_BCKGRND_RETINA	rs58379124	0.126	0.128	0.322
Cigarettes per Day	Background diabetic retinopathy	ieu-b-25	finn-b-DM_BCKGRND_RETINA	rs632811	0.126	0.126	0.317
Cigarettes per Day	Background diabetic retinopathy	ieu-b-25	finn-b-DM_BCKGRND_RETINA	rs73229090	0.136	0.125	0.278
Cigarettes per Day	Background diabetic retinopathy	ieu-b-25	finn-b-DM_BCKGRND_RETINA	rs7431710	0.111	0.126	0.376
Cigarettes per Day	Background diabetic retinopathy	ieu-b-25	finn-b-DM_BCKGRND_RETINA	rs75494138	0.113	0.125	0.367

Cigarettes per Day	Background diabetic retinopathy	ieu-b-25	finn-b-DM_BCKGRND_RETINA	rs790564	0.131	0.126	0.297
Cigarettes per Day	Background diabetic retinopathy	ieu-b-25	finn-b-DM_BCKGRND_RETINA	rs7928017	0.112	0.125	0.372
Cigarettes per Day	Background diabetic retinopathy	ieu-b-25	finn-b-DM_BCKGRND_RETINA	rs7951365	0.136	0.126	0.278
Cigarettes per Day	Background diabetic retinopathy	ieu-b-25	finn-b-DM_BCKGRND_RETINA	rs8034191	0.026	0.165	0.875
Cigarettes per Day	Background diabetic retinopathy	ieu-b-25	finn-b-DM_BCKGRND_RETINA	rs806798	0.119	0.125	0.344
Cigarettes per Day	Background diabetic retinopathy	ieu-b-25	finn-b-DM_BCKGRND_RETINA	rs895330	0.105	0.125	0.403
Cigarettes per Day	Background diabetic retinopathy	ieu-b-25	finn-b-DM_BCKGRND_RETINA	All	0.116	0.125	0.351
Cigarettes per Day	Diabetic retinopathy	ieu-b-25	finn-b-DM_RETINOPATHY_EXMORE	rs11725618	0.157	0.062	0.011

Cigarettes per Day	Diabetic retinopathy	ieu-b-25	finn-b-DM_RETINOPATHY_EXMORE	rs1579233	0.154	0.062	0.014
Cigarettes per Day	Diabetic retinopathy	ieu-b-25	finn-b-DM_RETINOPATHY_EXMORE	rs2072659	0.158	0.062	0.010
Cigarettes per Day	Diabetic retinopathy	ieu-b-25	finn-b-DM_RETINOPATHY_EXMORE	rs2084533	0.145	0.062	0.019
Cigarettes per Day	Diabetic retinopathy	ieu-b-25	finn-b-DM_RETINOPATHY_EXMORE	rs215600	0.138	0.061	0.023
Cigarettes per Day	Diabetic retinopathy	ieu-b-25	finn-b-DM_RETINOPATHY_EXMORE	rs2273500	0.159	0.063	0.012
Cigarettes per Day	Diabetic retinopathy	ieu-b-25	finn-b-DM_RETINOPATHY_EXMORE	rs2424888	0.151	0.063	0.016
Cigarettes per Day	Diabetic retinopathy	ieu-b-25	finn-b-DM_RETINOPATHY_EXMORE	rs3025383	0.151	0.063	0.017
Cigarettes per Day	Diabetic retinopathy	ieu-b-25	finn-b-DM_RETINOPATHY_EXMORE	rs34406232	0.150	0.063	0.018

Cigarettes per Day	Diabetic retinopathy	ieu-b-25	finn-b-DM_RETINOPATHY_EXMORE	rs4785587	0.145	0.062	0.019
Cigarettes per Day	Diabetic retinopathy	ieu-b-25	finn-b-DM_RETINOPATHY_EXMORE	rs56113850	0.178	0.066	0.007
Cigarettes per Day	Diabetic retinopathy	ieu-b-25	finn-b-DM_RETINOPATHY_EXMORE	rs58379124	0.142	0.063	0.025
Cigarettes per Day	Diabetic retinopathy	ieu-b-25	finn-b-DM_RETINOPATHY_EXMORE	rs632811	0.158	0.062	0.010
Cigarettes per Day	Diabetic retinopathy	ieu-b-25	finn-b-DM_RETINOPATHY_EXMORE	rs73229090	0.168	0.054	0.002
Cigarettes per Day	Diabetic retinopathy	ieu-b-25	finn-b-DM_RETINOPATHY_EXMORE	rs7431710	0.149	0.063	0.017
Cigarettes per Day	Diabetic retinopathy	ieu-b-25	finn-b-DM_RETINOPATHY_EXMORE	rs75494138	0.149	0.062	0.017
Cigarettes per Day	Diabetic retinopathy	ieu-b-25	finn-b-DM_RETINOPATHY_EXMORE	rs790564	0.159	0.061	0.009

Cigarettes per Day	Diabetic retinopathy	ieu-b-25	finn-b-DM_RETINOPATHY_EXMORE	rs7928017	0.139	0.058	0.017
Cigarettes per Day	Diabetic retinopathy	ieu-b-25	finn-b-DM_RETINOPATHY_EXMORE	rs7951365	0.144	0.062	0.020
Cigarettes per Day	Diabetic retinopathy	ieu-b-25	finn-b-DM_RETINOPATHY_EXMORE	rs8034191	0.111	0.081	0.173
Cigarettes per Day	Diabetic retinopathy	ieu-b-25	finn-b-DM_RETINOPATHY_EXMORE	rs806798	0.156	0.062	0.012
Cigarettes per Day	Diabetic retinopathy	ieu-b-25	finn-b-DM_RETINOPATHY_EXMORE	rs895330	0.138	0.057	0.016
Cigarettes per Day	Diabetic retinopathy	ieu-b-25	finn-b-DM_RETINOPATHY_EXMORE	All	0.150	0.061	0.013
Cigarettes per Day	Proliferative diabetic retinopathy	ieu-b-25	finn-b-DM_RETINA_PROLIF	rs11725618	0.174	0.067	0.009
Cigarettes per Day	Proliferative diabetic retinopathy	ieu-b-25	finn-b-DM_RETINA_PROLIF	rs1579233	0.167	0.069	0.016

Cigarettes per Day	Proliferative diabetic retinopathy	ieu-b-25	finn-b-DM_RETINA_PROLIF	rs2072659	0.165	0.070	0.019
Cigarettes per Day	Proliferative diabetic retinopathy	ieu-b-25	finn-b-DM_RETINA_PROLIF	rs2084533	0.167	0.069	0.016
Cigarettes per Day	Proliferative diabetic retinopathy	ieu-b-25	finn-b-DM_RETINA_PROLIF	rs215600	0.159	0.070	0.023
Cigarettes per Day	Proliferative diabetic retinopathy	ieu-b-25	finn-b-DM_RETINA_PROLIF	rs2273500	0.168	0.071	0.018
Cigarettes per Day	Proliferative diabetic retinopathy	ieu-b-25	finn-b-DM_RETINA_PROLIF	rs2424888	0.158	0.069	0.022
Cigarettes per Day	Proliferative diabetic retinopathy	ieu-b-25	finn-b-DM_RETINA_PROLIF	rs3025383	0.168	0.070	0.016
Cigarettes per Day	Proliferative diabetic retinopathy	ieu-b-25	finn-b-DM_RETINA_PROLIF	rs34406232	0.157	0.070	0.025
Cigarettes per Day	Proliferative diabetic retinopathy	ieu-b-25	finn-b-DM_RETINA_PROLIF	rs4785587	0.147	0.063	0.020

Cigarettes per Day	Proliferative diabetic retinopathy	ieu-b-25	finn-b-DM_RETINA_PROLIF	rs56113850	0.185	0.075	0.014
Cigarettes per Day	Proliferative diabetic retinopathy	ieu-b-25	finn-b-DM_RETINA_PROLIF	rs58379124	0.145	0.068	0.034
Cigarettes per Day	Proliferative diabetic retinopathy	ieu-b-25	finn-b-DM_RETINA_PROLIF	rs632811	0.172	0.069	0.012
Cigarettes per Day	Proliferative diabetic retinopathy	ieu-b-25	finn-b-DM_RETINA_PROLIF	rs73229090	0.183	0.063	0.004
Cigarettes per Day	Proliferative diabetic retinopathy	ieu-b-25	finn-b-DM_RETINA_PROLIF	rs7431710	0.164	0.070	0.019
Cigarettes per Day	Proliferative diabetic retinopathy	ieu-b-25	finn-b-DM_RETINA_PROLIF	rs75494138	0.161	0.069	0.020
Cigarettes per Day	Proliferative diabetic retinopathy	ieu-b-25	finn-b-DM_RETINA_PROLIF	rs790564	0.177	0.066	0.007
Cigarettes per Day	Proliferative diabetic retinopathy	ieu-b-25	finn-b-DM_RETINA_PROLIF	rs7928017	0.163	0.070	0.020

Cigarettes per Day	Proliferative diabetic retinopathy	ieu-b-25	finn-b-DM_RETINA_PROLIF	rs7951365	0.158	0.069	0.022
Cigarettes per Day	Proliferative diabetic retinopathy	ieu-b-25	finn-b-DM_RETINA_PROLIF	rs8034191	0.139	0.092	0.128
Cigarettes per Day	Proliferative diabetic retinopathy	ieu-b-25	finn-b-DM_RETINA_PROLIF	rs806798	0.165	0.070	0.018
Cigarettes per Day	Proliferative diabetic retinopathy	ieu-b-25	finn-b-DM_RETINA_PROLIF	rs895330	0.150	0.064	0.019
Cigarettes per Day	Proliferative diabetic retinopathy	ieu-b-25	finn-b-DM_RETINA_PROLIF	All	0.164	0.068	0.015
Ever smoked	Background diabetic retinopathy	ukb-b-20261	finn-b-DM_BCKGRND_RETINA	rs10179482	0.207	0.640	0.747
Ever smoked	Background diabetic retinopathy	ukb-b-20261	finn-b-DM_BCKGRND_RETINA	rs10212155	0.201	0.645	0.756
Ever smoked	Background diabetic retinopathy	ukb-b-20261	finn-b-DM_BCKGRND_RETINA	rs10233018	0.077	0.637	0.904

Ever smoked	Background diabetic retinopathy	ukb-b-20261	finn-b-DM_BCKGRND_RETINA	rs10774625	-0.112	0.586	0.848
Ever smoked	Background diabetic retinopathy	ukb-b-20261	finn-b-DM_BCKGRND_RETINA	rs10863714	0.239	0.637	0.708
Ever smoked	Background diabetic retinopathy	ukb-b-20261	finn-b-DM_BCKGRND_RETINA	rs10952199	0.089	0.636	0.889
Ever smoked	Background diabetic retinopathy	ukb-b-20261	finn-b-DM_BCKGRND_RETINA	rs10956808	0.164	0.642	0.798
Ever smoked	Background diabetic retinopathy	ukb-b-20261	finn-b-DM_BCKGRND_RETINA	rs10988799	0.196	0.641	0.760
Ever smoked	Background diabetic retinopathy	ukb-b-20261	finn-b-DM_BCKGRND_RETINA	rs11165623	0.194	0.640	0.762
Ever smoked	Background diabetic retinopathy	ukb-b-20261	finn-b-DM_BCKGRND_RETINA	rs1124639	0.102	0.637	0.873
Ever smoked	Background diabetic retinopathy	ukb-b-20261	finn-b-DM_BCKGRND_RETINA	rs1150023	0.085	0.631	0.893

Ever smoked	Background diabetic retinopathy	ukb-b-20261	finn-b-DM_BCKGRND_RETINA	rs1174864	0.135	0.639	0.833
Ever smoked	Background diabetic retinopathy	ukb-b-20261	finn-b-DM_BCKGRND_RETINA	rs12209519	0.221	0.639	0.730
Ever smoked	Background diabetic retinopathy	ukb-b-20261	finn-b-DM_BCKGRND_RETINA	rs12244388	0.119	0.643	0.854
Ever smoked	Background diabetic retinopathy	ukb-b-20261	finn-b-DM_BCKGRND_RETINA	rs12272735	0.218	0.639	0.734
Ever smoked	Background diabetic retinopathy	ukb-b-20261	finn-b-DM_BCKGRND_RETINA	rs12333760	0.273	0.636	0.667
Ever smoked	Background diabetic retinopathy	ukb-b-20261	finn-b-DM_BCKGRND_RETINA	rs12450028	0.263	0.634	0.678
Ever smoked	Background diabetic retinopathy	ukb-b-20261	finn-b-DM_BCKGRND_RETINA	rs1246265	0.251	0.635	0.693
Ever smoked	Background diabetic retinopathy	ukb-b-20261	finn-b-DM_BCKGRND_RETINA	rs12902636	0.152	0.640	0.812

Ever smoked	Background diabetic retinopathy	ukb-b-20261	finn-b-DM_BCKGRND_RETINA	rs13162305	0.130	0.638	0.838
Ever smoked	Background diabetic retinopathy	ukb-b-20261	finn-b-DM_BCKGRND_RETINA	rs1322525	0.219	0.639	0.731
Ever smoked	Background diabetic retinopathy	ukb-b-20261	finn-b-DM_BCKGRND_RETINA	rs1324481	0.074	0.632	0.907
Ever smoked	Background diabetic retinopathy	ukb-b-20261	finn-b-DM_BCKGRND_RETINA	rs1363101	0.166	0.640	0.796
Ever smoked	Background diabetic retinopathy	ukb-b-20261	finn-b-DM_BCKGRND_RETINA	rs1373178	0.181	0.641	0.777
Ever smoked	Background diabetic retinopathy	ukb-b-20261	finn-b-DM_BCKGRND_RETINA	rs150294	0.116	0.641	0.857
Ever smoked	Background diabetic retinopathy	ukb-b-20261	finn-b-DM_BCKGRND_RETINA	rs1549212	0.137	0.640	0.830
Ever smoked	Background diabetic retinopathy	ukb-b-20261	finn-b-DM_BCKGRND_RETINA	rs1718705	0.220	0.639	0.730

Ever smoked	Background diabetic retinopathy	ukb-b-20261	finn-b-DM_BCKGRND_RETINA	rs17584022	0.145	0.640	0.820
Ever smoked	Background diabetic retinopathy	ukb-b-20261	finn-b-DM_BCKGRND_RETINA	rs1876066	0.178	0.640	0.781
Ever smoked	Background diabetic retinopathy	ukb-b-20261	finn-b-DM_BCKGRND_RETINA	rs1899896	0.202	0.641	0.752
Ever smoked	Background diabetic retinopathy	ukb-b-20261	finn-b-DM_BCKGRND_RETINA	rs2155292	0.406	0.650	0.533
Ever smoked	Background diabetic retinopathy	ukb-b-20261	finn-b-DM_BCKGRND_RETINA	rs2175207	0.154	0.641	0.809
Ever smoked	Background diabetic retinopathy	ukb-b-20261	finn-b-DM_BCKGRND_RETINA	rs2183573	0.087	0.630	0.890
Ever smoked	Background diabetic retinopathy	ukb-b-20261	finn-b-DM_BCKGRND_RETINA	rs28809490	0.154	0.639	0.809
Ever smoked	Background diabetic retinopathy	ukb-b-20261	finn-b-DM_BCKGRND_RETINA	rs303753	0.215	0.640	0.737

Ever smoked	Background diabetic retinopathy	ukb-b-20261	finn-b-DM_BCKGRND_RETINA	rs34335016	0.188	0.642	0.769
Ever smoked	Background diabetic retinopathy	ukb-b-20261	finn-b-DM_BCKGRND_RETINA	rs35498642	0.257	0.636	0.686
Ever smoked	Background diabetic retinopathy	ukb-b-20261	finn-b-DM_BCKGRND_RETINA	rs35892365	0.100	0.636	0.875
Ever smoked	Background diabetic retinopathy	ukb-b-20261	finn-b-DM_BCKGRND_RETINA	rs3783177	0.184	0.641	0.774
Ever smoked	Background diabetic retinopathy	ukb-b-20261	finn-b-DM_BCKGRND_RETINA	rs3790286	0.248	0.636	0.696
Ever smoked	Background diabetic retinopathy	ukb-b-20261	finn-b-DM_BCKGRND_RETINA	rs41513151	0.095	0.634	0.881
Ever smoked	Background diabetic retinopathy	ukb-b-20261	finn-b-DM_BCKGRND_RETINA	rs4422110	0.197	0.645	0.760
Ever smoked	Background diabetic retinopathy	ukb-b-20261	finn-b-DM_BCKGRND_RETINA	rs465646	0.223	0.646	0.730

Ever smoked	Background diabetic retinopathy	ukb-b-20261	finn-b-DM_BCKGRND_RETINA	rs4680392	0.136	0.639	0.831
Ever smoked	Background diabetic retinopathy	ukb-b-20261	finn-b-DM_BCKGRND_RETINA	rs4856598	0.053	0.634	0.933
Ever smoked	Background diabetic retinopathy	ukb-b-20261	finn-b-DM_BCKGRND_RETINA	rs528301	0.287	0.636	0.651
Ever smoked	Background diabetic retinopathy	ukb-b-20261	finn-b-DM_BCKGRND_RETINA	rs529206	0.201	0.640	0.754
Ever smoked	Background diabetic retinopathy	ukb-b-20261	finn-b-DM_BCKGRND_RETINA	rs55864295	0.174	0.641	0.785
Ever smoked	Background diabetic retinopathy	ukb-b-20261	finn-b-DM_BCKGRND_RETINA	rs56166763	0.238	0.637	0.709
Ever smoked	Background diabetic retinopathy	ukb-b-20261	finn-b-DM_BCKGRND_RETINA	rs58400863	0.231	0.639	0.718
Ever smoked	Background diabetic retinopathy	ukb-b-20261	finn-b-DM_BCKGRND_RETINA	rs61785503	0.215	0.640	0.736

Ever smoked	Background diabetic retinopathy	ukb-b-20261	finn-b-DM_BCKGRND_RETINA	rs6265	0.309	0.626	0.621
Ever smoked	Background diabetic retinopathy	ukb-b-20261	finn-b-DM_BCKGRND_RETINA	rs6438208	0.195	0.641	0.760
Ever smoked	Background diabetic retinopathy	ukb-b-20261	finn-b-DM_BCKGRND_RETINA	rs6499595	0.132	0.639	0.836
Ever smoked	Background diabetic retinopathy	ukb-b-20261	finn-b-DM_BCKGRND_RETINA	rs67716713	0.266	0.638	0.677
Ever smoked	Background diabetic retinopathy	ukb-b-20261	finn-b-DM_BCKGRND_RETINA	rs7014143	0.065	0.628	0.918
Ever smoked	Background diabetic retinopathy	ukb-b-20261	finn-b-DM_BCKGRND_RETINA	rs7024687	0.101	0.634	0.873
Ever smoked	Background diabetic retinopathy	ukb-b-20261	finn-b-DM_BCKGRND_RETINA	rs71580759	0.213	0.640	0.740
Ever smoked	Background diabetic retinopathy	ukb-b-20261	finn-b-DM_BCKGRND_RETINA	rs7162423	0.223	0.639	0.726

Ever smoked	Background diabetic retinopathy	ukb-b-20261	finn-b-DM_BCKGRND_RETINA	rs7216173	0.178	0.640	0.781
Ever smoked	Background diabetic retinopathy	ukb-b-20261	finn-b-DM_BCKGRND_RETINA	rs72706955	0.045	0.627	0.943
Ever smoked	Background diabetic retinopathy	ukb-b-20261	finn-b-DM_BCKGRND_RETINA	rs73058737	0.245	0.636	0.701
Ever smoked	Background diabetic retinopathy	ukb-b-20261	finn-b-DM_BCKGRND_RETINA	rs7572027	0.183	0.640	0.775
Ever smoked	Background diabetic retinopathy	ukb-b-20261	finn-b-DM_BCKGRND_RETINA	rs75919030	0.224	0.639	0.725
Ever smoked	Background diabetic retinopathy	ukb-b-20261	finn-b-DM_BCKGRND_RETINA	rs763053	0.192	0.644	0.766
Ever smoked	Background diabetic retinopathy	ukb-b-20261	finn-b-DM_BCKGRND_RETINA	rs7758291	0.146	0.639	0.819
Ever smoked	Background diabetic retinopathy	ukb-b-20261	finn-b-DM_BCKGRND_RETINA	rs77878475	0.134	0.640	0.835

Ever smoked	Background diabetic retinopathy	ukb-b-20261	finn-b-DM_BCKGRND_RETINA	rs7870475	0.217	0.639	0.735
Ever smoked	Background diabetic retinopathy	ukb-b-20261	finn-b-DM_BCKGRND_RETINA	rs7901348	0.202	0.641	0.753
Ever smoked	Background diabetic retinopathy	ukb-b-20261	finn-b-DM_BCKGRND_RETINA	rs7969559	0.198	0.641	0.758
Ever smoked	Background diabetic retinopathy	ukb-b-20261	finn-b-DM_BCKGRND_RETINA	rs899632	0.211	0.641	0.742
Ever smoked	Background diabetic retinopathy	ukb-b-20261	finn-b-DM_BCKGRND_RETINA	rs904592	0.221	0.639	0.729
Ever smoked	Background diabetic retinopathy	ukb-b-20261	finn-b-DM_BCKGRND_RETINA	rs905871	0.164	0.640	0.798
Ever smoked	Background diabetic retinopathy	ukb-b-20261	finn-b-DM_BCKGRND_RETINA	rs9375371	0.149	0.641	0.816
Ever smoked	Background diabetic retinopathy	ukb-b-20261	finn-b-DM_BCKGRND_RETINA	rs9423279	0.254	0.636	0.690

Ever smoked	Background diabetic retinopathy	ukb-b-20261	finn-b-DM_BCKGRND_RETINA	rs9597810	0.263	0.633	0.678
Ever smoked	Background diabetic retinopathy	ukb-b-20261	finn-b-DM_BCKGRND_RETINA	rs9845144	0.321	0.613	0.600
Ever smoked	Background diabetic retinopathy	ukb-b-20261	finn-b-DM_BCKGRND_RETINA	All	0.181	0.633	0.775
Ever smoked	Diabetic retinopathy	ukb-b-20261	finn-b-DM_RETINOPATHY_EXMORE	rs10179482	-0.273	0.262	0.299
Ever smoked	Diabetic retinopathy	ukb-b-20261	finn-b-DM_RETINOPATHY_EXMORE	rs10212155	-0.231	0.264	0.381
Ever smoked	Diabetic retinopathy	ukb-b-20261	finn-b-DM_RETINOPATHY_EXMORE	rs10233018	-0.239	0.263	0.364
Ever smoked	Diabetic retinopathy	ukb-b-20261	finn-b-DM_RETINOPATHY_EXMORE	rs10774625	-0.362	0.243	0.136
Ever smoked	Diabetic retinopathy	ukb-b-20261	finn-b-DM_RETINOPATHY_EXMORE	rs10863714	-0.263	0.263	0.317

Ever smoked	Diabetic retinopathy	ukb-b-20261	finn-b-DM_RETINOPATHY_EXMORE	rs10952199	-0.322	0.257	0.210
Ever smoked	Diabetic retinopathy	ukb-b-20261	finn-b-DM_RETINOPATHY_EXMORE	rs10956808	-0.269	0.263	0.308
Ever smoked	Diabetic retinopathy	ukb-b-20261	finn-b-DM_RETINOPATHY_EXMORE	rs10988799	-0.240	0.262	0.360
Ever smoked	Diabetic retinopathy	ukb-b-20261	finn-b-DM_RETINOPATHY_EXMORE	rs11165623	-0.255	0.263	0.332
Ever smoked	Diabetic retinopathy	ukb-b-20261	finn-b-DM_RETINOPATHY_EXMORE	rs1124639	-0.306	0.259	0.238
Ever smoked	Diabetic retinopathy	ukb-b-20261	finn-b-DM_RETINOPATHY_EXMORE	rs1150023	-0.301	0.258	0.243
Ever smoked	Diabetic retinopathy	ukb-b-20261	finn-b-DM_RETINOPATHY_EXMORE	rs1174864	-0.256	0.263	0.330
Ever smoked	Diabetic retinopathy	ukb-b-20261	finn-b-DM_RETINOPATHY_EXMORE	rs12209519	-0.260	0.263	0.322

Ever smoked	Diabetic retinopathy	ukb-b-20261	finn-b-DM_RETINOPATHY_EXMORE	rs12244388	-0.274	0.264	0.299
Ever smoked	Diabetic retinopathy	ukb-b-20261	finn-b-DM_RETINOPATHY_EXMORE	rs12272735	-0.278	0.262	0.289
Ever smoked	Diabetic retinopathy	ukb-b-20261	finn-b-DM_RETINOPATHY_EXMORE	rs12333760	-0.175	0.252	0.486
Ever smoked	Diabetic retinopathy	ukb-b-20261	finn-b-DM_RETINOPATHY_EXMORE	rs12450028	-0.223	0.260	0.391
Ever smoked	Diabetic retinopathy	ukb-b-20261	finn-b-DM_RETINOPATHY_EXMORE	rs1246265	-0.236	0.261	0.366
Ever smoked	Diabetic retinopathy	ukb-b-20261	finn-b-DM_RETINOPATHY_EXMORE	rs12902636	-0.278	0.262	0.290
Ever smoked	Diabetic retinopathy	ukb-b-20261	finn-b-DM_RETINOPATHY_EXMORE	rs13162305	-0.228	0.260	0.382
Ever smoked	Diabetic retinopathy	ukb-b-20261	finn-b-DM_RETINOPATHY_EXMORE	rs1322525	-0.245	0.262	0.351

Ever smoked	Diabetic retinopathy	ukb-b-20261	finn-b-DM_RETINOPATHY_EXMORE	rs1324481	-0.306	0.259	0.238
Ever smoked	Diabetic retinopathy	ukb-b-20261	finn-b-DM_RETINOPATHY_EXMORE	rs1363101	-0.275	0.262	0.294
Ever smoked	Diabetic retinopathy	ukb-b-20261	finn-b-DM_RETINOPATHY_EXMORE	rs1373178	-0.277	0.262	0.291
Ever smoked	Diabetic retinopathy	ukb-b-20261	finn-b-DM_RETINOPATHY_EXMORE	rs150294	-0.253	0.264	0.338
Ever smoked	Diabetic retinopathy	ukb-b-20261	finn-b-DM_RETINOPATHY_EXMORE	rs1549212	-0.248	0.263	0.345
Ever smoked	Diabetic retinopathy	ukb-b-20261	finn-b-DM_RETINOPATHY_EXMORE	rs1718705	-0.209	0.257	0.416
Ever smoked	Diabetic retinopathy	ukb-b-20261	finn-b-DM_RETINOPATHY_EXMORE	rs17584022	-0.232	0.261	0.375
Ever smoked	Diabetic retinopathy	ukb-b-20261	finn-b-DM_RETINOPATHY_EXMORE	rs1876066	-0.281	0.262	0.283

Ever smoked	Diabetic retinopathy	ukb-b-20261	finn-b-DM_RETINOPATHY_EXMORE	rs1899896	-0.293	0.261	0.261
Ever smoked	Diabetic retinopathy	ukb-b-20261	finn-b-DM_RETINOPATHY_EXMORE	rs2155292	-0.193	0.268	0.473
Ever smoked	Diabetic retinopathy	ukb-b-20261	finn-b-DM_RETINOPATHY_EXMORE	rs2175207	-0.255	0.263	0.332
Ever smoked	Diabetic retinopathy	ukb-b-20261	finn-b-DM_RETINOPATHY_EXMORE	rs2183573	-0.274	0.262	0.296
Ever smoked	Diabetic retinopathy	ukb-b-20261	finn-b-DM_RETINOPATHY_EXMORE	rs28809490	-0.265	0.263	0.314
Ever smoked	Diabetic retinopathy	ukb-b-20261	finn-b-DM_RETINOPATHY_EXMORE	rs303753	-0.256	0.263	0.331
Ever smoked	Diabetic retinopathy	ukb-b-20261	finn-b-DM_RETINOPATHY_EXMORE	rs34335016	-0.231	0.262	0.377
Ever smoked	Diabetic retinopathy	ukb-b-20261	finn-b-DM_RETINOPATHY_EXMORE	rs35498642	-0.261	0.263	0.320

Ever smoked	Diabetic retinopathy	ukb-b-20261	finn-b-DM_RETINOPATHY_EXMORE	rs35892365	-0.261	0.263	0.322
Ever smoked	Diabetic retinopathy	ukb-b-20261	finn-b-DM_RETINOPATHY_EXMORE	rs3783177	-0.250	0.263	0.341
Ever smoked	Diabetic retinopathy	ukb-b-20261	finn-b-DM_RETINOPATHY_EXMORE	rs3790286	-0.260	0.263	0.322
Ever smoked	Diabetic retinopathy	ukb-b-20261	finn-b-DM_RETINOPATHY_EXMORE	rs41513151	-0.277	0.262	0.291
Ever smoked	Diabetic retinopathy	ukb-b-20261	finn-b-DM_RETINOPATHY_EXMORE	rs4422110	-0.268	0.265	0.312
Ever smoked	Diabetic retinopathy	ukb-b-20261	finn-b-DM_RETINOPATHY_EXMORE	rs465646	-0.238	0.265	0.368
Ever smoked	Diabetic retinopathy	ukb-b-20261	finn-b-DM_RETINOPATHY_EXMORE	rs4680392	-0.265	0.263	0.314
Ever smoked	Diabetic retinopathy	ukb-b-20261	finn-b-DM_RETINOPATHY_EXMORE	rs4856598	-0.268	0.264	0.309

Ever smoked	Diabetic retinopathy	ukb-b-20261	finn-b-DM_RETINOPATHY_EXMORE	rs528301	-0.225	0.262	0.390
Ever smoked	Diabetic retinopathy	ukb-b-20261	finn-b-DM_RETINOPATHY_EXMORE	rs529206	-0.303	0.258	0.241
Ever smoked	Diabetic retinopathy	ukb-b-20261	finn-b-DM_RETINOPATHY_EXMORE	rs55864295	-0.271	0.263	0.301
Ever smoked	Diabetic retinopathy	ukb-b-20261	finn-b-DM_RETINOPATHY_EXMORE	rs56166763	-0.287	0.261	0.270
Ever smoked	Diabetic retinopathy	ukb-b-20261	finn-b-DM_RETINOPATHY_EXMORE	rs58400863	-0.220	0.260	0.397
Ever smoked	Diabetic retinopathy	ukb-b-20261	finn-b-DM_RETINOPATHY_EXMORE	rs61785503	-0.250	0.263	0.342
Ever smoked	Diabetic retinopathy	ukb-b-20261	finn-b-DM_RETINOPATHY_EXMORE	rs6265	-0.259	0.263	0.325
Ever smoked	Diabetic retinopathy	ukb-b-20261	finn-b-DM_RETINOPATHY_EXMORE	rs6438208	-0.243	0.262	0.355

Ever smoked	Diabetic retinopathy	ukb-b-20261	finn-b-DM_RETINOPATHY_EXMORE	rs6499595	-0.249	0.263	0.344
Ever smoked	Diabetic retinopathy	ukb-b-20261	finn-b-DM_RETINOPATHY_EXMORE	rs67716713	-0.220	0.261	0.401
Ever smoked	Diabetic retinopathy	ukb-b-20261	finn-b-DM_RETINOPATHY_EXMORE	rs7014143	-0.292	0.260	0.262
Ever smoked	Diabetic retinopathy	ukb-b-20261	finn-b-DM_RETINOPATHY_EXMORE	rs7024687	-0.240	0.262	0.359
Ever smoked	Diabetic retinopathy	ukb-b-20261	finn-b-DM_RETINOPATHY_EXMORE	rs71580759	-0.229	0.261	0.379
Ever smoked	Diabetic retinopathy	ukb-b-20261	finn-b-DM_RETINOPATHY_EXMORE	rs7162423	-0.273	0.262	0.298
Ever smoked	Diabetic retinopathy	ukb-b-20261	finn-b-DM_RETINOPATHY_EXMORE	rs7216173	-0.303	0.258	0.239
Ever smoked	Diabetic retinopathy	ukb-b-20261	finn-b-DM_RETINOPATHY_EXMORE	rs72706955	-0.303	0.260	0.243

Ever smoked	Diabetic retinopathy	ukb-b-20261	finn-b-DM_RETINOPATHY_EXMORE	rs73058737	-0.298	0.259	0.250
Ever smoked	Diabetic retinopathy	ukb-b-20261	finn-b-DM_RETINOPATHY_EXMORE	rs7572027	-0.257	0.263	0.327
Ever smoked	Diabetic retinopathy	ukb-b-20261	finn-b-DM_RETINOPATHY_EXMORE	rs75919030	-0.224	0.260	0.388
Ever smoked	Diabetic retinopathy	ukb-b-20261	finn-b-DM_RETINOPATHY_EXMORE	rs763053	-0.256	0.264	0.333
Ever smoked	Diabetic retinopathy	ukb-b-20261	finn-b-DM_RETINOPATHY_EXMORE	rs7758291	-0.266	0.263	0.310
Ever smoked	Diabetic retinopathy	ukb-b-20261	finn-b-DM_RETINOPATHY_EXMORE	rs77878475	-0.303	0.260	0.244
Ever smoked	Diabetic retinopathy	ukb-b-20261	finn-b-DM_RETINOPATHY_EXMORE	rs7870475	-0.223	0.259	0.391
Ever smoked	Diabetic retinopathy	ukb-b-20261	finn-b-DM_RETINOPATHY_EXMORE	rs7901348	-0.239	0.262	0.363

Ever smoked	Diabetic retinopathy	ukb-b-20261	finn-b-DM_RETINOPATHY_EXMORE	rs7969559	-0.282	0.262	0.282
Ever smoked	Diabetic retinopathy	ukb-b-20261	finn-b-DM_RETINOPATHY_EXMORE	rs899632	-0.259	0.263	0.325
Ever smoked	Diabetic retinopathy	ukb-b-20261	finn-b-DM_RETINOPATHY_EXMORE	rs904592	-0.238	0.262	0.363
Ever smoked	Diabetic retinopathy	ukb-b-20261	finn-b-DM_RETINOPATHY_EXMORE	rs905871	-0.238	0.262	0.362
Ever smoked	Diabetic retinopathy	ukb-b-20261	finn-b-DM_RETINOPATHY_EXMORE	rs9375371	-0.257	0.263	0.330
Ever smoked	Diabetic retinopathy	ukb-b-20261	finn-b-DM_RETINOPATHY_EXMORE	rs9423279	-0.249	0.263	0.343
Ever smoked	Diabetic retinopathy	ukb-b-20261	finn-b-DM_RETINOPATHY_EXMORE	rs9597810	-0.233	0.261	0.371
Ever smoked	Diabetic retinopathy	ukb-b-20261	finn-b-DM_RETINOPATHY_EXMORE	rs9845144	-0.276	0.262	0.292

Ever smoked	Diabetic retinopathy	ukb-b-20261	finn-b-DM_RETINOPATHY_EXMORE	All	-0.259	0.260	0.318
Ever smoked	Proliferative diabetic retinopathy	ukb-b-20261	finn-b-DM_RETINA_PROLIF	rs10179482	-0.378	0.334	0.257
Ever smoked	Proliferative diabetic retinopathy	ukb-b-20261	finn-b-DM_RETINA_PROLIF	rs10212155	-0.371	0.336	0.270
Ever smoked	Proliferative diabetic retinopathy	ukb-b-20261	finn-b-DM_RETINA_PROLIF	rs10233018	-0.380	0.336	0.258
Ever smoked	Proliferative diabetic retinopathy	ukb-b-20261	finn-b-DM_RETINA_PROLIF	rs10774625	-0.524	0.307	0.088
Ever smoked	Proliferative diabetic retinopathy	ukb-b-20261	finn-b-DM_RETINA_PROLIF	rs10863714	-0.349	0.331	0.292
Ever smoked	Proliferative diabetic retinopathy	ukb-b-20261	finn-b-DM_RETINA_PROLIF	rs10952199	-0.491	0.321	0.125
Ever smoked	Proliferative diabetic retinopathy	ukb-b-20261	finn-b-DM_RETINA_PROLIF	rs10956808	-0.389	0.335	0.245

Ever smoked	Proliferative diabetic retinopathy	ukb-b-20261	finn-b-DM_RETINA_PROLIF	rs10988799	-0.388	0.335	0.246
Ever smoked	Proliferative diabetic retinopathy	ukb-b-20261	finn-b-DM_RETINA_PROLIF	rs11165623	-0.386	0.334	0.248
Ever smoked	Proliferative diabetic retinopathy	ukb-b-20261	finn-b-DM_RETINA_PROLIF	rs1124639	-0.422	0.333	0.206
Ever smoked	Proliferative diabetic retinopathy	ukb-b-20261	finn-b-DM_RETINA_PROLIF	rs1150023	-0.443	0.328	0.177
Ever smoked	Proliferative diabetic retinopathy	ukb-b-20261	finn-b-DM_RETINA_PROLIF	rs1174864	-0.357	0.333	0.283
Ever smoked	Proliferative diabetic retinopathy	ukb-b-20261	finn-b-DM_RETINA_PROLIF	rs12209519	-0.400	0.334	0.231
Ever smoked	Proliferative diabetic retinopathy	ukb-b-20261	finn-b-DM_RETINA_PROLIF	rs12244388	-0.391	0.336	0.246
Ever smoked	Proliferative diabetic retinopathy	ukb-b-20261	finn-b-DM_RETINA_PROLIF	rs12272735	-0.409	0.333	0.220

Ever smoked	Proliferative diabetic retinopathy	ukb-b-20261	finn-b-DM_RETINA_PROLIF	rs12333760	-0.300	0.325	0.356
Ever smoked	Proliferative diabetic retinopathy	ukb-b-20261	finn-b-DM_RETINA_PROLIF	rs12450028	-0.358	0.333	0.281
Ever smoked	Proliferative diabetic retinopathy	ukb-b-20261	finn-b-DM_RETINA_PROLIF	rs1246265	-0.368	0.333	0.270
Ever smoked	Proliferative diabetic retinopathy	ukb-b-20261	finn-b-DM_RETINA_PROLIF	rs12902636	-0.404	0.334	0.226
Ever smoked	Proliferative diabetic retinopathy	ukb-b-20261	finn-b-DM_RETINA_PROLIF	rs13162305	-0.369	0.334	0.269
Ever smoked	Proliferative diabetic retinopathy	ukb-b-20261	finn-b-DM_RETINA_PROLIF	rs1322525	-0.382	0.334	0.254
Ever smoked	Proliferative diabetic retinopathy	ukb-b-20261	finn-b-DM_RETINA_PROLIF	rs1324481	-0.435	0.331	0.190
Ever smoked	Proliferative diabetic retinopathy	ukb-b-20261	finn-b-DM_RETINA_PROLIF	rs1363101	-0.447	0.328	0.173

Ever smoked	Proliferative diabetic retinopathy	ukb-b-20261	finn-b-DM_RETINA_PROLIF	rs1373178	-0.415	0.333	0.214
Ever smoked	Proliferative diabetic retinopathy	ukb-b-20261	finn-b-DM_RETINA_PROLIF	rs150294	-0.372	0.335	0.267
Ever smoked	Proliferative diabetic retinopathy	ukb-b-20261	finn-b-DM_RETINA_PROLIF	rs1549212	-0.366	0.334	0.274
Ever smoked	Proliferative diabetic retinopathy	ukb-b-20261	finn-b-DM_RETINA_PROLIF	rs1718705	-0.350	0.332	0.292
Ever smoked	Proliferative diabetic retinopathy	ukb-b-20261	finn-b-DM_RETINA_PROLIF	rs17584022	-0.370	0.334	0.267
Ever smoked	Proliferative diabetic retinopathy	ukb-b-20261	finn-b-DM_RETINA_PROLIF	rs1876066	-0.423	0.332	0.202
Ever smoked	Proliferative diabetic retinopathy	ukb-b-20261	finn-b-DM_RETINA_PROLIF	rs1899896	-0.428	0.332	0.198
Ever smoked	Proliferative diabetic retinopathy	ukb-b-20261	finn-b-DM_RETINA_PROLIF	rs2155292	-0.316	0.342	0.356

Ever smoked	Proliferative diabetic retinopathy	ukb-b-20261	finn-b-DM_RETINA_PROLIF	rs2175207	-0.353	0.332	0.289
Ever smoked	Proliferative diabetic retinopathy	ukb-b-20261	finn-b-DM_RETINA_PROLIF	rs2183573	-0.390	0.334	0.244
Ever smoked	Proliferative diabetic retinopathy	ukb-b-20261	finn-b-DM_RETINA_PROLIF	rs28809490	-0.407	0.333	0.223
Ever smoked	Proliferative diabetic retinopathy	ukb-b-20261	finn-b-DM_RETINA_PROLIF	rs303753	-0.388	0.334	0.245
Ever smoked	Proliferative diabetic retinopathy	ukb-b-20261	finn-b-DM_RETINA_PROLIF	rs34335016	-0.369	0.334	0.269
Ever smoked	Proliferative diabetic retinopathy	ukb-b-20261	finn-b-DM_RETINA_PROLIF	rs35498642	-0.374	0.334	0.263
Ever smoked	Proliferative diabetic retinopathy	ukb-b-20261	finn-b-DM_RETINA_PROLIF	rs35892365	-0.404	0.335	0.227
Ever smoked	Proliferative diabetic retinopathy	ukb-b-20261	finn-b-DM_RETINA_PROLIF	rs3783177	-0.320	0.326	0.327

Ever smoked	Proliferative diabetic retinopathy	ukb-b-20261	finn-b-DM_RETINA_PROLIF	rs3790286	-0.392	0.334	0.240
Ever smoked	Proliferative diabetic retinopathy	ukb-b-20261	finn-b-DM_RETINA_PROLIF	rs41513151	-0.436	0.330	0.187
Ever smoked	Proliferative diabetic retinopathy	ukb-b-20261	finn-b-DM_RETINA_PROLIF	rs4422110	-0.348	0.335	0.299
Ever smoked	Proliferative diabetic retinopathy	ukb-b-20261	finn-b-DM_RETINA_PROLIF	rs465646	-0.393	0.337	0.244
Ever smoked	Proliferative diabetic retinopathy	ukb-b-20261	finn-b-DM_RETINA_PROLIF	rs4680392	-0.394	0.334	0.238
Ever smoked	Proliferative diabetic retinopathy	ukb-b-20261	finn-b-DM_RETINA_PROLIF	rs4856598	-0.394	0.336	0.241
Ever smoked	Proliferative diabetic retinopathy	ukb-b-20261	finn-b-DM_RETINA_PROLIF	rs528301	-0.373	0.335	0.265
Ever smoked	Proliferative diabetic retinopathy	ukb-b-20261	finn-b-DM_RETINA_PROLIF	rs529206	-0.442	0.329	0.179

Ever smoked	Proliferative diabetic retinopathy	ukb-b-20261	finn-b-DM_RETINA_PROLIF	rs55864295	-0.372	0.334	0.266
Ever smoked	Proliferative diabetic retinopathy	ukb-b-20261	finn-b-DM_RETINA_PROLIF	rs56166763	-0.411	0.333	0.217
Ever smoked	Proliferative diabetic retinopathy	ukb-b-20261	finn-b-DM_RETINA_PROLIF	rs58400863	-0.328	0.328	0.317
Ever smoked	Proliferative diabetic retinopathy	ukb-b-20261	finn-b-DM_RETINA_PROLIF	rs61785503	-0.343	0.331	0.299
Ever smoked	Proliferative diabetic retinopathy	ukb-b-20261	finn-b-DM_RETINA_PROLIF	rs6265	-0.351	0.332	0.291
Ever smoked	Proliferative diabetic retinopathy	ukb-b-20261	finn-b-DM_RETINA_PROLIF	rs6438208	-0.378	0.334	0.258
Ever smoked	Proliferative diabetic retinopathy	ukb-b-20261	finn-b-DM_RETINA_PROLIF	rs6499595	-0.410	0.334	0.220
Ever smoked	Proliferative diabetic retinopathy	ukb-b-20261	finn-b-DM_RETINA_PROLIF	rs67716713	-0.370	0.335	0.269

Ever smoked	Proliferative diabetic retinopathy	ukb-b-20261	finn-b-DM_RETINA_PROLIF	rs7014143	-0.420	0.333	0.207
Ever smoked	Proliferative diabetic retinopathy	ukb-b-20261	finn-b-DM_RETINA_PROLIF	rs7024687	-0.372	0.334	0.265
Ever smoked	Proliferative diabetic retinopathy	ukb-b-20261	finn-b-DM_RETINA_PROLIF	rs71580759	-0.368	0.334	0.269
Ever smoked	Proliferative diabetic retinopathy	ukb-b-20261	finn-b-DM_RETINA_PROLIF	rs7162423	-0.396	0.334	0.236
Ever smoked	Proliferative diabetic retinopathy	ukb-b-20261	finn-b-DM_RETINA_PROLIF	rs7216173	-0.441	0.328	0.179
Ever smoked	Proliferative diabetic retinopathy	ukb-b-20261	finn-b-DM_RETINA_PROLIF	rs72706955	-0.441	0.331	0.183
Ever smoked	Proliferative diabetic retinopathy	ukb-b-20261	finn-b-DM_RETINA_PROLIF	rs73058737	-0.406	0.334	0.224
Ever smoked	Proliferative diabetic retinopathy	ukb-b-20261	finn-b-DM_RETINA_PROLIF	rs7572027	-0.403	0.334	0.227

Ever smoked	Proliferative diabetic retinopathy	ukb-b-20261	finn-b-DM_RETINA_PROLIF	rs75919030	-0.318	0.326	0.328
Ever smoked	Proliferative diabetic retinopathy	ukb-b-20261	finn-b-DM_RETINA_PROLIF	rs763053	-0.369	0.336	0.272
Ever smoked	Proliferative diabetic retinopathy	ukb-b-20261	finn-b-DM_RETINA_PROLIF	rs7758291	-0.414	0.333	0.214
Ever smoked	Proliferative diabetic retinopathy	ukb-b-20261	finn-b-DM_RETINA_PROLIF	rs77878475	-0.456	0.329	0.165
Ever smoked	Proliferative diabetic retinopathy	ukb-b-20261	finn-b-DM_RETINA_PROLIF	rs7870475	-0.369	0.334	0.269
Ever smoked	Proliferative diabetic retinopathy	ukb-b-20261	finn-b-DM_RETINA_PROLIF	rs7901348	-0.367	0.334	0.272
Ever smoked	Proliferative diabetic retinopathy	ukb-b-20261	finn-b-DM_RETINA_PROLIF	rs7969559	-0.428	0.332	0.198
Ever smoked	Proliferative diabetic retinopathy	ukb-b-20261	finn-b-DM_RETINA_PROLIF	rs899632	-0.388	0.335	0.247

Ever smoked	Proliferative diabetic retinopathy	ukb-b-20261	finn-b-DM_RETINA_PROLIF	rs904592	-0.363	0.333	0.276
Ever smoked	Proliferative diabetic retinopathy	ukb-b-20261	finn-b-DM_RETINA_PROLIF	rs905871	-0.376	0.334	0.260
Ever smoked	Proliferative diabetic retinopathy	ukb-b-20261	finn-b-DM_RETINA_PROLIF	rs9375371	-0.389	0.335	0.246
Ever smoked	Proliferative diabetic retinopathy	ukb-b-20261	finn-b-DM_RETINA_PROLIF	rs9423279	-0.372	0.334	0.265
Ever smoked	Proliferative diabetic retinopathy	ukb-b-20261	finn-b-DM_RETINA_PROLIF	rs9597810	-0.365	0.333	0.273
Ever smoked	Proliferative diabetic retinopathy	ukb-b-20261	finn-b-DM_RETINA_PROLIF	rs9845144	-0.397	0.334	0.234
Ever smoked	Proliferative diabetic retinopathy	ukb-b-20261	finn-b-DM_RETINA_PROLIF	All	-0.389	0.330	0.239
Hip circumference	Background diabetic retinopathy	ieu-a-55	finn-b-DM_BCKGRND_RETINA	rs10123368	-0.321	0.143	0.025

Hip circumference	Background diabetic retinopathy	ieu-a-55	finn-b-DM_BCKGRND_RETINA	rs10140922	-0.360	0.144	0.012
Hip circumference	Background diabetic retinopathy	ieu-a-55	finn-b-DM_BCKGRND_RETINA	rs10195252	-0.323	0.144	0.025
Hip circumference	Background diabetic retinopathy	ieu-a-55	finn-b-DM_BCKGRND_RETINA	rs1046934	-0.349	0.144	0.015
Hip circumference	Background diabetic retinopathy	ieu-a-55	finn-b-DM_BCKGRND_RETINA	rs1053593	-0.324	0.143	0.024
Hip circumference	Background diabetic retinopathy	ieu-a-55	finn-b-DM_BCKGRND_RETINA	rs10748128	-0.332	0.144	0.021
Hip circumference	Background diabetic retinopathy	ieu-a-55	finn-b-DM_BCKGRND_RETINA	rs10804591	-0.323	0.144	0.025
Hip circumference	Background diabetic retinopathy	ieu-a-55	finn-b-DM_BCKGRND_RETINA	rs10950949	-0.316	0.143	0.027
Hip circumference	Background diabetic retinopathy	ieu-a-55	finn-b-DM_BCKGRND_RETINA	rs10958476	-0.344	0.144	0.017

Hip circumference	Background diabetic retinopathy	ieu-a-55	finn-b-DM_BCKGRND_RETINA	rs11205303	-0.319	0.145	0.028
Hip circumference	Background diabetic retinopathy	ieu-a-55	finn-b-DM_BCKGRND_RETINA	rs11242	-0.319	0.144	0.027
Hip circumference	Background diabetic retinopathy	ieu-a-55	finn-b-DM_BCKGRND_RETINA	rs11612228	-0.337	0.143	0.019
Hip circumference	Background diabetic retinopathy	ieu-a-55	finn-b-DM_BCKGRND_RETINA	rs1173771	-0.301	0.144	0.037
Hip circumference	Background diabetic retinopathy	ieu-a-55	finn-b-DM_BCKGRND_RETINA	rs12086130	-0.333	0.143	0.020
Hip circumference	Background diabetic retinopathy	ieu-a-55	finn-b-DM_BCKGRND_RETINA	rs12207675	-0.298	0.144	0.038
Hip circumference	Background diabetic retinopathy	ieu-a-55	finn-b-DM_BCKGRND_RETINA	rs1254257	-0.342	0.144	0.017
Hip circumference	Background diabetic retinopathy	ieu-a-55	finn-b-DM_BCKGRND_RETINA	rs12817549	-0.318	0.144	0.027

Hip circumference	Background diabetic retinopathy	ieu-a-55	finn-b-DM_BCKGRND_RETINA	rs1294410	-0.339	0.144	0.019
Hip circumference	Background diabetic retinopathy	ieu-a-55	finn-b-DM_BCKGRND_RETINA	rs12980348	-0.316	0.144	0.028
Hip circumference	Background diabetic retinopathy	ieu-a-55	finn-b-DM_BCKGRND_RETINA	rs1351394	-0.328	0.144	0.022
Hip circumference	Background diabetic retinopathy	ieu-a-55	finn-b-DM_BCKGRND_RETINA	rs1388251	-0.309	0.143	0.031
Hip circumference	Background diabetic retinopathy	ieu-a-55	finn-b-DM_BCKGRND_RETINA	rs143384	-0.362	0.145	0.013
Hip circumference	Background diabetic retinopathy	ieu-a-55	finn-b-DM_BCKGRND_RETINA	rs1545552	-0.323	0.144	0.025
Hip circumference	Background diabetic retinopathy	ieu-a-55	finn-b-DM_BCKGRND_RETINA	rs1662837	-0.333	0.144	0.021
Hip circumference	Background diabetic retinopathy	ieu-a-55	finn-b-DM_BCKGRND_RETINA	rs16894959	-0.331	0.144	0.022

Hip circumference	Background diabetic retinopathy	ieu-a-55	finn-b-DM_BCKGRND_RETINA	rs169797	-0.306	0.144	0.033
Hip circumference	Background diabetic retinopathy	ieu-a-55	finn-b-DM_BCKGRND_RETINA	rs17193922	-0.320	0.143	0.026
Hip circumference	Background diabetic retinopathy	ieu-a-55	finn-b-DM_BCKGRND_RETINA	rs1727294	-0.326	0.144	0.023
Hip circumference	Background diabetic retinopathy	ieu-a-55	finn-b-DM_BCKGRND_RETINA	rs17346473	-0.321	0.144	0.026
Hip circumference	Background diabetic retinopathy	ieu-a-55	finn-b-DM_BCKGRND_RETINA	rs17819328	-0.304	0.144	0.034
Hip circumference	Background diabetic retinopathy	ieu-a-55	finn-b-DM_BCKGRND_RETINA	rs1812175	-0.296	0.146	0.042
Hip circumference	Background diabetic retinopathy	ieu-a-55	finn-b-DM_BCKGRND_RETINA	rs181553	-0.317	0.144	0.028
Hip circumference	Background diabetic retinopathy	ieu-a-55	finn-b-DM_BCKGRND_RETINA	rs2034088	-0.314	0.143	0.029

Hip circumference	Background diabetic retinopathy	ieu-a-55	finn-b-DM_BCKGRND_RETINA	rs2098771	-0.342	0.143	0.017
Hip circumference	Background diabetic retinopathy	ieu-a-55	finn-b-DM_BCKGRND_RETINA	rs2247341	-0.329	0.144	0.022
Hip circumference	Background diabetic retinopathy	ieu-a-55	finn-b-DM_BCKGRND_RETINA	rs2326788	-0.320	0.143	0.026
Hip circumference	Background diabetic retinopathy	ieu-a-55	finn-b-DM_BCKGRND_RETINA	rs2377058	-0.314	0.143	0.028
Hip circumference	Background diabetic retinopathy	ieu-a-55	finn-b-DM_BCKGRND_RETINA	rs2597513	-0.331	0.143	0.021
Hip circumference	Background diabetic retinopathy	ieu-a-55	finn-b-DM_BCKGRND_RETINA	rs2638953	-0.332	0.144	0.021
Hip circumference	Background diabetic retinopathy	ieu-a-55	finn-b-DM_BCKGRND_RETINA	rs2820443	-0.332	0.146	0.022
Hip circumference	Background diabetic retinopathy	ieu-a-55	finn-b-DM_BCKGRND_RETINA	rs3747579	-0.317	0.143	0.027

Hip circumference	Background diabetic retinopathy	ieu-a-55	finn-b-DM_BCKGRND_RETINA	rs3748656	-0.319	0.144	0.026
Hip circumference	Background diabetic retinopathy	ieu-a-55	finn-b-DM_BCKGRND_RETINA	rs3791679	-0.310	0.144	0.032
Hip circumference	Background diabetic retinopathy	ieu-a-55	finn-b-DM_BCKGRND_RETINA	rs42235	-0.324	0.144	0.025
Hip circumference	Background diabetic retinopathy	ieu-a-55	finn-b-DM_BCKGRND_RETINA	rs4243400	-0.330	0.144	0.022
Hip circumference	Background diabetic retinopathy	ieu-a-55	finn-b-DM_BCKGRND_RETINA	rs4246307	-0.324	0.144	0.024
Hip circumference	Background diabetic retinopathy	ieu-a-55	finn-b-DM_BCKGRND_RETINA	rs4369779	-0.311	0.144	0.031
Hip circumference	Background diabetic retinopathy	ieu-a-55	finn-b-DM_BCKGRND_RETINA	rs473902	-0.314	0.145	0.030
Hip circumference	Background diabetic retinopathy	ieu-a-55	finn-b-DM_BCKGRND_RETINA	rs4973517	-0.330	0.144	0.021

Hip circumference	Background diabetic retinopathy	ieu-a-55	finn-b-DM_BCKGRND_RETINA	rs558003	-0.328	0.144	0.022
Hip circumference	Background diabetic retinopathy	ieu-a-55	finn-b-DM_BCKGRND_RETINA	rs561341	-0.318	0.143	0.027
Hip circumference	Background diabetic retinopathy	ieu-a-55	finn-b-DM_BCKGRND_RETINA	rs606452	-0.312	0.144	0.029
Hip circumference	Background diabetic retinopathy	ieu-a-55	finn-b-DM_BCKGRND_RETINA	rs6470764	-0.355	0.144	0.014
Hip circumference	Background diabetic retinopathy	ieu-a-55	finn-b-DM_BCKGRND_RETINA	rs6501392	-0.332	0.143	0.020
Hip circumference	Background diabetic retinopathy	ieu-a-55	finn-b-DM_BCKGRND_RETINA	rs6556079	-0.374	0.144	0.009
Hip circumference	Background diabetic retinopathy	ieu-a-55	finn-b-DM_BCKGRND_RETINA	rs6570509	-0.330	0.145	0.023
Hip circumference	Background diabetic retinopathy	ieu-a-55	finn-b-DM_BCKGRND_RETINA	rs6739772	-0.336	0.143	0.019

Hip circumference	Background diabetic retinopathy	ieu-a-55	finn-b-DM_BCKGRND_RETINA	rs686320	-0.319	0.144	0.026
Hip circumference	Background diabetic retinopathy	ieu-a-55	finn-b-DM_BCKGRND_RETINA	rs7008867	-0.312	0.144	0.030
Hip circumference	Background diabetic retinopathy	ieu-a-55	finn-b-DM_BCKGRND_RETINA	rs7187776	-0.332	0.143	0.021
Hip circumference	Background diabetic retinopathy	ieu-a-55	finn-b-DM_BCKGRND_RETINA	rs7223966	-0.304	0.144	0.034
Hip circumference	Background diabetic retinopathy	ieu-a-55	finn-b-DM_BCKGRND_RETINA	rs724016	-0.300	0.146	0.040
Hip circumference	Background diabetic retinopathy	ieu-a-55	finn-b-DM_BCKGRND_RETINA	rs7759938	-0.349	0.144	0.015
Hip circumference	Background diabetic retinopathy	ieu-a-55	finn-b-DM_BCKGRND_RETINA	rs798497	-0.306	0.144	0.034
Hip circumference	Background diabetic retinopathy	ieu-a-55	finn-b-DM_BCKGRND_RETINA	rs806794	-0.367	0.146	0.012

Hip circumference	Background diabetic retinopathy	ieu-a-55	finn-b-DM_BCKGRND_RETINA	rs849141	-0.328	0.144	0.023
Hip circumference	Background diabetic retinopathy	ieu-a-55	finn-b-DM_BCKGRND_RETINA	rs894345	-0.338	0.143	0.018
Hip circumference	Background diabetic retinopathy	ieu-a-55	finn-b-DM_BCKGRND_RETINA	rs9388766	-0.341	0.144	0.018
Hip circumference	Background diabetic retinopathy	ieu-a-55	finn-b-DM_BCKGRND_RETINA	rs978332	-0.331	0.144	0.021
Hip circumference	Background diabetic retinopathy	ieu-a-55	finn-b-DM_BCKGRND_RETINA	rs9890032	-0.324	0.144	0.024
Hip circumference	Background diabetic retinopathy	ieu-a-55	finn-b-DM_BCKGRND_RETINA	rs991967	-0.322	0.143	0.025
Hip circumference	Background diabetic retinopathy	ieu-a-55	finn-b-DM_BCKGRND_RETINA	rs9993613	-0.332	0.144	0.021
Hip circumference	Background diabetic retinopathy	ieu-a-55	finn-b-DM_BCKGRND_RETINA	All	-0.326	0.143	0.022

Hip circumference	Diabetic retinopathy	ieu-a-55	finn-b-DM_RETINOPATHY_EXMORE	rs10123368	-0.163	0.059	0.006
Hip circumference	Diabetic retinopathy	ieu-a-55	finn-b-DM_RETINOPATHY_EXMORE	rs10140922	-0.159	0.059	0.007
Hip circumference	Diabetic retinopathy	ieu-a-55	finn-b-DM_RETINOPATHY_EXMORE	rs10195252	-0.157	0.058	0.007
Hip circumference	Diabetic retinopathy	ieu-a-55	finn-b-DM_RETINOPATHY_EXMORE	rs1046934	-0.164	0.059	0.005
Hip circumference	Diabetic retinopathy	ieu-a-55	finn-b-DM_RETINOPATHY_EXMORE	rs1053593	-0.167	0.059	0.004
Hip circumference	Diabetic retinopathy	ieu-a-55	finn-b-DM_RETINOPATHY_EXMORE	rs10748128	-0.162	0.059	0.006
Hip circumference	Diabetic retinopathy	ieu-a-55	finn-b-DM_RETINOPATHY_EXMORE	rs10804591	-0.164	0.059	0.005
Hip circumference	Diabetic retinopathy	ieu-a-55	finn-b-DM_RETINOPATHY_EXMORE	rs10950949	-0.163	0.059	0.005

Hip circumference	Diabetic retinopathy	ieu-a-55	finn-b-DM_RETINOPATHY_EXMORE	rs10958476	-0.171	0.058	0.003
Hip circumference	Diabetic retinopathy	ieu-a-55	finn-b-DM_RETINOPATHY_EXMORE	rs11205303	-0.172	0.059	0.004
Hip circumference	Diabetic retinopathy	ieu-a-55	finn-b-DM_RETINOPATHY_EXMORE	rs11242	-0.159	0.059	0.007
Hip circumference	Diabetic retinopathy	ieu-a-55	finn-b-DM_RETINOPATHY_EXMORE	rs11612228	-0.170	0.058	0.003
Hip circumference	Diabetic retinopathy	ieu-a-55	finn-b-DM_RETINOPATHY_EXMORE	rs1173771	-0.160	0.059	0.006
Hip circumference	Diabetic retinopathy	ieu-a-55	finn-b-DM_RETINOPATHY_EXMORE	rs12086130	-0.169	0.058	0.004
Hip circumference	Diabetic retinopathy	ieu-a-55	finn-b-DM_RETINOPATHY_EXMORE	rs12207675	-0.155	0.058	0.008
Hip circumference	Diabetic retinopathy	ieu-a-55	finn-b-DM_RETINOPATHY_EXMORE	rs1254257	-0.172	0.058	0.003

Hip circumference	Diabetic retinopathy	ieu-a-55	finn-b-DM_RETINOPATHY_EXMORE	rs12817549	-0.163	0.059	0.006
Hip circumference	Diabetic retinopathy	ieu-a-55	finn-b-DM_RETINOPATHY_EXMORE	rs1294410	-0.168	0.059	0.004
Hip circumference	Diabetic retinopathy	ieu-a-55	finn-b-DM_RETINOPATHY_EXMORE	rs12980348	-0.162	0.059	0.006
Hip circumference	Diabetic retinopathy	ieu-a-55	finn-b-DM_RETINOPATHY_EXMORE	rs1351394	-0.160	0.059	0.006
Hip circumference	Diabetic retinopathy	ieu-a-55	finn-b-DM_RETINOPATHY_EXMORE	rs1388251	-0.157	0.058	0.007
Hip circumference	Diabetic retinopathy	ieu-a-55	finn-b-DM_RETINOPATHY_EXMORE	rs143384	-0.185	0.058	0.002
Hip circumference	Diabetic retinopathy	ieu-a-55	finn-b-DM_RETINOPATHY_EXMORE	rs1545552	-0.169	0.059	0.004
Hip circumference	Diabetic retinopathy	ieu-a-55	finn-b-DM_RETINOPATHY_EXMORE	rs1662837	-0.170	0.059	0.004

Hip circumference	Diabetic retinopathy	ieu-a-55	finn-b-DM_RETINOPATHY_EXMORE	rs16894959	-0.177	0.058	0.002
Hip circumference	Diabetic retinopathy	ieu-a-55	finn-b-DM_RETINOPATHY_EXMORE	rs169797	-0.159	0.058	0.007
Hip circumference	Diabetic retinopathy	ieu-a-55	finn-b-DM_RETINOPATHY_EXMORE	rs17193922	-0.168	0.058	0.004
Hip circumference	Diabetic retinopathy	ieu-a-55	finn-b-DM_RETINOPATHY_EXMORE	rs1727294	-0.159	0.059	0.007
Hip circumference	Diabetic retinopathy	ieu-a-55	finn-b-DM_RETINOPATHY_EXMORE	rs17346473	-0.173	0.058	0.003
Hip circumference	Diabetic retinopathy	ieu-a-55	finn-b-DM_RETINOPATHY_EXMORE	rs17819328	-0.162	0.059	0.006
Hip circumference	Diabetic retinopathy	ieu-a-55	finn-b-DM_RETINOPATHY_EXMORE	rs1812175	-0.170	0.059	0.004
Hip circumference	Diabetic retinopathy	ieu-a-55	finn-b-DM_RETINOPATHY_EXMORE	rs181553	-0.158	0.059	0.007

Hip circumference	Diabetic retinopathy	ieu-a-55	finn-b-DM_RETINOPATHY_EXMORE	rs2034088	-0.161	0.059	0.006
Hip circumference	Diabetic retinopathy	ieu-a-55	finn-b-DM_RETINOPATHY_EXMORE	rs2098771	-0.167	0.059	0.004
Hip circumference	Diabetic retinopathy	ieu-a-55	finn-b-DM_RETINOPATHY_EXMORE	rs2247341	-0.168	0.059	0.004
Hip circumference	Diabetic retinopathy	ieu-a-55	finn-b-DM_RETINOPATHY_EXMORE	rs2326788	-0.175	0.058	0.002
Hip circumference	Diabetic retinopathy	ieu-a-55	finn-b-DM_RETINOPATHY_EXMORE	rs2377058	-0.166	0.059	0.005
Hip circumference	Diabetic retinopathy	ieu-a-55	finn-b-DM_RETINOPATHY_EXMORE	rs2597513	-0.166	0.059	0.005
Hip circumference	Diabetic retinopathy	ieu-a-55	finn-b-DM_RETINOPATHY_EXMORE	rs2638953	-0.150	0.058	0.009
Hip circumference	Diabetic retinopathy	ieu-a-55	finn-b-DM_RETINOPATHY_EXMORE	rs2820443	-0.143	0.058	0.014

Hip circumference	Diabetic retinopathy	ieu-a-55	finn-b-DM_RETINOPATHY_EXMORE	rs3747579	-0.157	0.058	0.007
Hip circumference	Diabetic retinopathy	ieu-a-55	finn-b-DM_RETINOPATHY_EXMORE	rs3748656	-0.153	0.058	0.008
Hip circumference	Diabetic retinopathy	ieu-a-55	finn-b-DM_RETINOPATHY_EXMORE	rs3791679	-0.161	0.059	0.006
Hip circumference	Diabetic retinopathy	ieu-a-55	finn-b-DM_RETINOPATHY_EXMORE	rs42235	-0.157	0.059	0.007
Hip circumference	Diabetic retinopathy	ieu-a-55	finn-b-DM_RETINOPATHY_EXMORE	rs4243400	-0.165	0.059	0.005
Hip circumference	Diabetic retinopathy	ieu-a-55	finn-b-DM_RETINOPATHY_EXMORE	rs4246307	-0.164	0.059	0.005
Hip circumference	Diabetic retinopathy	ieu-a-55	finn-b-DM_RETINOPATHY_EXMORE	rs4369779	-0.163	0.059	0.006
Hip circumference	Diabetic retinopathy	ieu-a-55	finn-b-DM_RETINOPATHY_EXMORE	rs473902	-0.164	0.059	0.005

Hip circumference	Diabetic retinopathy	ieu-a-55	finn-b-DM_RETINOPATHY_EXMORE	rs4973517	-0.161	0.059	0.006
Hip circumference	Diabetic retinopathy	ieu-a-55	finn-b-DM_RETINOPATHY_EXMORE	rs558003	-0.165	0.059	0.005
Hip circumference	Diabetic retinopathy	ieu-a-55	finn-b-DM_RETINOPATHY_EXMORE	rs561341	-0.159	0.058	0.006
Hip circumference	Diabetic retinopathy	ieu-a-55	finn-b-DM_RETINOPATHY_EXMORE	rs606452	-0.168	0.059	0.004
Hip circumference	Diabetic retinopathy	ieu-a-55	finn-b-DM_RETINOPATHY_EXMORE	rs6470764	-0.155	0.058	0.008
Hip circumference	Diabetic retinopathy	ieu-a-55	finn-b-DM_RETINOPATHY_EXMORE	rs6501392	-0.159	0.058	0.006
Hip circumference	Diabetic retinopathy	ieu-a-55	finn-b-DM_RETINOPATHY_EXMORE	rs6556079	-0.176	0.058	0.002
Hip circumference	Diabetic retinopathy	ieu-a-55	finn-b-DM_RETINOPATHY_EXMORE	rs6570509	-0.180	0.058	0.002

Hip circumference	Diabetic retinopathy	ieu-a-55	finn-b-DM_RETINOPATHY_EXMORE	rs6739772	-0.163	0.059	0.005
Hip circumference	Diabetic retinopathy	ieu-a-55	finn-b-DM_RETINOPATHY_EXMORE	rs686320	-0.162	0.059	0.006
Hip circumference	Diabetic retinopathy	ieu-a-55	finn-b-DM_RETINOPATHY_EXMORE	rs7008867	-0.175	0.058	0.002
Hip circumference	Diabetic retinopathy	ieu-a-55	finn-b-DM_RETINOPATHY_EXMORE	rs7187776	-0.161	0.059	0.006
Hip circumference	Diabetic retinopathy	ieu-a-55	finn-b-DM_RETINOPATHY_EXMORE	rs7223966	-0.163	0.059	0.005
Hip circumference	Diabetic retinopathy	ieu-a-55	finn-b-DM_RETINOPATHY_EXMORE	rs724016	-0.152	0.059	0.010
Hip circumference	Diabetic retinopathy	ieu-a-55	finn-b-DM_RETINOPATHY_EXMORE	rs7759938	-0.171	0.058	0.003
Hip circumference	Diabetic retinopathy	ieu-a-55	finn-b-DM_RETINOPATHY_EXMORE	rs798497	-0.156	0.059	0.008

Hip circumference	Diabetic retinopathy	ieu-a-55	finn-b-DM_RETINOPATHY_EXMORE	rs806794	-0.174	0.059	0.003
Hip circumference	Diabetic retinopathy	ieu-a-55	finn-b-DM_RETINOPATHY_EXMORE	rs849141	-0.167	0.059	0.005
Hip circumference	Diabetic retinopathy	ieu-a-55	finn-b-DM_RETINOPATHY_EXMORE	rs894345	-0.165	0.059	0.005
Hip circumference	Diabetic retinopathy	ieu-a-55	finn-b-DM_RETINOPATHY_EXMORE	rs9388766	-0.154	0.058	0.008
Hip circumference	Diabetic retinopathy	ieu-a-55	finn-b-DM_RETINOPATHY_EXMORE	rs978332	-0.172	0.058	0.003
Hip circumference	Diabetic retinopathy	ieu-a-55	finn-b-DM_RETINOPATHY_EXMORE	rs9890032	-0.168	0.059	0.004
Hip circumference	Diabetic retinopathy	ieu-a-55	finn-b-DM_RETINOPATHY_EXMORE	rs991967	-0.170	0.058	0.004
Hip circumference	Diabetic retinopathy	ieu-a-55	finn-b-DM_RETINOPATHY_EXMORE	rs9993613	-0.158	0.059	0.007

Hip circumference	Diabetic retinopathy	ieu-a-55	finn-b-DM_RETINOPATHY_EXMORE	All	-0.164	0.058	0.005
Hip circumference	Proliferative diabetic retinopathy	ieu-a-55	finn-b-DM_RETINA_PROLIF	rs10123368	-0.159	0.072	0.026
Hip circumference	Proliferative diabetic retinopathy	ieu-a-55	finn-b-DM_RETINA_PROLIF	rs10140922	-0.163	0.072	0.024
Hip circumference	Proliferative diabetic retinopathy	ieu-a-55	finn-b-DM_RETINA_PROLIF	rs10195252	-0.152	0.072	0.035
Hip circumference	Proliferative diabetic retinopathy	ieu-a-55	finn-b-DM_RETINA_PROLIF	rs1046934	-0.161	0.072	0.025
Hip circumference	Proliferative diabetic retinopathy	ieu-a-55	finn-b-DM_RETINA_PROLIF	rs1053593	-0.160	0.072	0.025
Hip circumference	Proliferative diabetic retinopathy	ieu-a-55	finn-b-DM_RETINA_PROLIF	rs10748128	-0.161	0.072	0.025
Hip circumference	Proliferative diabetic retinopathy	ieu-a-55	finn-b-DM_RETINA_PROLIF	rs10804591	-0.159	0.072	0.027

Hip circumference	Proliferative diabetic retinopathy	ieu-a-55	finn-b-DM_RETINA_PROLIF	rs10950949	-0.159	0.072	0.026
Hip circumference	Proliferative diabetic retinopathy	ieu-a-55	finn-b-DM_RETINA_PROLIF	rs10958476	-0.166	0.072	0.021
Hip circumference	Proliferative diabetic retinopathy	ieu-a-55	finn-b-DM_RETINA_PROLIF	rs11205303	-0.166	0.072	0.022
Hip circumference	Proliferative diabetic retinopathy	ieu-a-55	finn-b-DM_RETINA_PROLIF	rs11242	-0.152	0.072	0.035
Hip circumference	Proliferative diabetic retinopathy	ieu-a-55	finn-b-DM_RETINA_PROLIF	rs11612228	-0.163	0.072	0.023
Hip circumference	Proliferative diabetic retinopathy	ieu-a-55	finn-b-DM_RETINA_PROLIF	rs1173771	-0.163	0.072	0.024
Hip circumference	Proliferative diabetic retinopathy	ieu-a-55	finn-b-DM_RETINA_PROLIF	rs12086130	-0.165	0.072	0.022
Hip circumference	Proliferative diabetic retinopathy	ieu-a-55	finn-b-DM_RETINA_PROLIF	rs12207675	-0.147	0.072	0.042

Hip circumference	Proliferative diabetic retinopathy	ieu-a-55	finn-b-DM_RETINA_PROLIF	rs1254257	-0.163	0.072	0.024
Hip circumference	Proliferative diabetic retinopathy	ieu-a-55	finn-b-DM_RETINA_PROLIF	rs12817549	-0.160	0.072	0.026
Hip circumference	Proliferative diabetic retinopathy	ieu-a-55	finn-b-DM_RETINA_PROLIF	rs1294410	-0.156	0.072	0.031
Hip circumference	Proliferative diabetic retinopathy	ieu-a-55	finn-b-DM_RETINA_PROLIF	rs12980348	-0.153	0.072	0.033
Hip circumference	Proliferative diabetic retinopathy	ieu-a-55	finn-b-DM_RETINA_PROLIF	rs1351394	-0.159	0.072	0.027
Hip circumference	Proliferative diabetic retinopathy	ieu-a-55	finn-b-DM_RETINA_PROLIF	rs1388251	-0.155	0.072	0.031
Hip circumference	Proliferative diabetic retinopathy	ieu-a-55	finn-b-DM_RETINA_PROLIF	rs143384	-0.177	0.073	0.015
Hip circumference	Proliferative diabetic retinopathy	ieu-a-55	finn-b-DM_RETINA_PROLIF	rs1545552	-0.164	0.072	0.023

Hip circumference	Proliferative diabetic retinopathy	ieu-a-55	finn-b-DM_RETINA_PROLIF	rs1662837	-0.172	0.072	0.017
Hip circumference	Proliferative diabetic retinopathy	ieu-a-55	finn-b-DM_RETINA_PROLIF	rs16894959	-0.172	0.072	0.017
Hip circumference	Proliferative diabetic retinopathy	ieu-a-55	finn-b-DM_RETINA_PROLIF	rs169797	-0.150	0.072	0.037
Hip circumference	Proliferative diabetic retinopathy	ieu-a-55	finn-b-DM_RETINA_PROLIF	rs17193922	-0.157	0.072	0.029
Hip circumference	Proliferative diabetic retinopathy	ieu-a-55	finn-b-DM_RETINA_PROLIF	rs1727294	-0.159	0.072	0.027
Hip circumference	Proliferative diabetic retinopathy	ieu-a-55	finn-b-DM_RETINA_PROLIF	rs17346473	-0.162	0.072	0.024
Hip circumference	Proliferative diabetic retinopathy	ieu-a-55	finn-b-DM_RETINA_PROLIF	rs17819328	-0.146	0.072	0.042
Hip circumference	Proliferative diabetic retinopathy	ieu-a-55	finn-b-DM_RETINA_PROLIF	rs1812175	-0.163	0.073	0.026

Hip circumference	Proliferative diabetic retinopathy	ieu-a-55	finn-b-DM_RETINA_PROLIF	rs181553	-0.155	0.072	0.031
Hip circumference	Proliferative diabetic retinopathy	ieu-a-55	finn-b-DM_RETINA_PROLIF	rs2034088	-0.161	0.072	0.024
Hip circumference	Proliferative diabetic retinopathy	ieu-a-55	finn-b-DM_RETINA_PROLIF	rs2098771	-0.162	0.072	0.024
Hip circumference	Proliferative diabetic retinopathy	ieu-a-55	finn-b-DM_RETINA_PROLIF	rs2247341	-0.164	0.072	0.023
Hip circumference	Proliferative diabetic retinopathy	ieu-a-55	finn-b-DM_RETINA_PROLIF	rs2326788	-0.161	0.072	0.025
Hip circumference	Proliferative diabetic retinopathy	ieu-a-55	finn-b-DM_RETINA_PROLIF	rs2377058	-0.156	0.072	0.030
Hip circumference	Proliferative diabetic retinopathy	ieu-a-55	finn-b-DM_RETINA_PROLIF	rs2597513	-0.156	0.072	0.030
Hip circumference	Proliferative diabetic retinopathy	ieu-a-55	finn-b-DM_RETINA_PROLIF	rs2638953	-0.150	0.072	0.036

Hip circumference	Proliferative diabetic retinopathy	ieu-a-55	finn-b-DM_RETINA_PROLIF	rs2820443	-0.144	0.073	0.048
Hip circumference	Proliferative diabetic retinopathy	ieu-a-55	finn-b-DM_RETINA_PROLIF	rs3747579	-0.151	0.072	0.035
Hip circumference	Proliferative diabetic retinopathy	ieu-a-55	finn-b-DM_RETINA_PROLIF	rs3748656	-0.150	0.072	0.037
Hip circumference	Proliferative diabetic retinopathy	ieu-a-55	finn-b-DM_RETINA_PROLIF	rs3791679	-0.151	0.072	0.036
Hip circumference	Proliferative diabetic retinopathy	ieu-a-55	finn-b-DM_RETINA_PROLIF	rs42235	-0.149	0.072	0.039
Hip circumference	Proliferative diabetic retinopathy	ieu-a-55	finn-b-DM_RETINA_PROLIF	rs4243400	-0.163	0.072	0.023
Hip circumference	Proliferative diabetic retinopathy	ieu-a-55	finn-b-DM_RETINA_PROLIF	rs4246307	-0.163	0.072	0.023
Hip circumference	Proliferative diabetic retinopathy	ieu-a-55	finn-b-DM_RETINA_PROLIF	rs4369779	-0.155	0.072	0.032

Hip circumference	Proliferative diabetic retinopathy	ieu-a-55	finn-b-DM_RETINA_PROLIF	rs473902	-0.162	0.072	0.025
Hip circumference	Proliferative diabetic retinopathy	ieu-a-55	finn-b-DM_RETINA_PROLIF	rs4973517	-0.157	0.072	0.029
Hip circumference	Proliferative diabetic retinopathy	ieu-a-55	finn-b-DM_RETINA_PROLIF	rs558003	-0.151	0.072	0.036
Hip circumference	Proliferative diabetic retinopathy	ieu-a-55	finn-b-DM_RETINA_PROLIF	rs561341	-0.151	0.072	0.035
Hip circumference	Proliferative diabetic retinopathy	ieu-a-55	finn-b-DM_RETINA_PROLIF	rs606452	-0.162	0.072	0.024
Hip circumference	Proliferative diabetic retinopathy	ieu-a-55	finn-b-DM_RETINA_PROLIF	rs6470764	-0.156	0.072	0.031
Hip circumference	Proliferative diabetic retinopathy	ieu-a-55	finn-b-DM_RETINA_PROLIF	rs6501392	-0.160	0.072	0.026
Hip circumference	Proliferative diabetic retinopathy	ieu-a-55	finn-b-DM_RETINA_PROLIF	rs6556079	-0.176	0.072	0.014

Hip circumference	Proliferative diabetic retinopathy	ieu-a-55	finn-b-DM_RETINA_PROLIF	rs6570509	-0.171	0.073	0.018
Hip circumference	Proliferative diabetic retinopathy	ieu-a-55	finn-b-DM_RETINA_PROLIF	rs6739772	-0.158	0.072	0.027
Hip circumference	Proliferative diabetic retinopathy	ieu-a-55	finn-b-DM_RETINA_PROLIF	rs686320	-0.155	0.072	0.031
Hip circumference	Proliferative diabetic retinopathy	ieu-a-55	finn-b-DM_RETINA_PROLIF	rs7008867	-0.159	0.072	0.027
Hip circumference	Proliferative diabetic retinopathy	ieu-a-55	finn-b-DM_RETINA_PROLIF	rs7187776	-0.155	0.072	0.031
Hip circumference	Proliferative diabetic retinopathy	ieu-a-55	finn-b-DM_RETINA_PROLIF	rs7223966	-0.153	0.072	0.033
Hip circumference	Proliferative diabetic retinopathy	ieu-a-55	finn-b-DM_RETINA_PROLIF	rs724016	-0.139	0.073	0.056
Hip circumference	Proliferative diabetic retinopathy	ieu-a-55	finn-b-DM_RETINA_PROLIF	rs7759938	-0.177	0.072	0.014

Hip circumference	Proliferative diabetic retinopathy	ieu-a-55	finn-b-DM_RETINA_PROLIF	rs798497	-0.164	0.072	0.023
Hip circumference	Proliferative diabetic retinopathy	ieu-a-55	finn-b-DM_RETINA_PROLIF	rs806794	-0.185	0.073	0.011
Hip circumference	Proliferative diabetic retinopathy	ieu-a-55	finn-b-DM_RETINA_PROLIF	rs849141	-0.151	0.072	0.036
Hip circumference	Proliferative diabetic retinopathy	ieu-a-55	finn-b-DM_RETINA_PROLIF	rs894345	-0.158	0.072	0.028
Hip circumference	Proliferative diabetic retinopathy	ieu-a-55	finn-b-DM_RETINA_PROLIF	rs9388766	-0.161	0.072	0.025
Hip circumference	Proliferative diabetic retinopathy	ieu-a-55	finn-b-DM_RETINA_PROLIF	rs978332	-0.169	0.072	0.019
Hip circumference	Proliferative diabetic retinopathy	ieu-a-55	finn-b-DM_RETINA_PROLIF	rs9890032	-0.167	0.072	0.020
Hip circumference	Proliferative diabetic retinopathy	ieu-a-55	finn-b-DM_RETINA_PROLIF	rs991967	-0.165	0.072	0.022

Hip circumference	Proliferative diabetic retinopathy	ieu-a-55	finn-b-DM_RETINA_PROLIF	rs9993613	-0.157	0.072	0.030
Hip circumference	Proliferative diabetic retinopathy	ieu-a-55	finn-b-DM_RETINA_PROLIF	All	-0.159	0.071	0.026
Waist circumference	Background diabetic retinopathy	ieu-a-67	finn-b-DM_BCKGRND_RETINA	rs10041657	-0.242	0.186	0.193
Waist circumference	Background diabetic retinopathy	ieu-a-67	finn-b-DM_BCKGRND_RETINA	rs10748826	-0.243	0.187	0.193
Waist circumference	Background diabetic retinopathy	ieu-a-67	finn-b-DM_BCKGRND_RETINA	rs11144688	-0.246	0.186	0.187
Waist circumference	Background diabetic retinopathy	ieu-a-67	finn-b-DM_BCKGRND_RETINA	rs11205277	-0.207	0.188	0.272
Waist circumference	Background diabetic retinopathy	ieu-a-67	finn-b-DM_BCKGRND_RETINA	rs12207675	-0.183	0.184	0.319
Waist circumference	Background diabetic retinopathy	ieu-a-67	finn-b-DM_BCKGRND_RETINA	rs12317176	-0.229	0.187	0.222

Waist circumference	Background diabetic retinopathy	ieu-a-67	finn-b-DM_BCKGRND_RETINA	rs12330322	-0.249	0.183	0.174
Waist circumference	Background diabetic retinopathy	ieu-a-67	finn-b-DM_BCKGRND_RETINA	rs12493901	-0.219	0.188	0.245
Waist circumference	Background diabetic retinopathy	ieu-a-67	finn-b-DM_BCKGRND_RETINA	rs12608504	-0.198	0.186	0.288
Waist circumference	Background diabetic retinopathy	ieu-a-67	finn-b-DM_BCKGRND_RETINA	rs12656497	-0.182	0.184	0.322
Waist circumference	Background diabetic retinopathy	ieu-a-67	finn-b-DM_BCKGRND_RETINA	rs12679556	-0.234	0.187	0.213
Waist circumference	Background diabetic retinopathy	ieu-a-67	finn-b-DM_BCKGRND_RETINA	rs12700664	-0.229	0.187	0.222
Waist circumference	Background diabetic retinopathy	ieu-a-67	finn-b-DM_BCKGRND_RETINA	rs12991495	-0.223	0.188	0.237
Waist circumference	Background diabetic retinopathy	ieu-a-67	finn-b-DM_BCKGRND_RETINA	rs13083798	-0.259	0.182	0.155

Waist circumference	Background diabetic retinopathy	ieu-a-67	finn-b-DM_BCKGRND_RETINA	rs13210323	-0.236	0.186	0.204
Waist circumference	Background diabetic retinopathy	ieu-a-67	finn-b-DM_BCKGRND_RETINA	rs1344674	-0.191	0.186	0.305
Waist circumference	Background diabetic retinopathy	ieu-a-67	finn-b-DM_BCKGRND_RETINA	rs16957304	-0.196	0.187	0.295
Waist circumference	Background diabetic retinopathy	ieu-a-67	finn-b-DM_BCKGRND_RETINA	rs17451107	-0.226	0.188	0.230
Waist circumference	Background diabetic retinopathy	ieu-a-67	finn-b-DM_BCKGRND_RETINA	rs1776897	-0.184	0.186	0.324
Waist circumference	Background diabetic retinopathy	ieu-a-67	finn-b-DM_BCKGRND_RETINA	rs1812175	-0.186	0.186	0.317
Waist circumference	Background diabetic retinopathy	ieu-a-67	finn-b-DM_BCKGRND_RETINA	rs2047937	-0.231	0.187	0.217
Waist circumference	Background diabetic retinopathy	ieu-a-67	finn-b-DM_BCKGRND_RETINA	rs2052670	-0.200	0.186	0.282

Waist circumference	Background diabetic retinopathy	ieu-a-67	finn-b-DM_BCKGRND_RETINA	rs2071449	-0.271	0.186	0.144
Waist circumference	Background diabetic retinopathy	ieu-a-67	finn-b-DM_BCKGRND_RETINA	rs2124969	-0.225	0.188	0.230
Waist circumference	Background diabetic retinopathy	ieu-a-67	finn-b-DM_BCKGRND_RETINA	rs2160077	-0.231	0.187	0.217
Waist circumference	Background diabetic retinopathy	ieu-a-67	finn-b-DM_BCKGRND_RETINA	rs2179129	-0.236	0.186	0.205
Waist circumference	Background diabetic retinopathy	ieu-a-67	finn-b-DM_BCKGRND_RETINA	rs2214442	-0.178	0.185	0.335
Waist circumference	Background diabetic retinopathy	ieu-a-67	finn-b-DM_BCKGRND_RETINA	rs2274432	-0.257	0.185	0.164
Waist circumference	Background diabetic retinopathy	ieu-a-67	finn-b-DM_BCKGRND_RETINA	rs2638953	-0.227	0.188	0.226
Waist circumference	Background diabetic retinopathy	ieu-a-67	finn-b-DM_BCKGRND_RETINA	rs272869	-0.203	0.187	0.279

Waist circumference	Background diabetic retinopathy	ieu-a-67	finn-b-DM_BCKGRND_RETINA	rs2745353	-0.174	0.186	0.348
Waist circumference	Background diabetic retinopathy	ieu-a-67	finn-b-DM_BCKGRND_RETINA	rs3760318	-0.211	0.188	0.261
Waist circumference	Background diabetic retinopathy	ieu-a-67	finn-b-DM_BCKGRND_RETINA	rs3786897	-0.208	0.187	0.267
Waist circumference	Background diabetic retinopathy	ieu-a-67	finn-b-DM_BCKGRND_RETINA	rs3791679	-0.192	0.188	0.307
Waist circumference	Background diabetic retinopathy	ieu-a-67	finn-b-DM_BCKGRND_RETINA	rs395962	-0.254	0.186	0.173
Waist circumference	Background diabetic retinopathy	ieu-a-67	finn-b-DM_BCKGRND_RETINA	rs4239436	-0.189	0.189	0.316
Waist circumference	Background diabetic retinopathy	ieu-a-67	finn-b-DM_BCKGRND_RETINA	rs4246302	-0.206	0.187	0.272
Waist circumference	Background diabetic retinopathy	ieu-a-67	finn-b-DM_BCKGRND_RETINA	rs4542783	-0.254	0.185	0.169

Waist circumference	Background diabetic retinopathy	ieu-a-67	finn-b-DM_BCKGRND_RETINA	rs4567683	-0.218	0.188	0.245
Waist circumference	Background diabetic retinopathy	ieu-a-67	finn-b-DM_BCKGRND_RETINA	rs459193	-0.194	0.187	0.298
Waist circumference	Background diabetic retinopathy	ieu-a-67	finn-b-DM_BCKGRND_RETINA	rs473902	-0.199	0.189	0.291
Waist circumference	Background diabetic retinopathy	ieu-a-67	finn-b-DM_BCKGRND_RETINA	rs4886782	-0.200	0.187	0.285
Waist circumference	Background diabetic retinopathy	ieu-a-67	finn-b-DM_BCKGRND_RETINA	rs606452	-0.197	0.186	0.292
Waist circumference	Background diabetic retinopathy	ieu-a-67	finn-b-DM_BCKGRND_RETINA	rs6556301	-0.218	0.189	0.249
Waist circumference	Background diabetic retinopathy	ieu-a-67	finn-b-DM_BCKGRND_RETINA	rs6751657	-0.215	0.188	0.252
Waist circumference	Background diabetic retinopathy	ieu-a-67	finn-b-DM_BCKGRND_RETINA	rs6905288	-0.230	0.189	0.223

Waist circumference	Background diabetic retinopathy	ieu-a-67	finn-b-DM_BCKGRND_RETINA	rs710841	-0.224	0.188	0.234
Waist circumference	Background diabetic retinopathy	ieu-a-67	finn-b-DM_BCKGRND_RETINA	rs7430034	-0.224	0.188	0.232
Waist circumference	Background diabetic retinopathy	ieu-a-67	finn-b-DM_BCKGRND_RETINA	rs757608	-0.287	0.180	0.111
Waist circumference	Background diabetic retinopathy	ieu-a-67	finn-b-DM_BCKGRND_RETINA	rs7684221	-0.214	0.187	0.253
Waist circumference	Background diabetic retinopathy	ieu-a-67	finn-b-DM_BCKGRND_RETINA	rs7801581	-0.215	0.188	0.254
Waist circumference	Background diabetic retinopathy	ieu-a-67	finn-b-DM_BCKGRND_RETINA	rs780159	-0.211	0.188	0.262
Waist circumference	Background diabetic retinopathy	ieu-a-67	finn-b-DM_BCKGRND_RETINA	rs7970350	-0.218	0.188	0.245
Waist circumference	Background diabetic retinopathy	ieu-a-67	finn-b-DM_BCKGRND_RETINA	rs798489	-0.183	0.185	0.322

Waist circumference	Background diabetic retinopathy	ieu-a-67	finn-b-DM_BCKGRND_RETINA	rs806794	-0.253	0.187	0.176
Waist circumference	Background diabetic retinopathy	ieu-a-67	finn-b-DM_BCKGRND_RETINA	rs822531	-0.206	0.187	0.271
Waist circumference	Background diabetic retinopathy	ieu-a-67	finn-b-DM_BCKGRND_RETINA	rs849140	-0.215	0.189	0.255
Waist circumference	Background diabetic retinopathy	ieu-a-67	finn-b-DM_BCKGRND_RETINA	rs9389986	-0.218	0.188	0.246
Waist circumference	Background diabetic retinopathy	ieu-a-67	finn-b-DM_BCKGRND_RETINA	rs9435732	-0.243	0.188	0.196
Waist circumference	Background diabetic retinopathy	ieu-a-67	finn-b-DM_BCKGRND_RETINA	rs979012	-0.263	0.186	0.158
Waist circumference	Background diabetic retinopathy	ieu-a-67	finn-b-DM_BCKGRND_RETINA	rs9864077	-0.214	0.188	0.255
Waist circumference	Background diabetic retinopathy	ieu-a-67	finn-b-DM_BCKGRND_RETINA	rs991967	-0.211	0.188	0.263

Waist circumference	Background diabetic retinopathy	ieu-a-67	finn-b-DM_BCKGRND_RETINA	rs9977276	-0.208	0.187	0.266
Waist circumference	Background diabetic retinopathy	ieu-a-67	finn-b-DM_BCKGRND_RETINA	All	-0.219	0.185	0.237
Waist circumference	Diabetic retinopathy	ieu-a-67	finn-b-DM_RETINOPATHY_EXMORE	rs10041657	-0.093	0.075	0.212
Waist circumference	Diabetic retinopathy	ieu-a-67	finn-b-DM_RETINOPATHY_EXMORE	rs10748826	-0.095	0.075	0.207
Waist circumference	Diabetic retinopathy	ieu-a-67	finn-b-DM_RETINOPATHY_EXMORE	rs11144688	-0.098	0.075	0.186
Waist circumference	Diabetic retinopathy	ieu-a-67	finn-b-DM_RETINOPATHY_EXMORE	rs11205277	-0.090	0.076	0.235
Waist circumference	Diabetic retinopathy	ieu-a-67	finn-b-DM_RETINOPATHY_EXMORE	rs12207675	-0.074	0.074	0.320
Waist circumference	Diabetic retinopathy	ieu-a-67	finn-b-DM_RETINOPATHY_EXMORE	rs12317176	-0.098	0.074	0.185

Waist circumference	Diabetic retinopathy	ieu-a-67	finn-b-DM_RETINOPATHY_EXMORE	rs12330322	-0.079	0.075	0.289
Waist circumference	Diabetic retinopathy	ieu-a-67	finn-b-DM_RETINOPATHY_EXMORE	rs12493901	-0.085	0.075	0.258
Waist circumference	Diabetic retinopathy	ieu-a-67	finn-b-DM_RETINOPATHY_EXMORE	rs12608504	-0.075	0.074	0.311
Waist circumference	Diabetic retinopathy	ieu-a-67	finn-b-DM_RETINOPATHY_EXMORE	rs12656497	-0.079	0.075	0.291
Waist circumference	Diabetic retinopathy	ieu-a-67	finn-b-DM_RETINOPATHY_EXMORE	rs12679556	-0.098	0.074	0.186
Waist circumference	Diabetic retinopathy	ieu-a-67	finn-b-DM_RETINOPATHY_EXMORE	rs12700664	-0.088	0.075	0.240
Waist circumference	Diabetic retinopathy	ieu-a-67	finn-b-DM_RETINOPATHY_EXMORE	rs12991495	-0.098	0.075	0.191
Waist circumference	Diabetic retinopathy	ieu-a-67	finn-b-DM_RETINOPATHY_EXMORE	rs13083798	-0.105	0.072	0.147

Waist circumference	Diabetic retinopathy	ieu-a-67	finn-b-DM_RETINOPATHY_EXMORE	rs13210323	-0.083	0.075	0.270
Waist circumference	Diabetic retinopathy	ieu-a-67	finn-b-DM_RETINOPATHY_EXMORE	rs1344674	-0.073	0.074	0.328
Waist circumference	Diabetic retinopathy	ieu-a-67	finn-b-DM_RETINOPATHY_EXMORE	rs16957304	-0.080	0.075	0.286
Waist circumference	Diabetic retinopathy	ieu-a-67	finn-b-DM_RETINOPATHY_EXMORE	rs17451107	-0.079	0.075	0.294
Waist circumference	Diabetic retinopathy	ieu-a-67	finn-b-DM_RETINOPATHY_EXMORE	rs1776897	-0.082	0.076	0.279
Waist circumference	Diabetic retinopathy	ieu-a-67	finn-b-DM_RETINOPATHY_EXMORE	rs1812175	-0.087	0.076	0.250
Waist circumference	Diabetic retinopathy	ieu-a-67	finn-b-DM_RETINOPATHY_EXMORE	rs2047937	-0.085	0.075	0.256
Waist circumference	Diabetic retinopathy	ieu-a-67	finn-b-DM_RETINOPATHY_EXMORE	rs2052670	-0.083	0.075	0.268

Waist circumference	Diabetic retinopathy	ieu-a-67	finn-b-DM_RETINOPATHY_EXMORE	rs2071449	-0.093	0.076	0.219
Waist circumference	Diabetic retinopathy	ieu-a-67	finn-b-DM_RETINOPATHY_EXMORE	rs2124969	-0.092	0.075	0.217
Waist circumference	Diabetic retinopathy	ieu-a-67	finn-b-DM_RETINOPATHY_EXMORE	rs2160077	-0.077	0.074	0.297
Waist circumference	Diabetic retinopathy	ieu-a-67	finn-b-DM_RETINOPATHY_EXMORE	rs2179129	-0.088	0.075	0.240
Waist circumference	Diabetic retinopathy	ieu-a-67	finn-b-DM_RETINOPATHY_EXMORE	rs2214442	-0.084	0.076	0.265
Waist circumference	Diabetic retinopathy	ieu-a-67	finn-b-DM_RETINOPATHY_EXMORE	rs2274432	-0.085	0.076	0.262
Waist circumference	Diabetic retinopathy	ieu-a-67	finn-b-DM_RETINOPATHY_EXMORE	rs2638953	-0.063	0.072	0.381
Waist circumference	Diabetic retinopathy	ieu-a-67	finn-b-DM_RETINOPATHY_EXMORE	rs272869	-0.078	0.075	0.297

Waist circumference	Diabetic retinopathy	ieu-a-67	finn-b-DM_RETINOPATHY_EXMORE	rs2745353	-0.073	0.075	0.328
Waist circumference	Diabetic retinopathy	ieu-a-67	finn-b-DM_RETINOPATHY_EXMORE	rs3760318	-0.090	0.075	0.232
Waist circumference	Diabetic retinopathy	ieu-a-67	finn-b-DM_RETINOPATHY_EXMORE	rs3786897	-0.080	0.075	0.288
Waist circumference	Diabetic retinopathy	ieu-a-67	finn-b-DM_RETINOPATHY_EXMORE	rs3791679	-0.079	0.076	0.297
Waist circumference	Diabetic retinopathy	ieu-a-67	finn-b-DM_RETINOPATHY_EXMORE	rs395962	-0.095	0.075	0.205
Waist circumference	Diabetic retinopathy	ieu-a-67	finn-b-DM_RETINOPATHY_EXMORE	rs4239436	-0.081	0.076	0.287
Waist circumference	Diabetic retinopathy	ieu-a-67	finn-b-DM_RETINOPATHY_EXMORE	rs4246302	-0.079	0.075	0.290
Waist circumference	Diabetic retinopathy	ieu-a-67	finn-b-DM_RETINOPATHY_EXMORE	rs4542783	-0.090	0.075	0.234

Waist circumference	Diabetic retinopathy	ieu-a-67	finn-b-DM_RETINOPATHY_EXMORE	rs4567683	-0.087	0.075	0.248
Waist circumference	Diabetic retinopathy	ieu-a-67	finn-b-DM_RETINOPATHY_EXMORE	rs459193	-0.060	0.072	0.404
Waist circumference	Diabetic retinopathy	ieu-a-67	finn-b-DM_RETINOPATHY_EXMORE	rs473902	-0.083	0.076	0.271
Waist circumference	Diabetic retinopathy	ieu-a-67	finn-b-DM_RETINOPATHY_EXMORE	rs4886782	-0.079	0.075	0.294
Waist circumference	Diabetic retinopathy	ieu-a-67	finn-b-DM_RETINOPATHY_EXMORE	rs606452	-0.091	0.075	0.226
Waist circumference	Diabetic retinopathy	ieu-a-67	finn-b-DM_RETINOPATHY_EXMORE	rs6556301	-0.090	0.076	0.236
Waist circumference	Diabetic retinopathy	ieu-a-67	finn-b-DM_RETINOPATHY_EXMORE	rs6751657	-0.091	0.075	0.226
Waist circumference	Diabetic retinopathy	ieu-a-67	finn-b-DM_RETINOPATHY_EXMORE	rs6905288	-0.100	0.075	0.184

Waist circumference	Diabetic retinopathy	ieu-a-67	finn-b-DM_RETINOPATHY_EXMORE	rs710841	-0.090	0.075	0.231
Waist circumference	Diabetic retinopathy	ieu-a-67	finn-b-DM_RETINOPATHY_EXMORE	rs7430034	-0.097	0.074	0.189
Waist circumference	Diabetic retinopathy	ieu-a-67	finn-b-DM_RETINOPATHY_EXMORE	rs757608	-0.087	0.076	0.249
Waist circumference	Diabetic retinopathy	ieu-a-67	finn-b-DM_RETINOPATHY_EXMORE	rs7684221	-0.075	0.073	0.304
Waist circumference	Diabetic retinopathy	ieu-a-67	finn-b-DM_RETINOPATHY_EXMORE	rs7801581	-0.082	0.075	0.274
Waist circumference	Diabetic retinopathy	ieu-a-67	finn-b-DM_RETINOPATHY_EXMORE	rs780159	-0.094	0.075	0.207
Waist circumference	Diabetic retinopathy	ieu-a-67	finn-b-DM_RETINOPATHY_EXMORE	rs7970350	-0.078	0.075	0.295
Waist circumference	Diabetic retinopathy	ieu-a-67	finn-b-DM_RETINOPATHY_EXMORE	rs798489	-0.077	0.075	0.306

Waist circumference	Diabetic retinopathy	ieu-a-67	finn-b-DM_RETINOPATHY_EXMORE	rs806794	-0.092	0.076	0.225
Waist circumference	Diabetic retinopathy	ieu-a-67	finn-b-DM_RETINOPATHY_EXMORE	rs822531	-0.082	0.075	0.274
Waist circumference	Diabetic retinopathy	ieu-a-67	finn-b-DM_RETINOPATHY_EXMORE	rs849140	-0.085	0.076	0.265
Waist circumference	Diabetic retinopathy	ieu-a-67	finn-b-DM_RETINOPATHY_EXMORE	rs9389986	-0.095	0.075	0.201
Waist circumference	Diabetic retinopathy	ieu-a-67	finn-b-DM_RETINOPATHY_EXMORE	rs9435732	-0.093	0.076	0.219
Waist circumference	Diabetic retinopathy	ieu-a-67	finn-b-DM_RETINOPATHY_EXMORE	rs979012	-0.108	0.074	0.142
Waist circumference	Diabetic retinopathy	ieu-a-67	finn-b-DM_RETINOPATHY_EXMORE	rs9864077	-0.083	0.075	0.268
Waist circumference	Diabetic retinopathy	ieu-a-67	finn-b-DM_RETINOPATHY_EXMORE	rs991967	-0.096	0.075	0.201

Waist circumference	Diabetic retinopathy	ieu-a-67	finn-b-DM_RETINOPATHY_EXMORE	rs9977276	-0.092	0.075	0.216
Waist circumference	Diabetic retinopathy	ieu-a-67	finn-b-DM_RETINOPATHY_EXMORE	All	-0.086	0.074	0.246
Waist circumference	Proliferative diabetic retinopathy	ieu-a-67	finn-b-DM_RETINA_PROLIF	rs10041657	-0.068	0.091	0.457
Waist circumference	Proliferative diabetic retinopathy	ieu-a-67	finn-b-DM_RETINA_PROLIF	rs10748826	-0.062	0.092	0.502
Waist circumference	Proliferative diabetic retinopathy	ieu-a-67	finn-b-DM_RETINA_PROLIF	rs11144688	-0.061	0.092	0.510
Waist circumference	Proliferative diabetic retinopathy	ieu-a-67	finn-b-DM_RETINA_PROLIF	rs11205277	-0.059	0.092	0.525
Waist circumference	Proliferative diabetic retinopathy	ieu-a-67	finn-b-DM_RETINA_PROLIF	rs12207675	-0.039	0.090	0.663
Waist circumference	Proliferative diabetic retinopathy	ieu-a-67	finn-b-DM_RETINA_PROLIF	rs12317176	-0.060	0.092	0.511

Waist circumference	Proliferative diabetic retinopathy	ieu-a-67	finn-b-DM_RETINA_PROLIF	rs12330322	-0.060	0.092	0.511
Waist circumference	Proliferative diabetic retinopathy	ieu-a-67	finn-b-DM_RETINA_PROLIF	rs12493901	-0.058	0.092	0.526
Waist circumference	Proliferative diabetic retinopathy	ieu-a-67	finn-b-DM_RETINA_PROLIF	rs12608504	-0.051	0.092	0.574
Waist circumference	Proliferative diabetic retinopathy	ieu-a-67	finn-b-DM_RETINA_PROLIF	rs12656497	-0.058	0.092	0.526
Waist circumference	Proliferative diabetic retinopathy	ieu-a-67	finn-b-DM_RETINA_PROLIF	rs12679556	-0.068	0.091	0.454
Waist circumference	Proliferative diabetic retinopathy	ieu-a-67	finn-b-DM_RETINA_PROLIF	rs12700664	-0.063	0.091	0.488
Waist circumference	Proliferative diabetic retinopathy	ieu-a-67	finn-b-DM_RETINA_PROLIF	rs12991495	-0.055	0.092	0.548
Waist circumference	Proliferative diabetic retinopathy	ieu-a-67	finn-b-DM_RETINA_PROLIF	rs13083798	-0.072	0.090	0.422

Waist circumference	Proliferative diabetic retinopathy	ieu-a-67	finn-b-DM_RETINA_PROLIF	rs13210323	-0.046	0.091	0.616
Waist circumference	Proliferative diabetic retinopathy	ieu-a-67	finn-b-DM_RETINA_PROLIF	rs1344674	-0.036	0.090	0.687
Waist circumference	Proliferative diabetic retinopathy	ieu-a-67	finn-b-DM_RETINA_PROLIF	rs16957304	-0.036	0.090	0.689
Waist circumference	Proliferative diabetic retinopathy	ieu-a-67	finn-b-DM_RETINA_PROLIF	rs17451107	-0.044	0.091	0.631
Waist circumference	Proliferative diabetic retinopathy	ieu-a-67	finn-b-DM_RETINA_PROLIF	rs1776897	-0.045	0.092	0.621
Waist circumference	Proliferative diabetic retinopathy	ieu-a-67	finn-b-DM_RETINA_PROLIF	rs1812175	-0.055	0.092	0.552
Waist circumference	Proliferative diabetic retinopathy	ieu-a-67	finn-b-DM_RETINA_PROLIF	rs2047937	-0.059	0.092	0.521
Waist circumference	Proliferative diabetic retinopathy	ieu-a-67	finn-b-DM_RETINA_PROLIF	rs2052670	-0.058	0.092	0.530

Waist circumference	Proliferative diabetic retinopathy	ieu-a-67	finn-b-DM_RETINA_PROLIF	rs2071449	-0.071	0.092	0.442
Waist circumference	Proliferative diabetic retinopathy	ieu-a-67	finn-b-DM_RETINA_PROLIF	rs2124969	-0.061	0.092	0.507
Waist circumference	Proliferative diabetic retinopathy	ieu-a-67	finn-b-DM_RETINA_PROLIF	rs2160077	-0.046	0.091	0.612
Waist circumference	Proliferative diabetic retinopathy	ieu-a-67	finn-b-DM_RETINA_PROLIF	rs2179129	-0.057	0.092	0.532
Waist circumference	Proliferative diabetic retinopathy	ieu-a-67	finn-b-DM_RETINA_PROLIF	rs2214442	-0.052	0.092	0.576
Waist circumference	Proliferative diabetic retinopathy	ieu-a-67	finn-b-DM_RETINA_PROLIF	rs2274432	-0.057	0.092	0.536
Waist circumference	Proliferative diabetic retinopathy	ieu-a-67	finn-b-DM_RETINA_PROLIF	rs2638953	-0.041	0.091	0.652
Waist circumference	Proliferative diabetic retinopathy	ieu-a-67	finn-b-DM_RETINA_PROLIF	rs272869	-0.058	0.092	0.530

Waist circumference	Proliferative diabetic retinopathy	ieu-a-67	finn-b-DM_RETINA_PROLIF	rs2745353	-0.021	0.090	0.815
Waist circumference	Proliferative diabetic retinopathy	ieu-a-67	finn-b-DM_RETINA_PROLIF	rs3760318	-0.062	0.092	0.499
Waist circumference	Proliferative diabetic retinopathy	ieu-a-67	finn-b-DM_RETINA_PROLIF	rs3786897	-0.052	0.092	0.573
Waist circumference	Proliferative diabetic retinopathy	ieu-a-67	finn-b-DM_RETINA_PROLIF	rs3791679	-0.041	0.092	0.652
Waist circumference	Proliferative diabetic retinopathy	ieu-a-67	finn-b-DM_RETINA_PROLIF	rs395962	-0.083	0.090	0.360
Waist circumference	Proliferative diabetic retinopathy	ieu-a-67	finn-b-DM_RETINA_PROLIF	rs4239436	-0.045	0.093	0.626
Waist circumference	Proliferative diabetic retinopathy	ieu-a-67	finn-b-DM_RETINA_PROLIF	rs4246302	-0.047	0.091	0.610
Waist circumference	Proliferative diabetic retinopathy	ieu-a-67	finn-b-DM_RETINA_PROLIF	rs4542783	-0.062	0.092	0.498

Waist circumference	Proliferative diabetic retinopathy	ieu-a-67	finn-b-DM_RETINA_PROLIF	rs4567683	-0.057	0.092	0.532
Waist circumference	Proliferative diabetic retinopathy	ieu-a-67	finn-b-DM_RETINA_PROLIF	rs459193	-0.033	0.090	0.714
Waist circumference	Proliferative diabetic retinopathy	ieu-a-67	finn-b-DM_RETINA_PROLIF	rs473902	-0.057	0.093	0.541
Waist circumference	Proliferative diabetic retinopathy	ieu-a-67	finn-b-DM_RETINA_PROLIF	rs4886782	-0.052	0.092	0.574
Waist circumference	Proliferative diabetic retinopathy	ieu-a-67	finn-b-DM_RETINA_PROLIF	rs606452	-0.058	0.092	0.526
Waist circumference	Proliferative diabetic retinopathy	ieu-a-67	finn-b-DM_RETINA_PROLIF	rs6556301	-0.060	0.092	0.514
Waist circumference	Proliferative diabetic retinopathy	ieu-a-67	finn-b-DM_RETINA_PROLIF	rs6751657	-0.059	0.092	0.520
Waist circumference	Proliferative diabetic retinopathy	ieu-a-67	finn-b-DM_RETINA_PROLIF	rs6905288	-0.068	0.092	0.461

Waist circumference	Proliferative diabetic retinopathy	ieu-a-67	finn-b-DM_RETINA_PROLIF	rs710841	-0.066	0.092	0.471
Waist circumference	Proliferative diabetic retinopathy	ieu-a-67	finn-b-DM_RETINA_PROLIF	rs7430034	-0.050	0.091	0.588
Waist circumference	Proliferative diabetic retinopathy	ieu-a-67	finn-b-DM_RETINA_PROLIF	rs757608	-0.065	0.092	0.478
Waist circumference	Proliferative diabetic retinopathy	ieu-a-67	finn-b-DM_RETINA_PROLIF	rs7684221	-0.047	0.091	0.604
Waist circumference	Proliferative diabetic retinopathy	ieu-a-67	finn-b-DM_RETINA_PROLIF	rs7801581	-0.043	0.091	0.635
Waist circumference	Proliferative diabetic retinopathy	ieu-a-67	finn-b-DM_RETINA_PROLIF	rs780159	-0.081	0.090	0.366
Waist circumference	Proliferative diabetic retinopathy	ieu-a-67	finn-b-DM_RETINA_PROLIF	rs7970350	-0.050	0.092	0.582
Waist circumference	Proliferative diabetic retinopathy	ieu-a-67	finn-b-DM_RETINA_PROLIF	rs798489	-0.062	0.092	0.497

Waist circumference	Proliferative diabetic retinopathy	ieu-a-67	finn-b-DM_RETINA_PROLIF	rs806794	-0.077	0.091	0.398
Waist circumference	Proliferative diabetic retinopathy	ieu-a-67	finn-b-DM_RETINA_PROLIF	rs822531	-0.051	0.092	0.580
Waist circumference	Proliferative diabetic retinopathy	ieu-a-67	finn-b-DM_RETINA_PROLIF	rs849140	-0.043	0.092	0.642
Waist circumference	Proliferative diabetic retinopathy	ieu-a-67	finn-b-DM_RETINA_PROLIF	rs9389986	-0.062	0.092	0.500
Waist circumference	Proliferative diabetic retinopathy	ieu-a-67	finn-b-DM_RETINA_PROLIF	rs9435732	-0.051	0.092	0.582
Waist circumference	Proliferative diabetic retinopathy	ieu-a-67	finn-b-DM_RETINA_PROLIF	rs979012	-0.093	0.091	0.306
Waist circumference	Proliferative diabetic retinopathy	ieu-a-67	finn-b-DM_RETINA_PROLIF	rs9864077	-0.065	0.091	0.474
Waist circumference	Proliferative diabetic retinopathy	ieu-a-67	finn-b-DM_RETINA_PROLIF	rs991967	-0.065	0.092	0.480

Waist circumference	Proliferative diabetic retinopathy	ieu-a-67	finn-b-DM_RETINA_PROLIF	rs9977276	-0.058	0.092	0.530
Waist circumference	Proliferative diabetic retinopathy	ieu-a-67	finn-b-DM_RETINA_PROLIF	All	-0.056	0.091	0.535
Waist-to-hip ratio	Background diabetic retinopathy	ieu-a-79	finn-b-DM_BCKGRND_RETINA	rs10245353	-0.036	0.220	0.868
Waist-to-hip ratio	Background diabetic retinopathy	ieu-a-79	finn-b-DM_BCKGRND_RETINA	rs10804591	-0.078	0.217	0.721
Waist-to-hip ratio	Background diabetic retinopathy	ieu-a-79	finn-b-DM_BCKGRND_RETINA	rs10842707	-0.085	0.219	0.698
Waist-to-hip ratio	Background diabetic retinopathy	ieu-a-79	finn-b-DM_BCKGRND_RETINA	rs10991437	-0.058	0.217	0.789
Waist-to-hip ratio	Background diabetic retinopathy	ieu-a-79	finn-b-DM_BCKGRND_RETINA	rs11231693	-0.091	0.218	0.677
Waist-to-hip ratio	Background diabetic retinopathy	ieu-a-79	finn-b-DM_BCKGRND_RETINA	rs1128249	-0.081	0.219	0.712

Waist-to-hip ratio	Background diabetic retinopathy	ieu-a-79	finn-b-DM_BCKGRND_RETINA	rs12143789	-0.066	0.218	0.762
Waist-to-hip ratio	Background diabetic retinopathy	ieu-a-79	finn-b-DM_BCKGRND_RETINA	rs12608504	-0.027	0.218	0.901
Waist-to-hip ratio	Background diabetic retinopathy	ieu-a-79	finn-b-DM_BCKGRND_RETINA	rs12679556	-0.083	0.218	0.704
Waist-to-hip ratio	Background diabetic retinopathy	ieu-a-79	finn-b-DM_BCKGRND_RETINA	rs1294410	-0.046	0.220	0.833
Waist-to-hip ratio	Background diabetic retinopathy	ieu-a-79	finn-b-DM_BCKGRND_RETINA	rs1385167	-0.040	0.218	0.854
Waist-to-hip ratio	Background diabetic retinopathy	ieu-a-79	finn-b-DM_BCKGRND_RETINA	rs1440372	-0.110	0.218	0.613
Waist-to-hip ratio	Background diabetic retinopathy	ieu-a-79	finn-b-DM_BCKGRND_RETINA	rs1569135	-0.084	0.218	0.699
Waist-to-hip ratio	Background diabetic retinopathy	ieu-a-79	finn-b-DM_BCKGRND_RETINA	rs17451107	-0.070	0.218	0.747

Waist-to-hip ratio	Background diabetic retinopathy	ieu-a-79	finn-b-DM_BCKGRND_RETINA	rs17819328	-0.119	0.218	0.585
Waist-to-hip ratio	Background diabetic retinopathy	ieu-a-79	finn-b-DM_BCKGRND_RETINA	rs1936805	0.042	0.224	0.850
Waist-to-hip ratio	Background diabetic retinopathy	ieu-a-79	finn-b-DM_BCKGRND_RETINA	rs2071449	-0.124	0.219	0.570
Waist-to-hip ratio	Background diabetic retinopathy	ieu-a-79	finn-b-DM_BCKGRND_RETINA	rs2294239	-0.095	0.218	0.665
Waist-to-hip ratio	Background diabetic retinopathy	ieu-a-79	finn-b-DM_BCKGRND_RETINA	rs2645294	0.004	0.219	0.984
Waist-to-hip ratio	Background diabetic retinopathy	ieu-a-79	finn-b-DM_BCKGRND_RETINA	rs2820443	-0.077	0.220	0.727
Waist-to-hip ratio	Background diabetic retinopathy	ieu-a-79	finn-b-DM_BCKGRND_RETINA	rs303084	-0.060	0.217	0.782
Waist-to-hip ratio	Background diabetic retinopathy	ieu-a-79	finn-b-DM_BCKGRND_RETINA	rs4081724	-0.035	0.217	0.872

Waist-to-hip ratio	Background diabetic retinopathy	ieu-a-79	finn-b-DM_BCKGRND_RETINA	rs459193	-0.023	0.218	0.917
Waist-to-hip ratio	Background diabetic retinopathy	ieu-a-79	finn-b-DM_BCKGRND_RETINA	rs4646404	-0.053	0.219	0.808
Waist-to-hip ratio	Background diabetic retinopathy	ieu-a-79	finn-b-DM_BCKGRND_RETINA	rs4765219	-0.082	0.219	0.707
Waist-to-hip ratio	Background diabetic retinopathy	ieu-a-79	finn-b-DM_BCKGRND_RETINA	rs6090583	-0.009	0.218	0.965
Waist-to-hip ratio	Background diabetic retinopathy	ieu-a-79	finn-b-DM_BCKGRND_RETINA	rs6772129	-0.046	0.220	0.834
Waist-to-hip ratio	Background diabetic retinopathy	ieu-a-79	finn-b-DM_BCKGRND_RETINA	rs714515	-0.051	0.219	0.817
Waist-to-hip ratio	Background diabetic retinopathy	ieu-a-79	finn-b-DM_BCKGRND_RETINA	rs7705502	-0.012	0.219	0.955
Waist-to-hip ratio	Background diabetic retinopathy	ieu-a-79	finn-b-DM_BCKGRND_RETINA	rs8030605	-0.067	0.217	0.756

Waist-to-hip ratio	Background diabetic retinopathy	ieu-a-79	finn-b-DM_BCKGRND_RETINA	rs8042543	-0.118	0.218	0.588
Waist-to-hip ratio	Background diabetic retinopathy	ieu-a-79	finn-b-DM_BCKGRND_RETINA	rs878639	-0.050	0.218	0.819
Waist-to-hip ratio	Background diabetic retinopathy	ieu-a-79	finn-b-DM_BCKGRND_RETINA	rs905938	-0.074	0.218	0.735
Waist-to-hip ratio	Background diabetic retinopathy	ieu-a-79	finn-b-DM_BCKGRND_RETINA	rs979012	-0.111	0.218	0.612
Waist-to-hip ratio	Background diabetic retinopathy	ieu-a-79	finn-b-DM_BCKGRND_RETINA	rs998584	-0.114	0.224	0.612
Waist-to-hip ratio	Background diabetic retinopathy	ieu-a-79	finn-b-DM_BCKGRND_RETINA	rs9991328	-0.066	0.217	0.762
Waist-to-hip ratio	Background diabetic retinopathy	ieu-a-79	finn-b-DM_BCKGRND_RETINA	All	-0.064	0.215	0.767
Waist-to-hip ratio	Diabetic retinopathy	ieu-a-79	finn-b-DM_RETINOPATHY_EXMORE	rs10245353	0.142	0.109	0.195

Waist-to-hip ratio	Diabetic retinopathy	ieu-a-79	finn-b-DM_RETINOPATHY_EXMORE	rs10804591	0.134	0.108	0.213
Waist-to-hip ratio	Diabetic retinopathy	ieu-a-79	finn-b-DM_RETINOPATHY_EXMORE	rs10842707	0.107	0.106	0.311
Waist-to-hip ratio	Diabetic retinopathy	ieu-a-79	finn-b-DM_RETINOPATHY_EXMORE	rs10991437	0.126	0.107	0.238
Waist-to-hip ratio	Diabetic retinopathy	ieu-a-79	finn-b-DM_RETINOPATHY_EXMORE	rs11231693	0.130	0.108	0.229
Waist-to-hip ratio	Diabetic retinopathy	ieu-a-79	finn-b-DM_RETINOPATHY_EXMORE	rs1128249	0.119	0.108	0.270
Waist-to-hip ratio	Diabetic retinopathy	ieu-a-79	finn-b-DM_RETINOPATHY_EXMORE	rs12143789	0.140	0.108	0.197
Waist-to-hip ratio	Diabetic retinopathy	ieu-a-79	finn-b-DM_RETINOPATHY_EXMORE	rs12608504	0.159	0.105	0.130
Waist-to-hip ratio	Diabetic retinopathy	ieu-a-79	finn-b-DM_RETINOPATHY_EXMORE	rs12679556	0.123	0.107	0.252

Waist-to-hip ratio	Diabetic retinopathy	ieu-a-79	finn-b-DM_RETINOPATHY_EXMORE	rs1294410	0.144	0.109	0.187
Waist-to-hip ratio	Diabetic retinopathy	ieu-a-79	finn-b-DM_RETINOPATHY_EXMORE	rs1385167	0.126	0.108	0.242
Waist-to-hip ratio	Diabetic retinopathy	ieu-a-79	finn-b-DM_RETINOPATHY_EXMORE	rs1440372	0.151	0.107	0.156
Waist-to-hip ratio	Diabetic retinopathy	ieu-a-79	finn-b-DM_RETINOPATHY_EXMORE	rs1569135	0.135	0.108	0.214
Waist-to-hip ratio	Diabetic retinopathy	ieu-a-79	finn-b-DM_RETINOPATHY_EXMORE	rs17451107	0.153	0.107	0.154
Waist-to-hip ratio	Diabetic retinopathy	ieu-a-79	finn-b-DM_RETINOPATHY_EXMORE	rs17819328	0.131	0.108	0.225
Waist-to-hip ratio	Diabetic retinopathy	ieu-a-79	finn-b-DM_RETINOPATHY_EXMORE	rs1936805	0.181	0.108	0.094
Waist-to-hip ratio	Diabetic retinopathy	ieu-a-79	finn-b-DM_RETINOPATHY_EXMORE	rs2071449	0.135	0.109	0.215

Waist-to-hip ratio	Diabetic retinopathy	ieu-a-79	finn-b-DM_RETINOPATHY_EXMORE	rs2294239	0.140	0.109	0.196
Waist-to-hip ratio	Diabetic retinopathy	ieu-a-79	finn-b-DM_RETINOPATHY_EXMORE	rs2645294	0.147	0.109	0.176
Waist-to-hip ratio	Diabetic retinopathy	ieu-a-79	finn-b-DM_RETINOPATHY_EXMORE	rs2820443	0.097	0.105	0.354
Waist-to-hip ratio	Diabetic retinopathy	ieu-a-79	finn-b-DM_RETINOPATHY_EXMORE	rs303084	0.130	0.107	0.226
Waist-to-hip ratio	Diabetic retinopathy	ieu-a-79	finn-b-DM_RETINOPATHY_EXMORE	rs4081724	0.149	0.106	0.161
Waist-to-hip ratio	Diabetic retinopathy	ieu-a-79	finn-b-DM_RETINOPATHY_EXMORE	rs459193	0.182	0.097	0.062
Waist-to-hip ratio	Diabetic retinopathy	ieu-a-79	finn-b-DM_RETINOPATHY_EXMORE	rs4646404	0.133	0.109	0.222
Waist-to-hip ratio	Diabetic retinopathy	ieu-a-79	finn-b-DM_RETINOPATHY_EXMORE	rs4765219	0.119	0.107	0.269

Waist-to-hip ratio	Diabetic retinopathy	ieu-a-79	finn-b-DM_RETINOPATHY_EXMORE	rs6090583	0.128	0.108	0.235
Waist-to-hip ratio	Diabetic retinopathy	ieu-a-79	finn-b-DM_RETINOPATHY_EXMORE	rs6772129	0.152	0.109	0.164
Waist-to-hip ratio	Diabetic retinopathy	ieu-a-79	finn-b-DM_RETINOPATHY_EXMORE	rs714515	0.142	0.109	0.191
Waist-to-hip ratio	Diabetic retinopathy	ieu-a-79	finn-b-DM_RETINOPATHY_EXMORE	rs7705502	0.136	0.109	0.211
Waist-to-hip ratio	Diabetic retinopathy	ieu-a-79	finn-b-DM_RETINOPATHY_EXMORE	rs8030605	0.136	0.108	0.208
Waist-to-hip ratio	Diabetic retinopathy	ieu-a-79	finn-b-DM_RETINOPATHY_EXMORE	rs8042543	0.122	0.107	0.256
Waist-to-hip ratio	Diabetic retinopathy	ieu-a-79	finn-b-DM_RETINOPATHY_EXMORE	rs878639	0.158	0.105	0.131
Waist-to-hip ratio	Diabetic retinopathy	ieu-a-79	finn-b-DM_RETINOPATHY_EXMORE	rs905938	0.151	0.107	0.157

Waist-to-hip ratio	Diabetic retinopathy	ieu-a-79	finn-b-DM_RETINOPATHY_EXMORE	rs979012	0.116	0.107	0.275
Waist-to-hip ratio	Diabetic retinopathy	ieu-a-79	finn-b-DM_RETINOPATHY_EXMORE	rs998584	0.128	0.112	0.250
Waist-to-hip ratio	Diabetic retinopathy	ieu-a-79	finn-b-DM_RETINOPATHY_EXMORE	rs9991328	0.108	0.100	0.280
Waist-to-hip ratio	Diabetic retinopathy	ieu-a-79	finn-b-DM_RETINOPATHY_EXMORE	All	0.136	0.106	0.197
Waist-to-hip ratio	Proliferative diabetic retinopathy	ieu-a-79	finn-b-DM_RETINA_PROLIF	rs10245353	0.166	0.147	0.260
Waist-to-hip ratio	Proliferative diabetic retinopathy	ieu-a-79	finn-b-DM_RETINA_PROLIF	rs10804591	0.157	0.145	0.279
Waist-to-hip ratio	Proliferative diabetic retinopathy	ieu-a-79	finn-b-DM_RETINA_PROLIF	rs10842707	0.127	0.144	0.378
Waist-to-hip ratio	Proliferative diabetic retinopathy	ieu-a-79	finn-b-DM_RETINA_PROLIF	rs10991437	0.147	0.144	0.308

Waist-to-hip ratio	Proliferative diabetic retinopathy	ieu-a-79	finn-b-DM_RETINA_PROLIF	rs11231693	0.162	0.146	0.265
Waist-to-hip ratio	Proliferative diabetic retinopathy	ieu-a-79	finn-b-DM_RETINA_PROLIF	rs1128249	0.139	0.145	0.338
Waist-to-hip ratio	Proliferative diabetic retinopathy	ieu-a-79	finn-b-DM_RETINA_PROLIF	rs12143789	0.171	0.145	0.238
Waist-to-hip ratio	Proliferative diabetic retinopathy	ieu-a-79	finn-b-DM_RETINA_PROLIF	rs12608504	0.171	0.145	0.239
Waist-to-hip ratio	Proliferative diabetic retinopathy	ieu-a-79	finn-b-DM_RETINA_PROLIF	rs12679556	0.146	0.145	0.316
Waist-to-hip ratio	Proliferative diabetic retinopathy	ieu-a-79	finn-b-DM_RETINA_PROLIF	rs1294410	0.150	0.147	0.307
Waist-to-hip ratio	Proliferative diabetic retinopathy	ieu-a-79	finn-b-DM_RETINA_PROLIF	rs1385167	0.154	0.146	0.292
Waist-to-hip ratio	Proliferative diabetic retinopathy	ieu-a-79	finn-b-DM_RETINA_PROLIF	rs1440372	0.177	0.144	0.217

Waist-to-hip ratio	Proliferative diabetic retinopathy	ieu-a-79	finn-b-DM_RETINA_PROLIF	rs1569135	0.143	0.144	0.321
Waist-to-hip ratio	Proliferative diabetic retinopathy	ieu-a-79	finn-b-DM_RETINA_PROLIF	rs17451107	0.183	0.144	0.204
Waist-to-hip ratio	Proliferative diabetic retinopathy	ieu-a-79	finn-b-DM_RETINA_PROLIF	rs17819328	0.131	0.141	0.354
Waist-to-hip ratio	Proliferative diabetic retinopathy	ieu-a-79	finn-b-DM_RETINA_PROLIF	rs1936805	0.254	0.138	0.066
Waist-to-hip ratio	Proliferative diabetic retinopathy	ieu-a-79	finn-b-DM_RETINA_PROLIF	rs2071449	0.148	0.146	0.312
Waist-to-hip ratio	Proliferative diabetic retinopathy	ieu-a-79	finn-b-DM_RETINA_PROLIF	rs2294239	0.167	0.146	0.252
Waist-to-hip ratio	Proliferative diabetic retinopathy	ieu-a-79	finn-b-DM_RETINA_PROLIF	rs2645294	0.207	0.140	0.141
Waist-to-hip ratio	Proliferative diabetic retinopathy	ieu-a-79	finn-b-DM_RETINA_PROLIF	rs2820443	0.130	0.146	0.371

Waist-to-hip ratio	Proliferative diabetic retinopathy	ieu-a-79	finn-b-DM_RETINA_PROLIF	rs303084	0.142	0.143	0.321
Waist-to-hip ratio	Proliferative diabetic retinopathy	ieu-a-79	finn-b-DM_RETINA_PROLIF	rs4081724	0.178	0.143	0.212
Waist-to-hip ratio	Proliferative diabetic retinopathy	ieu-a-79	finn-b-DM_RETINA_PROLIF	rs459193	0.200	0.139	0.151
Waist-to-hip ratio	Proliferative diabetic retinopathy	ieu-a-79	finn-b-DM_RETINA_PROLIF	rs4646404	0.138	0.145	0.341
Waist-to-hip ratio	Proliferative diabetic retinopathy	ieu-a-79	finn-b-DM_RETINA_PROLIF	rs4765219	0.155	0.146	0.289
Waist-to-hip ratio	Proliferative diabetic retinopathy	ieu-a-79	finn-b-DM_RETINA_PROLIF	rs6090583	0.141	0.144	0.327
Waist-to-hip ratio	Proliferative diabetic retinopathy	ieu-a-79	finn-b-DM_RETINA_PROLIF	rs6772129	0.146	0.147	0.320
Waist-to-hip ratio	Proliferative diabetic retinopathy	ieu-a-79	finn-b-DM_RETINA_PROLIF	rs714515	0.179	0.145	0.218

Waist-to-hip ratio	Proliferative diabetic retinopathy	ieu-a-79	finn-b-DM_RETINA_PROLIF	rs7705502	0.184	0.145	0.204
Waist-to-hip ratio	Proliferative diabetic retinopathy	ieu-a-79	finn-b-DM_RETINA_PROLIF	rs8030605	0.158	0.145	0.277
Waist-to-hip ratio	Proliferative diabetic retinopathy	ieu-a-79	finn-b-DM_RETINA_PROLIF	rs8042543	0.126	0.141	0.372
Waist-to-hip ratio	Proliferative diabetic retinopathy	ieu-a-79	finn-b-DM_RETINA_PROLIF	rs878639	0.172	0.145	0.235
Waist-to-hip ratio	Proliferative diabetic retinopathy	ieu-a-79	finn-b-DM_RETINA_PROLIF	rs905938	0.164	0.146	0.261
Waist-to-hip ratio	Proliferative diabetic retinopathy	ieu-a-79	finn-b-DM_RETINA_PROLIF	rs979012	0.122	0.141	0.387
Waist-to-hip ratio	Proliferative diabetic retinopathy	ieu-a-79	finn-b-DM_RETINA_PROLIF	rs998584	0.167	0.150	0.266
Waist-to-hip ratio	Proliferative diabetic retinopathy	ieu-a-79	finn-b-DM_RETINA_PROLIF	rs9991328	0.127	0.138	0.356

Waist-to-hip ratio	Proliferative diabetic retinopathy	ieu-a-79	finn-b-DM_RETINA_PROLIF	All	0.159	0.142	0.264
